# Development of a nitrogen-bound hydrophobic auxiliary: application to solid/hydrophobic-tag relay synthesis of calpinactam†

**DOI:** 10.1039/d3sc01432k

**Published:** 2023-05-01

**Authors:** Hiroki Nakahara, Goh Sennari, Yoshihiko Noguchi, Tomoyasu Hirose, Toshiaki Sunazuka

**Affiliations:** a Ōmura Satoshi Memorial Institute and Graduate School of Infection Control Sciences, Kitasato University 5-9-1 Shirokane, Minato-ku Tokyo 108-8641 Japan thirose@lisci.kitasato-u.ac.jp sunazuka@lisci.kitasato-u.ac.jp

## Abstract

In the last couple of decades, technologies and strategies for peptide synthesis have advanced rapidly. Although solid-phase peptide synthesis (SPPS) and liquid-phase peptide synthesis (LPPS) have contributed significantly to the development of the field, there have been remaining challenges for C-terminal modifications of peptide compounds in SPPS and LPPS. Orthogonal to the current standard approach that relies on installation of a carrier molecule at the C-terminus of amino acids, we developed a new hydrophobic-tag carbonate reagent which facilitated robust preparation of nitrogen-tag-supported peptide compounds. This auxiliary was easily installed on a variety of amino acids including oligopeptides that have a broad range of noncanonical residues, allowing simple purification of the products by crystallization and filtration. We demonstrated a *de novo* solid/hydrophobic-tag relay synthesis (STRS) strategy using the nitrogen-bound auxiliary for total synthesis of calpinactam.

## Abstract 2

Development of a new hydrophobic auxiliary, TCbz group at the N-terminus of peptides is disclosed. This auxiliary enabled C-terminal modification in LPPS and a *de novo* solid/hydrophobic-tag relay synthesis (STRS) of calpinactam.

## Graphical abstract

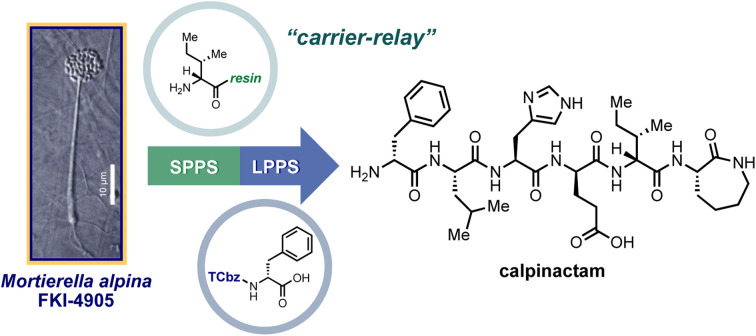

## Introduction

Peptide compounds are an important class in the research fields of biological, medical, and pharmaceutical sciences.^1^ Because of the increasing needs for peptides as therapeutics,^2^ methodologies including strategic developments for peptide synthesis have become one of the top interests in the community.^3^ A major challenge in classical solution peptide synthesis (CSPS)^4^ is manipulation and purification of compounds that possess inherent polarity as well as solubility.^5^ Because amino acids representing zwitterionic compounds^6^ have polar and/or non-polar side chains at the α-position of a carboxyl group, the traditional synthetic approach has been difficult to adapt especially for polypeptide syntheses.^5^

**Fig. 1:**
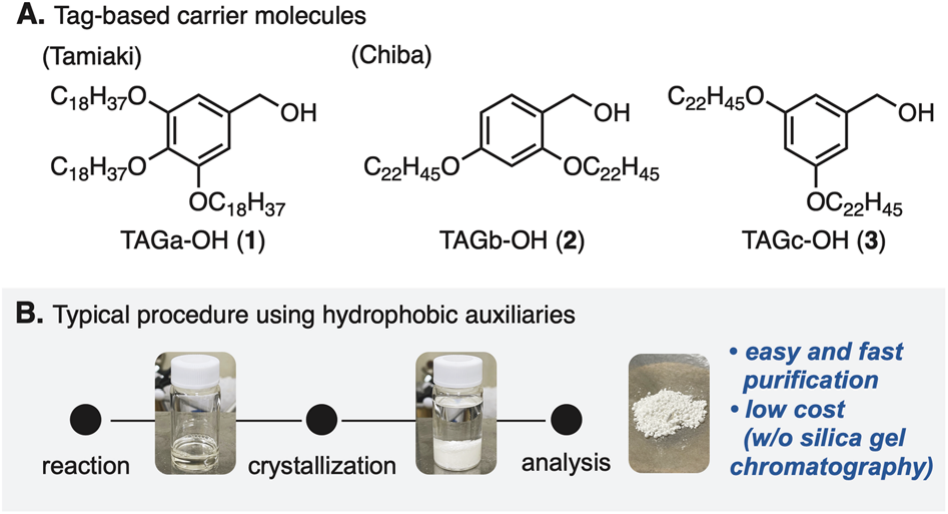
Hydrophobic tags and a flowchart for a typical purification procedure.

In 1963, Merrifield and co-workers developed a complementary approach,^7^ so-called solid-phase peptide synthesis (SPPS).^8^ This approach enabled robust manipulation of peptide compounds by attaching the carboxyl group of an amino acid to the solid-phase, overcoming a bottleneck of the CSPS method. However, due to the nature of SPPS that iterates peptide elongation without chromatographic purification, undesired side reactions attributed to the purities of intermediates are sometimes problematic.^9^ In addition, SPPS generally requires excess amounts of reactants and reagents, because of heterogeneous reaction circumstances. In 2001, a pioneering study by Tamiaki and co-workers for tag-based peptide chemistry in liquid-phase peptide synthesis (LPPS)^10^ demonstrated that 3,4,5-tris(octadecyloxy)benzyl alcohol (TAGa-OH: 1, Fig. 1A) could be used as a soluble carrier molecule for a novel method that combines the advantages of SPPS and CSPS.^11^ Later, Chiba and co-workers further developed this method^12^ by changing the alkoxy groups (TAGb-OH: 2 and TAGc-OH: 3) to allow application to Fmoc- and Boc-protected LPPS.^13^ In this approach, because tag-installed amino acids are soluble in various organic solvents (*e.g.* DCM, THF, and PhMe) but insoluble in polar solvents (*e.g.* MeOH and MeCN), reactions can be operated in a similar manner to CSPS while simple purification processes similar to those in SPPS by washing reagents off after crystallization are applicable (Fig. 1B). This powerful LPPS strategy has been adapted to natural product syntheses.^14^

**Scheme 1:**
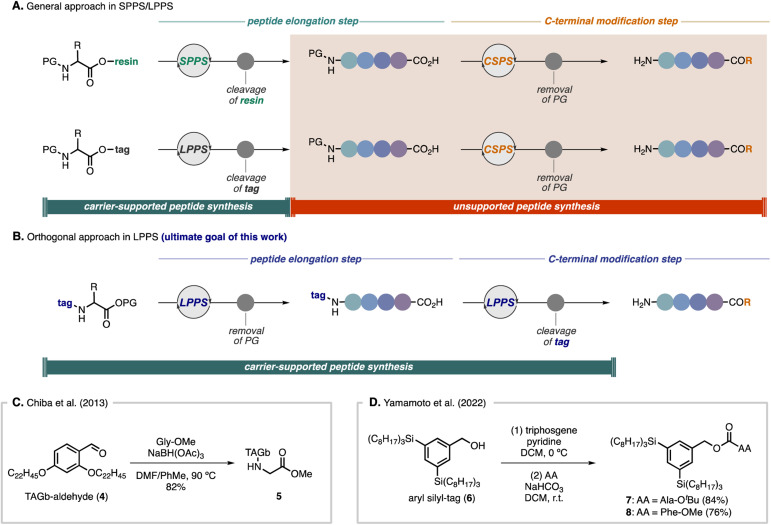
Strategies in SPPS and LPPS.

Although SPPS and tag-based LPPS have been used as robust and general strategies for peptide synthesis,^15^ there are challenges for C-terminal modifications. Because current strategies generally rely on the introduction of a carrier molecule to the carboxyl group of an amino acid, when C-terminal modifications are required, it is indispensable to handle free amino acids by CSPS after removal of the carrier molecule (Scheme 1A).^16^ We envisioned an orthogonal approach in which a hydrophobic-tag auxiliary would be installed on the amine group of an amino acid, enabling direct C-terminal peptide elongation and modifications in LPPS (Scheme 1B). There are a few limited examples of utilizing nitrogen-bound tag peptides for LPPS. In Chiba's total synthesis of mahafacyclin B,^14*c*^ the TAGb-based carrier was installed on Gly-OMe by reductive amination with aldehyde 4 to afford *N*-alkyl-tag compound 5 in 82% yield (Scheme 1C). Recently, Yamamoto and co-workers showcased two preliminary examples to install their silicon-based hydrophobic tag (6) to the nitrogen atom of amino acids *via* the corresponding chloroformate by treatment with triphosgene, providing carbamate-tag compounds 7 (84% yield) and 8 (76% yield), respectively (Scheme 1D).^17^ In both cases, chromatographic purification was conducted after introduction of the tags to the amino acids. In general, for tag-based LPPS, because installation of the carrier molecule to the carboxyl group requires activation of the acid moiety, side products derived from the amino acid can be easily separated by dissolving in polar solvents as filtrates, whereas, introducing hydrophobic auxiliaries to the amine group involving activation of the carrier molecule often results in difficulties in removing carrier-derived side products by only crystallization and filtration.^14*c*,*g*,17^ In this regard, the key to success in employing this approach by simple operations would be the strict control of the reactivity, which allows activation of the tag carrier molecule without any side reactions but being reactive enough with amino acids under mild conditions. Therefore, we sought to explore versatile activating groups to install a hydrophobic-tag on the nitrogen atom and to develop broadly applicable strategies for a new direction in LPPS.

Herein, we describe the development of a novel tag-carbonate, which enabled robust introduction of a hydrophobic auxiliary to the nitrogen atom of various amino acids by simple operations. Tag-supported peptides showed practical properties in LPPS and are tolerant to a variety of deprotection conditions, prompting us to develop a *de novo* peptide synthetic strategy. We applied this tag-based chemistry to “solid/hydrophobic-tag relay synthesis (STRS)” of the natural product, calpinactam.

## Results and discussion

### Development of TCbz-OAr_F_

**Scheme 2:**
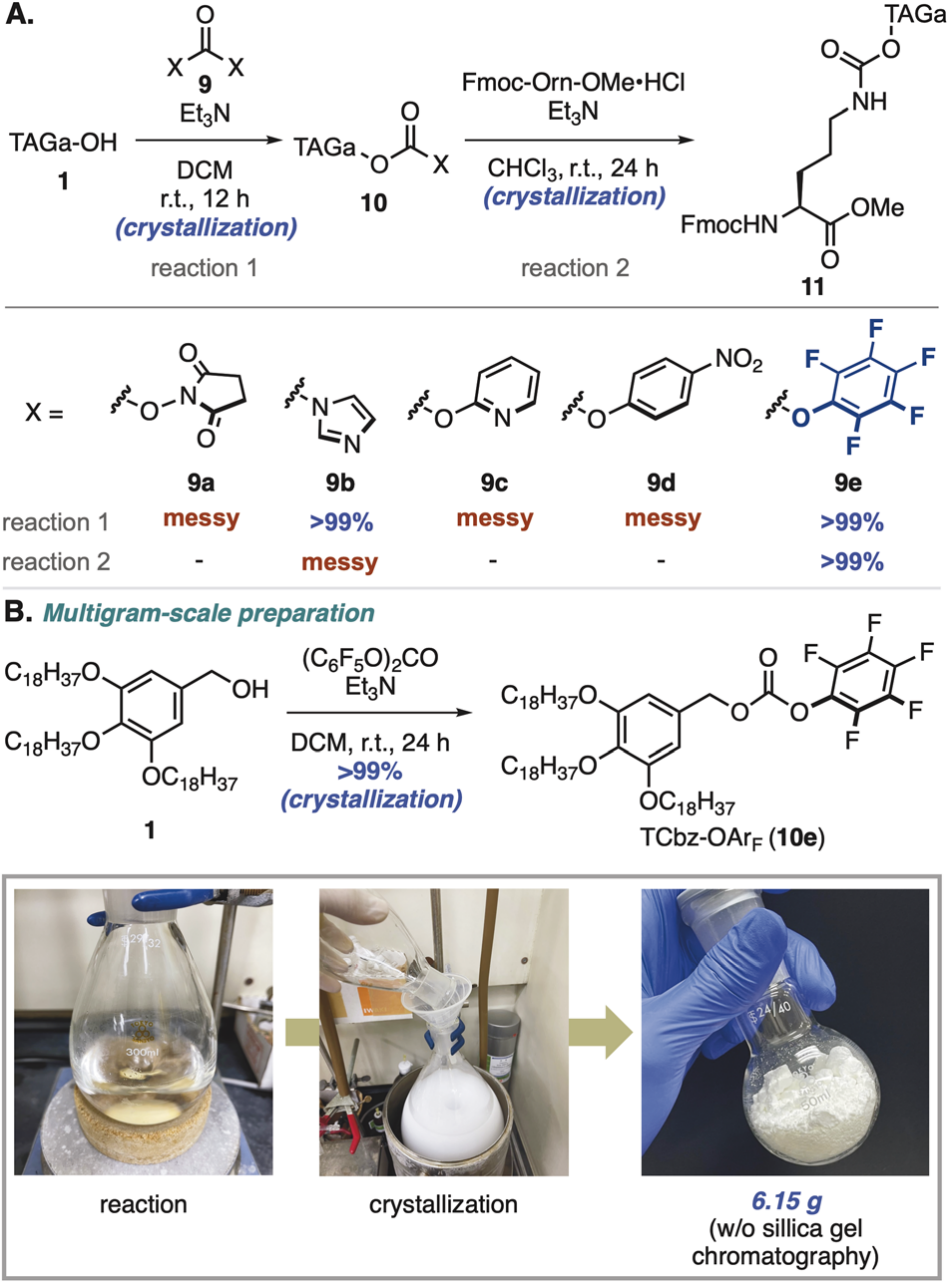
Development of the TCbz-OAr_F_ reagent.

To install hydrophobic-tag auxiliaries on the nitrogen atom of amino acids, we initially thought to utilize general protecting groups for peptides^18^ and turned our attention to the carbamate group (Scheme 2A).^19^ Our investigation began with exploration of activating groups^20^ of TAGa-OH (1), and common condensation reagents such as *N*,*N*′-disuccinimidyl carbonate (DSC, 9a)^21^ and *N*,*N*′-carbonyldiimidazole (CDI, 9b)^22^ were employed. While treatment of 1 with DSC gave a messy reaction mixture, using CDI as an activating reagent provided the desired 10b in quantitative yield by simple crystallization. Unfortunately, condensation of 10b with the primary amine of Fmoc-Orn-OMe was not effective under several conditions including *N*-methylation of the imidazole moiety (see the ESI, Table S2†).

After screening of other activating groups such as bis(2-pyridine)carbonate (9c)^23^ and bis(4-nitrophenyl)carbonate (9d),^24^ we found that treatment with bis(pentafluorophenyl)carbonate (9e)^25^ gave the desired tagged-Cbz-*O*-pentafluorobenzene (TCbz-OAr_F_, 10e) in quantitative yield by simple crystallization. To our delight, pentafluorophenol was an effective leaving group for the subsequent coupling reaction with the amine in the presence of Et_3_N at room temperature, providing 11 in quantitative yield by crystallization. Based on this result, we could prepare TCbz-OAr_F_ (10e) on a multigram-scale by simple crystallization-filtration operations (Scheme 2B). During the completion of this work, Yamamoto and co-workers reported a similar carbamate-type auxiliary which was installed by treatment of a silyl-tag alcohol with triphosgene.^17^ It is noteworthy that our method using 10e as a tethering reagent enabled introduction of the carrier *via* the carbamate linkage without chromatographic purification in a simple and scalable manner.

### Introduction of the TCbz group to various amino acids

**Scheme 3:**
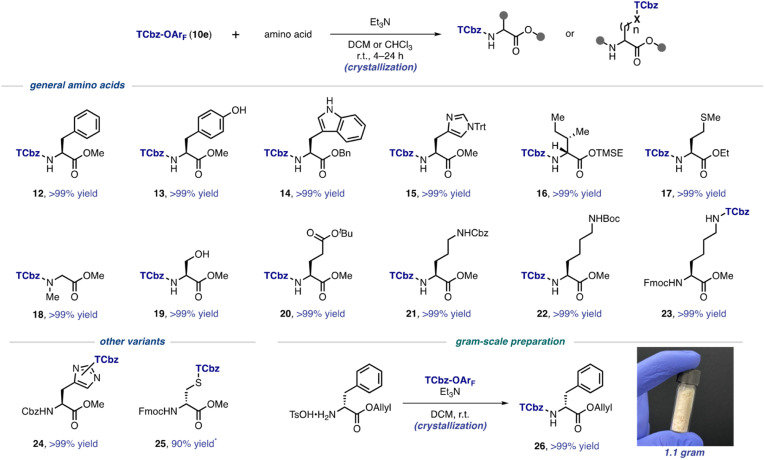
Substrate scope for installation of the TCbz group. * isolated yield using preparative TLC.

With the optimal conditions to install the TCbz group in hand, we investigated the substrate generality using various amino acids (Scheme 3). First, aromatic amino acids were employed for the TCbz-introduction to the amine group to give the desired product (12–15) in quantitative yield by the typical crystallization purification procedure. The phenolic hydroxy group and the free indole nitrogen were intact under the conditions. The amine groups of aliphatic amino acids possessing the trimethylsilyl ethyl (TMSE) ester^26^ and sulphide group were effectively reacted, affording the corresponding products (16 and 17) quantitatively. *N*-Methyl and hydrophilic amino acids were also tolerant under the conditions, providing 18–20 without events by crystallization, even though the hydroxyl group in serine was unprotected.

We showcased that the TCbz group could be installed either on the N-terminus or side chain when using basic amino acids (see 21–23). The amine-protections with Cbz, Boc, and Fmoc groups were compatible under the conditions. In particular, installation of the TCbz group on the amines in the side chain of ornithine (see 11, Scheme 2A) and lysine (see 23) would allow both C → N and N → C elongation strategies for LPPS by selective deprotection of the amine or acid.

We were also interested in other variants to utilize TCbz-OAr_F_ (10e) because of its property of acting as a versatile electrophile under very mild conditions. In this regard, 10e was treated with amine-protected amino acids which have a nucleophilic functional group in the molecule. The nucleophilic imidazole in histidine was smoothly reacted with 10e to provide 24 in quantitative yield as a 1.3 : 1 mixture of the constitutional isomers. The thiol group in cysteine was capable of effecting nucleophilic addition into 10e, affording 25 in 90% yield after chromatographic purification to remove impurities in this case. Unfortunately, further attempts using these compounds (24 and 25) for LPPS revealed that the TCbz groups were not tolerant under deprotection or condensation conditions. However, it is worth noting that this auxiliary might be useful to help purify polar peptides containing these amino acid residues as a catch and release protocol.^27^ Lastly, we demonstrated gram-scale preparation of TCbz-D-Phe-OAllyl (26) under the optimal conditions in quantitative yield by simple crystallization purification.

### TCbz-cleavage and deprotection of amino acids

**Scheme 4:**
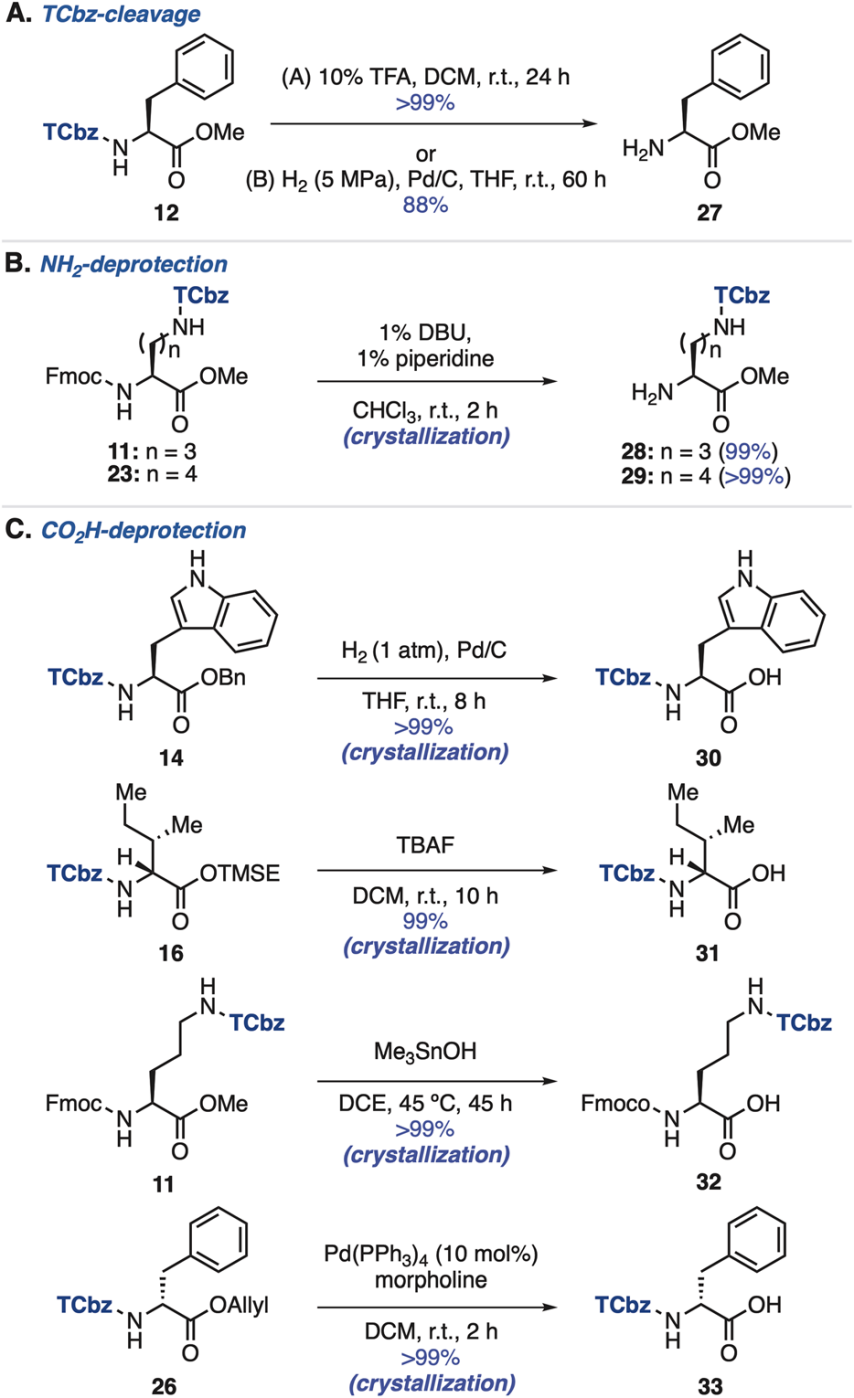
Removal of the TCbz group and protecting groups.

We then investigated removal of the TCbz group from amino acids (see the ESI, Table S3†). As a result, treatment of 12 with 10% TFA in DCM at room temperature cleanly cleaved the TCbz group to afford the TFA salt of 27 quantitatively. Alternatively, hydrogenolysis^28^ under pressurized conditions was also effective, providing 27 in 88% yield. In both cases, the pure material was obtained after the simple filtration purification (Scheme 4A). Next, we attempted selective deprotection of amino acids. For deprotection of the amine group (Scheme 4B),^29^ the Fmoc group was smoothly removed by treatment of Fmoc-Orn(TCbz)-OMe (11) and Fmoc-Lys(TCbz)-OMe (23) with 1% DBU and 1% piperidine in CHCl_3_ to afford 28 and 29, respectively in quantitative yield by crystallization. We tried deprotection of carboxylic acids using several esters (Scheme 4C). The Bn group of 14 was selectively removed under hydrogenolysis conditions (1 atm),^28^ affording 30 in quantitative yield. While the TMSE group of 16 was cleaved by treatment with TBAF^30^ to provide 31, subjection of methyl ester 11 to Me_3_SnOH-mediated conditions^31^ gave the corresponding acid (32) without events. In addition, cleavage of the allyl group in 26 was effective under the conditions using catalytic amounts of Pd(PPh_3_)_4_ and morpholine,^32^ affording 33 in quantitative yield. In all cases, the desired deprotected products were obtained by the routine crystallization and filtration procedure. These results highlighted the orthogonal reactivities of the TCbz group with various conventional protecting groups in peptide chemistry.

### Introduction of the TCbz group to oligopeptides

**Scheme 5:**
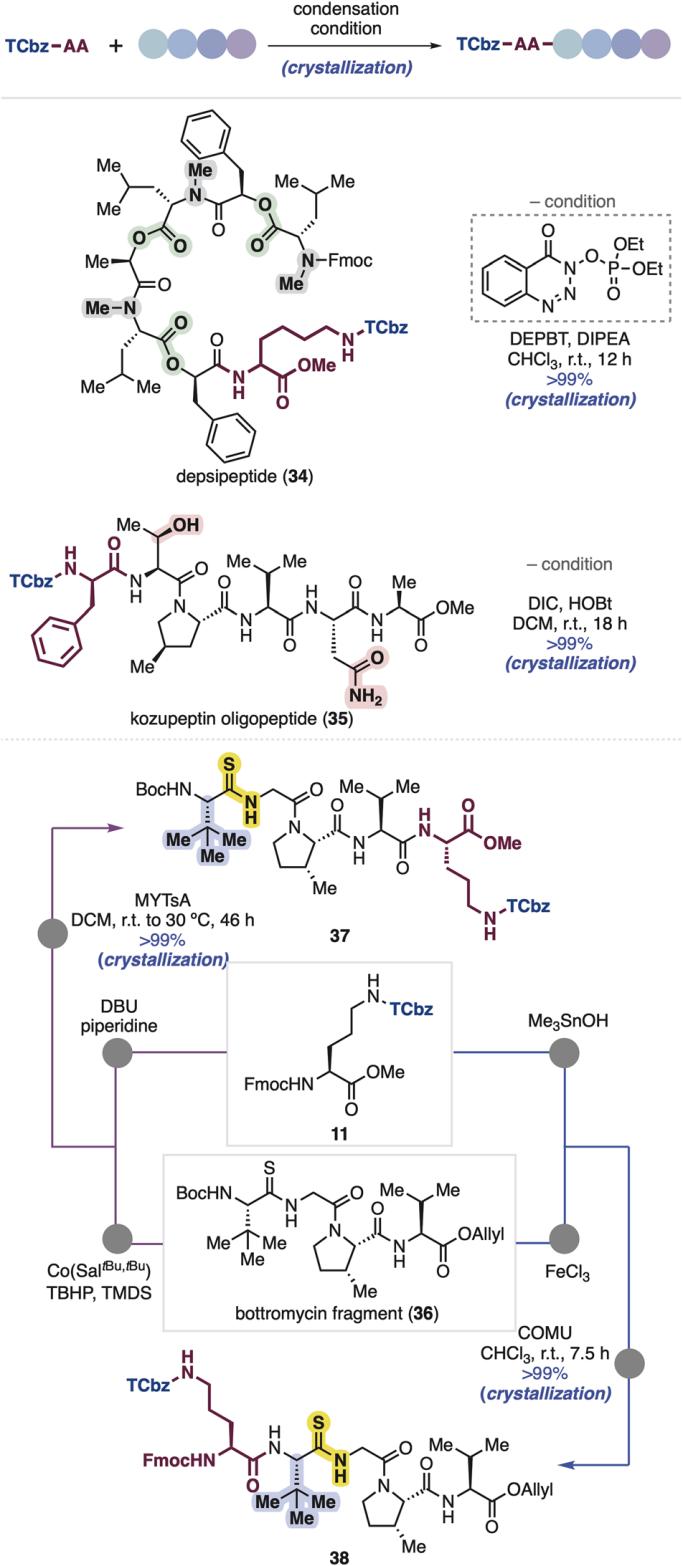
TCbz-oligopeptide synthesis.

Because oligopeptides are sometimes difficult to handle due to their polarity and solubility, we further tested robustness and properties of the TCbz group using several peptide compounds possessing a variety of side chains in our peptide library (Scheme 5). First, we attempted to couple Lys(TCbz)-OMe (29) with a depsipeptide fragment having lipophilic side chains, which was previously prepared for the synthesis of emodepside^33^ by our group.^14*i*^ Condensation at the C-terminus of the depsipeptide proceeded smoothly using DEPBT^34^ in the presence of DIPEA, providing TCbz-depsipeptide (34) in quantitative yield. Next, we used a kozupeptin fragment which possesses hydrophilic amino acids.^35^ Peptide coupling with TCbz-D-Phe-OH (33) at the N-terminus of the pentapeptide fragment using DIC and HOBt^36^ gave rise to oligopeptide 35 in quantitative yield. Remarkably, in both cases using the fragments containing lipophilic and hydrophilic functions, the TCbz-oligopeptides were easily purified by the typical crystallization and filtration procedure without loss of the desired products.

Next, we thought to demonstrate an application of both N- and C-terminal elongation strategies of Fmoc-Orn(TCbz)-OMe (11) using bottromycin tetrapeptide 36 containing unusual *tert*-leucine and thioamide moieties.^37^ In this regard, we found that treatment of 36 under metal-hydride hydrogen atom transfer (MHAT) conditions^38^ cleaved the allyl ester to give the corresponding acid, which was condensed with amine 28 derived from 11 (see Scheme 4B) using *N*-methylynetoluenesulfonamide (MYTsA),^39^ providing 37 in quantitative yield, whereas the Boc group of 36 was removed by using FeCl_3_ (ref. ^40^) to provide the corresponding amine, followed by condensation with acid 32 derived from 11 (see Scheme 4C) using COMU^41^ gave the desired product (38) quantitatively. The steric encumbrance around the amine did not affect the reaction outcome. In both cases, the products could be purified by the typical procedure, suggesting the generality of the TCbz group even in the presence of noncanonical thioamide residues.

### Application to total synthesis of calpinactam

**Scheme 6:**
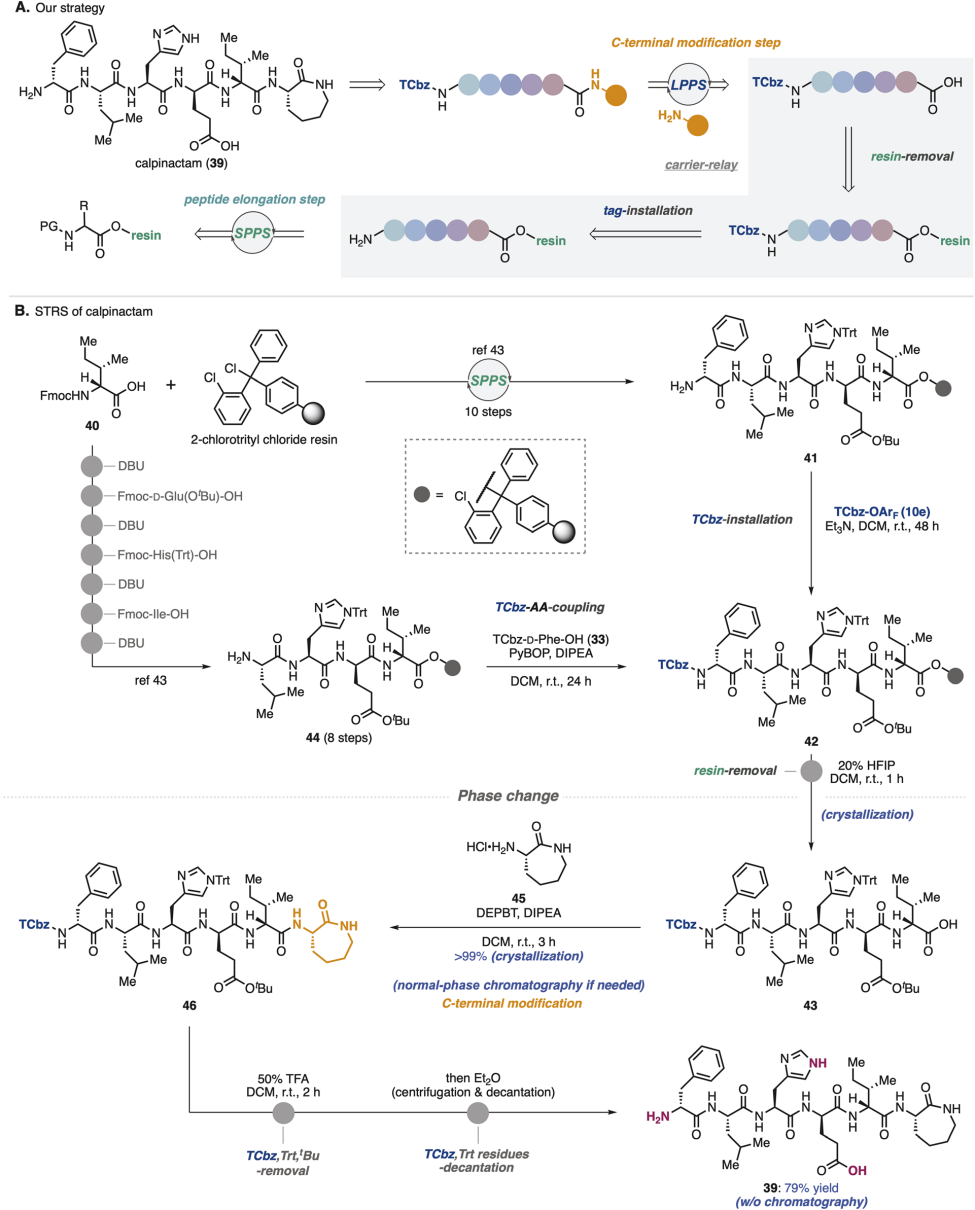
Sold/hydrophobic-tag relay synthesis (STRS) of calpinactam.

To show synthetic utilities of the nitrogen-bound hydrophobic auxiliary, we aimed to develop a new strategy for total synthesis of calpinactam (39, Scheme 6A).^42^ This unique hexapeptide natural product consists of both basic and acidic amino acids and a caprolactam at the C-terminus. Because of their characteristic structure and promising antimycobacterial properties, total syntheses in both CSPS and SPPS have been accomplished to date in our university.^42,43^ However, due to the instability of 39 under the purification conditions that were employed previously, it was capricious to obtain this natural product by fermentation as well as chemical synthesis. To overcome this issue, we proposed solid/hydrophobic-tag relay synthesis (STRS) as the third-generation route to this natural product.

In the previous SPPS route to 39, after removal of 2-chlorotrityl resin, amino caprolactam was condensed at the C-terminus in the general CSPS manner.^43^ We thought that before cleaving the solid-phase carrier, installation of the hydrophobic auxiliary (TCbz group) at the N-terminus would allow the supporting carriers to be exchanged. Selective removal of the solid-phase carrier could relay SPPS to LPPS, enabling simple purification by crystallization without the use of HPLC. Notably, in this relay strategy, even if peptide fragments contain impurities during the SPPS processes, which is a common problem, TCbz-peptides can be purified using a normal-phase system. As a result, it would allow us to avoid time-consuming operations.

To verify the STRS strategy (Scheme 6B), solid-supported pentapeptide 41 was prepared by following the previous SPPS *via* the 10-step sequence from commercially available Fmoc-D-*allo*-Ile-OH (40). Treatment of 41 with TCbz-OAr_F_ (10e) in the presence of Et_3_N successfully installed the hydrophobic auxiliary on the solid-supported peptide, affording 42. Our control experiment (see the ESI, Table S3†) suggested that the TCbz group would be intact under common resin-removal conditions using HFIP.^44^ In fact, subjection of 42 to 20% HFIP in DCM selectively cleaved the 2-chlorotrityl resin to provide TCbz-pentapeptide 43 in quantitative yield after purification by the simple filtration and subsequent crystallization. Although this result showcased the proof of concept for the relay strategy, quite an excess amount of 10e (33 equiv.) was needed for the completion of the reaction with resin-supported 41 within 48 h. To circumvent this concern, we thought to couple TCbz-D-Phe-OH (33) with the tetrapeptide-resin intermediate instead of direct introduction of the TCbz group.

In a similar manner to prepare 41, resin-supported tetrapeptide 44 was synthesized over 8 steps. Condensation of amine 44 with TCbz-acid 33 using the usual Fmoc amino acid coupling conditions, followed by cleavage of the resin afforded 43 quantitatively by the established purification. We found that DEPBT^34^ was effective for the condensation of acid 43 with amino caprolactam 45, affording TCbz-hexapeptide 46 in quantitative yield by the typical crystallization purification. This result showcased the power of the current approach to modify the C-terminus of peptides in LPPS. Finally, global cleavage of the Trt, ^*t*^Bu and TCbz groups in 46 by treatment with 50% TFA in DCM gave rise to calpinactam (39) in 79% yield. High purity of 39 could be obtained by a simple purification procedure without any chromatographic processes (centrifugation and decantation, see the ESI in detail†), highlighting the utility of the hydrophobic auxiliary.

## Conclusions

We developed a novel, stable carbonate reagent “TCbz-OAr_F_” to install a hydrophobic auxiliary on the nitrogen atom of amino acids. Using this reagent, the TCbz group was easily introduced to a variety of amino acids under mild conditions. We demonstrated that the TCbz-peptides could be purified simply by crystallization and filtration, which was also applicable to oligopeptides having a broad range of unconventional peptide residues. Furthermore, we showcased a *de novo* solid/hydrophobic-tag relay synthesis (STRS) strategy for total synthesis of the natural product, calpinactam. Introduction of the TCbz group at the N-terminus of peptide intermediates allowed a protocol that facilitates not only isolation and purification of the target peptide compounds but also C-terminal modification in LPPS. Although SPPS and LPPS have been used broadly but independently depending on needs, we hope that the TCBz group and our developed STRS strategy will serve as a bridging method for further development in this field.

## Data availability

All data associated with this publication are provided in the ESI.†

## Author contributions

T. H. and T. S. supervised the project. The design of this work was conceptualized by G. S. with input from H. N., Y. N. and T. H. H. N. carried out the experimental work. The experimental data were recorded by H. N. and the ESI† was written by G. S. and reviewed by H. N., Y. N. and T. H. The manuscript was written by H. N. and G. S. and reviewed by all authors.

## Conflicts of interest

There are no conflicts to declare.

## Supplementary Material

SC-014-D3SC01432K-s001
