# Supplementary material for: Development of a nitrogen-bound hydrophobic auxiliary: application to solid/hydrophobic-tag relay synthesis of calpinactam
Source: Chem Sci. 2023 May 1;14(25):6882–9. doi: 10.1039/d3sc01432k (PMC10306108; doi:10.1039/d3sc01432k)

Electronic Supplementary Material (ESI) for Chemical Science.
This journal is © The Royal Society of Chemistry 2023

Supplementary Information

Development of a nitrogen-bound hydrophobic auxiliary: application to solid/hydrophobic-tag relay synthesis of calpinactam

Hiroki Nakahara, Goh Sennari, Yoshihiko Noguchi
Tomoyasu Hirose,* Toshiaki Sunazuka*

Ōmura Satoshi Memorial Institute and Graduate School of Infection Control Sciences
Kitasato University, 5-9-1 Shirokane, Minato-ku, Tokyo 108-8641, Japan

Corresponding Authors

*thirose@lisci.kitasato-u.ac.jp

*sunazuka@lisci.kitasato-u.ac.jp

1. General Considerations S1

1-1. Solvents and Reagents S1

1-2. Experimental Procedures S1

1-3. Analytical Instrumentation S2

2. Reaction Investigations S3

2-1. Investigation of Hydrophobic Auxiliary Installation (Table S1 and S2) S3

2-2. Investigation for Removal of the TCbz Group (Table S3) S5

2-3. Unsuccessful Deprotection of TCbz-amino acids S6

3. Experimental Procedures and Characterization Data S7

3-1. Typical Procedure for Investigation of Leaving Groups to Install the TCbz Group S7

3-2. Gram-Scale Preparation of TCbz-OAr$_F$ (**10e**) S9

3-3. General Procedure 1 for Installation of TCbz to Amino Acids S10

3-4. Typical Procedure for Cleavage of the TCbz Group S22

3-5. Typical Procedure for Deprotection of TCbz-Amino Acids S24

3-6. Syntheses of TCbz-Supported Oligopeptides S30

3-7. Solid/Hydrophobic-Tag Relay Synthesis of Calpinactam S40

4. Spectral Data Comparison of Natural and Synthetic Calpinactam S51

5. References S55

6. NMR Spectra Charts S56

1. General Considerations

1-1. Solvents and Reagents

Unless otherwise noted, commercial reagents were purchased from Sigma Aldrich, Combi-blocks, TCI, Strem Chemicals, FUJIFILM Wako Pure Chemical Co., Watanabe Chemical Industries LTD., BLDPharm and/or Kanto Chemical Co., and used without additional purification. Solvents were purchased from Sigma Aldrich, TCI and/or Kanto Chemical Co, and used without additional purification (stored over molecular sieves). THF and DCM were sparged with argon and dried over molecular sieves prior to use. *N*-Fmoc-D-*allo*-isoleucine-trimethylsilylethyl ester (Ile-OTMSE) was prepared by following the standard literature procedure (*Bioorg. Med. Chem.*, 2000, **8**, 1677–1696.). 3,4,5-Tris(octadecyloxy)benzyl alcohol (TAG-OH) was prepared by following the literature procedure (*Tetrahedron*, 2011, **67**, 6633–6643.).

1-2. Experimental Procedures

Unless otherwise noted in the experimental procedures, reactions were carried out in flame- or oven-dried glassware under a positive pressure of N_2 in anhydrous solvents using standard Schlenk techniques. Reaction temperatures above room temperature (20–25 °C) were controlled by an IKA® temperature modulator or AS ONE Co. Oil Bath and monitored using liquid-in-glass thermometers. Reaction progress was monitored by thin-layer chromatography (TLC) on Sigma Aldrich/Millipore silica gel TLC plates (60 Å, F254 indicator). TLC plates were visualized by exposure to ultraviolet light (254 nm), and/or stained by submersion in aqueous potassium permanganate solution ($KMnO_4$), *p*-anisaldehyde, ceric ammonium molybdate, ninhydrin, or phosphomolybdic acid stains and heating with a heat gun or heating plate. Organic solutions were concentrated under reduced pressure on an EYELA temperature-controlled rotary evaporator equipped with a cooling condenser. Flash column chromatography was performed with glass columns using Kanto Chemical silica gel (60 N, spherical neutral, 40–50 µm particle size), using ACS grade solvents. All yields refer to spectroscopically (^{1}H and ^{13}C NMR) pure material.

1-3. Analytical Instrumentation

^{1}H NMR and ^{13}C NMR data were recorded on a JEOL JNM-ECA-500 (500 MHz for ^{1}H NMR and 126 MHz for ^{13}C NMR) spectrometer using $CDCl_3$, CD_3OD, or $(CD_3)_2SO$ as a solvent, typically at 20–23 °C. Chemical shifts (δ) are reported in ppm relative to the residual solvent signal (δ 7.26 for ^{1}H NMR & δ 77.2 for ^{13}C NMR in $CDCl_3$, δ 3.31 for ^{1}H NMR & δ 49.0 for ^{13}C NMR in CD_3OD, and δ 2.49 for ^{1}H NMR & δ 39.5 for ^{13}C NMR in $(CD_3)_2SO$). Data for ^{1}H and ^{13}C spectroscopy are reported as follows: chemical shift (δ ppm), multiplicity (s = singlet, d = doublet, t = triplet, q = quartet, p = pentet, m = multiplet, br = broad, app = apparent), coupling constant (Hz), integration. High-resolution mass spectra (HRMS) were measured on a JEOL JMS-AX505HA, JEOL JMS-700 MStation and/or JEOL JMS-T100LP. Data acquisition and processing were performed using the Xcalibur™ software. Optical rotations were measured on a JASCO P-2020 polarimeter. Infrared spectra were measured on a Horiba FT-210 spectrometer. Melting points were measured on a YANACO MP-500P or OptiMelt (Standard Research Systems) apparatus.

2. Reaction Investigations

2-1. Investigation of Hydrophobic Auxiliary Installation (Table S1 and S2)

• *N*-Oxysuccinimide (Table S1)

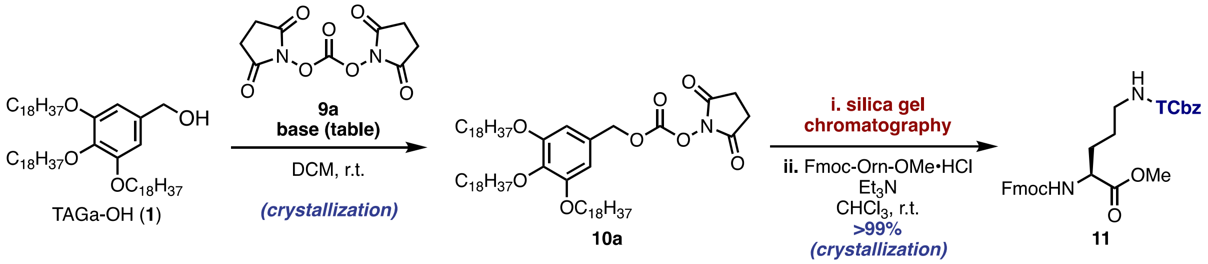

entry	base	result
1	none	**no reaction**
2	pyridine	**mixture w/ unknown products**
3	DIPEA	**mixture w/ unknown products**
4	Et_3N	**mixture w/ unknown products**

We initially thought to use *N,N'*-disuccinimidyl carbonate (**9a**) for a tethering group to install the hydrophobic auxiliary. In this regard, TAGa-OH (**1**) was treated with **9a** under several conditions (entries 1–4). In the absence of base, no reaction was observed (entry 1), whereas we saw the desired product in the presence of the bases, shown in entries 2–4. After routine crystallization for TAG-based chemistry, we detected unknown side products in these cases. As such, it was needed to purify by silica gel column chromatography. Although we confirmed that the isolated **9a** showed the desired reactivity with the amino acid to give rise to Fmoc-Orn(TCbz)-OMe (**11**) in quantitative yield by crystallization, it was not ideal to run column chromatography, especially for large-scale synthesis, and thus we continued to seek for alternative tethering groups.

• Imidazole (Table S2)

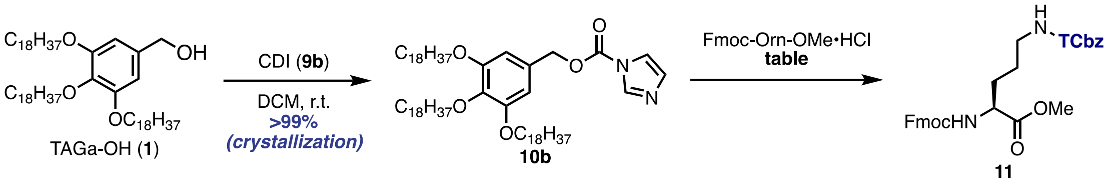

entry	condition	result
1	Et_3N, $CHCl_3$, r.t.	**no reaction**
2	DMAP, $CHCl_3$, r.t.	**no reaction**
3	DMAP, $CHCl_3$, reflux	**decomposition**
4	MeI, $CHCl_3$, r.t.	**complex mixture**
5	MeOTf, DIPEA, $CHCl_3$, r.t.	**decomposition**

In the contrast to table S1, treatment of **1** with CDI (**9b**) in the absence of bases smoothly gave the desired product (**10b**) in a pure form without events. However, in this case, the imidazole group was, in turn, not reactive enough for the following peptide coupling reaction (entries 1–5). We attempted several conditions including activation of the imidazole group (entries 4 and 5).[1] However, in all cases that were tried, we did not observe any productive reactions.

At the end, we found that the pentafluorophenoxy group is the optimal leaving group in terms of both preparation of the precursor and installation of the amino acid (*vide infra*).

(1) J. A. Grzyb and R. A. Batey, *Tetrahedron Lett.*, 2003, **44**, 7485–7488.

2-2. Investigation for Removal of the TCbz Group (Table S3)

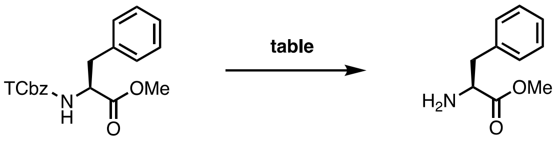

entry	condition	time	12	27
1	50% AcOH in DCM, r.t.	24 h	>99%	-
2	50% TFA in DCM, r.t.	24 h	-	>99%
3	10% TFA in DCM, r.t.	24 h	-	**>99%**
4	1% TFA in DCM, r.t.	24 h	89%	9%
5	20% HFIP in DCM, r.t.	72 h	>99%	-
6	DDQ, DCM/H_2O (20:1 v/v), r.t.	24 h	88%	0%
7	CAN, DCM/H_2O (9:1 v/v), r.t.	22 h	0%	0%
8	H_2 (1 atm), Pd/C, THF	24 h	>99%	-
9	H_2 (1 atm), $Pd(OH)_2$/C, THF	24 h	>99%	-
10	H_2 (5 MPa), Pd/C, THF	60 h	-	**88%**

isolated yields shown.

We began investigation with weakly acidic conditions, 50% AcOH in DCM resulted in no reaction (entry 1). Treatment with 50% TFA in DCM successfully cleaved the TCbz group in quantitative yield (entry 2), which later in turn 10% TFA in DCM was strong enough for the removal (entry 3). However, 1 % TFA in DCM was not effectively cleaving within 24 h (entry 4), suggesting that selective cleavage of other protecting groups under the condition might be possible.

Importantly, treatment of **12** with 20% HFIP in DCM, which we often employ for removal of solid-phase resins, resulted in no reaction. This result was encouraging us to pursue resin-TCbz relay synthesis strategy.

Oxidative conditions (entries 6 and 7)[2,3] was not successful, resulting in (partial) decomposition, whereas reductive hydrogenolysis conditions gave no reaction (entries 8 and 9). These results were encouraging us to investigate selective cleavage of Bn groups under these conditions.

We found that hydrogenolysis under pressurized conditions (5 MPa) successfully cleaved the TCBz group in 88% yield (entry 10).

(2) J. Hassfeld, U. Eggert and M. Kalesse, *Synthesis*, 2005, **7**, 1183–1199.

(3) J. J. Fleming and J. Du Bois, *J. Am. Chem. Soc.*, 2006, **128**, 3926–3927.

2-3. Unsuccessful Deprotection of TCbz-amino acids

- Cleavage of the Trt group

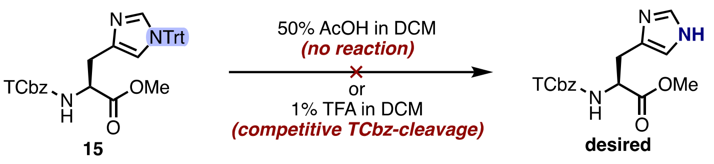

- Cleavage of the Boc group[4–6]

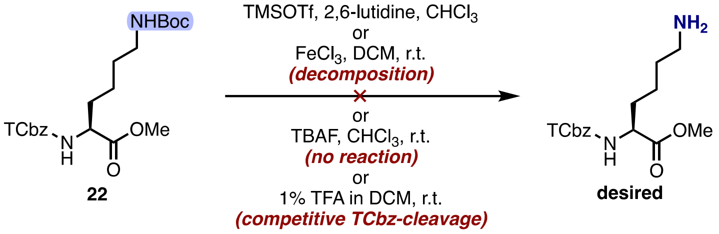

- Cleavage of the Et group

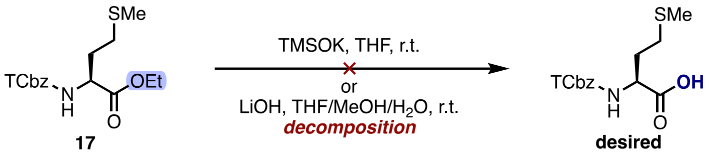

(4) P. Singh and G. Panda, *RSC Adv.*, 2014, **4**, 2161–2166.

(5) R. S. Giri, S. Roy, G. Dolai, S. R. Manne and B. Mandal, *ChemistrySelect*, 2020, **5**, 2050–2056.

(6) U. Jacquemard, V. Bénéteau, M. Lefoix, S. Routier, J.-Y. Mérour and G. Coudert, *Tetrahedron*, 2004, **60**, 10039–10047.

3. Experimental Procedures and Characterization Data

3-1. Typical Procedure for Investigation of Leaving Groups to Install the TCbz Group

- Activation

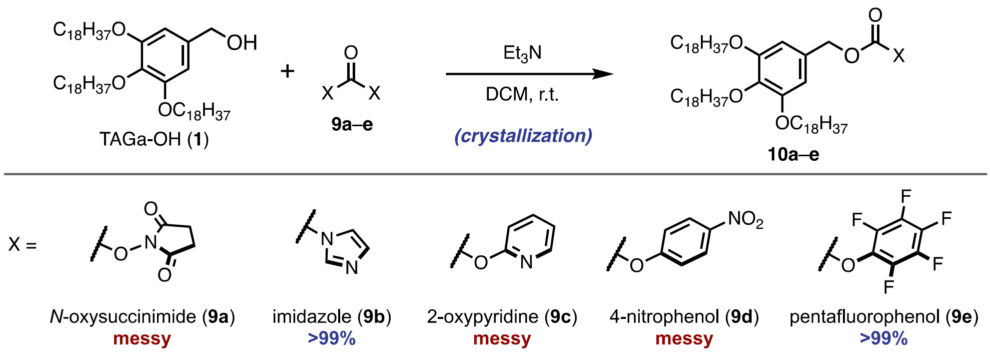

To a solution of TAGa-OH (**1**) (100.0 mg, 0.109 mmol) in DCM (2.19 mL, 0.05 M) was added the corresponding carbonate or CDI (0.130 mmol, 1.2 equiv) and Et_3N (22.7 µL, 0.164 mmol, 1.5 equiv) at room temperature. After stirring at room temperature until the consumption of **1** (monitored by TLC), the reaction mixture was poured into cold MeOH (11.0 mL, 0.01 M). The resulting suspension was stirred for 30 min at 0 ºC and filtered through a pad of Celite (washed with excess MeOH). The product collected on the filter cake was dissolved in $CHCl_3$ and then eluted by suction filtration using excess $CHCl_3$. The resulting filtrate was concentrated *in vacuo* and dried under high vacuum. The reaction outcome was analyzed by ^{1}H NMR (table), yielding the corresponding TCbz-X **10b** (111.2 mg, 0.110 mmol, >99%) as a white powder and **10e** (112.5 mg, 0.109 mmol, >99%) as a white powder.

TCbz-imidazole (**10b**)

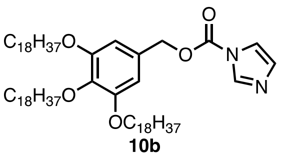

10b

Rf-value: 0.32 (hexane/EtOAc = 4:1, stained with phosphomolybdic acid)

HRMS (*m/z*): FAB $[M]^+$ calculated for $C_{65}H_{118}N_2O_5$: 1006.9041, found: 1006.9037.

^{1}H NMR (500 MHz, $CDCl_3$): δ 8.15 (t, *J* = 1.1 Hz, 1H), 7.43 (t, *J* = 1.5 Hz, 1H), 7.06 (dd, *J* = 1.5, 1.1 Hz, 1H), 6.61 (s, 2H), 5.30 (s, 2H), 3.99 – 3.93 (m, 6H), 1.84 – 1.76 (m, 4H), 1.73 (app q, *J* = 7.0 Hz, 2H), 1.50 – 1.43 (m, 6H), 1.38 – 1.17 (m, 84H), 0.88 (t, *J* = 7.0 Hz, 9H).

^{13}C NMR (126 MHz, $CDCl_3$): δ 153.5 (2C), 148.8, 139.0, 137.3, 130.8, 128.9, 117.3, 107.6 (2C), 73.6, 70.5, 69.3 (2C), 32.0 (3C), 30.4, 30.0 – 29.7 (overlap, 29C), 29.6 – 29.4 (overlap, 8C), 26.24, 26.21 (3C), 22.8 (3C), 14.2 (3C).

- Installation

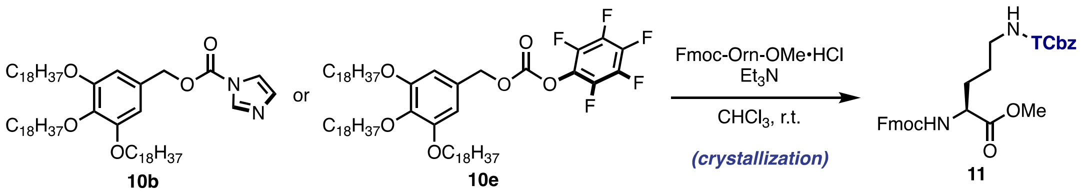

To a solution of **10b** (100.8 mg, 0.100 mmol) or **10e** (112.4 mg, 0.100 mmol) in $CHCl_3$ (2.00 mL, 0.05 M) was added Fmoc-Orn-OMe•HCl (56.7 mg, 0.140 mmol, 1.4 equiv), Et_3N (22.2 µL, 0.160 mmol, 1.6 equiv) at room temperature under air. After stirring at room temperature until the consumption of **10b** or **10e** (monitored by TLC), the reaction mixture was poured into cold MeOH (10.0 mL, 0.01 M). The resulting suspension was stirred for 30 min at 0 ºC and filtered through a pad of Celite (washed with excess MeOH). The product collected on the filter cake was dissolved in $CHCl_3$ and then eluted by suction filtration using excess $CHCl_3$. The resulting filtrate was concentrated *in vacuo* and dried under high vacuum. The reaction outcome was analyzed by ^{1}H NMR.

Characterization data of **11 are provided on page S16–17 (vide infra).*

3-2. Gram-Scale Preparation of TCbz-OAr$_F$ (10e)

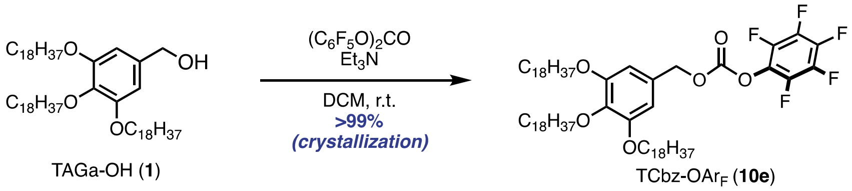

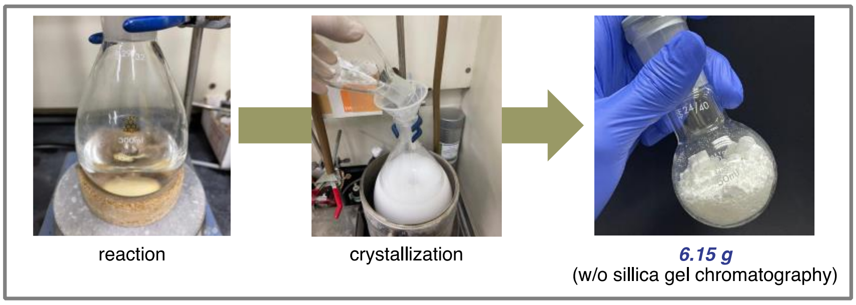

To a solution of TAGa-OH (**1**) (5.00 g, 5.47 mmol) in DCM (109 ml, 0.05 M) was added bis(pentafluoro phenyl)carbonate (2.59 g, 6.57 mmol, 1.2 equiv), Et_3N (1.15 ml, 8.21 mmol, 1.5 equiv) at room temperature under air. After stirring at room temperature for 24 h, the reaction mixture was poured into cold MeOH (545 mL, 0.01 M). The resulting suspension was stirred for 30 min at 0 ºC and filtered through a pad of Celite (washed with excess MeOH). The product collected on the filter cake was dissolved in $CHCl_3$ and then eluted by suction filtration using excess $CHCl_3$. The resulting filtrate was concentrated *in vacuo* and dried under high vacuum, yielding TCbz-OAr$_F$ (**10e**) (6.15 g, 5.47 mmol, >99%).

Rf-value: 0.35 (hexane/EtOAc = 50:1, stained with phosphomolybdic acid)

HRMS (*m/z*): FAB $[M]^+$ calculated for $C_{68}H_{115}F_5O_6$: 1122.8614, found: 1122.8619.

^{1}H NMR (500 MHz, $CDCl_3$): δ 6.59 (s, 2H), 5.22 (s, 2H), 3.99 – 3.94 (m, 6H), 1.80 (tt, *J* = 14.3, 6.7 Hz, 4H), 1.74 (tt, *J* = 14.4, 6.8 Hz, 2H), 1.50 – 1.43 (m, 6H), 1.38 – 1.22 (m, 84H), 0.88 (t, *J* = 6.9 Hz, 9H).

^{13}C NMR (126 MHz, $CDCl_3$): δ 153.5 (2C), 151.4, 142.3 (m), 138.9, 139.7 (m, 2C), 138.0 (m, 2C) 128.7, 125.8 (m), 107.2 (2C), 73.6, 72.7, 69.3 (2C), 50.8, 32.0 (3C), 30.4, 30.0 – 29.7 (overlapped, 29C), 29.6 – 29.4 (overlapped, 8C), 26.2 (3C), 22.8 (3C), 14.2 (3C).

3-3. General Procedure 1 for Installation of TCbz to Amino Acids

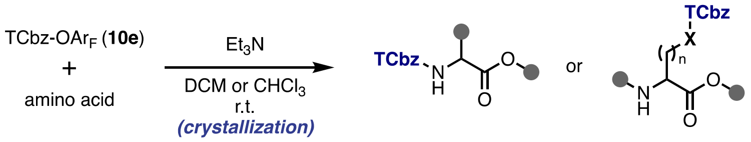

To a solution of TCbz-OAr$_F$ (**10e**) (112.4 mg, 0.100 mmol) in DCM or $CHCl_3$ (2.00 mL, 0.05 M) was added an amino acid (0.140 mmol, 1.4 equiv), Et_3N (22.2 µL, 0.160 mmol, 1.6 equiv) at room temperature under air. After stirring at room temperature until the consumption of **10e** (monitored by TLC, typically 24 – 48 h), the reaction mixture was poured into cold MeOH (10.0 mL, 0.01 M). The resulting suspension was stirred for 30 min at 0 °C and filtered through a pad of Celite (washed with excess MeOH). The product collected on the filter cake was dissolved in $CHCl_3$ and then eluted by suction filtration using excess $CHCl_3$. The resulting filtrate was concentrated *in vacuo* and dried under high vacuum, yielding the corresponding TCbz-AA (**11–26**) as a powder.

TCbz-Phe-OMe (**12**)

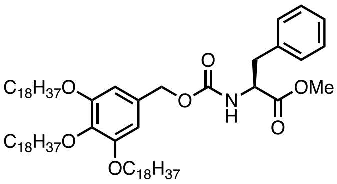

12

Prepared according to General Procedure 1 using Phe-OMe•HCl as the amino acid, **12** (111.9 mg, 0.100 mmol, >99%) was obtained as a white powder.

Rf-value: 0.41 (hexane/EtOAc = 4:1, stained with phosphomolybdic acid)

HRMS (*m/z*): FAB $[M]^+$ calculated for $C_{72}H_{127}NO_7$: 1117.9613, found: 1117.9613.

$[\alpha]_D^{22}$ = +43.0 (c = 0.1, $CHCl_3$)

^{1}H NMR (500 MHz, $CDCl_3$): δ 7.30 – 7.20 (m, 3H), 7.11 – 7.07 (m, 2H), 6.52 (s, 2H), 5.20 (br-d, *J* = 8.3 Hz, 1H), 4.98 (s, 2H), 4.69 – 4.64 (br-m, 1H), 3.97 – 3.91 (m, 6H), 3.72 (s, 3H), 3.11 (app qd, *J* = 13.9, 5.5 Hz, 2H), 1.79 (app p, *J* = 6.8 Hz, 4H), 1.72 (app q, *J* = 7.1 Hz, 2H), 1.50 – 1.42 (m, 6H), 1.37 – 1.23 (m, 84H), 0.88 (t, *J* = 6.9 Hz, 9H).

^{13}C NMR (126 MHz, $CDCl_3$): δ 172.1, 155.8, 153.3 (2C), 138.2, 135.8, 131.2, 129.4 (2C), 128.7 (2C), 127.3, 106.9 (2C), 73.5 (2C), 69.2 (2C), 67.5, 54.9, 52.4, 38.3, 32.1 (3C), 30.5, 30.0 – 29.7 (overlap, 29C), 29.6 – 29.4 (overlap, 8C), 26.3, 26.2 (2C), 22.8 (3C), 14.2 (3C).

TCbz-Tyr-OMe (**13**)

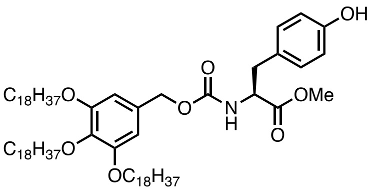

13

Prepared according to General Procedure 1 using Tyr-OMe•HCl as the amino acid, **13** (113.5 mg, 0.100 mmol, >99%) was obtained as a pale-yellow powder.

Rf-value: 0.40 (hexane/EtOAc = 4:1, stained with phosphomolybdic acid)

HRMS (*m/z*): FAB $[M]^+$ calculated for $C_{72}H_{127}NO_8$: 1133.9562, found: 1133.9543.

$[\alpha]_D^{23}$ = +21.6 (c = 0.1, $CHCl_3$)

^{1}H NMR (500 MHz, $CDCl_3$): δ 6.94 (d, *J* = 8.4 Hz, 2H), 6.73 – 6.69 (m, 2H), 6.52 (s, 2H), 5.18 (d, *J* = 8.4 Hz, 1H), 4.99 (d, *J* = 6.9 Hz, 1H), 4.95 (d, *J* = 6.9 Hz, 1H), 4.76 (br-s, 1H), 4.60 (app dt, *J* = 8.4, 6.2 Hz, 1H), 3.97 – 3.91 (m, 6H), 3.72 (s, 3H), 3.06 (dd, *J* = 14.1, 6.2 Hz, 1H), 2.99 (dd, *J* = 14.1, 6.2 Hz, 1H), 1.79 (app p, *J* = 6.6 Hz, 4H), 1.73 (app q, *J* = 7.0 Hz, 2H), 1.50 – 1.41 (m, 6H), 1.38 – 1.20 (m, 84H), 0.88 (t, *J* = 7.0 Hz, 9H).

^{13}C NMR (126 MHz, $CDCl_3$): δ 172.4, 155.9, 155.3, 153.3 (2C), 138.1, 131.2, 130.4 (2C), 127.3, 115.7 (2C), 107.0 (2C), 73.6 (2C), 69.3 (2C), 67.6, 55.1, 52.5, 37.6, 32.1 (3C), 30.4, 30.0 – 29.7 (overlap, 29C), 29.6 – 29.4 (overlap, 8C), 26.3 (3C), 22.8 (3C), 14.2 (3C).

TCbz-Trp-OBn (**14**)

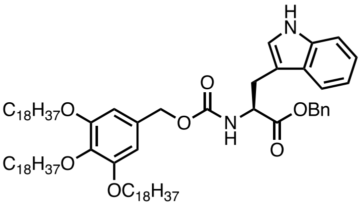

14

Prepared according to General Procedure 1 using Typ-OBn•HCl as the amino acid, **14** (123.4 mg, 0.100 mmol, >99%) was obtained as a pale-yellow powder.

Rf-value: 0.40 (hexane/EtOAc = 4:1, stained with phosphomolybdic acid)

HRMS (*m/z*): FAB $[M]^+$ calculated for $C_{80}H_{132}N_2O_7$: 1233.0035, found: 1233.0033.

$[\alpha]_D^{23}$ = +5.94 (c = 0.1, $CHCl_3$)

^{1}H NMR (500 MHz, $CDCl_3$): δ 7.93 (s, 1H), 7.50 (d, *J* = 8.0 Hz, 1H), 7.36 – 7.31 (m, 4H), 7.25 – 7.21 (m, 2H), 7.17 (t, *J* = 7.6 Hz, 1H), 7.06 (t, *J* = 7.6 Hz, 1H), 6.75 (d, *J* = 2.4 Hz, 1H), 6.52 (s, 2H), 5.31 (d, *J* = 8.0 Hz, 1H), 5.09 (s, 2H), 5.01 (d, *J* = 12.0 Hz, 1H), 4.96 (d, *J* = 12.0 Hz, 1H), 4.79 – 4.74 (m, 1H), 3.93 (app q, *J* = 6.7 Hz, 6H), 3.31 (d, *J* = 5.5 Hz, 2H), 1.79 (app q, *J* = 7.1 Hz, 4H), 1.72 (app q, *J* = 7.0 Hz, 2H), 1.51 – 1.41 (m, 6H), 1.38 – 1.20 (m, 84H), 0.88 (t, *J* = 6.8 Hz, 9H).

^{13}C NMR (126 MHz, $CDCl_3$): δ 171.9, 155.9, 153.3 (2C), 138.2, 136.2, 135.4, 131.3, 128.7 (2C), 128.6 (2C), 128.5 (2C), 127.7, 123.1, 122.3, 119.8, 118.7, 111.3, 109.8, 107.0 (2C), 73.6 (2C), 69.2 (2C), 67.5, 67.3, 54.8, 32.1 (3C), 30.5, 30.0 – 29.7 (overlap, 29C), 29.6 – 29.4 (overlap, 8C), 28.1, 26.3 (3C), 22.8 (3C), 14.2 (3C).

TCbz-His(Trt)-OMe (**15**)

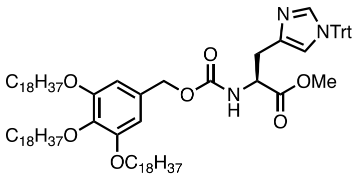

15

Prepared according to General Procedure 1 using His(Trt)-OMe•HCl as the amino acid, **15** (135.1 mg, 0.100 mmol, >99%) was obtained as a pale-yellow powder.

Rf-value: 0.42 (hexane/EtOAc = 4:1, stained with phosphomolybdic acid)

HRMS (*m/z*): FAB $[M+Na]^+$ calculated for $C_{88}H_{139}N_3O_7Na$: 1373.0511, found: 1373.0514.

$[\alpha]_D^{23}$ = +8.08 (c = 0.1, $CHCl_3$)

^{1}H NMR (500 MHz, $CDCl_3$): δ 7.36 – 7.29 (m, 10H), 7.11 – 7.07 (m, 6H), 6.54 (s, 2H), 6.52 (s, 1H), 6.36 (d, *J* = 8.2 Hz, 1H), 4.98 (s, 2H), 4.60 (dt, *J* = 8.2, 4.8 Hz, 1H), 3.97 – 3.90 (m, 6H), 3.60 (s, 3H), 3.10 – 3.03 (m, 1H), 3.04 – 2.95 (m, 1H), 1.78 (app p, *J* = 6.6 Hz, 4H), 1.72 (app q, *J* = 7.0 Hz, 2H), 1.50 – 1.41 (m, 6H), 1.38 – 1.22 (m, 84H), 0.88 (t, *J* = 6.9 Hz, 9H).

^{13}C NMR (126 MHz, $CDCl_3$): δ 172.2, 156.3, 153.2 (2C), 142.4 (3C), 138.9, 138.1, 136.4, 131.5, 129.9 (6C), 128.2 (9C), 119.7, 106.9 (2C), 77.4, 75.4, 73.5, 69.2 (2C), 67.4, 54.3, 52.2, 32.1 (3C), 30.5, 30.2, 30.0 – 29.7 (overlap, 29C), 29.6 – 29.4 (overlap, 8C), 26.2 (3C), 22.8 (3C), 14.2 (3C).

TCbz-Ile-OTMSE (**16**)

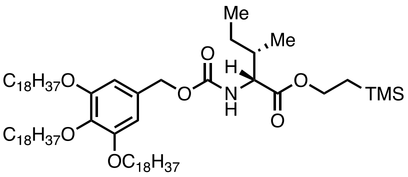

16

Prepared according to General Procedure 1 using Ile-OTMSE as the amino acid, **16** (118.7 mg, 0.101 mmol, >99%) was obtained as a white powder.

Rf-value: 0.49 (hexane/EtOAc = 4:1, stained with phosphomolybdic acid)

HRMS (*m/z*): FAB $[M]^+$ calculated for $C_{73}H_{139}NO_7Si$: 1170.0321, found: 1170.0334.

$[\alpha]_D^{22}$ = +7.16 (c = 0.1, $CHCl_3$)

^{1}H NMR (500 MHz, $CDCl_3$): δ 6.54 (s, 2H), 5.29 (d, *J* = 9.0 Hz, 1H), 4.99 (app q, *J* = 11.9 Hz, 2H), 4.31 (dd, *J* = 9.0, 4.7 Hz, 1H), 4.26 – 4.17 (m, 2H), 3.96 (t, *J* = 6.5 Hz, 4H), 3.93 (t, *J* = 6.6 Hz, 2H), 1.95 – 1.84 (m, 1H), 1.83 – 1.75 (m, 4H), 1.75 – 1.69 (m, 2H), 1.51 – 1.39 (m, 6H), 1.38 – 1.23 (m, 84H), 1.21 – 1.12 (m, 2H), 1.04 – 0.99 (m, 2H), 0.95 – 0.89 (m, 6H), 0.88 (t, *J* = 7.0 Hz, 9H), 0.05 (s, 9H).

^{13}C NMR (126 MHz, $CDCl_3$): δ 172.3, 156.2, 153.3 (2C), 138.2, 131.3, 106.8 (2C), 73.5 (2C), 69.2 (2C), 67.5, 63.7, 58.5, 38.3, 32.1 (3C), 30.5, 30.0 – 29.7 (overlap, 29C), 29.6 – 29.4 (overlap, 8C), 26.2 (3C), 25.1, 22.8 (3C), 17.6, 15.6, 14.2 (3C), 11.8, -1.4 (3C).

TCbz-Met-OEt (**17**)

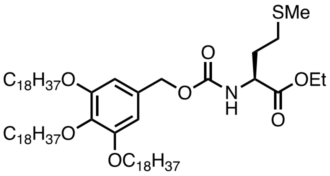

17

Prepared according to General Procedure 1 using Met-OEt•HCl as the amino acid, **17** (111.7 mg, 0.100 mmol, >99%) was obtained as a pale-yellow powder.

Rf-value: 0.43 (hexane/EtOAc = 4:1, stained with phosphomolybdic acid)

HRMS (*m/z*): FAB $[M]^+$ calculated for $C_{69}H_{129}NO_7S$: 1115.9490, found: 1115.9486.

$[\alpha]_D^{22}$ = +6.90 (c = 0.1, $CHCl_3$)

^{1}H NMR (500 MHz, $CDCl_3$): δ 6.54 (s, 2H), 5.39 (d, *J* = 8.3 Hz, 1H), 5.00 (s, 2H), 4.51 – 4.46 (m, 1H), 4.21 (q, *J* = 7.2 Hz, 2H), 3.96 (t, *J* = 6.5 Hz, 4H), 3.93 (t, *J* = 6.6 Hz, 2H), 2.56 – 2.50 (m, 2H), 2.21 – 2.12 (m, 1H), 2.09 (s, 3H), 2.02 – 1.93 (m, 1H), 1.79 (app p, *J* = 6.7 Hz, 4H), 1.76 – 1.69 (m, 2H), 1.50 – 1.42 (m, 6H), 1.38 – 1.19 (m, 87H), 0.91 – 0.85 (m, 9H).

^{13}C NMR (126 MHz, $CDCl_3$): δ 172.1, 156.0, 153.3 (2C), 138.3, 131.1, 106.9 (2C), 73.5 (2C), 69.2 (2C), 67.6, 61.8, 53.3, 32.2, 32.1 (3C), 30.5, 30.0, 30.0 – 29.7 (overlap, 29C), 29.6 – 29.4 (overlap, 8C), 26.2 (3C), 22.8 (3C), 15.6, 14.3, 14.2 (3C).

TCbz-*N*-Me-Gly-OMe (**18**)

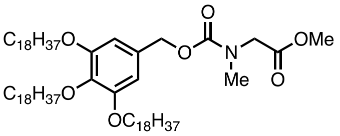

18

Prepared according to General Procedure 1 using *N*-Me-Gly-OMe•HCl as the amino acid, **18** (104.3 mg, >99%) was obtained as a pale-yellow powder.

Rf-value: 0.44 (hexane/EtOAc = 4:1, stained with phosphomolybdic acid)

HRMS (*m/z*): FAB $[M]^+$ calculated for $C_{66}H_{123}NO_7$: 1041.9300, found: 1041.9293.

^{1}H NMR (500 MHz, $CDCl_3$): δ 6.55 (s, 1.2H), 6.49 (*rotamer, s, 0.8H), 5.04 (s, 1.2H), 5.02 (*rotamer, s, 0.8H), 4.06 (s, 1.2H), 4.00 (*rotamer, s, 0.8H), 3.99 – 3.90 (m, 6H), 3.75 (s, 1.8H), 3.68 (*rotamer, s, 1.2H), 3.01 (*rotamer, m, 1.2H), 3.00 (m, 1.8H), 1.83 – 1.69 (m, 6H), 1.50 – 1.41 (m, 6H), 1.38 – 1.19 (m, 84H), 0.88 (t, *J* = 6.9 Hz, 9H).

^{13}C NMR (126 MHz, $CDCl_3$): δ 170.1, 156.9 & 156.2* (*rotamer), 153.3 (2C), 138.1 & 138.0* (*rotamer), 131.65* & 131.58 (*rotamer), 106.7 & 106.5* (*rotamer, 2C), 73.6 (2C), 69.2 (2C), 68.0 & 67.8* (*rotamer), 52.22 & 52.19* (*rotamer), 50.8 & 50.6* (*rotamer), 36.2* & 35.5 (*rotamer), 32.1 (3C), 30.5, 30.0 – 29.7 (overlap, 29C), 29.6 – 29.4 (overlap, 8C), 26.3 (3C), 22.8 (3C), 14.3 (3C).

TCbz-Ser-OMe (**19**)

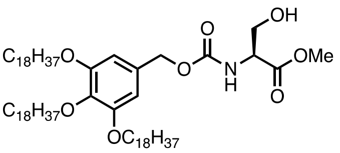

19

Prepared according to General Procedure 1 using Ser-OMe•HCl as the amino acid, **19** (105.9 mg, 0.100 mmol, >99%) was obtained as a pale-yellow powder.

Rf-value: 0.40 (hexane/EtOAc = 4:1, stained with phosphomolybdic acid)

HRMS (*m/z*): FAB $[M]^+$ calculated for $C_{66}H_{123}NO_8$: 1057.9249, found: 1057.9244.

$[\alpha]_D^{23}$ = +13.9 (c = 0.1, $CHCl_3$)

^{1}H NMR (500 MHz, $CDCl_3$): δ 6.55 (s, 2H), 5.68 (br-d, *J* = 7.6 Hz, 1H), 5.01 (s, 2H), 4.46 (br-s, 1H), 4.05 – 3.90 (m, 8H), 3.79 (s, 3H), 2.09 (br-s, 1H), 1.79 (app p, *J* = 7.0 Hz, 4H), 1.72 (app q, *J* = 7.1 Hz, 2H), 1.50 – 1.42 (m, 6H), 1.37 – 1.20 (m, 84H), 0.87 (app td, *J* = 6.9, 1.6 Hz, 9H).

^{13}C NMR (126 MHz, $CDCl_3$): δ 171.1, 156.3, 153.3 (2C), 138.3, 131.0, 106.9 (2C), 73.6 (2C), 69.2 (2C), 67.8, 63.4, 56.2, 52.9, 32.1 (3C), 30.5, 30.0 – 29.7 (overlap, 29C), 29.6 – 29.4 (overlap, 8C), 26.3 (3C), 22.8 (3C), 14.2 (3C).

TCbz-Glu(O^{t}Bu)-OMe (**20**)

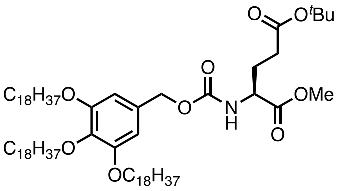

20

Prepared according to General Procedure 1 using Glu(O^{t}Bu)-OMe•HCl as the amino acid, **20** (115.7 mg, 0.100 mmol, >99%) was obtained as a pale-yellow powder.

Rf-value: 0.49 (hexane/EtOAc = 4:1, stained with phosphomolybdic acid)

HRMS (*m/z*): FAB $[M+Na]^+$ calculated for $C_{72}H_{133}NO_9Na$: 1178.9878, found: 1178.9884.

$[\alpha]_D^{23}$ = +5.76 (c = 0.1, $CHCl_3$)

^{1}H NMR (500 MHz, $CDCl_3$): δ 6.53 (s, 2H), 5.38 (d, *J* = 8.2 Hz, 1H), 4.99 (s, 2H), 4.43 – 4.37 (m, 1H), 3.96 (t, *J* = 6.5 Hz, 4H), 3.93 (t, *J* = 6.5 Hz, 2H), 3.75 (s, 3H), 2.39 – 2.25 (m, 2H), 2.15 (dt, *J* = 13.4, 6.9 Hz, 1H), 1.95 (dt, *J* = 14.3, 6.9 Hz, 1H), 1.79 (app p, *J* = 6.6 Hz, 4H), 1.72 (app q, *J* = 7.0 Hz, 2H), 1.50 – 1.44 (m, 6H), 1.43 (s, 9H), 1.38 – 1.19 (m, 84H), 0.88 (t, *J* = 6.9 Hz, 9H).

^{13}C NMR (126 MHz, $CDCl_3$): δ 172.6, 172.1, 156.0, 153.3 (2C), 138.2, 131.1, 106.9 (2C), 80.9, 73.5 (2C), 69.2 (2C), 67.6, 53.5, 52.6, 32.1 (3C), 31.5, 30.5, 30.0 – 29.7 (overlap, 29C), 29.6 – 29.4 (overlap, 8C), 28.1 (3C), 27.7, 26.2 (3C), 22.8 (3C), 14.2 (3C).

TCbz-Orn(Cbz)-OMe (**21**)

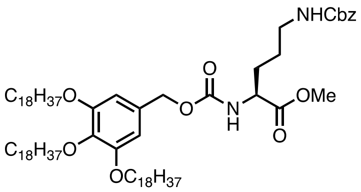

21

Prepared according to General Procedure 1 using Orn(Cbz)-OMe•HCl as the amino acid, **21** (122.0 mg, 0.100 mmol, >99%) was obtained as a pale-yellow powder.

Rf-value: 0.39 (hexane/EtOAc = 4:1, stained with phosphomolybdic acid)

HRMS (*m/z*): FAB $[M+Na]^+$ calculated for $C_{76}H_{134}N_2O_9Na$: 1241.9987, found: 1241.9991.

$[\alpha]_D^{23}$ = +4.16 (c = 0.1, $CHCl_3$)

^{1}H NMR (500 MHz, $CDCl_3$): δ 7.38 – 7.29 (m, 5H), 6.54 (s, 2H), 5.33 (d, *J* = 8.1 Hz, 1H), 5.09 (s, 2H), 4.98 (app q, *J* = 12.0 Hz, 2H), 4.80 (br-s, 1H), 4.42 – 4.35 (m, 1H), 3.96 (t, *J* = 6.5 Hz, 4H), 3.93 (t, *J* = 6.7 Hz, 2H), 3.74 (s, 3H), 3.24 – 3.19 (m, 2H), 1.88 (s, 1H), 1.79 (app p, *J* = 6.7 Hz, 4H), 1.75 – 1.63 (m, 3H), 1.61 – 1.50 (m, 2H), 1.50 – 1.41 (m, 6H), 1.38 – 1.21 (m, 84H), 0.88 (t, *J* = 7.0 Hz, 9H).

^{13}C NMR (126 MHz, $CDCl_3$): δ 172.8, 156.5, 156.0, 153.3 (2C), 138.3, 136.6, 131.1, 128.7 (3C), 128.3 (2C), 106.9 (2C), 73.5 (2C), 69.3 (2C), 67.7, 66.8, 53.6, 52.6, 40.6, 32.1 (3C), 30.5, 30.1, 30.0 – 29.7 (overlap, 29C), 29.6 – 29.4 (overlap, 8C), 26.3 (3C), 26.0, 22.8 (3C), 14.2 (3C).

Fmoc-Orn(TCbz)-OMe (**11**)

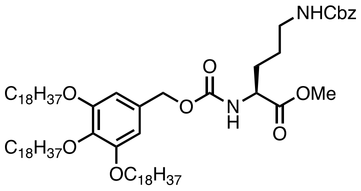

21

Prepared according to General Procedure 1 using Fmoc-Orn-OMe•HCl as the amino acid, **11** (130.8 mg, 0.100 mmol, >99%) was obtained as a pale-yellow powder.

Rf-value: 0.41 (hexane/EtOAc = 4:1, stained with phosphomolybdic acid)

HRMS (*m/z*): FAB $[M]^+$ calculated for $C_{83}H_{138}N_2O_9$: 1307.0402, found: 1307.0417.

$[\alpha]_D^{23}$ = +8.98 (c = 0.1, $CHCl_3$)

^{1}H NMR (500 MHz, $CDCl_3$): δ 7.76 (d, *J* = 7.6 Hz, 2H), 7.59 (br-s, 2H), 7.40 (t, *J* = 7.5 Hz, 2H), 7.31 (t, *J* = 7.5 Hz, 2H), 6.54 (d, *J* = 1.9 Hz, 2H), 5.40 (d, *J* = 8.1 Hz, 1H), 4.97 (s, 2H), 4.80 (br-s, 1H), 4.44 – 4.34 (m, 3H), 4.22 (t, *J* = 6.8 Hz, 1H), 3.99 – 3.88 (m, 6H), 3.75 (s, 3H), 3.22 (d, *J* = 6.8 Hz, 2H), 1.94 – 1.83 (m, 1H), 1.82 – 1.63 (m, 8H), 1.50 – 1.39 (m, 7H), 1.38 – 1.09 (m, 84H), 0.87 (app td, *J* = 7.0, 2.0 Hz, 9H).

^{13}C NMR (126 MHz, $CDCl_3$): δ 172.8, 156.6, 156.1, 153.3 (2C), 144.0, 143.8, 141.5, 141.4, 138.2, 131.4, 127.8 (2C), 127.2 (2C), 125.2 (2C), 120.1 (2C), 107.0 (2C), 73.5 (2C), 69.2 (2C), 67.3, 67.1, 53.6, 52.6, 47.3, 40.6, 32.1 (3C), 30.5, 30.0, 30.0 – 29.7 (overlap, 29C), 29.6 – 29.4 (overlap, 8C), 26.3 (3C), 26.1, 22.8 (3C), 14.2 (3C).

TCbz-Lys(Boc)-OMe (**22**)

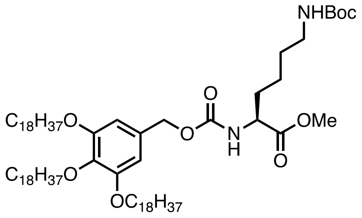

22

Prepared according to General Procedure 1 using Lys(Boc)-OMe•HCl as the amino acid, **22** (120.0 mg, 1.00 mmol, >99%) was obtained as a pale-yellow powder.

Rf-value: 0.41 (hexane/EtOAc = 4:1, stained with phosphomolybdic acid)

HRMS (*m/z*): FAB $[M]^+$ calculated for $C_{74}H_{138}N_2O_9$: 1199.0402, found: 1199.0404.

$[\alpha]_D^{23}$ = +3.66 (c = 0.1, $CHCl_3$)

^{1}H NMR (500 MHz, $CDCl_3$): δ 6.54 (s, 2H), 5.34 (br-d, *J* = 8.2 Hz, 1H), 4.98 (app q, *J* = 11.9 Hz, 2H), 4.55 (br-s, 1H), 4.40 – 4.33 (m, 1H), 3.96 (t, *J* = 6.5 Hz, 4H), 3.93 (t, *J* = 6.6 Hz, 2H), 3.74 (s, 3H), 3.14 – 3.04 (m, 2H), 1.90 – 1.64 (m, 9H), 1.52 – 1.43 (m, 7H), 1.42 (s, 9H), 1.39 – 1.20 (m, 86H), 0.88 (t, *J* = 6.8 Hz, 9H).

^{13}C NMR (126 MHz, $CDCl_3$): δ 173.0, 156.2, 156.1, 153.3 (2C), 138.2, 131.1, 107.0 (2C), 79.3, 77.4, 73.5 (2C), 69.2 (2C), 67.6, 53.8, 52.5, 40.1, 32.3, 32.1 (3C), 30.5, 30.0 – 29.7 (overlap, 29C), 29.6 – 29.4 (overlap, 8C), 28.5 (3C), 26.3 (3C), 22.8 (3C), 22.4, 14.2 (3C).

Fmoc-Lys(TCbz)-OMe (**23**)

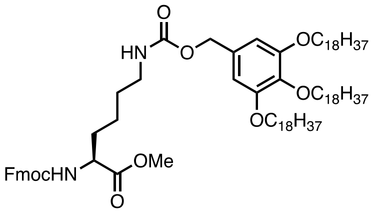

23

Prepared according to General Procedure 1 using Fmoc-Lys-OMe•HCl as the amino acid, **23** (132.2 mg, 0.100 mmol, >99%) was obtained as a pale-yellow powder.

Rf-value: 0.49 (hexane/EtOAc = 4:1, stained with phosphomolybdic acid)

HRMS (*m/z*): FAB $[M+Na]^+$ calculated for $C_{84}H_{140}N_2O_9Na$: 1344.0457, found: 1344.0457.

$[\alpha]_D^{23}$ = +12.3 (c = 0.1, $CHCl_3$)

^{1}H NMR (500 MHz, $CDCl_3$): δ 7.76 (d, *J* = 7.5 Hz, 2H), 7.60 (d, *J* = 7.5 Hz, 2H), 7.39 (t, *J* = 7.5 Hz, 2H), 7.31 (tt, *J* = 7.5, 1.2 Hz, 2H), 6.52 (s, 2H), 5.41 (d, *J* = 8.2 Hz, 1H), 5.01 – 4.93 (m, 2H), 4.81 (br-d, *J* = 6.4 Hz, 1H), 4.44 – 4.33 (m, 3H), 4.22 (t, *J* = 7.0 Hz, 1H), 3.98 – 3.87 (m, 6H), 3.75 (s, 3H), 3.24 – 3.16 (m, 2H), 1.91 – 1.82 (m, 1H), 1.82 – 1.66 (m, 6H), 1.59 – 1.49 (m, 3H), 1.49 – 1.37 (m, 8H), 1.25 (s, 84H), 0.88 (t, *J* = 6.9 Hz, 9H).

^{13}C NMR (126 MHz, $CDCl_3$): δ 173.0, 156.6, 156.1, 153.3 (2C), 144.0, 143.8, 141.4 (2C), 138.1, 131.5, 127.8 (2C), 127.2 (2C), 125.2 (2C), 120.1 (2C), 106.8 (2C), 73.5 (2C), 69.2 (2C), 67.2, 67.1, 53.7, 52.5, 47.3, 40.6, 32.1, 32.0 (3C), 30.4, 30.0 – 29.7 (overlap, 30C), 29.6 – 29.4 (overlap, 8C), 26.2 (3C), 22.8 (3C), 22.3, 14.2 (3C).

Cbz-His(TCbz)-OMe (**24**)

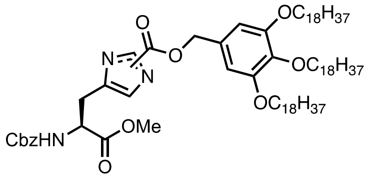

24

Prepared according to General Procedure 1 using Cbz-His-OMe as the amino acid, a 1.3:1 constitutional mixture of **24** (124.3 mg, 0.100 mmol, >99%) as a white powder was obtained by crystallization using MeCN as a polar solvent instead of MeOH. The isomers were separated by thin-layer preparative TLC (hexane/EtOAc = 2:1) for characterization purposes.

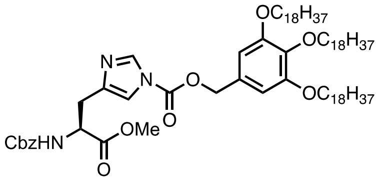

24a

Rf-value: 0.52 (hexane/EtOAc = 2:1, stained with phosphomolybdic acid)

HRMS (*m/z*): FAB $[M]^+$ calculated for $C_{77}H_{131}N_3O_9$: 1241.9885, found: 1241.9895.

$[\alpha]_D^{23}$ = +13.0 (c = 0.1, $CHCl_3$)

^{1}H NMR (500 MHz, $CDCl_3$): δ 8.02 (s, 1H), 7.37 – 7.28 (m, 5H), 7.19 (s, 1H), 6.60 (s, 2H), 6.04 (d, *J* = 8.3 Hz, 1H), 5.26 (s, 2H), 5.10 (s, 2H), 4.66 (dt, *J* = 8.3, 5.1 Hz, 1H), 3.99 – 3.92 (m, 6H), 3.71 (s, 3H), 3.10 (dd, *J* = 15.0, 5.1 Hz, 1H), 3.04 (dd, *J* = 15.0, 5.1 Hz, 1H), 1.80 (app p, *J* = 6.7 Hz, 4H), 1.73 (app q, *J* = 7.1 Hz, 2H), 1.46 (app p, *J* = 7.5 Hz, 6H), 1.38 – 1.18 (m, 84H), 0.88 (t, *J* = 6.8 Hz, 9H).

^{13}C NMR (126 MHz, $CDCl_3$): δ 172.0, 156.1, 153.5 (2C), 148.5, 139.1, 139.0, 137.2, 136.5, 128.8, 128.6 (3C), 128.2 (2C), 114.8, 107.8 (2C), 73.6 (2C), 70.6, 69.4 (2C), 67.0, 53.6, 52.6, 32.1 (3C), 30.5, 30.1, 30.0 – 29.7 (overlap, 29C), 29.6 – 29.4 (overlap, 8C), 26.3 (3C), 22.8 (3C), 14.2 (3C).

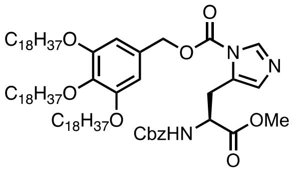

24b

Rf-value: 0.39 (hexane/EtOAc = 2:1, stained with phosphomolybdic acid)

HRMS (*m/z*): FAB $[M]^+$ calculated for $C_{77}H_{131}N_3O_9$: 1241.9885, found: 1241.9872.

$[\alpha]_D^{23}$ = +6.00 (c = 0.1, $CHCl_3$)

^{1}H NMR (500 MHz, $CDCl_3$): δ 8.09 (s, 1H), 7.38 – 7.28 (m, 5H), 6.82 (d, *J* = 1.0 Hz, 1H), 6.61 (s, 2H), 5.34 (d, *J* = 8.5 Hz, 1H), 5.32 – 5.25 (m, 2H), 5.03 (s, 2H), 4.75 (td, *J* = 8.5, 5.4 Hz, 1H), 3.99 – 3.92 (m, 6H), 3.70 (s, 3H), 3.46 (dd, *J* = 14.9, 5.4 Hz, 1H), 3.17 (dd, *J* = 14.9, 5.4 Hz, 1H), 1.84 – 1.76 (m, 4H), 1.76 – 1.70 (m, 2H), 1.50 – 1.41 (m, 6H), 1.38 – 1.19 (m, 84H), 0.88 (t, *J* = 7.0 Hz, 9H).

^{13}C NMR (126 MHz, $CDCl_3$): δ 172.0, 171.3, 155.8, 153.5 (2C), 149.3, 139.0, 138.9, 136.2, 131.5, 128.7 (2C), 128.3, 128.2 (2C), 127.5, 107.6 (2C), 73.6 (2C), 70.5, 69.4 (2C), 67.2, 53.1, 52.7, 32.1 (3C), 30.5, 30.0 – 29.7 (overlap, 29C), 29.6 – 29.4 (overlap, 8C), 29.2, 26.3 (3C), 22.8 (3C), 14.3 (3C).

Fmoc-D-Cys(TCbz)-OAllyl (**25**)

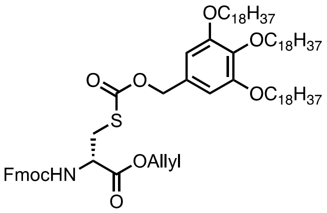

25

Prepared according to General Procedure 1 using Fmoc-D-Cys-OAllyl as the amino acid, **25** (119.1 mg, 0.900 mmol, 90%) was obtained as a pale-yellow powder after purification by thin-layer preparative TLC (hexane/EtOAc = 4:1).

Rf-value: 0.49 (hexane/EtOAc = 4:1, stained with phosphomolybdic acid)

HRMS (*m/z*): FAB $[M]^+$ calculated for $C_{83}H_{135}NO_9S$: 1321.9858, found: 1321.9867.

$[\alpha]_D^{23}$ = –7.46 (c = 0.1, $CHCl_3$)

^{1}H NMR (500 MHz, $CDCl_3$): δ 7.76 (d, *J* = 7.5 Hz, 2H), 7.60 (d, *J* = 7.5 Hz, 2H), 7.43 – 7.37 (m, 2H), 7.31 (tt, *J* = 7.5, 1.3 Hz, 2H), 6.50 (s, 2H), 5.91 (ddt, *J* = 16.5, 11.0, 5.9 Hz, 1H), 5.68 (d, *J* = 8.0 Hz, 1H), 5.35 (dd, *J* = 16.5, 1.7 Hz, 1H), 5.27 (dd, *J* = 11.0, 1.7 Hz, 1H), 5.15 – 5.05 (m, 2H), 4.72 – 4.60 (m, 3H), 4.42 (dd, *J* = 10.6, 7.2 Hz, 1H), 4.35 (dd, *J* = 10.6, 7.2 Hz, 1H), 4.25 (t, *J* = 7.2 Hz, 1H), 3.93 (app q, *J* = 6.4 Hz, 6H), 3.48 (dd, *J* = 14.4, 4.7 Hz, 1H), 3.36 (dd, *J* = 14.4, 6.2 Hz, 1H), 1.77 (app p, *J* = 6.7 Hz, 4H), 1.74 – 1.68 (m, 2H), 1.49 – 1.40 (m, 6H), 1.37 – 1.20 (m, 84H), 0.88 (t, *J* = 6.9 Hz, 9H).

^{13}C NMR (126 MHz, $CDCl_3$): δ 170.5, 169.9, 155.8, 153.4 (2C), 143.92, 143.88, 141.4 (2C), 138.6, 131.4, 129.7, 127.9 (2C), 127.2 (2C), 125.3 (2C), 120.1 (2C), 119.4, 107.3 (2C), 73.5 (2C), 70.3, 69.2 (2C), 67.4, 66.7, 54.0, 47.2, 33.3, 32.1 (3C), 30.5, 30.1 – 29.7 (overlap, 29C), 29.6 – 29.4 (overlap, 8C), 26.2 (3C), 22.8 (3C), 14.2 (3C).

TCbz-D-Phe-OAllyl (**26**)

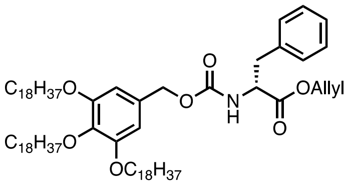

26

Prepared according to General Procedure 1 using D-Phe-OAllyl•TsOH as the amino acid, **26** (1.15 g, 1.00 mmol, >99%) was obtained as a pale-pink powder.

Rf-value: 0.50 (hexane/EtOAc = 4:1, stained with phosphomolybdic acid)

HRMS (*m/z*): FAB $[M]^+$ calculated for $C_{74}H_{129}NO_7$: 1143.9769, found: 1143.9773.

$[\alpha]_D^{23}$ = –8.42 (c = 0.1, $CHCl_3$)

^{1}H NMR (500 MHz, $CDCl_3$): δ 7.30 – 7.19 (m, 3H), 7.13 – 7.08 (m, 2H), 6.52 (s, 2H), 5.85 (ddd, *J* = 16.4, 10.8, 5.5 Hz, 1H), 5.34 – 5.17 (m, 3H), 4.98 (s, 2H), 4.68 (app q, *J* = 6.2 Hz, 1H), 4.61 (d, *J* = 6.2 Hz, 2H), 3.97 – 3.91 (m, 6H), 3.18 – 3.07 (m, 2H), 1.79 (app p, *J* = 6.8 Hz, 4H), 1.72 (app q, *J* = 7.0 Hz, 2H), 1.46 (app p, *J* = 7.2 Hz, 6H), 1.37 – 1.20 (m, 84H), 0.88 (t, *J* = 6.9 Hz, 9H).

^{13}C NMR (126 MHz, $CDCl_3$): δ 175.8, 155.9, 153.3 (2C), 138.2, 135.9, 132.5, 132.4, 129.5 (2C), 128.8, 128.7 (2C), 127.2, 106.9 (2C), 73.6 (2C), 69.2 (2C), 67.6, 64.2, 54.9, 37.9, 32.1 (3C), 30.5, 30.0 – 29.7 (overlap, 29C), 29.6 – 29.4 (overlap, 8C), 26.3 (3C), 22.8 (3C), 14.3 (3C).

3-4. Typical Procedure for Cleavage of the TCbz Group

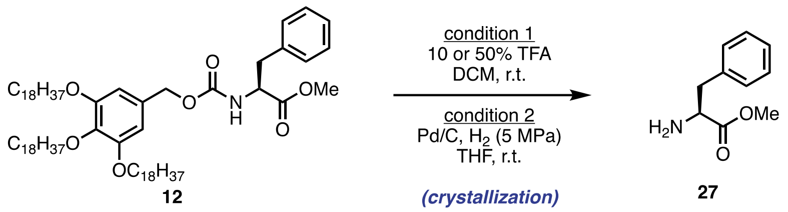

• Condition 1

To a glass vessel charged with **12** (36.0 mg, 32.2 μmol) was added DCM/TFA (9:1 or 1:1 v/v, 0.644 mL, 0.05 M) at room temperature under air. After stirring at room temperature for 24 h, the reaction mixture was concentrated *in vacuo* and the resulting residue was azeotropically concentrated using PhMe three times. The resulting residue was dissolved in DCM (0.644 mL, 0.05 M) and cooled down to 0 °C. The resulting solution was poured into cold MeOH (3.22 mL, 0.01 M). The resulting suspension was stirred for 30 min at 0 ºC and filtered through a pad of Celite (washed with excess MeOH). The filtrate was concentrated *in vacuo* and the resulting residue was dissolved in $CHCl_3$ (5 mL). The resulting solution was washed with sat. aq. $NaHCO_3$ (5 mL and the organic phase was concentrated *in vacuo*, dried under high vacuum, yielding Phe-OMe (**27**) as a white solid.

^{1}H NMR of the obtained **27 was identical to the commercially available material.*

• Condition 2

To a solution of **12** (125.4 mg, 0.112 mmol) in THF (2.24 mL, 0.05 M) was added Pd/C (10 wt% loading, 51.3 mg, 48.2 μmol, 43 mol%) at room temperature under air. The reaction mixture was pressurized to 5 MPa under a H_2 atmosphere in a Parr bomb. After stirring at room temperature for 60 h under the H_2 atmosphere (5 MPa), the reaction mixture was poured into cold MeOH (11.2 mL, 0.01 M). The resulting suspension was stirred for 30 min at 0 ºC and filtered through a pad of Celite (washed with excess MeOH). The filtrate was concentrated *in vacuo* and dried under high vacuum, yielding Phe-OMe (**27**) (17.6 mg, 98.2 μmol, 88%) as a pale-yellow solid. For characterization purposes, the solid collected on the filter cake was dissolved in $CHCl_3$ and then eluted by suction filtration using excess $CHCl_3$. The resulting filtrate was concentrated *in vacuo* and dried under high vacuum, yielding TAGa-H (**S1**) (103 mg, 0.114 mmol, >99%) as a white powder.

^{1}H NMR of the obtained **27 was identical to the commercially available material.*

TAGa-H (**S1**)

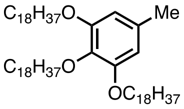

S1

Rf-value: 0.31 (hexane/EtOAc = 50:1, stained with phosphomolybdic acid phosphomolybdic acid)

HRMS (*m/z*): FAB $[M]^+$ calculated for $C_{61}H_{116}O_3$: 896.8924, found: 896.8932.

^{1}H NMR (500 MHz, $CDCl_3$): δ 6.35 (s, 2H), 3.94 (t, *J* = 6.5 Hz, 4H), 3.90 (t, *J* = 6.6 Hz, 2H), 2.27 (s, 3H), 1.78 (app p, *J* = 6.6 Hz, 4H), 1.75 – 1.69 (m, 2H), 1.49 – 1.41 (m, 6H), 1.37 – 1.21 (m, 84H), 0.88 (t, *J* = 7.0 Hz, 9H).

^{13}C NMR (126 MHz, $CDCl_3$): δ 153.0 (2C), 136.2, 133.1, 107.7 (2C), 73.6, 69.2 (2C), 32.1 (3C), 30.5, 30.0 – 29.7 (overlap, 29C), 29.6 – 29.4 (overlap, 8C), 26.31, 26.26 (3C), 22.8, 21.9 (3C), 14.3 (3C).

3-5. Typical Procedure for Deprotection of TCbz-Amino Acids

• General procedure 2 for cleavage of the Fmoc group

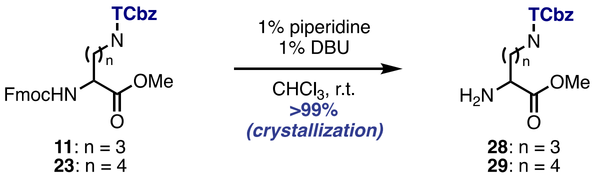

To a glass vessel charged with Fmoc-Orn(TCbz)-OMe (**11**) or Fmoc-Lys(TCbz)-OMe (**23**) was added a solution of piperidine/DBU/$CHCl_3$ (1:1:98 v/v, 0.05 M) at room temperature under air. After stirring at room temperature until the consumption of the starting material (judged by TLC), the reaction mixture was cooled down to 0 °C and poured into cold MeOH (0.01 M). The resulting suspension was stirred for 30 min at 0 °C and filtered through a pad of Celite (washed with excess MeOH). The product collected on the filter cake was dissolved in $CHCl_3$ and then eluted by suction filtration using excess $CHCl_3$. The resulting filtrate was concentrated *in vacuo* and dried under high vacuum, yielding the corresponding amine **28** or **29** as a yellow powder.

Orn(TCbz)-OMe (**28**)

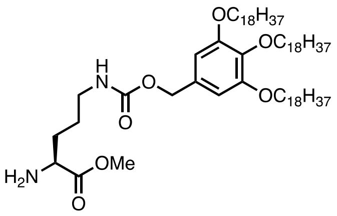

28

Prepared according to General Procedure 2 using Fmoc-Orn(TCbz)-OMe (**11**) (32.7 mg, 25.3 µmol) as the starting material, **28** (27.1 mg, 25.0 µmol, 99%) was obtained as a yellow powder.

Rf-value: 0.45 ($CHCl_3$/MeOH = 10:1, stained with phosphomolybdic acid)

HRMS (*m/z*): FAB $[M+H]^+$ calculated for $C_{68}H_{129}N_2O_7$: 1085.9800, found: 1085.9783.

$[\alpha]_D^{22}$ = +7.22 (c = 0.1, $CHCl_3$)

^{1}H NMR (500 MHz, $CDCl_3$): δ 6.53 (d, *J* = 1.9 Hz, 2H), 5.05 – 4.95 (br-m, 1H), 4.97 (s, 2H), 3.99 – 3.89 (m, 6H), 3.71 (s, 3H), 3.45 (d, *J* = 7.4 Hz, 1H), 3.22 (app t, *J* = 6.8 Hz, 2H), 1.78 (app p, *J* = 7.2 Hz, 4H), 1.72 (app q, *J* = 6.9 Hz, 2H), 1.66 – 1.53 (m, 4H), 1.49 – 1.41 (m, 6H), 1.38 – 1.19 (m, 84H), 0.87 (app td, *J* = 7.0, 1.9 Hz, 9H).

^{13}C NMR (126 MHz, $CDCl_3$): δ 176.4, 156.5, 153.3 (2C), 138.2, 131.5, 107.0 (2C), 73.6 (2C), 69.2 (2C), 67.2, 54.2, 52.2, 40.9, 32.1 (3C), 30.5, 30.0 – 29.7 (overlap, 29C), 29.6 – 29.4 (overlap, 8C), 26.4, 26.3 (3C), 26.2, 22.8 (3C), 14.3 (3C).

Lys(TCbz)-OMe (**29**)

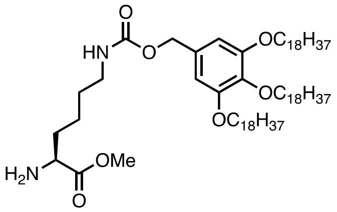

29

Prepared according to General Procedure 2 using Fmoc-Lys(TCbz)-OMe (**23**) (132.2 mg, 0.100 mmol) as the starting material, **29** (110.0 mg, 0.100 mmol, >99%) was obtained as a yellow powder.

Rf-value: 0.45 ($CHCl_3$/MeOH = 10:1, stained with phosphomolybdic acid)

HRMS (*m/z*): FAB $[M+H]^+$ calculated for $C_{69}H_{131}N_2O_7$: 1099.9956, found: 1099.9958.

$[\alpha]_D^{22}$ = +8.52 (c = 0.1, $CHCl_3$)

^{1}H NMR (500 MHz, $CDCl_3$): δ 6.54 (s, 2H), 4.97 (s, 2H), 4.76 (s, 1H), 3.96 (t, *J* = 6.5 Hz, 4H), 3.92 (t, *J* = 6.4 Hz, 2H), 3.72 (s, 3H), 3.44 (t, *J* = 6.5 Hz, 1H), 3.20 (app q, *J* = 6.5 Hz, 2H), 1.78 (app p, *J* = 6.9 Hz, 4H), 1.72 (app q, *J* = 7.1 Hz, 2H), 1.64 – 1.50 (m, 4H), 1.49 – 1.38 (m, 8H), 1.38 – 1.21 (m, 84H), 0.88 (app td, *J* = 7.0, 1.2 Hz, 9H).
(*NH protons were not detected.)

^{13}C NMR (126 MHz, $CDCl_3$): δ 176.5, 156.5, 153.3 (2C), 138.1, 131.5, 106.9 (2C), 77.4, 73.5 (2C), 69.2 (2C), 67.1, 54.3, 52.0, 40.9, 34.5, 32.0 (3C), 30.4, 30.0 – 29.7 (overlap, 29C), 29.6 – 29.4 (overlap, 8C), 26.2 (3C), 22.9, 22.8 (3C), 14.2 (3C).

• Cleavage of the Bn group: TCbz-Trp-OH (**30**)

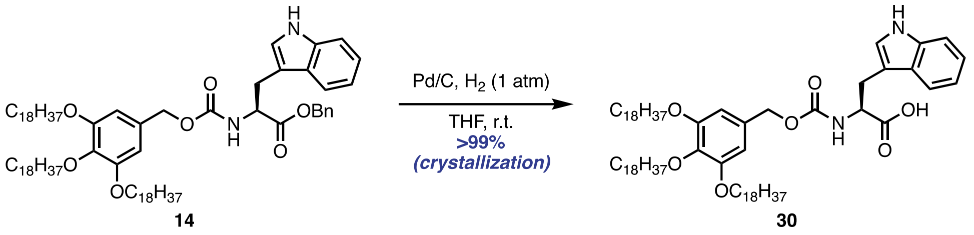

To a solution of TCbz-Trp-OBn (**14**) (30.8 mg, 25.0 µmol) in THF (0.500 mL, 0.05 M) was added Pd/C (10 wt% loading, 13.0 mg, 12.2 µmol, 49 mol%) at room temperature under air. The reaction vessel was carefully evacuated and backfilled with a H_2 atmosphere (balloon, 1 atm). After stirring at room temperature for 8 h under a H_2 atmosphere (1 atm), the reaction mixture was cooled down to 0 °C and poured into cold MeOH (25.0 mL, 0.01 M). The resulting suspension was stirred for 30 min at 0 ºC and filtered through a pad of Celite (washed with excess MeOH). The product collected on the filter cake was dissolved in $CHCl_3$ and then eluted by suction filtration using excess $CHCl_3$. The resulting filtrate was concentrated *in vacuo* and dried under high vacuum, yielding **30** (28.6 mg, 25.0 µmol, >99%) as a brown powder.

Rf-value: 0.46 ($CHCl_3$/MeOH = 10:1, stained with phosphomolybdic acid)

HRMS (*m/z*): FAB $[M+Na]^+$ calculated for $C_{73}H_{126}N_2O_7Na$: 1165.9463, found: 1165.9469.

$[\alpha]_D^{22}$ = +10.9 (c = 0.1, $CHCl_3$)

^{1}H NMR (500 MHz, $CDCl_3$): δ 8.10 (s, 1H), 7.57 (d, *J* = 8.0 Hz, 1H), 7.35 (d, *J* = 8.0 Hz, 1H), 7.19 (t, *J* = 7.5 Hz, 1H), 7.09 (t, *J* = 7.5 Hz, 1H), 6.97 (s, 1H), 6.51 (s, 2H), 5.25 (d, *J* = 7.9 Hz, 1H), 5.02 (d, *J* = 12.1 Hz, 1H), 4.96 (d, *J* = 12.1 Hz, 1H), 4.77 – 4.71 (m, 1H), 3.93 (t, *J* = 6.5 Hz, 6H), 3.42 – 3.29 (m, 2H), 1.82 – 1.69 (m, 6H), 1.50 – 1.41 (m, 6H), 1.38 – 1.17 (m, 84H), 0.88 (t, *J* = 6.9 Hz, 9H).

^{13}C NMR (126 MHz, $CDCl_3$): δ 176.1, 156.2, 153.3 (2C), 138.2, 136.2, 131.2, 127.8, 123.3, 122.3, 119.8, 118.7, 111.4, 109.6, 107.0 (2C), 73.6 (2C), 69.3 (2C), 67.6, 54.7, 32.1 (3C), 30.5, 30.0 – 29.7 (overlap, 29C), 29.6 – 29.4 (overlap, 8C), 27.7, 26.3 (3C), 22.8 (3C), 14.3 (3C).

• Cleavage of the TMSE group: TCbz-Ile-OH (**31**)

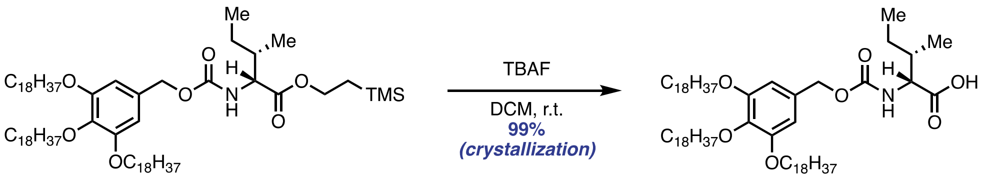

To a solution of TCbz-Ile-OTMSE (**16**) (13.4 mg, 11.4 µmol) in DCM (0.228 mL, 0.05 M) was added TBAF (1.0 M in THF, 13.7 µL, 13.7 µmol, 1.2 equiv) at room temperature under air. After stirring at room temperature for 10 h, the reaction mixture was cooled down to 0 °C and poured into cold MeOH (25.0 mL, 0.01 M). The resulting suspension was stirred for 30 min at 0 ºC and filtered through a pad of Celite (washed with excess MeOH). The product collected on the filter cake was dissolved in $CHCl_3$ and then eluted by suction filtration using excess $CHCl_3$. The resulting filtrate was concentrated *in vacuo* and dried under high vacuum, yielding **31** (12.1 mg, 11.3 µmol, 99%) as a white powder.

Rf-value: 0.40 ($CHCl_3$/MeOH = 10:1, stained with phosphomolybdic acid)

HRMS (*m/z*): FAB $[M]^+$ calculated for $C_{68}H_{127}NO_7$: 1069.9613, found: 1069.9611.

$[\alpha]_D^{22}$ = –31.0 (c = 0.1, $CHCl_3$)

^{1}H NMR (500 MHz, $CDCl_3$): δ 6.54 (s, 2H), 5.23 (d, *J* = 8.9 Hz, 1H), 5.03 (d, *J* = 12.3 Hz, 1H), 4.97 (d, *J* = 12.3 Hz, 1H), 4.39 (br-s, 1H), 3.99 – 3.89 (m, 6H), 2.00 – 1.91 (m, 1H), 1.78 (app q, *J* = 6.8 Hz, 4H), 1.73 (app t, *J* = 7.2 Hz, 2H), 1.52 – 1.41 (m, 6H), 1.38 – 1.21 (m, 84H), 1.20 – 1.09 (m, 2H), 1.01 – 0.96 (m, 3H), 0.96 – 0.90 (m, 3H), 0.87 (app dt, *J* = 7.3, 3.4 Hz, 9H).

^{13}C NMR (126 MHz, $CDCl_3$): δ 176.4, 156.6, 153.4 (2C), 138.3, 131.1, 106.9 (2C), 73.6 (2C), 69.3 (2C), 67.8, 57.1, 37.6, 32.1 (3C), 31.1, 30.5, 30.0 – 29.7 (overlap, 29C), 29.6 – 29.4 (overlap, 8C), 26.4, 26.3 (2C), 22.8 (3C), 14.6, 14.3 (3C), 11.9.

• Cleavage of the Me group: Fmoc-Orn(TCbz)-OH (**32**)

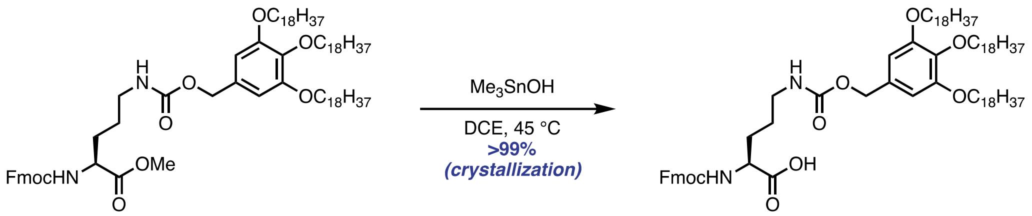

11 **32**

To a solution of Fmoc-Orn(TCbz)-OMe (**11**) (65.4 mg, 50.0 μmol) in DCE (1.00 mL, 0.05 M) was added Me_3SnOH (27.1 mg, 0.150 mmol, 3.0 equiv) at room temperature under air. After stirring at 45 °C for 45 h, the reaction mixture was cooled down to 0 °C and poured into cold MeOH (5.00 mL, 0.01 M). The resulting suspension was stirred for 30 min at 0 ºC and filtered through a pad of Celite (washed with excess MeOH). The product collected on the filter cake was dissolved in $CHCl_3$ and then eluted by suction filtration using excess $CHCl_3$. The resulting filtrate was concentrated *in vacuo* and dried under high vacuum, yielding **32** (65.4 mg, 50.0 μmol, >99%) as a white powder.

Rf-value: 0.38 ($CHCl_3$/MeOH = 10:1, stained with phosphomolybdic acid)

HRMS (*m/z*): FAB $[M+Na]^+$ calculated for $C_{82}H_{136}N_2O_9Na$: 1316.0144, found: 1316.0149.

$[\alpha]_D^{22}$ = +7.24 (c = 0.1, $CHCl_3$)

^{1}H NMR (500 MHz, $CDCl_3$): δ 7.73 (br-s, 2H), 7.58 (br-s, 2H), 7.37 (br-s, 2H), 7.32 (br-s, 2H), 6.53 (br-s, 2H), 5.56 (br-s, 1H), 4.97 (br-s, 2H), 4.39 (br-s, 2H), 4.19 (br-s, 1H), 3.92 (br-s, 6H), 3.22 (br-s, 2H), 1.91 (br-s, 1H), 1.81 – 1.67 (m, 6H), 1.66 – 1.52 (m, 3H), 1.50 – 1.37 (m, 6H), 1.36 – 1.05 (m, 86H), 0.88 (t, *J* = 6.9 Hz, 9H).

^{13}C NMR (126 MHz, $CDCl_3$): δ 179.3, 158.2, 157.0, 153.3 (2C), 144.0, 143.8, 141.4 (2C), 138.2, 131.3, 127.8 (2C), 127.2 (2C), 125.2 (2C), 120.1 (2C), 107.0 (2C), 73.6 (2C), 69.3 (2C), 67.5, 67.2, 53.7, 47.2, 40.5, 34.1, 32.1 (3C), 30.5, 30.0 – 29.7 (overlap, 29C), 29.6 – 29.4 (overlap, 8C), 26.3 (3C), 24.8, 22.8 (3C), 14.3 (3C).

• Cleavage of the Allyl group: TCbz-D-Phe-OH (**33**)

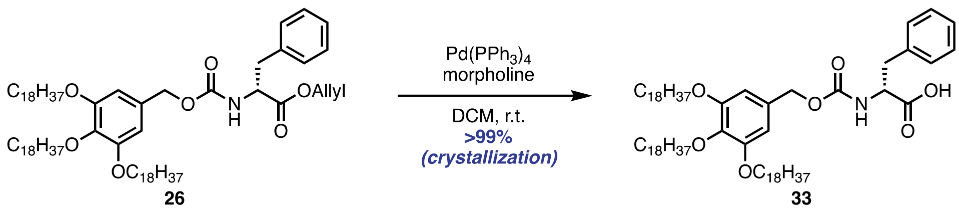

To a solution of TCbz-D-Phe-OAllyl (**26**) (1.14 g, 1.00 mmol) in DCE (20.0 mL, 0.05 M) was added $Pd(PPh_3)_4$ (115.6 mg, 0.100 mmol, 10 mol%) and morpholine (0.413 mL, 5.00 mmol, 5.0 equiv) at room temperature under air. After stirring at room temperature for 2 h, the reaction mixture was cooled down to 0 °C and poured into cold MeOH (100 mL, 0.01 M). The resulting suspension was stirred for 30 min at 0 ºC and filtered through a pad of Celite (washed with excess MeOH). The product collected on the filter cake was dissolved in $CHCl_3$ and then eluted by suction filtration using excess $CHCl_3$. The resulting filtrate was concentrated *in vacuo* and dried under high vacuum, yielding **33** (1.10 g, 0.996 mmol, >99%) as a yellow powder.

Rf-value: 0.44 ($CHCl_3$/MeOH = 10:1, stained with phosphomolybdic acid)

HRMS (*m/z*): FAB $[M]^+$ calculated for $C_{71}H_{125}NO_7$: 1103.9456, found: 1103.9456.

$[\alpha]_D^{22}$ = –6.32 (c = 0.1, $CHCl_3$)

^{1}H NMR (500 MHz, $CDCl_3$): δ 7.31 – 7.11 (m, 5H), 6.51 (s, 2H), 5.17 (br-s, 1H), 4.98 (s, 2H), 4.66 (br-s, 1H), 3.93 (app q, *J* = 6.8 Hz, 6H), 3.25 – 3.18 (m, 1H), 3.16 – 3.08 (m, 1H), 1.82 – 1.75 (m, 4H), 1.73 (t, *J* = 7.4 Hz, 2H), 1.49 – 1.41 (m, 6H), 1.36 – 1.17 (m, 84H), 0.88 (t, *J* = 6.8 Hz, 9H).

^{13}C NMR (126 MHz, $CDCl_3$): δ 173.7, 156.0, 153.4 (2C), 138.3, 135.5, 131.0, 129.5 (2C), 128.9 (2C), 127.5, 107.0 (2C), 73.6 (2C), 69.3 (2C), 67.8, 54.6, 37.8, 32.1 (3C), 30.5, 30.0 – 29.7 (overlap, 29C), 29.6 – 29.4 (overlap, 8C), 26.3 (3C), 22.8 (3C), 14.3 (3C).

3-6. Syntheses of TCbz-Supported Oligopeptides

Depsipeptide fragment **S2**

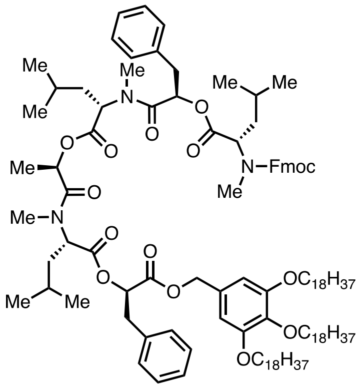

S2

Prepared by following our previous work.[7] In the previous work, we used depsipeptide fragment **S2** (white powder) for one-pot synthesis without characterization and those data are provided herewith.

Rf-value: 0.32 (hexane/EtOAc = 4:1, stained with phosphomolybdic acid)

HRMS (*m/z*): FAB [M+Na]$^+$ calculated for $C_{118}H_{185}N_3O_{15}Na$: 1907.3703, found: 1907.3691.

$[\alpha]_D^{22}$ = –8.70 (c = 0.1, $CHCl_3$)

^{1}H NMR (500 MHz, $CDCl_3$): δ 7.76 (t, *J* = 11.5 Hz, 2H), 7.63 (t, *J* = 7.8 Hz, 1H), 7.56 – 7.06 (m, 15H), 6.45 (s, 2H), 5.55 – 5.37 (m, 1H), 5.36 – 5.11 (m, 3H), 5.08 – 4.90 (m, 2H), 4.73 – 4.64 (*rotamer, m, 0.4H), 4.47 – 4.24 (m, 3H), 4.14 (*rotamer, m, 0.4H), 3.98 – 3.88 (m, 6H), 3.25 – 3.11 (m, 2H), 3.11 – 2.95 (m, 5H), 2.91 – 2.83 (m, 3H), 2.83 – 2.71 (m, 3H), 1.84 – 1.54 (m, 13H), 1.52 – 1.40 (m, 11H), 1.39 – 1.21 (m, 86H), 0.96 – 0.76 (m, 27H).

^{13}C NMR (126 MHz, $CDCl_3$): δ 174.1, 171.5 (*rotamer), 171.4, 171.3 (*rotamer), 171.21, 171.16 (*rotamer), 170.9, 170.6 (*rotamer), 170.3, 169.3, 169.2 (*rotamer), 168.8 (*rotamer), 157.0, 156.5 (*rotamer), 153.4 (*rotamer, 2C), 153.3 (2C), 144.23, 144.18 (*rotamer), 144.04, 144.00 (*rotamer), 141.45, 141.42, 141.3 (*rotamer, 2C), 138.6 (*rotamer), 138.4, 136.1 (*rotamer), 136.0, 135.8 (*rotamer), 135.7, 130.0, 129.9 (2C), 129.7, 129.8 (*rotamer), 129.7 (*rotamer), 129.5 (2C), 129.4 (*rotamer, 2C), 129.3 (*rotamer), 128.72 (*rotamer), 128.69 (*rotamer, 2C), 128.6 (2C), 128.5 (*rotamer, 2C), 128.4 (2C), 127.8 (2C), 127.7 (*rotamer, 2C), 127.24 (*rotamer), 127.18 (2C), 127.1, 127.0 (*rotamer), 125.3, 125.2, 125.1 (*rotamer), 125.0 (*rotamer), 120.1 (2C), 120.0 (*rotamer, 2C), 107.6 (*rotamer, 2C), 107.1 (2C), 74.0, 73.9 (*rotamer), 73.5 (2C), 72.3, 72.1, 71.3, 69.2 (2C), 68.0, 67.95 (*rotamer), 67.87 (*rotamer), 67.8, 67.7, 57.9 (*rotamer), 57.5 (*rotamer), 56.5, 55.1, 54.9 (*rotamer), 54.4, 47.4, 47.3 (*rotamer), 40.6, 38.8, 37.8, 37.7, 37.6 (*rotamer), 37.3, 37.12, 37.08 (*rotamer), 32.1 (3C), 31.9 (*rotamer), 31.8, 31.2 (*rotamer), 31.0, 30.6 (*rotamer), 30.5, 30.3, 30.0 –

29.7 (overlap, 29C), 29.6 – 29.4 (overlap, 8C), 26.3 (3C), 24.9, 24.8, 24.72 (*rotamer), 24.68 (*rotamer), 23.4, 23.3, 23.2 (*rotamer), 22.8 (3C), 21.41, 21.35, 21.2 (*rotamer), 16.6, 16.5 (*rotamer), 14.2 (3C).

Depsipeptide-CO_2H **S3**

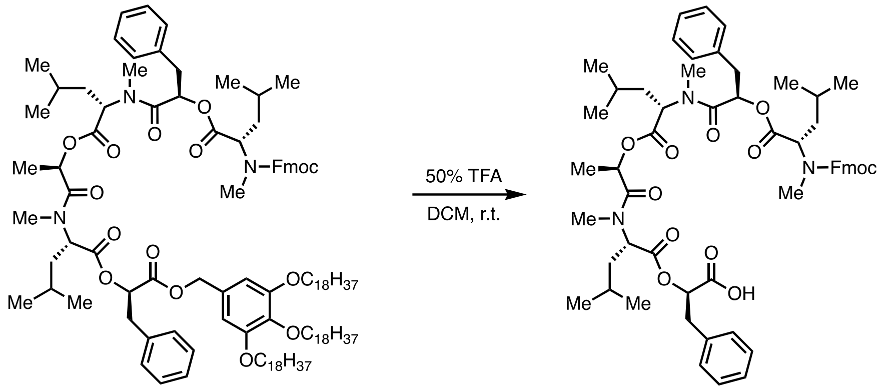

S2 **S3**

To a glass vessel charged with depsipeptide fragment **S2** (188.6 mg, 0.100 mmol) was added DCM/TFA (1:1 v/v, 2.00 mL, 0.05 M) at room temperature under air. After stirring at room temperature for 1 h, the reaction mixture was concentrated *in vacuo* and the resulting residue was azeotropically concentrated using PhMe three times. The resulting residue was dissolved in DCM (2.00 mL, 0.05 M) and cooled down to 0 °C. The resulting solution was poured into cold MeCN (10.0 mL, 0.01 M). The resulting suspension was stirred for 30 min at 0 °C and filtered through a pad of Celite (washed with excess MeCN). The filtrate was concentrated *in vacuo* and dried under high vacuum. This crude material was used in the next reaction without further purification.

TCbz-oligodepsipeptide **34**

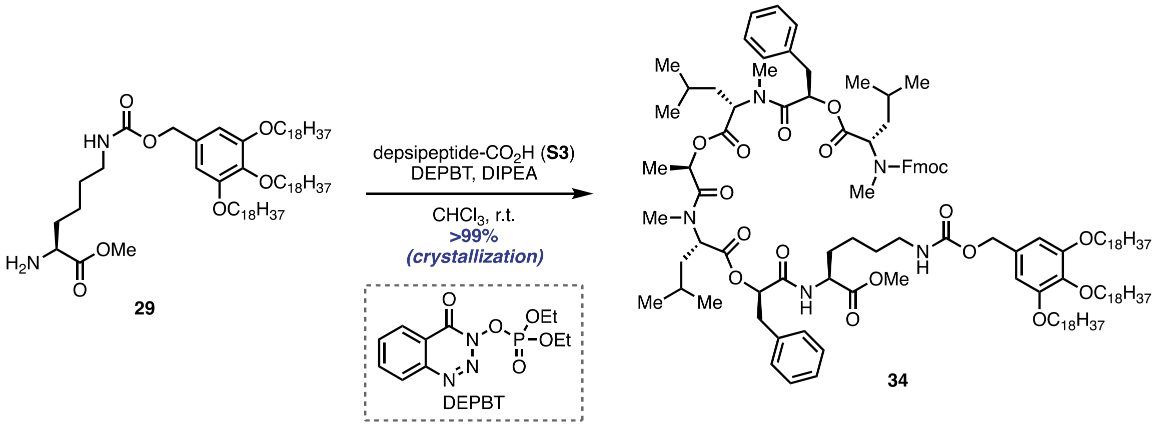

29 **34**

To a solution of Lys(TCbz)-OMe (**29**) (78.6 mg, 71.5 μmol) and the crude depsipeptide **S3** (ca. 99.0 mg, 0.100 mmol, 1.4 equiv) in $CHCl_3$ (1.43 mL, 0.05 M) was added DEPBT (42.7 mg, 0.143 mmol, 2.0 equiv) and DIPEA (43.5 µL, 0.250 mmol, 3.5 equiv) at room temperature under air. After stirring at room temperature for 12 h, the reaction mixture was cooled down to 0 °C and poured into cold MeOH (7.15 mL, 0.01 M). The resulting suspension was stirred for 30 min at 0 ºC and filtered through a pad of Celite (washed with excess MeOH). The product collected on the filter cake was dissolved in $CHCl_3$ and then eluted by suction filtration using excess $CHCl_3$. The resulting filtrate was concentrated *in vacuo* and dried under high vacuum, yielding **34** (148.0 mg, 71.4 μmol, >99%) as a white powder.

Rf-value: 0.19 (hexane/EtOAc = 4:1, stained with phosphomolybdic acid)

HRMS (*m/z*): FAB $[M+Na]^+$ calculated for $C_{126}H_{199}N_5O_{18}Na$: 2093.4708, found: 2093.4702.

$[\alpha]_D^{22}$ = –14.9 (c = 0.1, $CHCl_3$)

^{1}H NMR (500 MHz, $CDCl_3$): δ 7.79 – 7.68 (m, 2H), 7.66 – 7.52 (m, 1H), 7.47 – 7.34 (m, 3H), 7.33 – 7.05 (m, 12H), 6.52 (s, 2H), 5.50 – 5.35 (m, 1H), 5.30 – 5.22 (m, 1H), 5.19 – 5.08 (m, 2H), 5.08 – 4.90 (m, 3H), 4.74 – 4.59 (*rotamer, m, 0.4H), 4.56 – 4.46 (m, 1H), 4.44 – 4.35 (m, 2H), 4.35 – 4.23 (m, 1H), 4.19 – 4.12 (*rotamer, m, 0.4H), 4.01 – 3.83 (m, 6H), 3.74 – 3.60 (m, 1H), 3.67 (app d, *J* = 3.3 Hz, 3H), 3.26 – 3.09 (m, 4H), 3.09 – 3.00 (m, 4H), 2.97 (app dd, *J* = 8.4, 3.3 Hz, 3H), 2.91 (app d, *J* = 3.3 Hz, 3H), 2.88 – 2.82 (m, 1H), 2.86 (app t, *J* = 4.4 Hz, 3H), 1.91 – 1.68 (m, 9H), 1.68 – 1.54 (m, 7H), 1.54 – 1.39 (m, 11H), 1.38 – 1.10 (m, 86H), 1.00 – 0.77 (m, 27H).

^{13}C NMR (126 MHz, $CDCl_3$): δ 172.4, 171.7 (*rotamer), 171.6 (*rotamer), 171.5, 171.3, 171.2 (*rotamer), 171.1, 170.4, 170.1, 169.4, 169.3 (*rotamer), 156.9, 156.5, 156.4 (*rotamer), 153.2 (2C), 144.2 (*rotamer), 144.1, 144.0, 143.9 (*rotamer), 141.4, 141.35 (*rotamer), 141.29 (*rotamer), 138.0, 136.41, 136.37 (*rotamer), 135.6 (*rotamer), 135.5, 131.7, 129.6 (2C), 129.52 (*rotamer), 129.48 (2C), 129.44 (*rotamer), 128.7 (*rotamer), 128.6 (2C), 128.5 (*rotamer), 128.4 (2C), 127.8 (2C), 127.7 (*rotamer), 127.2 (2C), 127.1 (2C), 127.0, 125.2, 125.15, 125.09 (*rotamer), 125.05 (*rotamer), 120.1 (2C), 120.0 (*rotamer), 106.8 (2C), 75.4, 73.5 (2C), 72.2 (*rotamer), 72.0, 69.1 (2C), 68.0 (*rotamer), 67.9 (*rotamer), 67.8, 67.0, 60.0, 57.7, 56.5, 55.0 (*rotamer), 54.9, 52.2, 51.9, 47.3, 40.8, 38.7, 38.0, 37.7 (*rotamer), 37.6, 37.4, 37.3, 35.8, 32.0 (3C), 31.6 (*rotamer), 31.5, 30.9, 30.4, 30.2, 30.0 – 29.7 (overlap, 29C), 29.6 – 29.4 (overlap, 8C), 29.1, 26.2 (3C), 24.8, 24.71, 24.67, 23.4 (*rotamer), 23.23, 23.20, 22.9, 22.8 (3C), 21.6, 21.5 (*rotamer), 21.4, 21.3, 21.2, 15.5, 15.4 (*rotamer), 14.2 (3C).

Pentapeptide-NH_2 **S5**

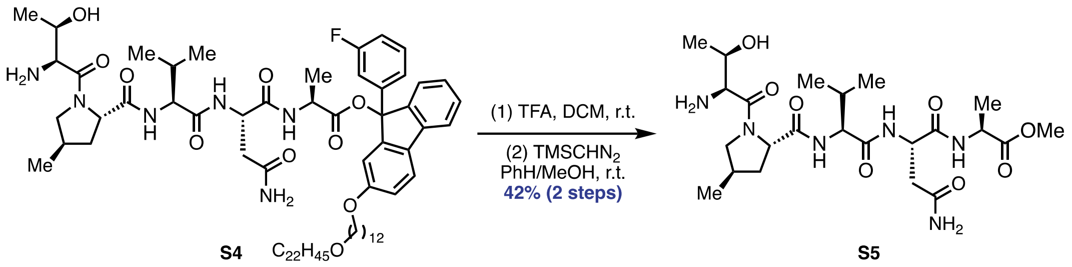

Kozupeptine fragment **S4** was prepared by following our previous work.[8] To a glass vessel charged with **S4** (133.8 mg, 0.100 mmol) was added DCM/TFA (4:1 v/v, 2.00 mL, 0.05 M) at room temperature under air. After stirring at room temperature for 1 h, the reaction mixture was concentrated *in vacuo* and the resulting residue was azeotropically concentrated using PhMe three times. The resulting residue was dissolved in DCM (2.00 mL, 0.05 M) and cooled down to 0 °C. The resulting solution was poured into cold MeOH (10.0 mL, 0.01 M). The resulting suspension was stirred for 30 min at 0 ºC and filtered through a pad of Celite (washed with excess MeOH). The filtrate was concentrated *in vacuo* and dried under high vacuum. This crude material was used in the next reaction without further purification.

To a solution of the crude material in PhH/MeOH (9:1 v/v, 10.0 mL, 0.01 M) was added $TMSCHN_2$ (0.6 M in hexane, 0.333 mL, 0.200 mmol, 2.0 equiv) at room temperature under air. After stirring at room temperature for 1 h, the reaction mixture was concentrated *in vacuo*. The resulting residue was purified by reverse-phase HPLC (a 20 min linear gradient, 10–30% MeCN/H_2O with 0.05% TFA, 8 mL/min flow rate, UV = 210 nm detection, 10–12 min retention time), yielding **S5** (22.0 mg, 41.6 µmol, 42% over 2 steps) as a white solid.

Rf-value: 0.05 ($CHCl_3$/MeOH = 1:1, stained with phosphomolybdic acid)

HRMS (*m/z*): ESI $[M+H]^+$ calculated for $C_{23}H_{41}N_6O_8$: 529.2986, found: 529.2983.

$[\alpha]_D^{22}$ = –52.2 (c = 0.1, $CHCl_3$)

^{1}H NMR (500 MHz, CD_3OD): δ 4.73 (td, *J* = 6.4, 3.2 Hz, 1H), 4.68 (dd, *J* = 8.7, 4.2 Hz, 1H), 4.39 (q, *J* = 7.0 Hz, 1H), 4.13 (d, *J* = 6.5 Hz, 1H), 4.19 – 4.01 (m, 2H), 3.88 (dd, *J* = 9.9, 7.0 Hz, 1H), 3.71 (s, 3H), 3.32 – 3.26 (m, 1H), 2.79 – 2.66 (m, 2H), 2.53 (app tt, *J* = 14.0, 5.9 Hz, 1H), 2.19 (dqd, *J* = 12.1, 6.4, 3.4 Hz, 1H), 2.15 – 2.04 (m, 1H), 1.88 (app dt, *J* = 12.9, 8.6 Hz, 1H), 1.38 (d, *J* = 7.5 Hz, 3H), 1.36 (d, *J* = 7.8 Hz, 3H), 1.08 (dd, *J* = 6.4, 3.4 Hz, 3H), 1.04 – 0.94 (m, 6H).

^{13}C NMR (126 MHz, CD_3OD): δ 174.9, 174.4, 174.2, 173.3, 172.6, 168.2, 67.7, 61.5, 60.5, 58.3, 55.6, 52.8, 51.2, 49.8, 37.9, 37.6, 33.9, 31.9, 19.9, 19.7, 18.7, 17.6, 17.5.

TCbz-oligopeptide **35**

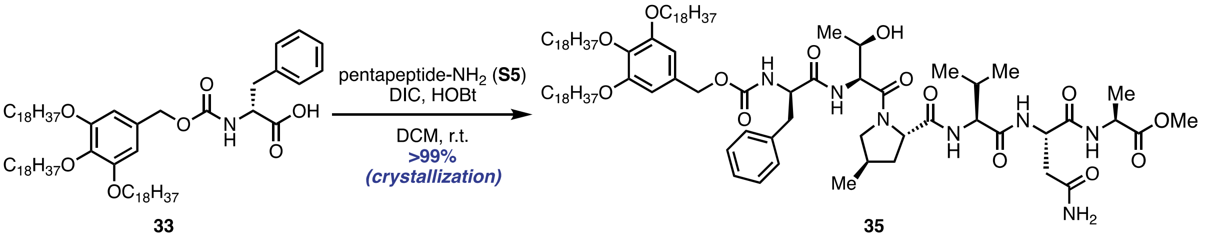

To a solution of TCbz-D-Phe-OH (**33**) (39.5 mg, 35.8 μmol) and **S5** (20.8 mg, 39.3 μmol, 1.1 equiv) in DCM (0.716 mL, 0.05 M) was added DIC (6.6 μL, 42.9 μmol, 1.2 equiv) and HOBt (5.8 mg, 42.9 μmol, 1.2 equiv) at room temperature under air. After stirring at room temperature for 18 h, the reaction mixture was cooled down to 0 °C and poured into cold MeOH (3.58 mL, 0.01 M). The resulting suspension was stirred for 30 min at 0 °C and filtered through a pad of Celite (washed with excess MeOH). The product collected on the filter cake was dissolved in $CHCl_3$ and then eluted by suction filtration using excess $CHCl_3$. The resulting filtrate was concentrated *in vacuo* and dried under high vacuum, yielding **35** (57.8 mg, 35.7 μmol, >99%) as a white powder.

Rf-value: 0.38 ($CHCl_3$/MeOH = 4:1, stained with phosphomolybdic acid)

HRMS (*m/z*): FAB $[M+Na]^+$ calculated for $C_{94}H_{163}N_7O_{14}Na$: 1637.2156, found: 1637.2156.

$[\alpha]_D^{24}$ = –4.76 (c = 0.1, $CHCl_3$)

^{1}H NMR (500 MHz, $CDCl_3$) at 40 °C: δ 8.01 (br-s, 1H), 7.76 – 7.46 (br-m, 1H), 7.36 (br-s, 1H), 7.31 – 7.13 (m, 5H), 6.87 (br-s, 1H), 6.68 (br-s, 1H), 6.52 (br-s, 1H), 6.47 (s, 2H), 5.35 (br-s, 1H), 5.07 – 4.94 (m, 2H), 4.94– 4.77 (m, 2H), 4.67 – 4.42 (m, 3H), 4.41 – 4.25 (m, 2H), 4.25 – 4.02 (m, 2H), 3.99 – 3.86 (m, 7H), 3.70 (s, 3H), 3.35 (app t, *J* = 8.9 Hz, 1H), 3.12 (br-s, 1H), 3.09 – 2.74 (m, 1H), 2.53 – 2.39 (m, 2H), 2.28 – 2.20 (m, 1H), 2.16 (m, 1H), 2.06 – 1.88 (m, 1H), 1.79 (app t, *J* = 7.1 Hz, 4H), 1.76 – 1.68 (m, 2H), 1.46 (app q, *J* = 7.5 Hz, 6H), 1.42 (d, *J* = 7.2 Hz, 3H), 1.39 – 1.19 (m, 87H), 1.10 (d, *J* = 6.6 Hz, 3H), 0.94 (d, *J* = 6.8 Hz, 3H), 0.88 (t, *J* = 6.9 Hz, 12H).

^{13}C NMR (126 MHz, $CDCl_3$) at 40 °C: δ 172.7, 172.3, 171.6, 171.2, 170.4, 161.2, 156.9, 156.3, 153.4 (2C), 138.6, 136.4, 132.5, 131.1, 129.5, 128.9 (2C), 127.2 (2C), 107.2, 73.6 (2C), 69.5 (2C), 68.1, 67.8, 61.5, 59.2, 56.2, 54.6, 52.5, 50.3, 48.9, 48.3, 39.0, 36.9, 33.2, 33.1, 32.1 (3C), 30.5, 30.0 – 29.7 (overlap, 29C), 29.6 – 29.7 (overlap, 8C), 26.3 (3C), 22.8 (3C), 19.7 (2C), 19.2, 18.4, 17.7, 17.5, 14.2 (3C).

Bottromycin fragment **36**

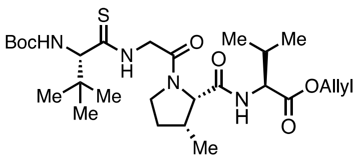

36

Prepared by following our previous work.[9] In the previous work, we used bottromycin fragment **36** (white solid) for investigation of unsuccessful macrocyclization and thus did not include characterization and those data are provided herewith.

Rf-value: 0.70 ($CHCl_3$/MeOH = 10:1, stained with phosphomolybdic acid)

HRMS (*m/z*): ESI $[M+H]^+$ calculated for $C_{27}H_{47}N_4O_6S$: 555.3216, found: 555.3217.

$[\alpha]_D^{22}$ = +23.2 (c = 0.1, $CHCl_3$)

^{1}H NMR (500 MHz, $CDCl_3$): δ 9.20 (br-s, 1H), 6.99 (br-s, 1H), 5.88 (ddt, *J* = 16.5, 11.1, 5.9 Hz, 1H), 5.76 (br-d, *J* = 10.0 Hz, 1H), 5.36 – 5.26 (m, 1H), 5.23 (d, *J* = 11.1 Hz, 1H), 4.75 (br-s, 1H), 4.63 (dd, *J* = 12.9, 6.0 Hz, 1H), 4.60 – 4.48 (m, 2H), 4.57 (dd, *J* = 9.2, 5.1 Hz, 1H), 4.41 (dd, *J* = 19.1, 5.1 Hz, 1H), 4.29 – 4.21 (m, 1H), 3.78 (app t, *J* = 9.2 Hz, 1H), 3.52 – 3.44 (m, 1H), 2.40 (dq, *J* = 13.6, 7.1 Hz, 1H), 2.23 – 2.15 (m, 1H), 2.14 – 2.01 (m, 2H), 1.44 (s, 9H), 1.11 (d, *J* = 6.8 Hz, 3H), 0.93 (s, 9H), 0.88 (d, *J* = 6.8 Hz, 3H), 0.81 (d, *J* = 6.9 Hz, 3H).

^{13}C NMR (126 MHz, $CDCl_3$): δ 202.4, 171.6, 170.3, 165.2, 155.8, 132.0, 118.7, 80.0, 65.6, 64.8, 63.5, 56.9, 49.0, 46.5, 36.08, 36.06, 31.8, 30.5, 28.6 (3C), 26.8 (3C), 19.2, 17.2, 14.3.

Tetrapeptide-CO_2H **S6**

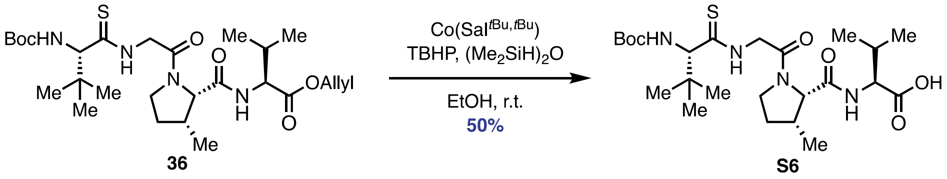

To a solution of bottromycin fragment **36** (55.5 mg, 0.100 mmol) and Co(Sal^{tBu,tBu}) (1.21 mg, 2.00 µmol, 2 mol%) in EtOH (0.714, mL, 0.14 M) was added TBHP (70% in H_2O, 0.3 µL, 2.00 µmol, 2 mol%) at room temperature. After stirring at room temperature for 5 min, to the reaction mixture was added $(Me_2SiH)_2O$ (17.7 µL, 0.100 mmol, 1.0 equiv). After stirring at room temperature for 48 h, the reaction mixture was concentrated *in vacuo*. The resulting residue was purified by thin-layer preparative TLC ($CHCl_3$/MeOH = 10:1), yielding **S6** (25.7 mg, 49.9 µmol, 50%) as a brown solid.

Rf-value: 0.21 ($CHCl_3$/MeOH = 10:1, stained with phosphomolybdic acid)

HRMS (*m/z*): ESI $[M–H]^-$ calculated for $C_{24}H_{41}N_4O_6S$: 513.2747, found: 513.2747.

$[\alpha]_D^{22}$ = –13.7 (c = 0.1, $CHCl_3$)

^{1}H NMR (500 MHz, CD_3OD): δ 6.30 (*rotamer, s, 0.4H), 4.66 – 4.53 (m, 1H), 4.51 – 4.41 (m, 1H), 4.38 – 4.17 (m, 3H), 4.13 (*rotamer, s, 0.4H), 3.81 – 3.71 (m, 1H), 3.67 – 3.55 (m, 1H), 3.51 – 3.40 (*rotamer, m, 0.4H), 2.68 – 2.55 (*rotamer, m, 0.4H), 2.53 – 2.39 (m, 1H), 2.26 – 2.11 (m, 1H), 2.11 – 1.94 (m, 1H), 1.93 – 1.70 (m, 1H), 1.43 (app d, *J* = 5.7 Hz, 9H), 1.09 (app dt, *J* = 11.2, 6.4 Hz, 3H), 1.05 – 0.92 (m, 15H).
(*NH and CO_2H protons were not detected.)

^{13}C NMR (126 MHz, CD_3OD): δ 204.3, 172.6, 168.0, 167.8, 157.3, 80.6, 68.5, 65.0, 59.8, 47.3, 39.6, 37.1, 36.2, 33.4, 32.5, 32.3 (*rotamer), 31.3 (*rotamer), 28.7 (3C), 27.4 (3C), 20.3 (*rotamer), 19.9, 18.7 (*rotamer), 18.4, 15.2 (*rotamer), 14.9.

Tetrapeptide-NH_2 **S7**

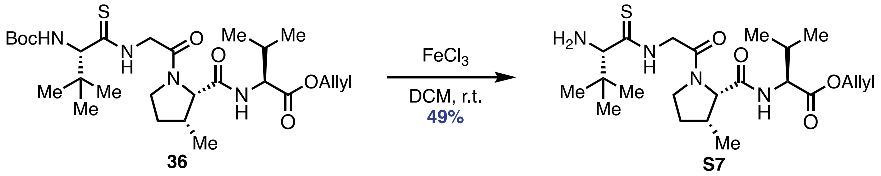

36 **S7**

To a solution of bottromycin fragment **36** (55.5 mg, 0.100 mmol) in DCM (2.00 mL, 0.05 M) was added $FeCl_3$ (32.4 mg, 0.200 mmol, 2.0 equiv) at room temperature under air. After stirring at room temperature for 2 h, the reaction mixture was quenched by dropwise addition of 1% aq. H_3PO_4 (ca. 500 µL) until brown color turned to slightly yellow–colorless. After adjusting pH to 7 by slow addition of DIPEA, the resulting suspension was filtered through a pad of Celite (washed with DCM). The filtrate was concentrated *in vacuo*. The resulting residue was purified by thin-layer preparative TLC ($CHCl_3$/MeOH = 10:1), yielding **S7** (22.1 mg, 48.6 µmol, 49%) as a yellow oil.

Rf-value: 0.39 ($CHCl_3$/MeOH = 10:1, stained with phosphomolybdic acid)

HRMS (*m/z*): ESI $[M+H]^+$ calculated for $C_{22}H_{39}N_4O_4S$: 455.2692, found: 455.2692.

$[\alpha]_D^{22}$ = –145 (c = 0.1, $CHCl_3$)

^{1}H NMR (500 MHz, CD_3OD): δ 6.00 – 5.90 (m, 1H), 5.40 – 5.31 (m, 1H), 5.27 – 5.20 (m, 1H), 4.68 – 4.58 (m, 2H), 4.56 – 4.27 (m, 4H), 3.87 – 3.57 (m, 3H), 3.50 – 3.40 (*rotamer, m, 0.4H), 2.70 – 2.60 (*rotamer, m, 0.4H), 2.53 – 2.43 (m, 0.6H), 2.28 – 2.12 (m, 1H), 2.12 – 1.95 (m, 1H), 1.88 (app q, *J* = 11.1 Hz, 0.6H), 1.79 – 1.66 (*rotamer, m, 0.4H), 1.18 – 1.13 (m, 3H), 1.12 – 0.91 (m, 15H).

*This compound (**S7**) is not stable as a solution in deuterium solvents ($CDCl_3$ and CD_3OD) and we could not record ^{13}C NMR. The product was immediately used in the next reaction after the identification by ^{1}H NMR.

TCbz-oligopeptide **37**

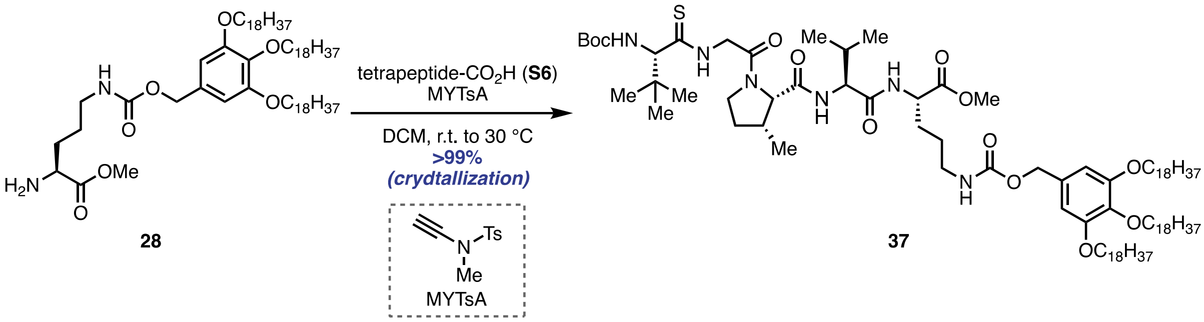

28 37

To a solution of tetrapeptide-CO_2H **S6** (51.5 mg, 0.100 mmol, 1.6 equiv) in DCM (0.313 mL, 0.2 M) was added *N*-methylynetoluenesulfonamide (MYTsA; 26.2 mg, 0.125 mmol, 2.0 equiv) at room temperature under air. After stirring at room temperature for 2 h, to the reaction mixture was added Orn(TCbz)-OMe (**28**) (67.9 mg, 62.5 μmol) at room temperature. After stirring at 30 °C for 46 h, the reaction mixture was cooled down to 0 °C and poured into cold MeOH (1.57 mL, 0.025 M). The resulting suspension was stirred for 30 min at 0 °C and filtered through a pad of Celite (washed with excess MeOH). The product collected on the filter cake was dissolved in $CHCl_3$ and then eluted by suction filtration using excess $CHCl_3$. The resulting filtrate was concentrated *in vacuo* and dried under high vacuum, yielding **37** (98.9 mg, 62.5 μmol, >99%) as a pale-yellow powder.

Rf-value: 0.49 ($CHCl_3$/MeOH = 20:1, stained with phosphomolybdic acid)

HRMS (*m/z*): FAB $[M+Na]^+$ calculated for $C_{92}H_{168}N_6O_{12}Na$: 1604.2339, found: 1604.2354.

$[\alpha]_D^{24}$ = +14.0 (c = 0.1, $CHCl_3$)

^{1}H NMR (500 MHz, $CDCl_3$: δ 6.76 (br-s, 1H), 6.61 – 6.50 (m, 2H), 5.75 – 5.62 (m, 1H), 5.20 (* rotamer, br-s, 0.3H), 5.12 – 4.91 (m, 2H), 4.66 – 4.20 (m, 4H), 4.15 – 4.00 (m, 1H), 3.99 – 3.88 (m, 7H), 3.88 – 3.78 (*rotamer, m, 0.6H), 3.77 – 3.65 (m, 3H), 3.63 – 3.55 (*rotamer, m, 0.4H), 3.50 – 3.39 (*rotamer, m, 0.6H), 3.29 – 3.06 (m, 2H), 2.60 – 2.37 (m, 1H), 2.24 – 2.01 (m, 1H), 2.00 – 1.83 (m, 1H), 1.80 – 1.58 (m, 2H), 1.78 (app p, *J* = 6.7 Hz, 4H), 1.72 (app q, *J* = 7.1 Hz, 2H), 1.57 – 1.48 (m, 1H), 1.50 – 1.39 (m, 15H), 1.38 – 1.18 (m, 87H), 1.15 – 1.03 (m, 3H), 1.03 – 0.90 (m, 15H), 0.87 (t, *J* = 6.9 Hz, 9H).

^{13}C NMR (126 MHz, $CDCl_3$): δ 202.8, 172.4, 171.3, 170.5, 156.7, 155.8, 153.3 (2C), 138.1, 131.6, 106.8 (2C), 79.9, 73.5 (2C), 70.0, 69.2 (2C), 67.1, 65.7, 64.0, 59.0, 52.4, 52.0, 48.3, 46.4, 40.6, 36.5, 35.9, 32.0 (3C), 30.5 (2C), 30.0 – 29.7 (overlap, 30C), 29.6 – 29.4 (8C), 28.5 (3C), 26.9, 26.8 (3C), 26.3 (3C), 25.9, 22.8 (3C), 19.5, 18.0, 14.6, 14.2 (3C).

TCbz-oligopeptide **38**

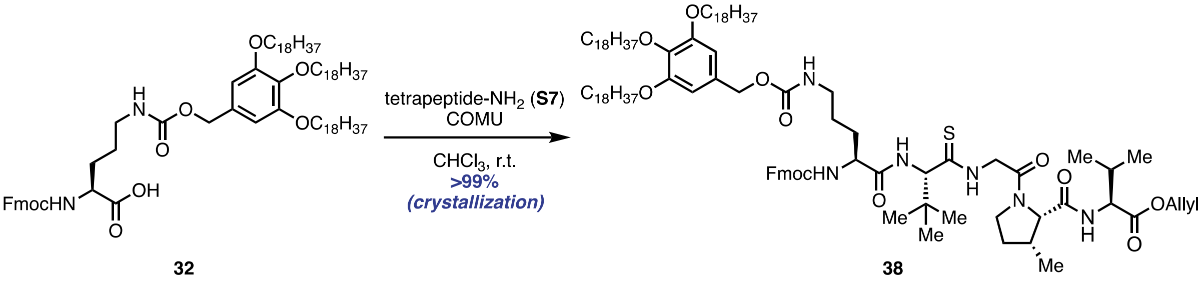

To a solution of Orn(TCbz)-OH (**32**) (107.8 mg, 83.3 µmol) and **S7** (47.1 mg, 0.100 mmol, 1.2 equiv) in $CHCl_3$ (1.67 mL, 0.05 M) was added COMU (42.8 mg, 0.100 mmol, 1.2 equiv) at room temperature under air. After stirring at room temperature for 7.5 h, the reaction mixture was cooled down to 0 °C and poured into cold MeOH (8.33 mL, 0.01 M). The resulting suspension was stirred for 30 min at 0 °C and filtered through a pad of Celite (washed with excess MeOH). The product collected on the filter cake was dissolved in $CHCl_3$ and then eluted by suction filtration using excess $CHCl_3$. The resulting filtrate was concentrated *in vacuo* and dried under high vacuum, yielding **38** (144.2 mg, 83.3 µmol, >99%) as a white powder.

Rf-value: 0.57 ($CHCl_3$/MeOH = 20:1, stained with phosphomolybdic acid)

HRMS (*m/z*): FAB $[M+Na]^+$ calculated for $C_{104}H_{172}N_6O_{12}Na$: 1752.2652, found: 1752.2660.

$[\alpha]_D^{22}$ = +7.18 (c = 0.1, $CHCl_3$)

^{1}H NMR (500 MHz, $CDCl_3$: δ 7.75 (d, *J* = 7.5 Hz, 2H), 7.64 (t, *J* = 7.2 Hz, 2H), 7.38 (td, *J* = 7.5, 2.7 Hz, 2H), 7.30 – 7.22 (m, 2H), 6.57 (s, 2H), 6.18 (br-s, 1H), 5.85 (ddt, *J* = 16.5, 11.0, 5.2 Hz, 1H), 5.31 – 5.23 (m, 1H), 5.22 – 5.15 (m, 1H), 5.14 – 4.92 (br-m, 2H), 5.09 (d, *J* = 11.9 Hz, 1H), 5.04 (d, *J* = 11.9 Hz, 1H), 4.97 (t, *J* = 6.5 Hz, 1H), 4.73 – 4.54 (m, 4H), 4.50 (br-d, *J* = 19.4 Hz, 1H), 4.44 – 4.29 (m, 3H), 4.27 – 4.19 (m, 2H), 3.96 (t, *J* = 6.4 Hz, 4H), 3.92 (t, *J* = 6.6 Hz, 2H), 3.82 – 3.73 (m, 1H), 3.58 – 3.51 (m, 1H), 3.50 – 3.43 (m, 1H), 3.19 – 3.09 (m, 1H), 2.39 – 2.27 (m, 1H), 2.15 – 1.95 (m, 3H), 1.78 (app p, *J* = 6.5 Hz, 4H), 1.72 (app q, *J* = 7.0 Hz, 2H), 1.69 – 1.59 (m, 4H), 1.59 – 1.50 (m, 2H), 1.50 – 1.40 (m, 6H), 1.37 – 1.20 (m, 84H), 1.06 (d, *J* = 6.7 Hz, 3H), 0.93 (s, 9H), 0.88 (t, *J* = 6.8 Hz, 9H), 0.81 (d, *J* = 6.8 Hz, 3H), 0.69 (d, *J* = 6.8 Hz, 3H).

^{13}C NMR (126 MHz, $CDCl_3$): δ 201.9, 172.3, 171.9, 170.1, 165.0, 157.4, 156.7, 153.2 (2C), 144.0, 141.4, 138.1, 131.7, 131.6, 127.7 (2C), 127.1 (2C), 125.5, 125.3, 120.0 (2C), 119.0, 107.0 (2C), 73.5 (2C), 69.1 (2C), 67.5, 67.0, 65.7, 64.0, 63.2, 56.8, 52.8, 51.8, 49.2, 47.3, 46.5, 40.7, 39.3, 36.1, 35.6, 32.0 (3C), 31.9, 31.6, 30.4, 30.3, 30.0 – 29.7 (overlap, 29C), 29.6 – 29.4 (overlap, 8C), 27.0 (3C), 26.4, 26.2 (3C), 22.8 (3C), 19.0, 17.4, 14.3, 14.2 (3C).

3-7. Solid/Hydrophobic-Tag Relay Synthesis of Calpinactam

Fmoc-D-*allo*-Ile-OTrt-resin (**S9**)

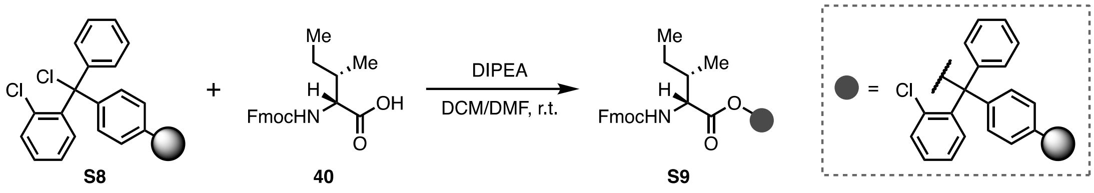

To a polypropylene stopcock-frit tube charged with 2-chlorotrityl chloride resin (**S8**) (1.30 mmol/g, 121 mg, 0.157 mmol) was added DCM (2.00 mL, 0.079 M) at room temperature to swell the resin for 30 min prior to the reaction. After removal of DCM, to the tube was added a solution of DIPEA (0.110 mL, 0.628 mmol, 4.0 equiv) and Fmoc-D-*allo*-Ile-OH (**40**) (110 mg, 0.314 mmol, 2.0 equiv) in DCM/DMF (4:1 v/v, 2.00 mL, 0.079 M) at room temperature. After shaking at room temperature for 1 h, the reaction mixture was quenched with MeOH (0.100 mL). After shaking at room temperature for 5 min, the resulting solution was removed by suction filtration through the stopcock. The residual resin on the frit was washed with DCM (2.00 mL, shaking for 1 min) twice and DMF (2.00 mL, shaking for 1 min) twice. After removal of the washing solvents, the residual resin (**S9**) was used in the next reaction without further purification.

• General procedure 3 for removal of the Fmoc group of resin-supported amino acids

To the tube containing the residual resin was added 2% DBU in DMF (2.00 mL, 0.079M) at room temperature. After shaking at room temperature for 5 min, the resulting solution was removed by suction filtration through the stopcock. This process was repeated two more times. The residual resin on the frit was washed with DCM (2.00 mL, shaking for 1 min) twice and DMF (2.00 mL, shaking for 1 min) twice, and DCM (2.00 mL, shaking for 1 min) twice. After removal of the washing solvents, the residual resin was used in the next reaction without further purification.

D-*allo*-Ile-OTrt-resin (**S10**)

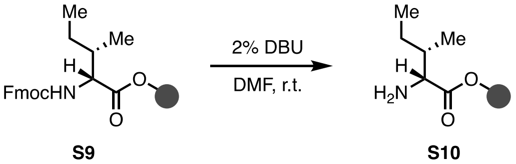

Prepared according to General Procedure 3 from Fmoc-D-*allo*-Ile-OTrt-resin (**S9**).

• General procedure 4 for condensation of resin-supported amines with an amino acid

To the tube containing the residual resin was added a solution of the corresponding Fmoc-amino acid (0.471 mmol, 3.0 equiv) in DCM/DMF (4:1 v/v, 2.00 mL, 0.079 M) at room temperature. To the resulting mixture was added PyBOP (270 mg, 0.518 mmol, 3.3 equiv) and DIPEA (0.164 mL, 0.942 mmol, 6.0 equiv) at room temperature. After shaking at room temperature for 1 h, the resulting solution was removed by suction filtration through the stopcock. The residual resin on the frit was washed with DCM (2.00 mL, shaking for 1 min) twice and DMF (2.00 mL, shaking for 1 min) twice. After removal of the washing solvents, the residual resin was used in the next reaction without further purification.

Fmoc-dipeptide-OTrt-resin (**S11**)

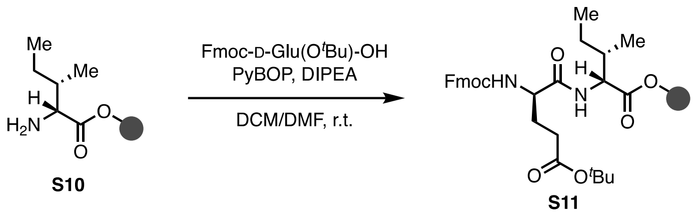

Prepared according to General Procedure 4 from D-*allo*-Ile-OTrt-resin (**S10**) using Fmoc-D-Glu(O^{t}Bu)-OH (208 mg, 0.471 mmol, 3.0 equiv) as the amino acid.

Fmoc-tripeptide-OTrt-resin (**S13**)

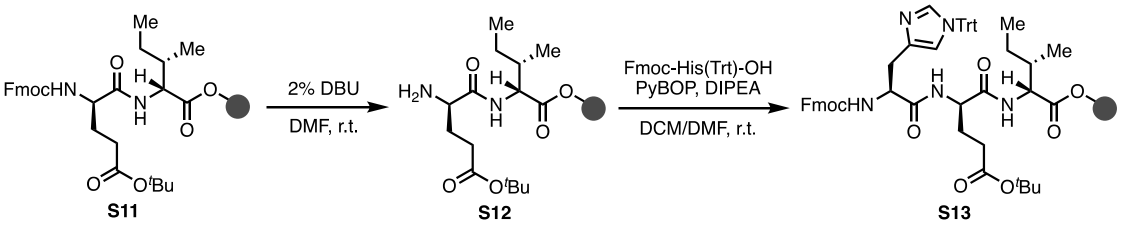

Prepared according to General Procedure 3 from Fmoc-dipeptide-OTrt-resin (**S11**), followed by General Procedure 4 using Fmoc-His(Trt)-OH (290 mg, 0.471 mmol, 3.0 equiv) as the amino acid

Fmoc-tetrapeptide-OTrt-resin (**S14**)

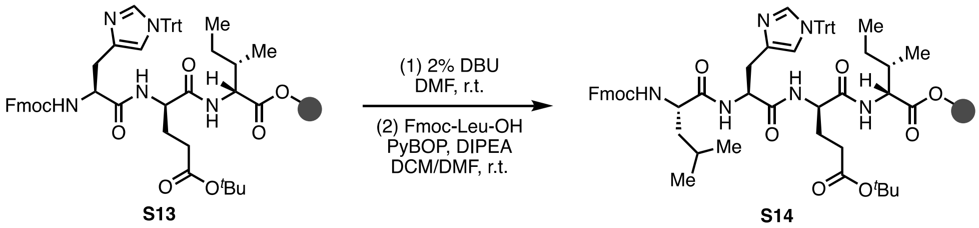

Prepared according to General Procedure 3 from Fmoc-tripeptide-OTrt-resin (**S13**), followed by General Procedure 4 using Fmoc-Leu-OH (166 mg, 0.471 mmol, 3.0 equiv) as the amino acid.

Tetrapeptide-OTrt-resin (**44**)

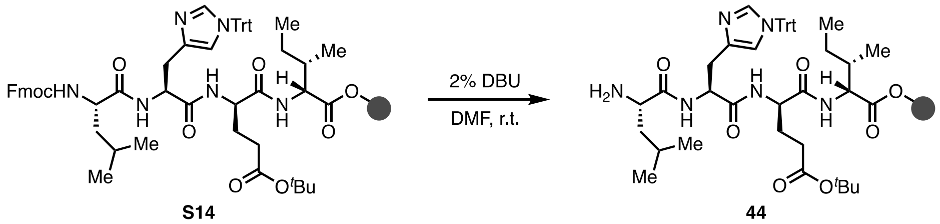

Prepared according to General Procedure 3 from Fmoc-tetrapeptide-OTrt-resin (**S14**).

Pentapeptide-OTrt-resin (**41**)

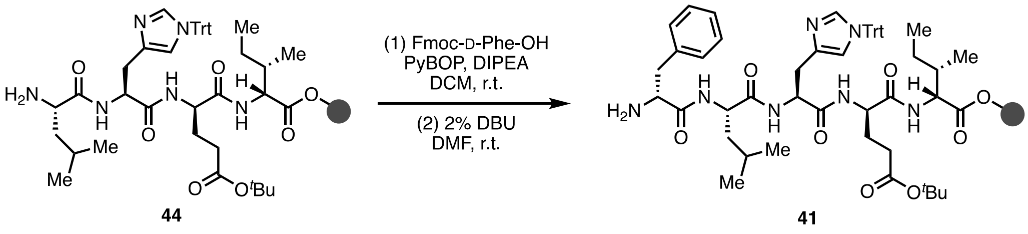

Prepared according to General Procedure 4 using Fmoc-D-Phe-OH (182 mg, 0.471 mmol, 3.0 equiv) as the amino acid, followed by General Procedure 3.

Pentapeptide-OH (**S15**)

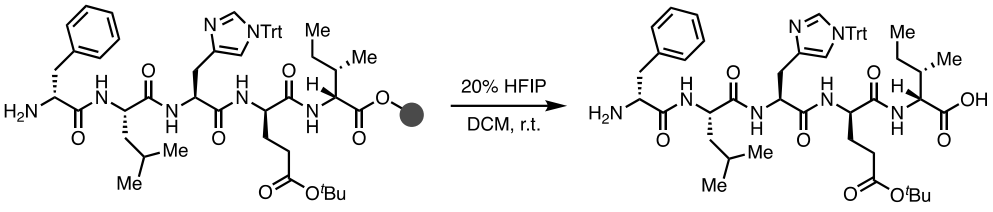

To a polypropylene stopcock-frit tube charged with the resin-supported peptide (10.0 mg) was added DCM (0.200 mL) at room temperature to swell the resin for 30 min prior to the reaction. After removal of DCM, to the tube was added a solution of 20% HFIP in DCM (0.200 mL) at room temperature. After shaking at room temperature for 1 h, the resulting resin was filtered off (washed with excess DCM) and the filtrate was concentrated *in vacuo* and dried under high vacuum, yielding carboxylic acid **S15** (4.5 mg, 4.71 μmol) as a white amorphous solid. *This experiment was conducted to calculate the amount of the corresponding resin-supported pentapeptide for the next reaction.

TCbz-pentapeptide-OTrt-resin (**42**)

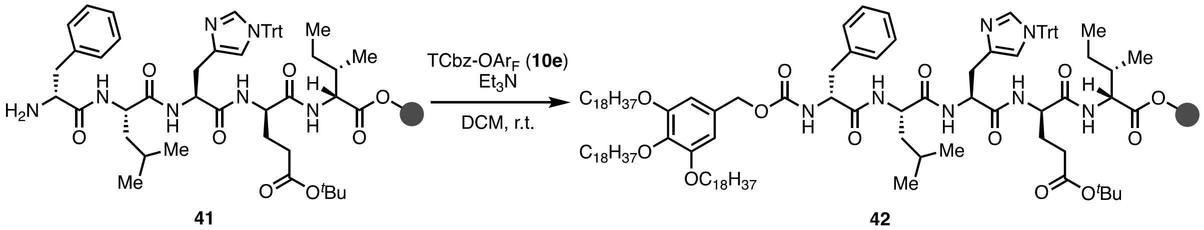

To a polypropylene stopcock-frit tube charged with the resin-supported peptide **41** (10.0 mg, ca. 4.71 μmol) was added DCM (1.00 mL, calculated as 0.16 M to **10e**) at room temperature to swell the resin for 30 min prior to the reaction. After removal of DCM, to the tube was added a solution of TCbz-OAr$_F$ (**10e**) (176 mg, 0.157 mmol, 33 equiv) in DCM (1.00 mL, calculated as 0.16 M to **10e**) and Et_3N (34.8 μL, 0.250 mmol, 53 equiv) at room temperature. After shaking at room temperature for 48 h, the resulting solution was removed by suction filtration through the stopcock. The residual resin on the frit was washed with DCM (1.00 mL, shaking for 1 min) twice, DMF (1.00 mL, shaking for 1 min) twice and DCM (1.00 mL, shaking for 1 min) twice. After removal of the washing solvents, the residual resin was used in the next reaction without further purification.

Tetrapeptide-OH (**S16**)

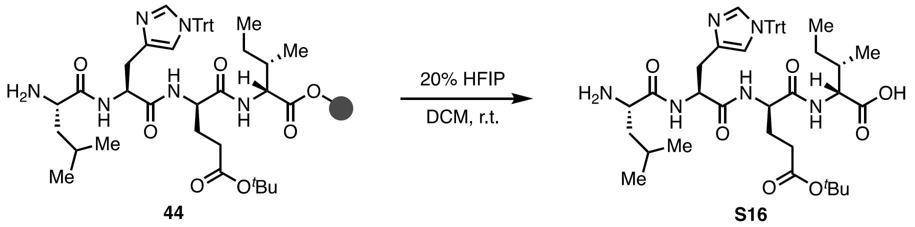

To a polypropylene stopcock-frit tube charged with the resin-supported peptide (12.1 mg) was added DCM (0.200 mL) at room temperature to swell the resin for 30 min prior to the reaction. After removal of DCM, to the tube was added a solution of 20% HFIP in DCM (0.200 mL) at room temperature. After shaking at room temperature for 1 h, the resulting resin was filtered off (washed with excess DCM) and the filtrate was concentrated *in vacuo* and dried under high vacuum, yielding carboxylic acid **S16** (4.1 mg, 5.07 μmol) as a white amorphous solid. *This experiment was conducted to calculate the amount of the corresponding resin-supported pentapeptide for the next reaction.

TCbz-pentapeptide-OTrt-resin (**42**)

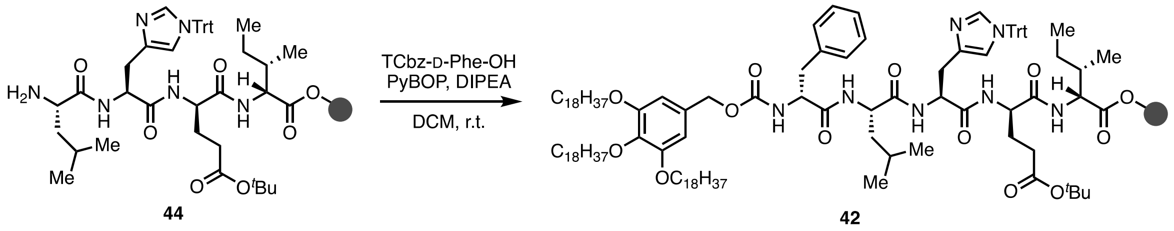

To a polypropylene stopcock-frit tube charged with the resin-supported peptide (12.1 mg, ca. 5.07 μmol) was added DCM (0.200 mL, 0.025 M) at room temperature to swell the resin for 30 min prior to the reaction. After removal of DCM, to the tube was added a solution of TCbz-D-Phe-OH (52.0 mg, 47.1 μmol, 9.3 equiv) in DCM (0.507 mL, 0.01 M) at room temperature. To the resulting mixture was added PyBOP (27.0 mg, 51.8 μmol, 10.2 equiv) and DIPEA (16.4 μL, 94.2 μmol, 18.6 equiv) at room temperature. After shaking at room temperature for 24 h, the resulting solution was removed by suction filtration through the stopcock. The residual resin on the frit was washed with DCM (1.00 mL, shaking for 1 min) twice, DMF (1.00 mL, shaking for 1 min) twice and DCM (1.00 mL, shaking for 1 min) twice. After removal of the washing solvents, the residual resin was used in the next reaction without further purification.

TCbz-pentapeptide-OH (**43**)

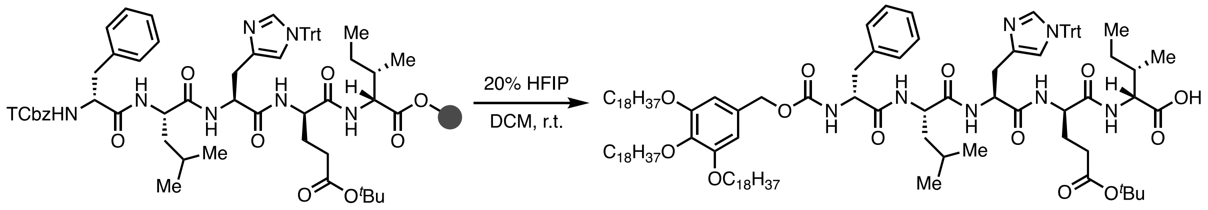

42 **43**

To the tube containing the residual resin was added 20% HFIP in DMF (0.507 mL, 0.01 M) at room temperature. After shaking at room temperature for 1 h, the resulting resin was filtered off (washed with excess DCM) and the filtrate was concentrated *in vacuo*. The resulting residue was dissolved in DCM (0.100 mL, 0.05 M) and cooled down to 0 °C. The resulting solution was poured into cold MeOH (0.500 mL, 0.01 M). The resulting suspension was stirred for 30 min at 0 °C and filtered through a pad of Celite (washed with excess MeOH). The product collected on the filter cake was dissolved in $CHCl_3$ and then eluted by suction filtration using excess $CHCl_3$. The resulting filtrate was concentrated *in vacuo* and dried under high vacuum, yielding **43** (9.6 mg, 5.06 µmol, >99%) as a white powder.

Rf-value: 0.45 ($CHCl_3$/MeOH = 10:1, stained with phosphomolybdic acid)

HRMS (*m/z*): FAB $[M+Na]^+$ calculated for $C_{117}H_{183}N_7O_{13}Na$: 1918.3805, found: 1918.3763.

$[\alpha]_D^{22}$ = +6.08 (c = 0.1, $CHCl_3$)

^{1}H NMR (500 MHz, $CDCl_3$: δ 7.76 – 7.56 (br-m, 1H), 7.31 (s, 10H), 7.23 – 7.10 (m, 6H), 7.10 – 7.00 (m, 7H), 6.77 (br-s, 1H), 6.47 (s, 2H), 5.01 – 4.94 (m, 1H), 4.76 – 4.62 (m, 2H), 4.57 – 4.35 (m, 3H), 4.32 – 4.14 (m, 1H), 4.02 – 3.72 (m, 2H), 3.91 (t, *J* = 6.6 Hz, 6H), 3.24 – 3.10 (m, 1H), 3.08 – 2.88 (m, 3H), 2.40 – 2.25 (m, 2H), 2.13 – 2.01 (m, 1H), 2.01 – 1.89 (m, 2H), 1.81 – 1.59 (m, 10H), 1.51 – 1.41 (m, 8H), 1.38 (s, 9H), 1.36 – 1.17 (m, 84H), 0.87 (app t, *J* = 6.8 Hz, 15H), 0.73 (app br-s, 6H).

^{13}C NMR (126 MHz, $CDCl_3$): δ 173.1, 172.8, 171.7, 170.8, 167.7, 156.2 (3C), 153.3, 141.3 (3C), 138.2, 136.6, 131.2, 129.8 (6C), 129.4 (3C), 128.6 (5C), 128.5 (6C), 128.1, 126.9, 120.6, 107.0 (2C), 80.8, 73.5 (2C), 69.2 (2C), 67.6, 65.7, 57.4, 54.0, 53.6, 52.6, 49.7, 40.8, 38.7, 37.3, 32.1 (3C), 31.1, 30.5, 30.0 – 29.7 (overlap, 29C), 29.6 – 19.4 (overlap, 8C), 28.22, 28.18 (3C), 26.3 (3C), 25.8, 24.5, 23.5, 23.0, 22.8 (3C), 21.8, 15.1, 14.2 (3C), 11.9.

TCbz-hexapeptide (**46**)

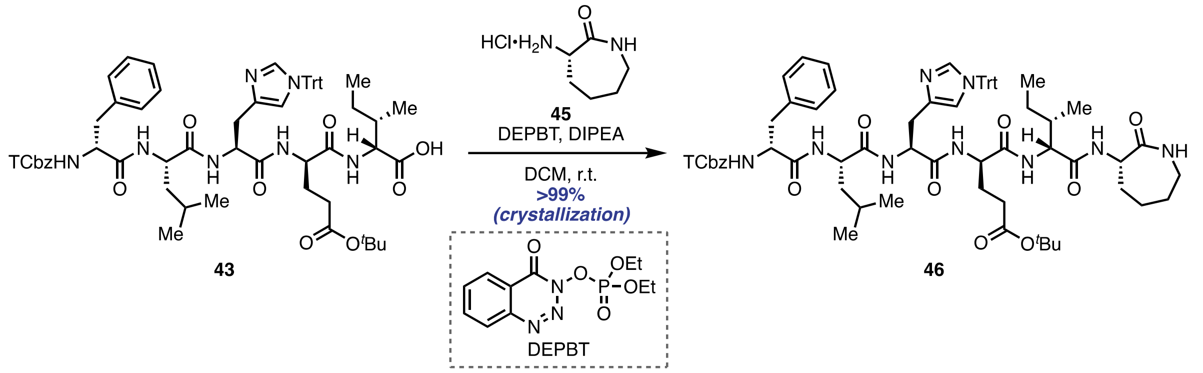

To a solution of TCbz-pentapeptide-OH (**43**) (13.0 mg, 6.86 µmol) and (–)-α-amino-ε-caprolactam (**45**) (2.3 mg, 13.7 µmol, 2.0 equiv) in DCM (0.137 mL, 0.05 M) was added DEPBT (5.1 mg, 17.1 µmol, 2.5 equiv) and DIPEA (4.2 µL, 24.0 µmol, 3.5 equiv) at room temperature under air. After stirring at room temperature for 3 h, the reaction mixture was cooled down to 0 °C and poured into cold MeOH (0.686 mL, 0.01 M). The resulting suspension was stirred for 30 min at 0 ºC and filtered through a pad of Celite (washed with excess MeOH). The product collected on the filter cake was dissolved in $CHCl_3$ and then eluted by suction filtration using excess $CHCl_3$. The resulting filtrate was concentrated *in vacuo* and dried under high vacuum, yielding **46** (13.8 mg, 6.87 µmol, >99%) as a white powder.

Rf-value: 0.50 ($CHCl_3$/MeOH = 10:1, stained with phosphomolybdic acid)

HRMS (*m/z*): FAB $[M+Na]^+$ calculated for $C_{123}H_{193}N_9O_{13}Na$: 2027.4616, found: 2027.4636.

$[\alpha]_D^{22}$ = +5.44 (c = 0.1, $CHCl_3$)

^{1}H NMR (500 MHz, $CDCl_3$: δ 7.80 (br-s, 2H), 7.39 – 7.28 (m, 12H), 7.25 – 7.14 (m, 6H), 7.14 – 7.04 (m, 8H), 6.63 (s, 1H), 6.45 (s, 2H), 6.14 (br-s, 1H), 5.01 – 4.84 (m, 1H), 4.80 – 4.56 (m, 1H), 4.67 – 4.25 (m, 5H), 3.99 – 3.83 (m, 6H), 3.68 – 3.63 (m, 1H), 3.45 – 3.38 (m, 1H), 3.19 – 3.03 (m, 2H), 3.02 – 2.72 (m, 3H), 2.43 – 2.29 (m, 2H), 2.16 – 2.06 (m, 1H), 2.06 – 1.89 (m, 3H), 1.86 (br-s, 3H), 1.82 – 1.65 (s, 8H), 1.65 – 1.49 (m, 3H), 1.49 – 1.37 (m, 16H), 1.36 – 1.07 (m, 84H), 0.88 (app t, *J* = 7.0 Hz, 15H), 0.86 – 0.75 (m, 6H).

^{13}C NMR (126 MHz, $CDCl_3$): δ 175.6, 173.0, 172.2, 171.7, 171.3, 170.8, 156.3, 153.3 (3C), 142.4 (3C), 138.6, 138.1, 137.0, 136.9, 131.0, 129.8 (6C), 129.4 (2C), 128.6 (2C), 128.2 (9C), 126.8, 119.4, 106.9 (2C), 80.9, 75.4 (2C), 73.5 (2C), 69.2, 67.6, 56.9, 56.7, 54.7, 53.9, 52.2, 51.9, 41.9, 41.6, 38.5, 36.9, 32.0 (3C), 31.9, 31.0, 30.5, 30.0 – 29.7 (overlap, 29C), 29.6 – 29.4 (overlap, 8C), 28.9, 28.1 (3C), 28.0, 27.5, 26.4, 26.3 (3C), 24.8, 24.7, 23.3, 22.8 (3C), 21.9, 14.7, 14.2 (3C), 11.7.

Calpinactam (**39**)

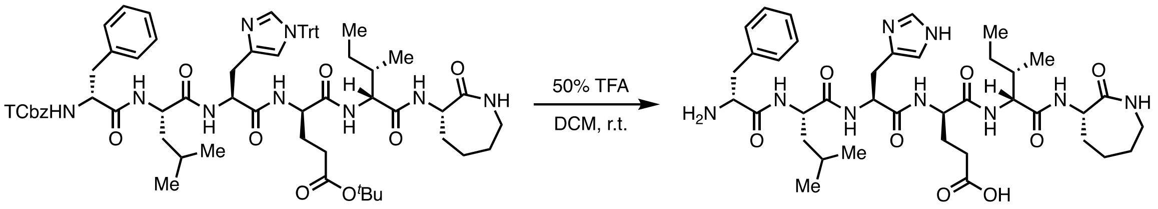

To a glass vessel charged with hexapeptide **46** (25.0 mg, 12.5 µmol) was added DCM/TFA (1:1 v/v, 0.250 mL, 0.05 M) at room temperature under air. After stirring at room temperature for 2 h, to the reaction mixture was Et_2O (12.5 mL, 0.001 M) at room temperature. After stirring at room temperature for 30 min, the magnetic stir bar was removed, and the suspension was centrifuged (4000 rpm) for 1 min. The resulting solution was removed by decantation and to the residue was added Et_2O (12.5 mL, 0.001 M) at room temperature. After repeating this centrifugation and decantation protocol for three times, the resulting solid was dried under high vacuum, yielding calpinactam (**39**) (7.6 mg, 9.90 µmol, 79%) as a white solid.

**Note: The spectra data of the natural calpinactam were reported as a TFA salt. We found that NMR spectra of the synthetic material matched with those reported in the literature by adding 1 equiv of TFA. ^{1}H and ^{13}C NMR spectra provided here are a TFA salt of calpinactam. Because calpinactam is fragile in the presence of acids, it is crucial to remove acid-containing solvents by a freeze-pump drying operation.*

Rf-value: 0.20–0.35, tailing (MeOH only, stained with ninhydrin)

HRMS (*m/z*): ESI $[M+H]^+$ calculated for $C_{38}H_{58}N_9O_8$: 768.4408, found: 768.4406.

$[\alpha]_D^{23}$ = –30.3 (c = 0.05, AcOH); [lit[10], $[\alpha]_D^{25}$ = –26.0 (c = 0.05, AcOH)]

^{1}H NMR (400 MHz, $(CD_3)_2SO$) *two protons of the imidazole ring not detected: δ 8.59 (d, *J* = 7.9 Hz, 1H), 8.43 (d, *J* = 7.3 Hz, 1H), 7.98 (d, *J* = 8.4 Hz, 1H), 7.92 (d, *J* = 7.5 Hz, 1H), 7.84 (app t, *J* = 7.0 Hz, 1H), 7.80 (d, *J* = 6.7 Hz, 1H), 7.35 – 7.29 (m, 2H), 7.29 – 7.22 (m, 3H), 7.16 (br-s, 1H), 4.57 (dt, *J* = 8.4, 7.3 Hz, 1H), 4.44 – 4.35 (m, 2H), 4.32 (dd, *J* = 8.4, 6.5 Hz, 1H), 4.27 (dt, *J* = 7.9, 7.5 Hz 1H), 4.08 (t, *J* = 7.0 Hz, 1H), 3.22 – 3.12 (m, 1H), 3.10 – 2.99 (m, 3H), 2.96 (dd, *J* = 14.5, 7.0 Hz, 1H), 2.84 (dd, *J* = 14.7, 8.4 Hz, 1H), 2.09 (t, *J* = 7.6 Hz, 2H), 1.94 – 1.77 (m, 3H), 1.77 – 1.68 (m, 3H), 1.68 – 1.55 (m, 1H), 1.40 – 1.31 (m, 1H), 1.31 – 1.25 (m, 3H), 1.25 – 1.11 (m, 2H), 1.11 – 1.00 (m, 1H), 0.82 (t, *J* = 7.2 Hz, 3H), 0.78 (d, *J* = 6.6 Hz, 3H), 0.76 (d, *J* = 6.6 Hz, 3H), 0.73 (d, *J* = 6.2 Hz, 3H).

*As a result of investigation, we obtained ^{1}H NMR spectra detecting the missing imidazole protons in the isolation paper which are also provided herewith.

^{1}H NMR (500 MHz, $(CD_3)_2SO$): 8.97 (d, *J* = 1.1 Hz, 1H), 8.63 (d, *J* = 7.9 Hz, 1H), 8.52 (d, *J* = 8.2 Hz,

1H), 8.19 (br-s, 3H), 8.01 (d, *J* = 8.9 Hz, 1H), 7.98 (d, *J* = 8.2 Hz, 1H), 7.86 (dd, *J* = 7.0, 5.0 Hz, 1H), 7.82 (d, *J* = 6.7 Hz, 1H), 7.34 (s, 1H), 7.34 – 7.28 (m, 2H), 7.28 – 7.23 (m, 3H), 4.61 (dt, *J* = 8.2, 7.6 Hz, 1H), 4.42 – 4.36 (m, 2H), 4.32 (dd, *J* = 8.2, 5.7 Hz, 1H), 4.23 (app q, *J* = 7.9 Hz, 2H), 4.09 (br-s, 1H), 3.21 – 3.13 (m, 1H), 3.13 – 3.00 (m, 3H), 2.97 (dd, *J* = 13.8, 7.7 Hz, 1H), 2.90 (dd, *J* = 15.3, 7.6 Hz, 1H), 2.12 – 2.07 (m, 2H), 1.92 – 1.77 (m, 3H), 1.76 – 1.56 (m, 4H), 1.40 – 1.32 (m, 1H), 1.32 – 1.23 (m, 3H), 1.23 – 1.12 (m, 2H), 1.09 – 1.02 (m, 1H), 0.81 (t, *J* = 7.4 Hz, 3H), 0.78 (d, *J* = 7.0 Hz, 3H), 0.76 (d, *J* = 6.4 Hz, 3H), 0.72 (d, *J* = 6.4 Hz, 3H).

^{13}C NMR (126 MHz, $(CD_3)_2SO$): δ 174.1, 173.9, 171.7, 171.1, 169.8, 169.6, 167.9, 134.8, 133.8, 129.5, 129.2, 128.5, 127.2, 117.3, 56.1, 53.4, 51.64, 51.60, 51.4, 51.2, 40.71, 40.65, 37.2, 36.6, 31.2, 30.0, 28.8, 27.8, 27.7, 27.0, 25.7, 23.8, 23.0, 21.3, 14.4, 11.6.

• **HPLC data** (Purif-Rp2, column: Senshu Pak PEGASIL ODS 20 φ × 250 mm, flow rate: 8 mL/min, solvent condition: 5% MeCN (10 min, isocratic) – 40% (30 min, gradient) – 100% (10 min, gradient then 10 min, isocratic) / H_2O with 0.05% TFA

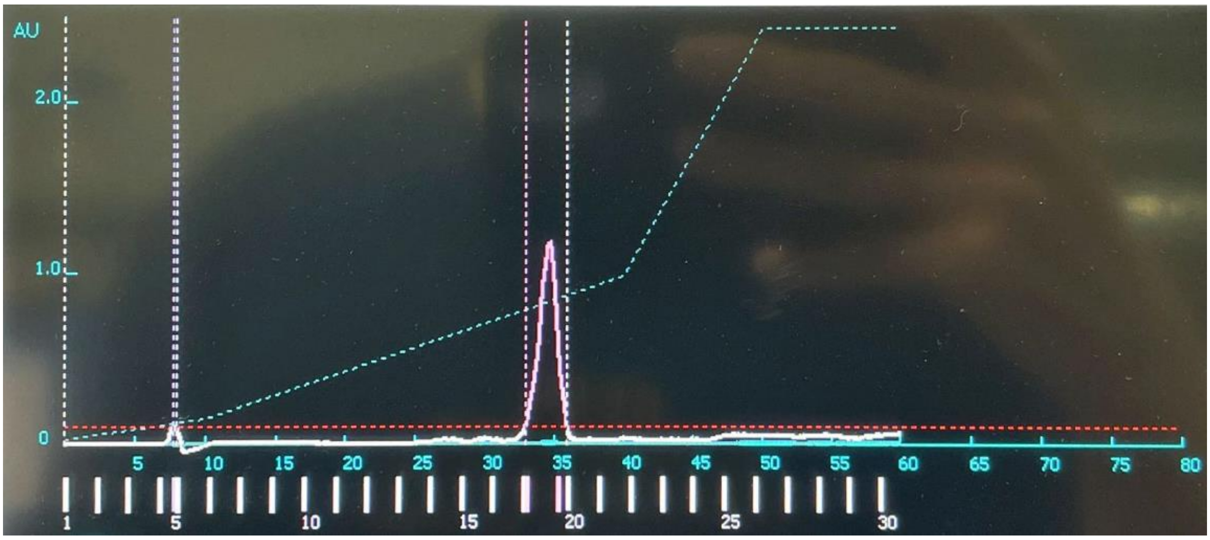

• Spectral charts comparison (in $(CD_3)_2SO$) on investigation of purification procedures

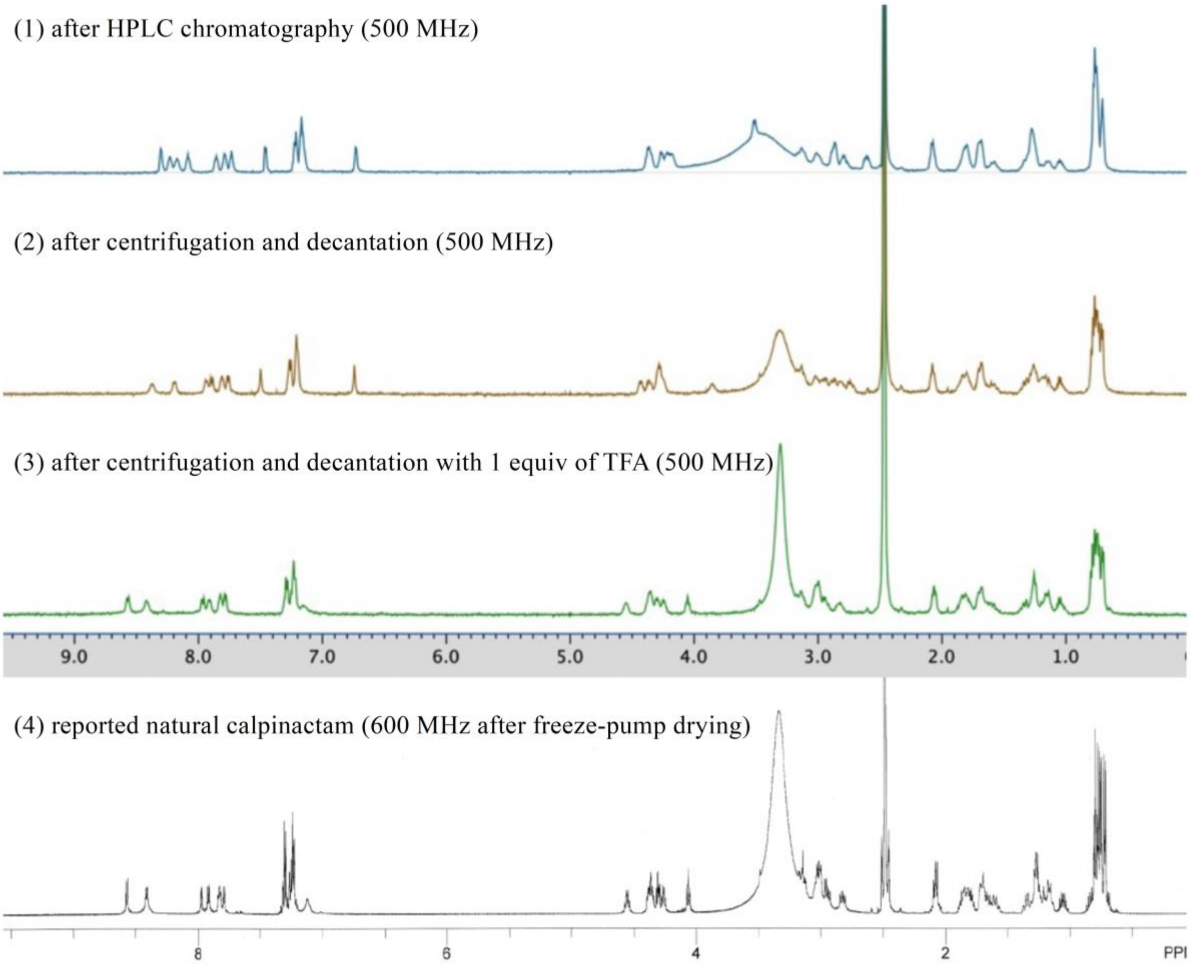

• **LC-UV and MS analysis for investigation of epimerization**

(JEOL JMS-T100LP, column: CAPCELL CORE C18 2.1 φ × 250 mm, flow rate: 0.4 mL/min, solvent condition: 10% – 100 MeCN (12 min, gradient) / H_2O with 0.1% HCO_2H)

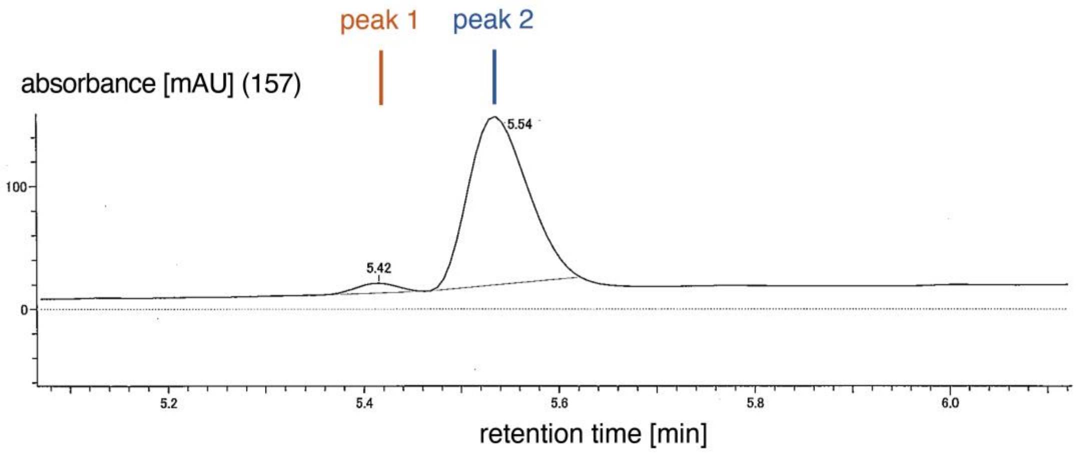

peak	retention time [min]	type	half width [min]	area [intens.*sec]	height	start point time [min]/height	end point time [min]/height
1	5.42	BB	0.0488	24.34	8.20	5.36/12	5.46/14
2	5.54	BB	0.0684	577.94	137.16	5.47/15	5.62/26

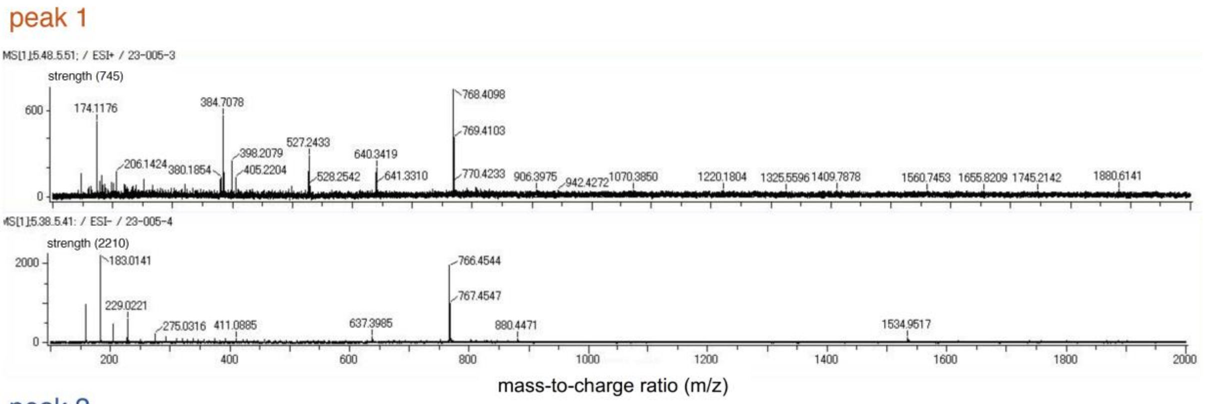

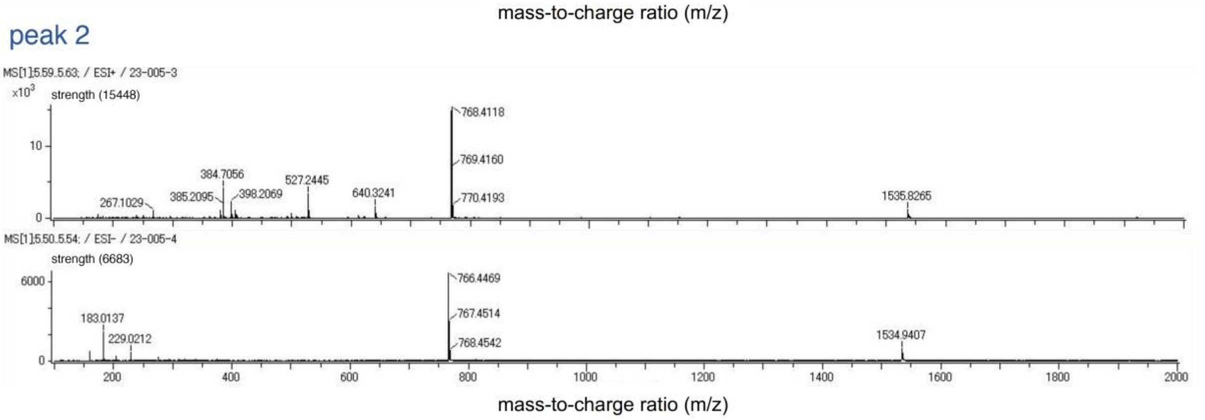

4. Spectral Data Comparison of Natural and Synthetic Calpinactam

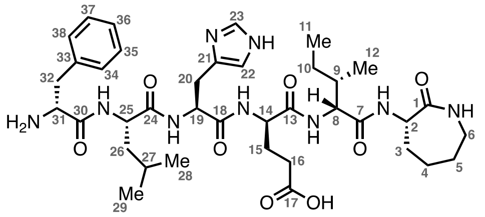

calpinactam (**39**)

• **^{1}H NMR**

Position	Natural[10] (600 MHz, $CDCl_3$) δ_H	**This work (400 MHz, $CDCl_3$) δ_H**
2	4.36 (m)	**4.44 – 4.35 (overlap, m, 2H)**
2-NH	7.79 (d, *J* = 6.5 Hz)	**7.80 (d, *J* = 6.7 Hz)**
3	1.70 (m)<br>1.34 (m)	**1.77 – 1.68 (overlap, m, 3H)**<br>**1.40 – 1.31 (m)**
4	1.84 (m)<br>1.60 (m)	**1.94 – 1.77 (overlap, m, 3H)**<br>**1.68 – 1.55 (m, 1H)**
5	1.71 (m)<br>1.17 (m)	**1.77 – 1.68 (overlap, m, 3H)**<br>**1.25 – 1.11 (overlap, m, 2H)**
6	3.14 (m)<br>3.02 (m)	**3.22 – 3.12 (m)**<br>**3.10 – 2.99 (overlap, m, 3H)**
6-NH	7.83 (dd, *J* = 7.0, 5.0 Hz)	**7.84 (app t, *J* = 7.0 Hz)**
8	4.31 (dd, *J* = 9.0, 6.0 Hz)	**4.32 (dd, *J* = 8.4, 6.5 Hz)**
8-NH	7.97 (d, *J* = 9.0 Hz)	**7.98 (d, *J* = 8.4 Hz)**
9	1.80 (m)	**1.94 – 1.77 (overlap, m, 3H)**
10	1.27 (m)<br>1.05 (m)	**1.31 – 1.25 (overlap, m, 3H)**<br>**1.11 – 1.00 (m)**
11	0.80 (d, *J* = 7.0 Hz, 3H)	**0.82 (t, *J* = 7.2 Hz, 3H)**

12	0.77 (d, *J* = 6.0 Hz, 3H)	**0.78 (d, *J* = 6.6 Hz, 3H)**
14	4.36 (m)	**4.44 – 4.35 (overlap, m, 2H)**
14-NH	7.92 (d, *J* = 8.0)	**7.92 (d, *J* = 7.5 Hz)**
15	1.85 (m)<br>1.70 (m)	**1.94 – 1.77 (overlap, m, 3H)**<br>**1.77 – 1.68 (overlap, m, 3H)**
16	2.07 (m, 2H)	**2.09 (t, *J* = 7.6 Hz, 2H)**
19	4.55 (dt, *J* = 8.0, 7.5 Hz)	**4.57 (dt, *J* = 8.4, 7.3 Hz)**
19-NH	8.42 (d, *J* = 7.5 Hz)	**8.43 (d, *J* = 7.3 Hz)**
20	3.01 (m)<br>2.82 (dd, *J* = 16.0, 8.0 Hz)	**3.10 – 2.99 (overlap, m, 3H)**<br>**2.84 (dd, *J* = 14.7, 8.4 Hz)**
22	not detected*	not detected
23	not detected*	not detected
25	4.26 (dt, *J* = 8.0, 7.0 Hz)	**4.27 (dt, *J* = 7.9, 7.5 Hz)**
25-NH	8.57 (d, *J* = 8.0 Hz)	**8.59 (d, *J* = 7.9 Hz)**
26	1.26 (m, 2H)	**1.31 – 1.25 (overlap, m, 3H)**
27	1.16 (m)	**1.25 – 1.11 (overlap, m, 2H)**
28	0.75 (d, *J* = 6.0 Hz, 3H)	**0.76 (d, *J* = 6.6 Hz, 3H)**
29	0.72 (d, *J* = 6.0 Hz, 3H)	**0.73 (d, *J* = 6.2 Hz, 3H)**
31	4.06 (t, *J* = 7.0 Hz)	**4.08 (t, *J* = 7.0 Hz)**
32	3.00 (m)<br>2.96 (dd, *J* = 14.0, 7.0 Hz)	**3.10 – 2.99 (overlap, m, 3H)**<br>**2.96 (dd, *J* = 14.5, 7.0 Hz)**
34, 38	7.24 (m, 2H)	**7.29 – 7.22 (overlap, m, 3H)**
35, 37	7.30 (m, 2H)	**7.35 – 7.29 (m, 2H)**
36	7.26 (m)	**7.29 – 7.22 (overlap, m, 3H)**

*The authors described these protons were not detected in DMSO-d_6.

• **^{13}C NMR**

Position	Natural[10] (150 MHz, $CDCl_3$) δ_C	**This work (126 MHz, $CDCl_3$) δ_C**
1	174.1	**174.1**
2	51.4	**51.4**
3	31.2	**31.2**
4	27.6	**27.7**
5	28.8	**28.8**
6	40.7	**40.65**
7	169.8	**169.6**
8	56.2	**56.1**
9	36.6	**36.6**
10	25.7	**25.7**
11	11.6	**11.6**
12	14.5	**14.4**
13	171.1	**171.1**
14	51.6	**51.60**
15	27.6	**27.8**
16	30.1	**30.0**
17	173.9	**173.9**
18	170.0	**169.8**
19	52.2	**51.64**
20	27.7	**27.0**

21	130.5	**129.2**
22	117.2	**117.3**
23	134.2	**133.8**
24	171.5	**171.7**
25	51.2	**51.2**
26	40.8	**40.71**
27	23.8	**23.8**
28	21.4	**21.3**
29	23.1	**23.0**
30	167.9	**167.9**
31	53.5	**53.4**
32	37.3	**37.2**
33	134.9	**134.8**
34, 38	129.5 (2C)	**129.5 (2C)**
35, 37	128.6 (2C)	**128.5 (2C)**
36	127.2	**127.2**

5. References

(1) J. A. Grzyb and R. A. Batey, *Tetrahedron Lett.*, 2003, **44**, 7485–7488.

(2) J. Hassfeld, U. Eggert and M. Kalesse, *Synthesis*, 2005, **7**, 1183–1199.

(3) J. J. Fleming and J. Du Bois, *J. Am. Chem. Soc.*, 2006, **128**, 3926–3927.

(4) P. Singh and G. Panda, *RSC Adv.*, 2014, **4**, 2161–2166.

(5) R. S. Giri, S. Roy, G. Dolai, S. R. Manne and B. Mandal, *ChemistrySelect*, 2020, **5**, 2050–2056.

(6) U. Jacquemard, V. Bénéteau, M. Lefoix, S. Routier, J.-Y. Mérour and G. Coudert, *Tetrahedron*, 2004, **60**, 10039–10047.

(7) Y. Noguchi, S. Sekikawa, Y. Nogaki, Y. Satake, N. Murashima, T. Kirisawa, G. Schiffer, J. Koebberling, T. Hirose and T. Sunazuka, *Tetrahedron*, 2022, **128**, 133100.

(8) Y. Hayashi, W. Fukasawa, T. Hirose, M. Iwatsuki, R. Hokari, A. Ishiyama, M. Kanaida, K. Nonaka, A. Take, K. Otoguro, S. Ōmura, K. Shiomi and T. Sunazuka, *Org. Lett.*, 2019, **21**, 2180–2184.

(9) T. Yamada, M. Yagita, Y. Kobayashi, G. Sennari, H. Shimamura, H. Matsui, Y. Horimatsu, H. Hanaki, T. Hirose, S. Ōmura and T. Sunazuka, *J. Org. Chem.*, 2018, **83**, 7135–7149.

(10) N. Koyama, S. Kojima, T. Fukuda, T. Nagamitsu, T. Yasuhara, S. Ōmura and H. Tomoda, *Org. Lett.*, 2010, **12**, 432.

6. NMR Spectra Charts

- TCbz-imidazole (**10b**): ^{1}H NMR ($CDCl_3$)

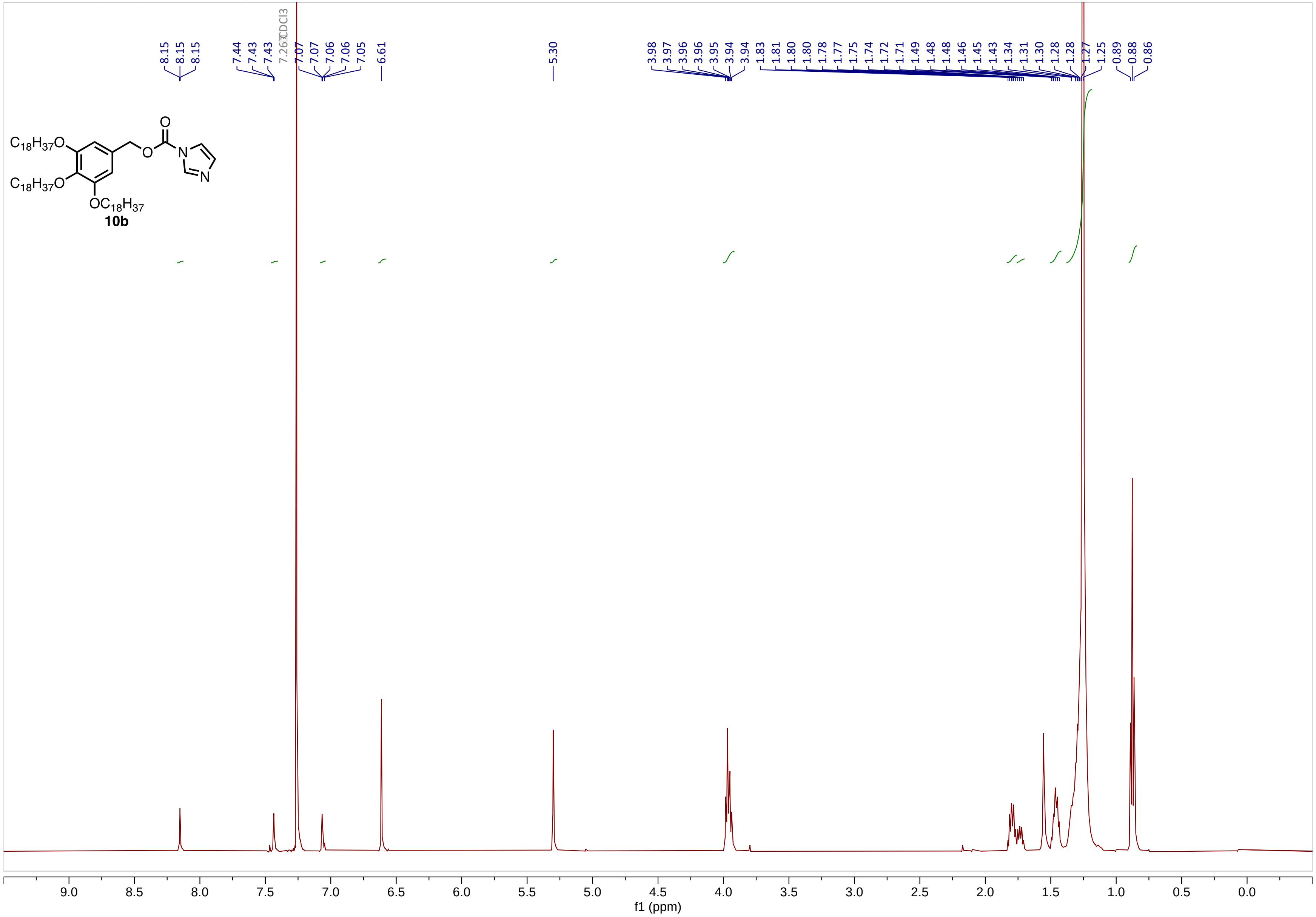

• TCbz-imidazole (**10b**): ^{13}C NMR ($CDCl_3$)

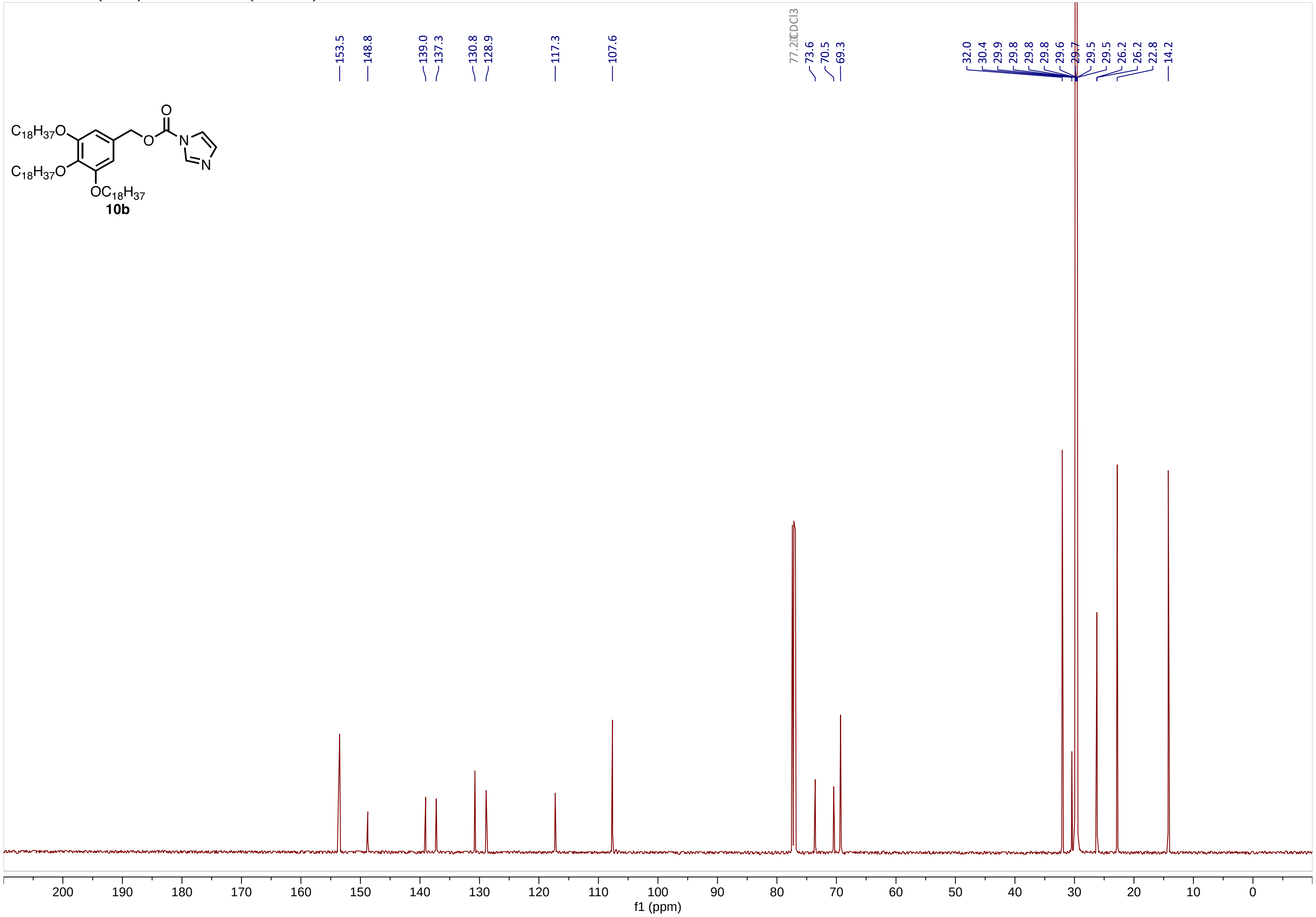

- TCbz-OAr$_F$ (**10e**): ^{1}H NMR ($CDCl_3$)

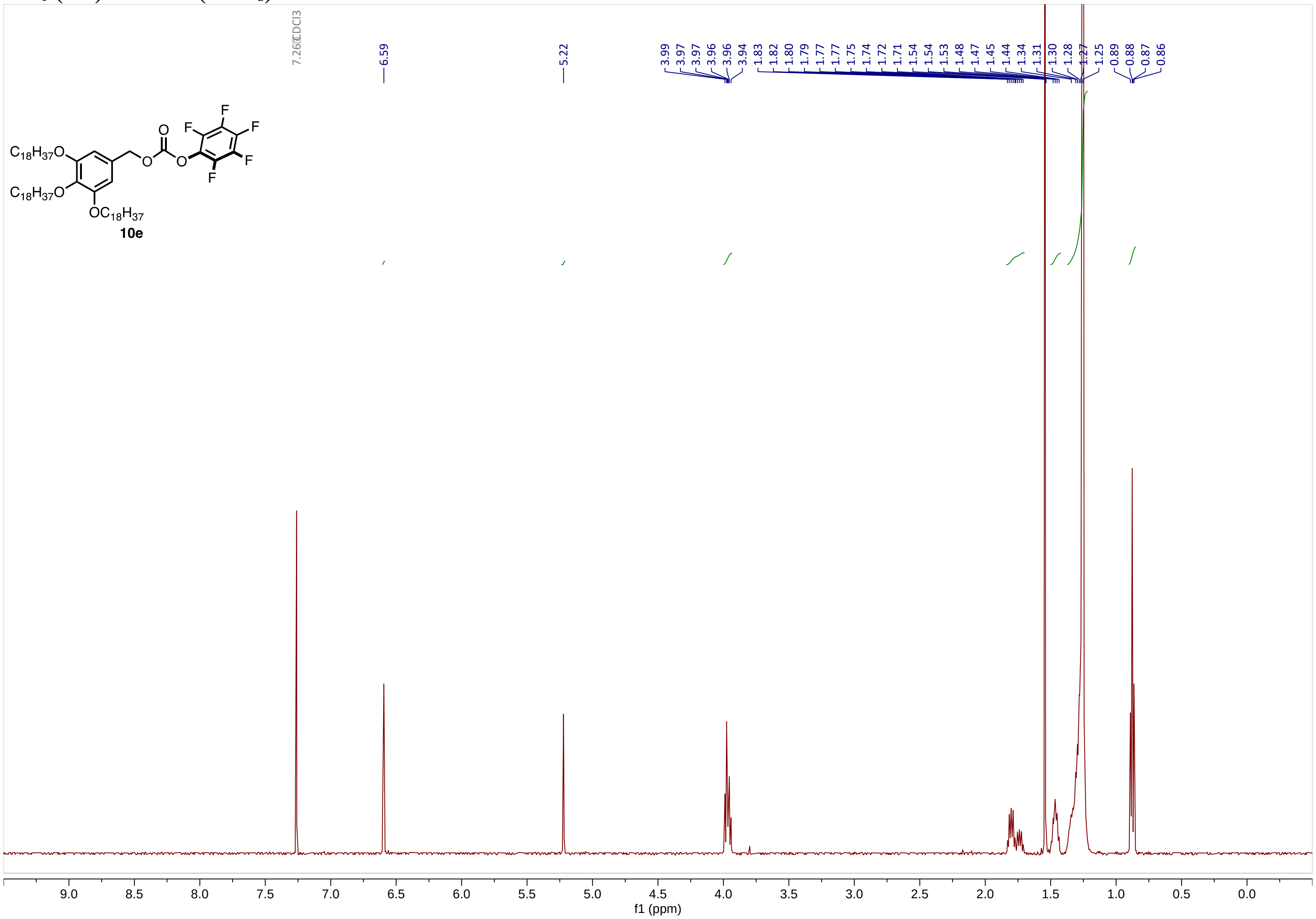

- TCbz-OAr$_F$ (**10e**): ^{13}C NMR ($CDCl_3$)

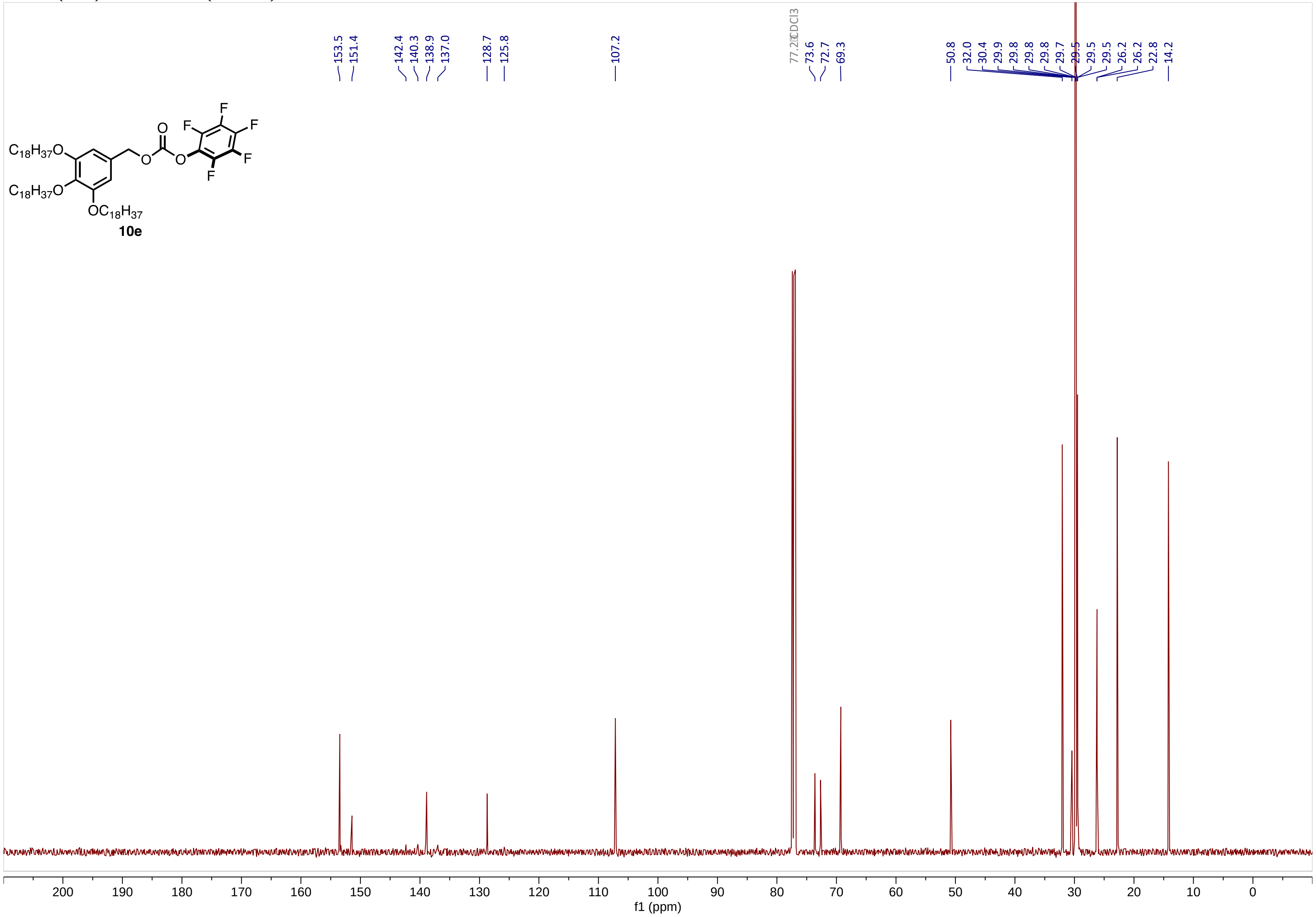

• TCbz-Phe-OMe (**12**): ^{1}H NMR ($CDCl_3$)

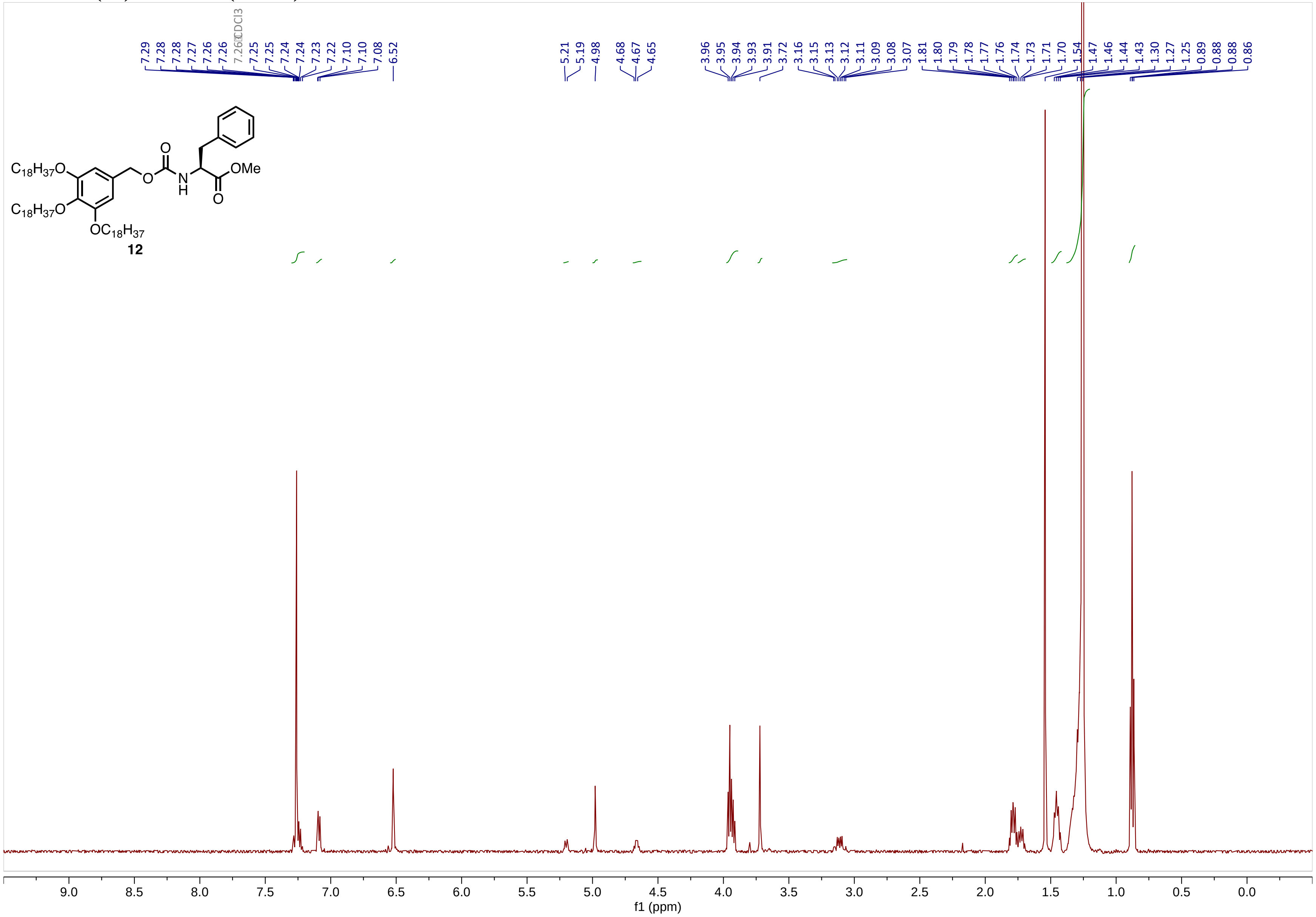

• TCbz-Phe-OMe (**12**): ^{13}C NMR ($CDCl_3$)

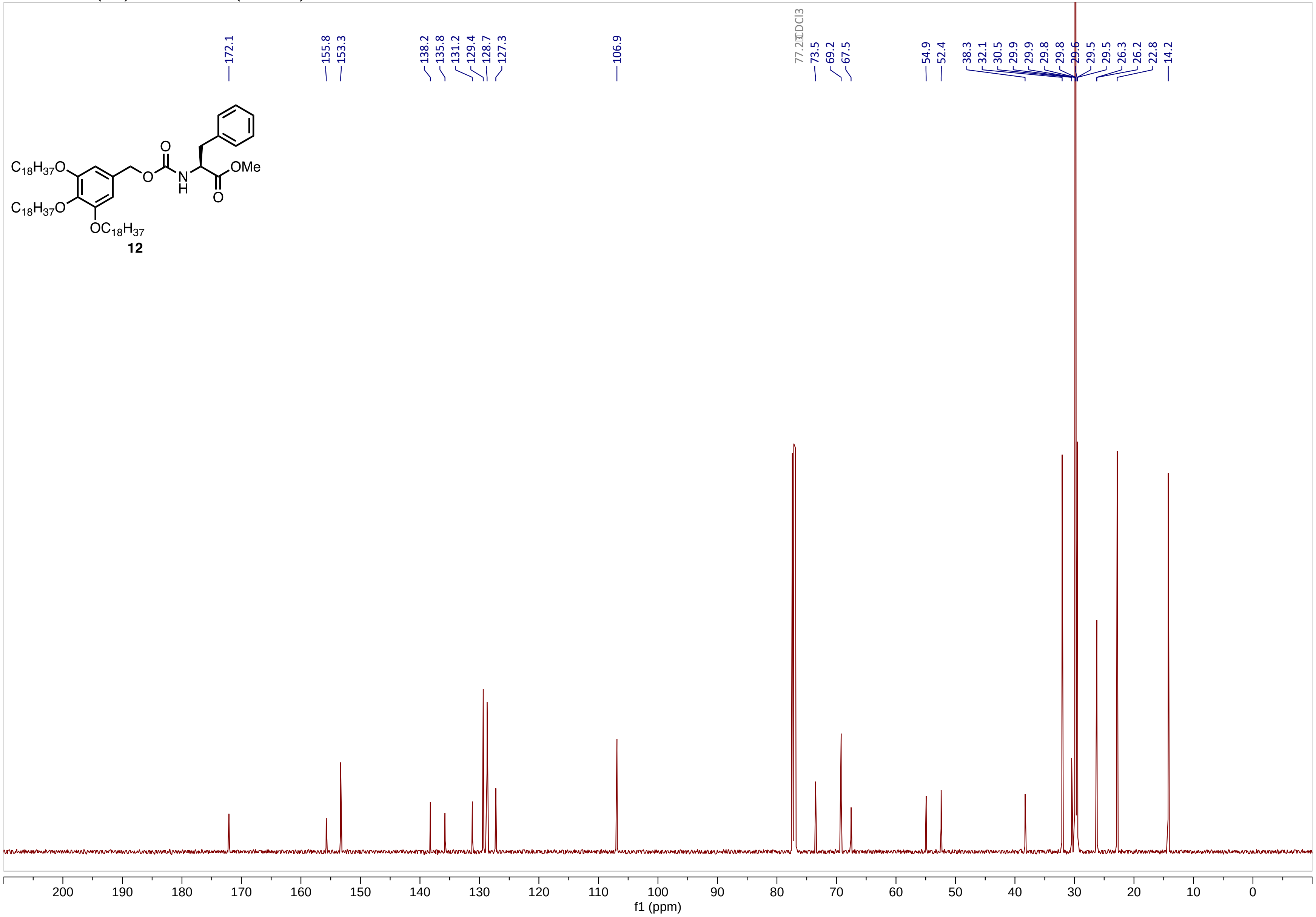

• TCbz-Tyr-OMe (**13**): ^{1}H NMR ($CDCl_3$)

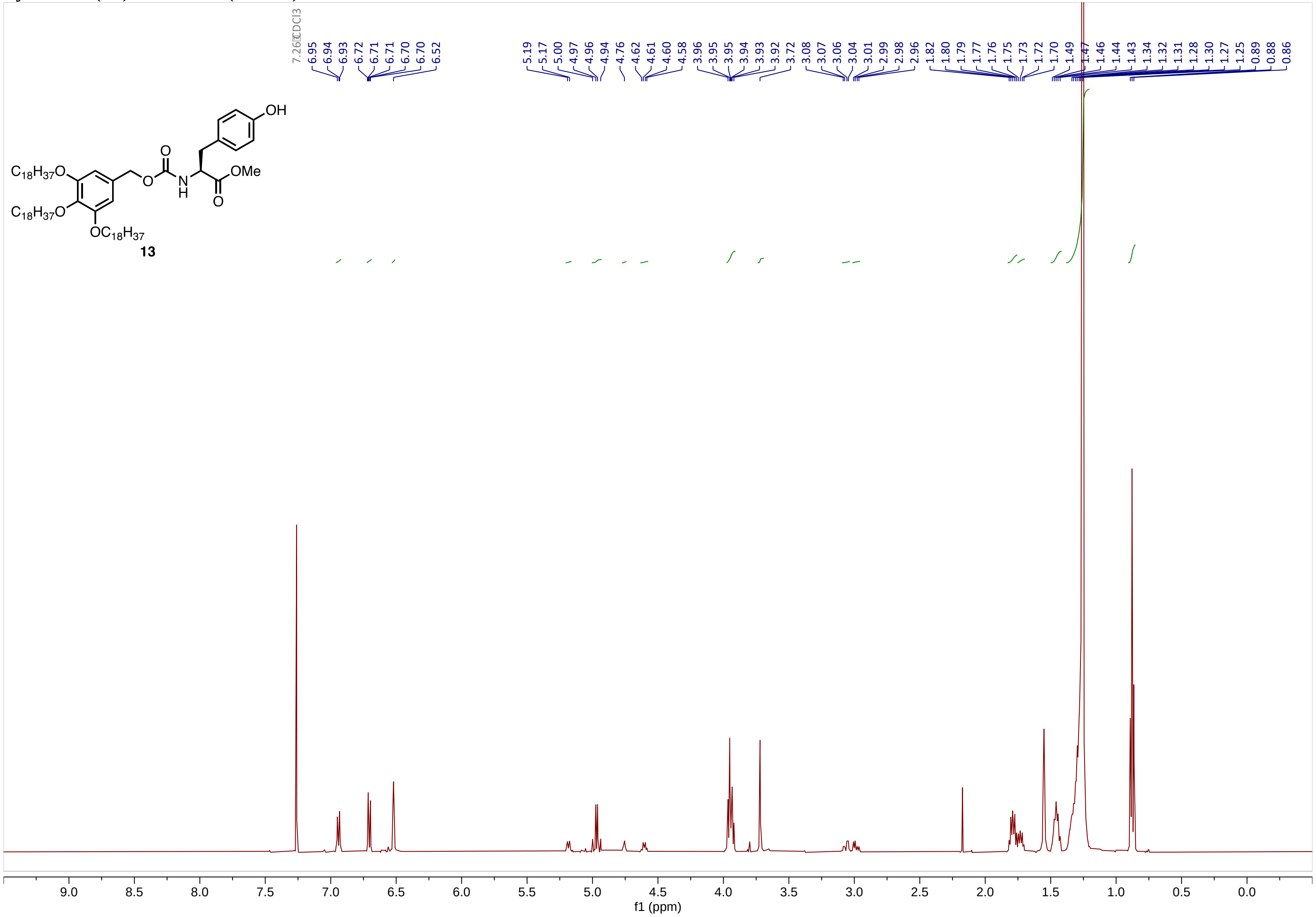

13

• TCbz-Tyr-OMe (**13**): ^{13}C NMR ($CDCl_3$)

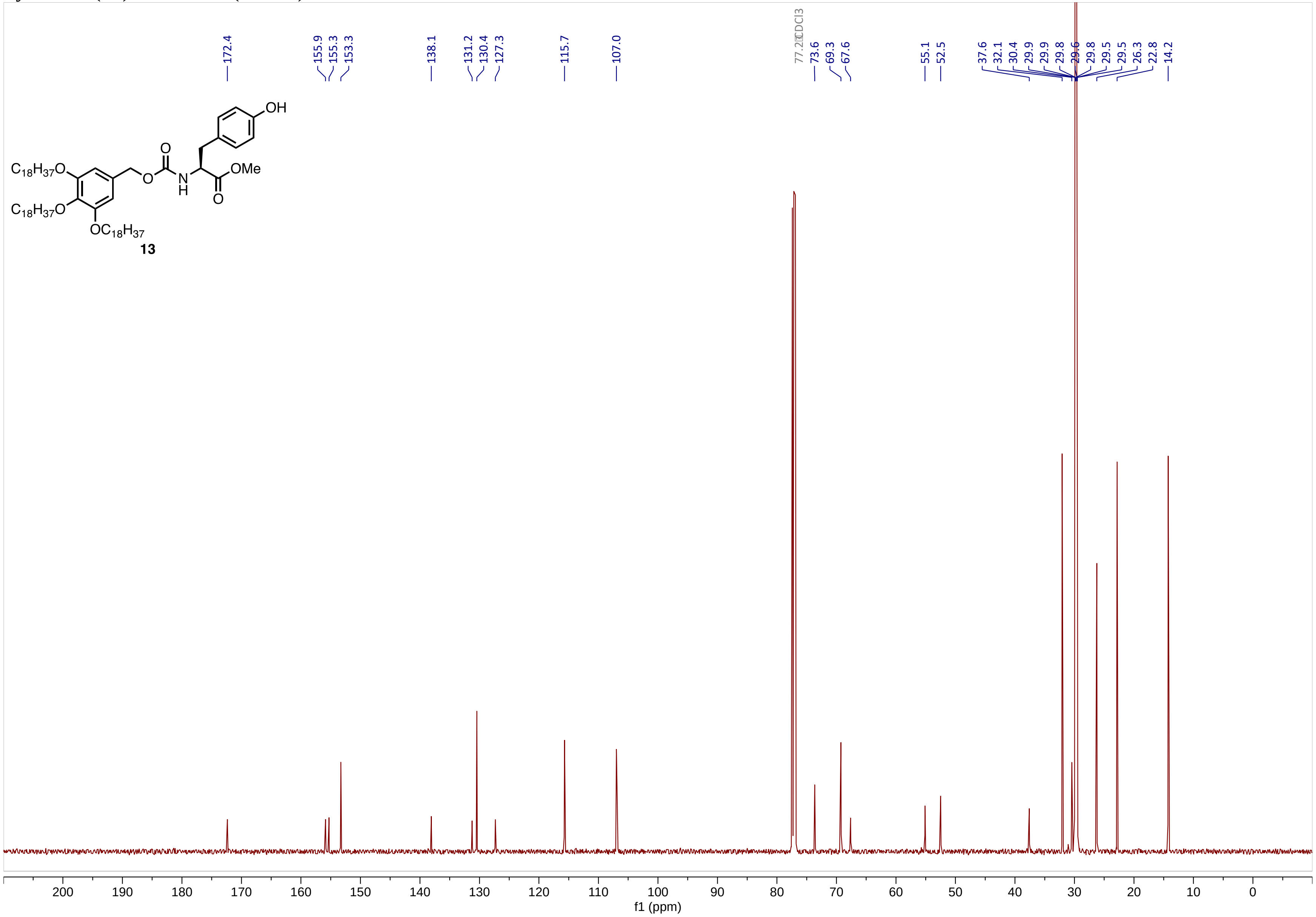

- TCbz-Trp-OBn (**14**): ^{1}H NMR ($CDCl_3$)

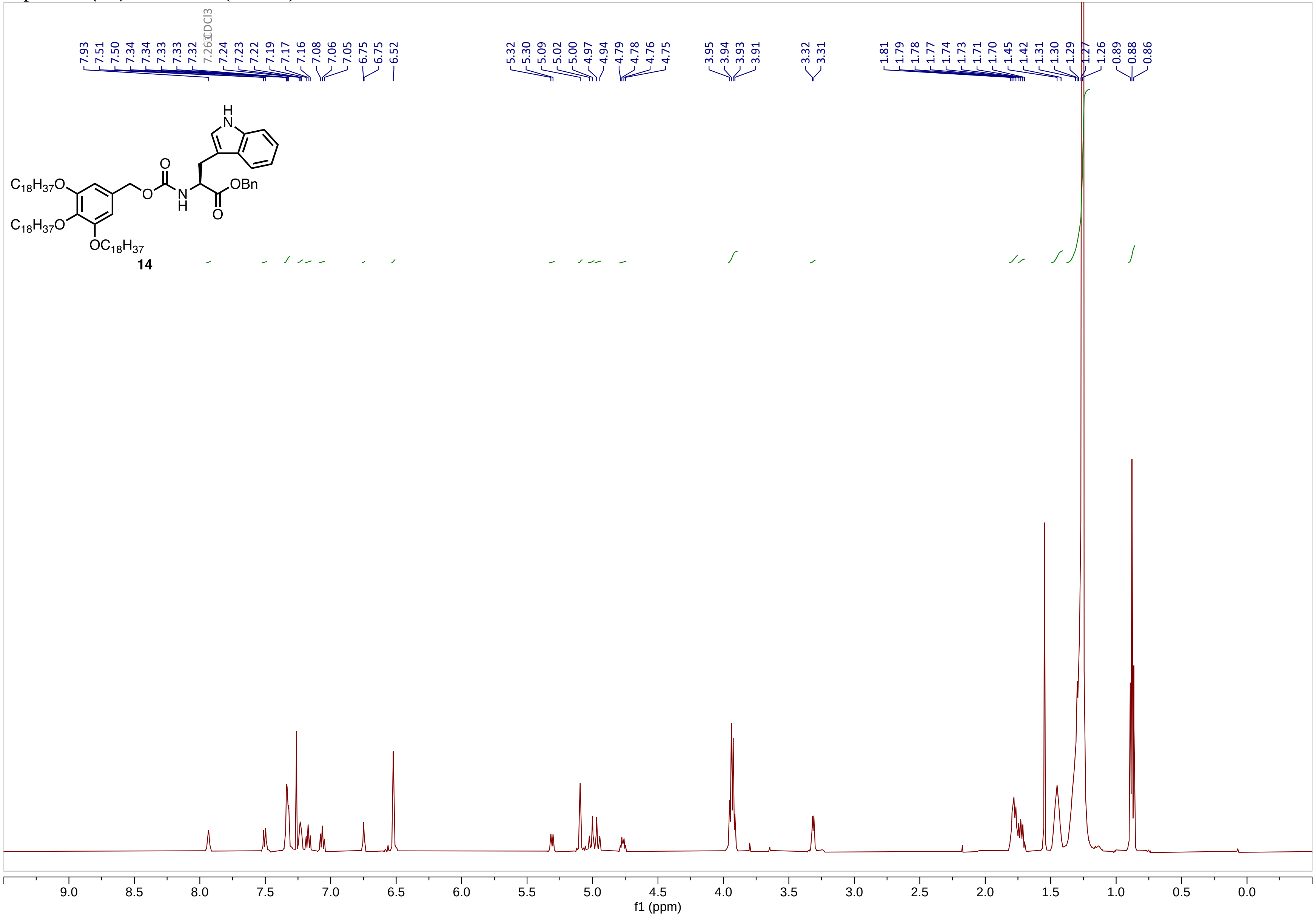

• TCbz-Trp-OBn (**14**): ^{13}C NMR ($CDCl_3$)

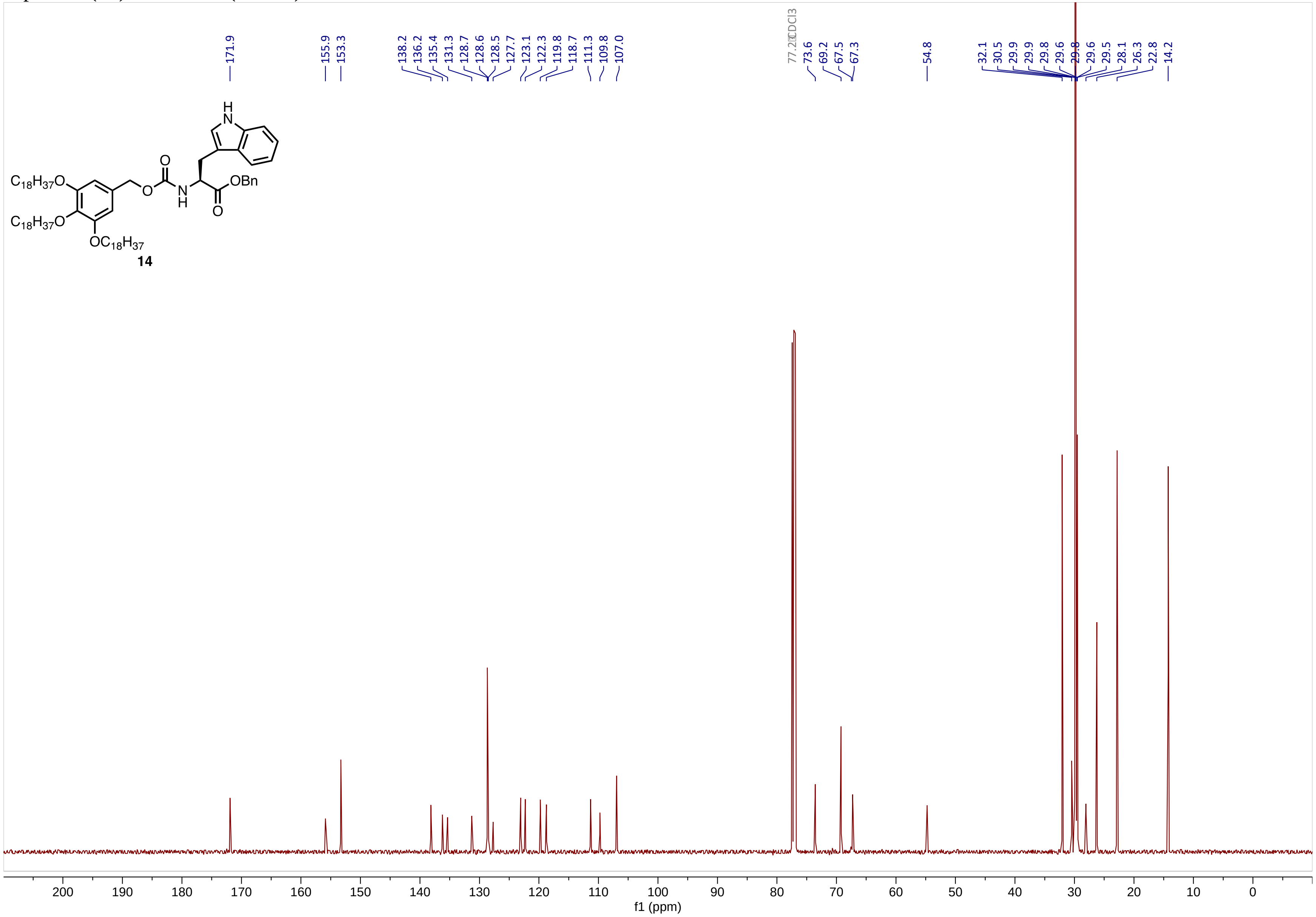

• TCbz-His(Trt)-OMe (**15**): ^{1}H NMR ($CDCl_3$)

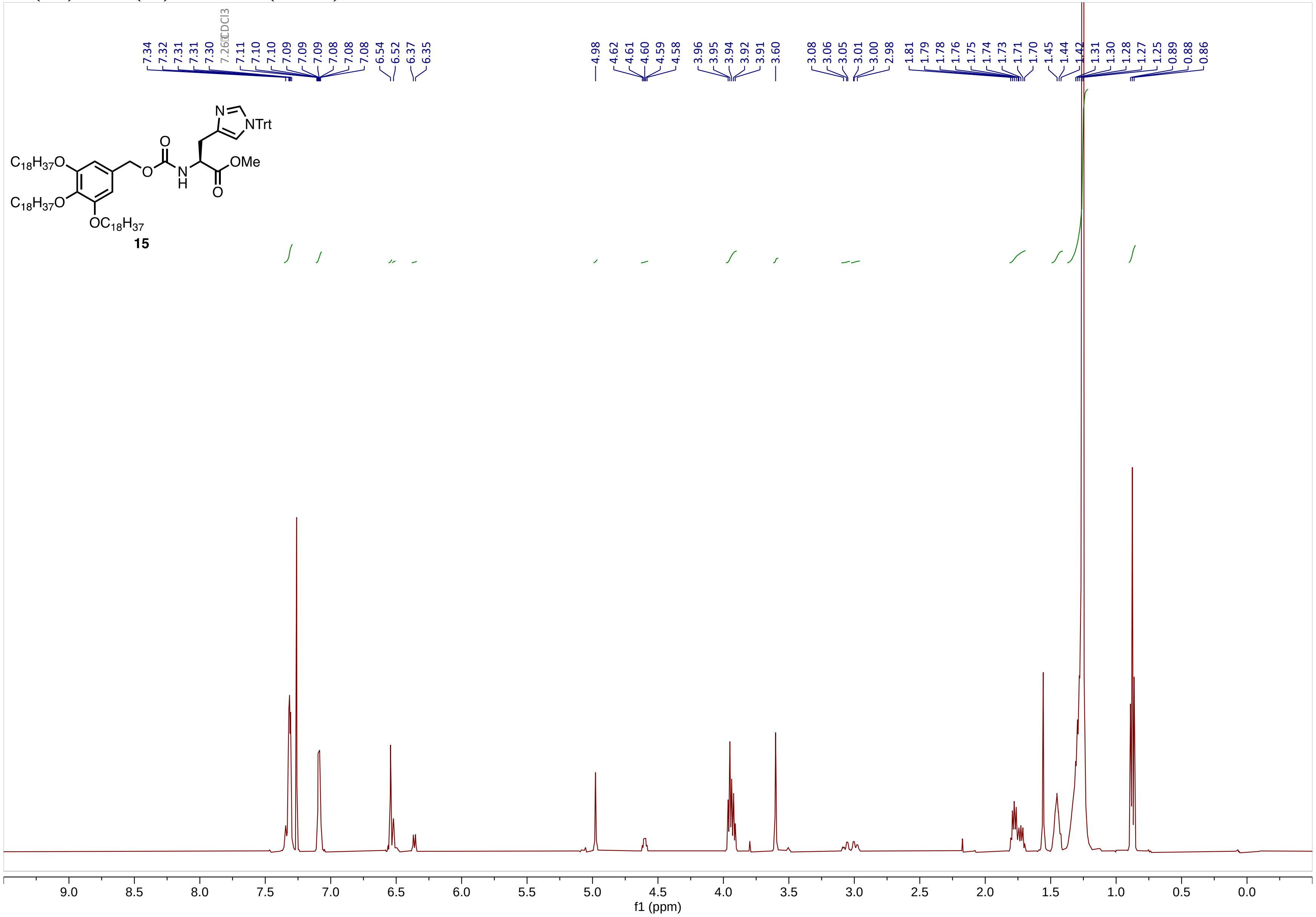

• TCbz-His(Trt)-OMe (**15**): ^{13}C NMR ($CDCl_3$)

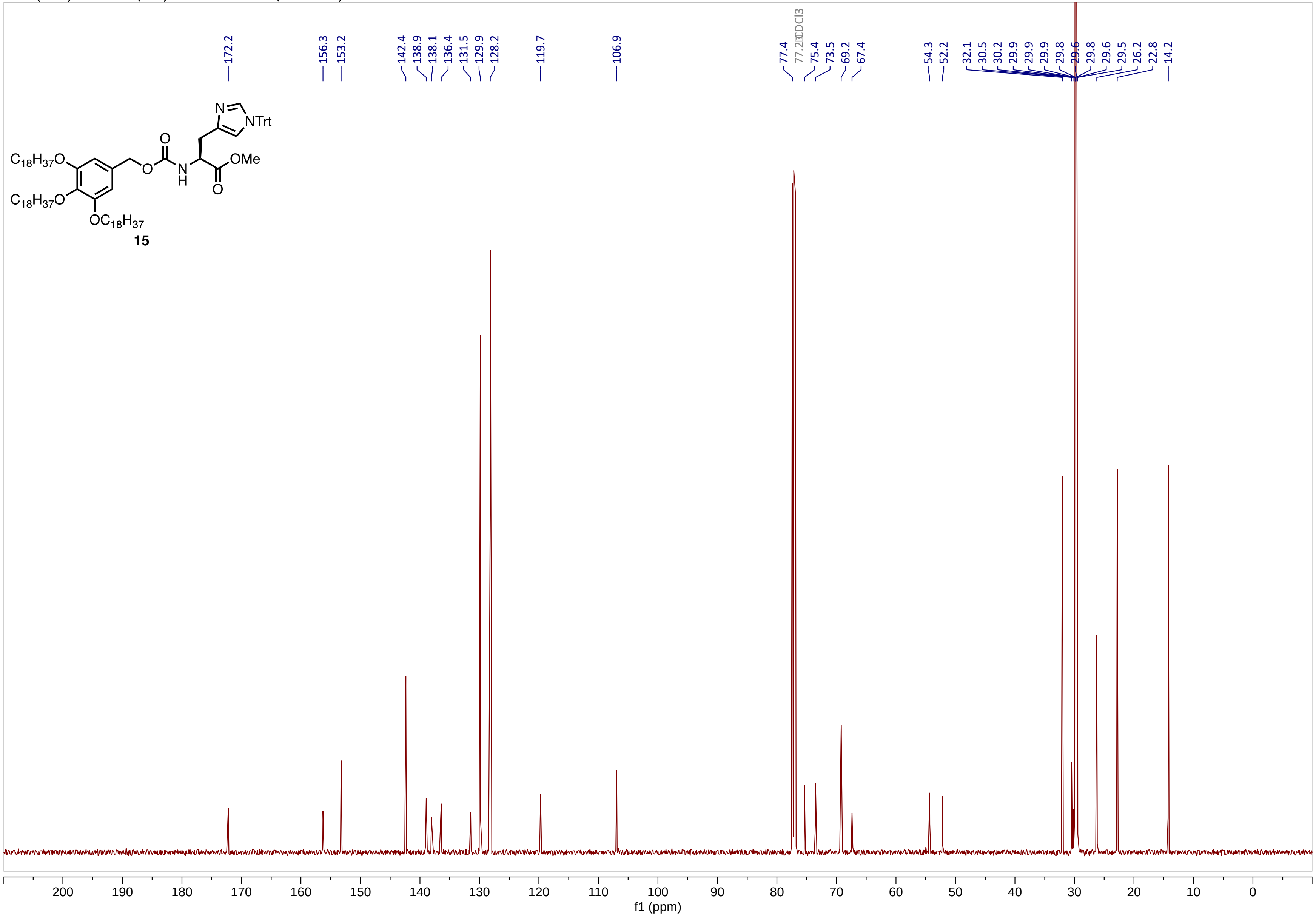

• TCbz-Ile-OTMSE (**16**): ^{1}H NMR ($CDCl_3$)

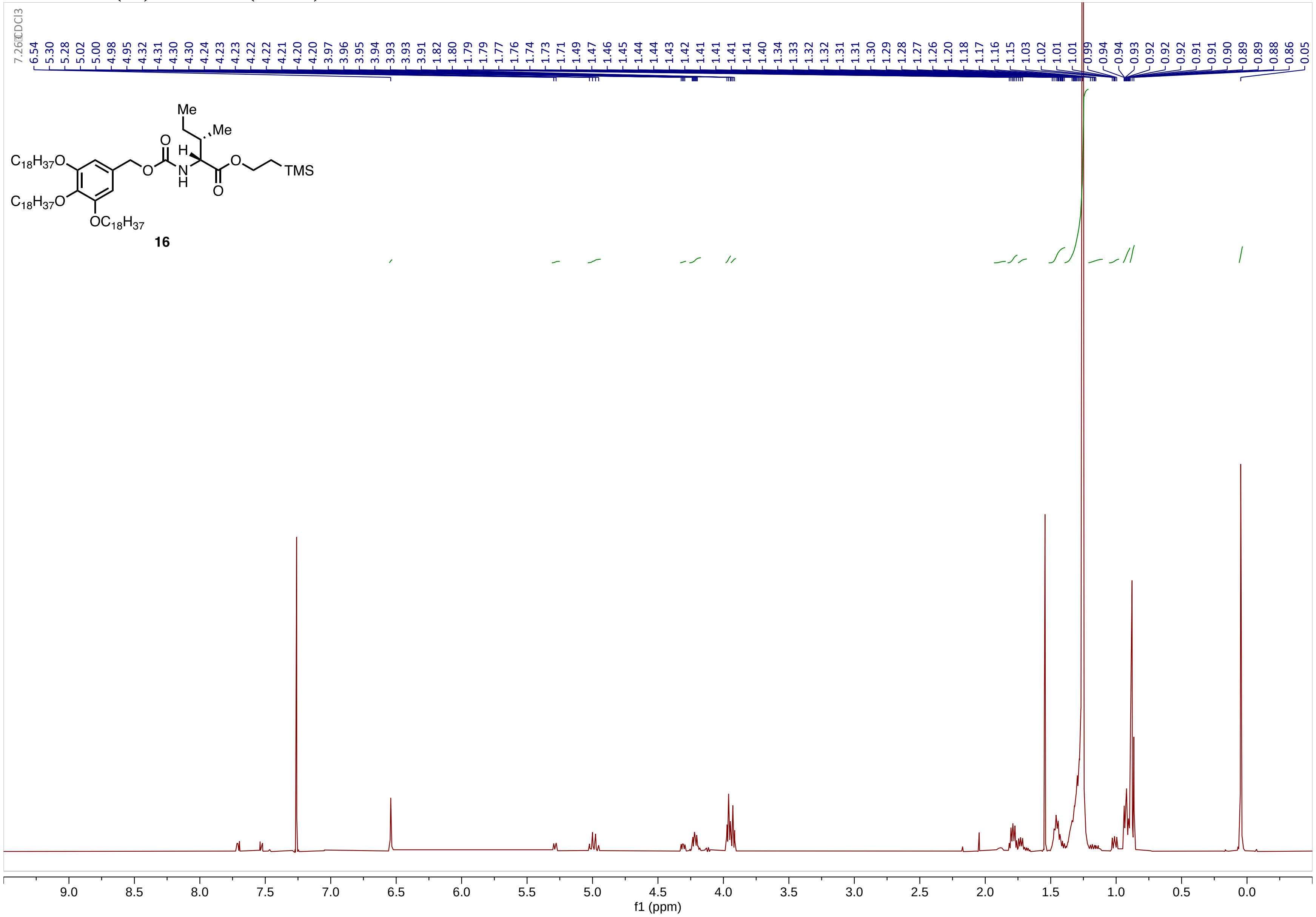

• TCbz-Ile-OTMSE (**16**): ^{13}C NMR ($CDCl_3$)

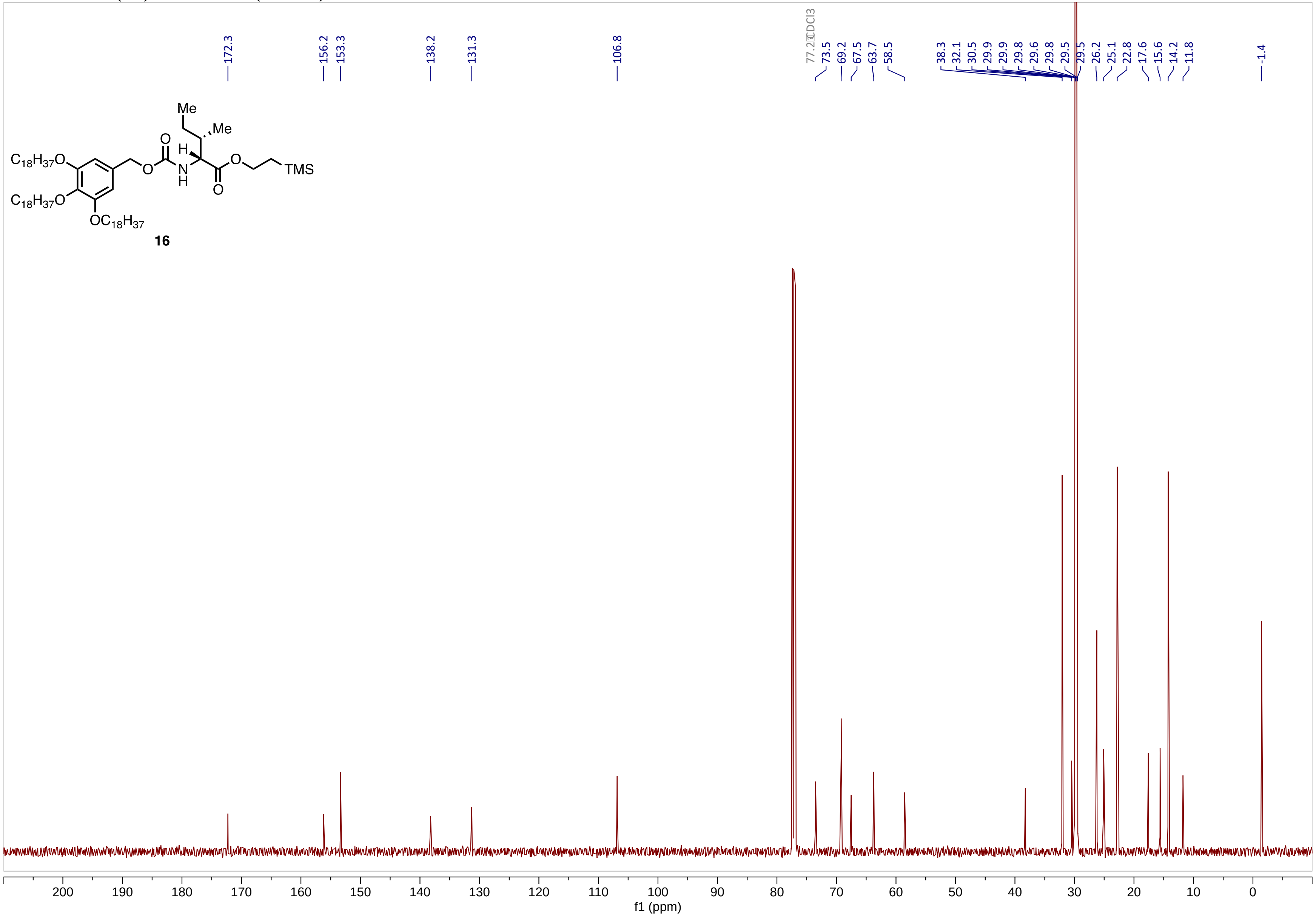

• TCbz-Met-OEt (**17**): ^{1}H NMR ($CDCl_3$)

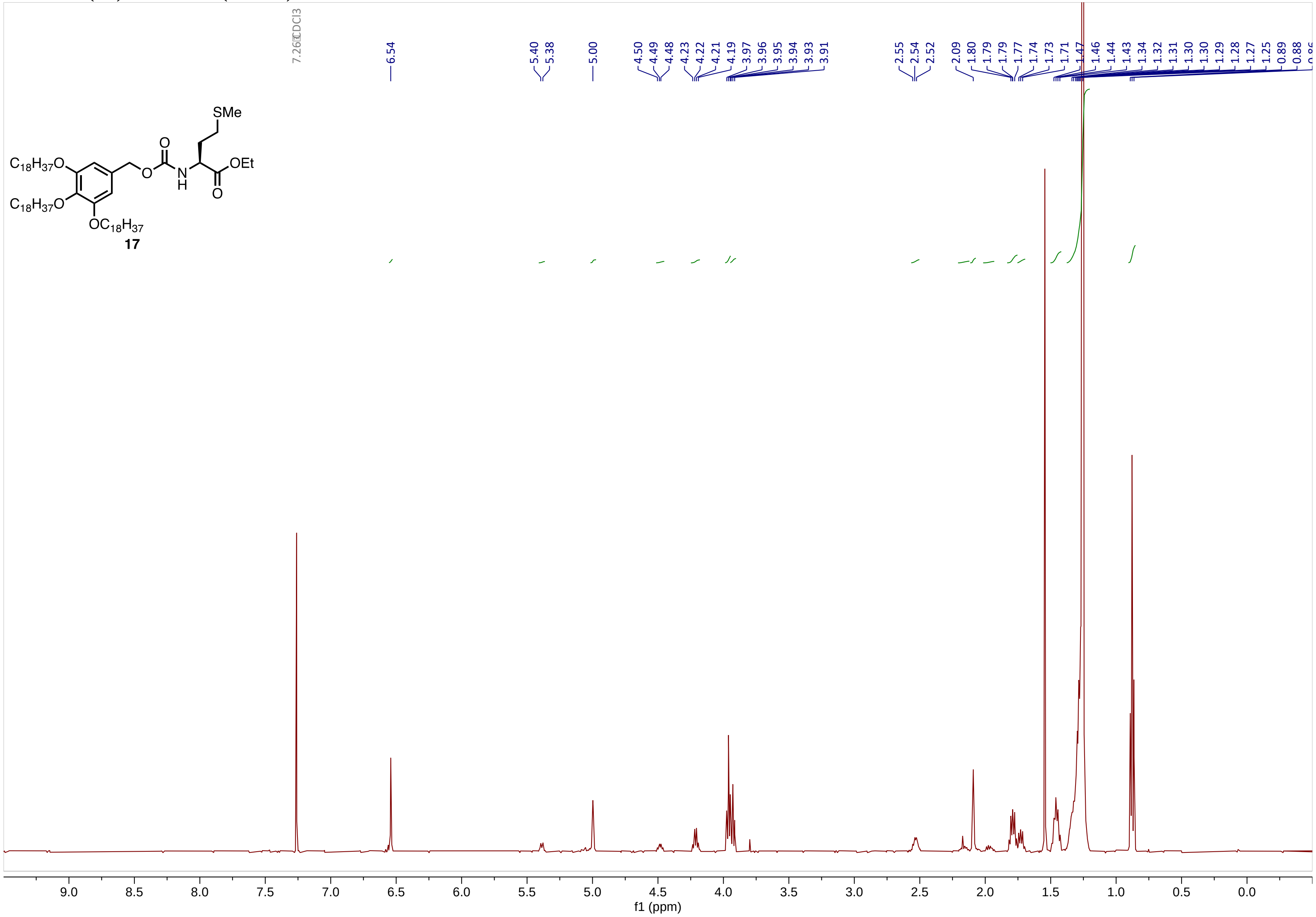

• TCbz-Met-OEt (**17**): ^{13}C NMR ($CDCl_3$)

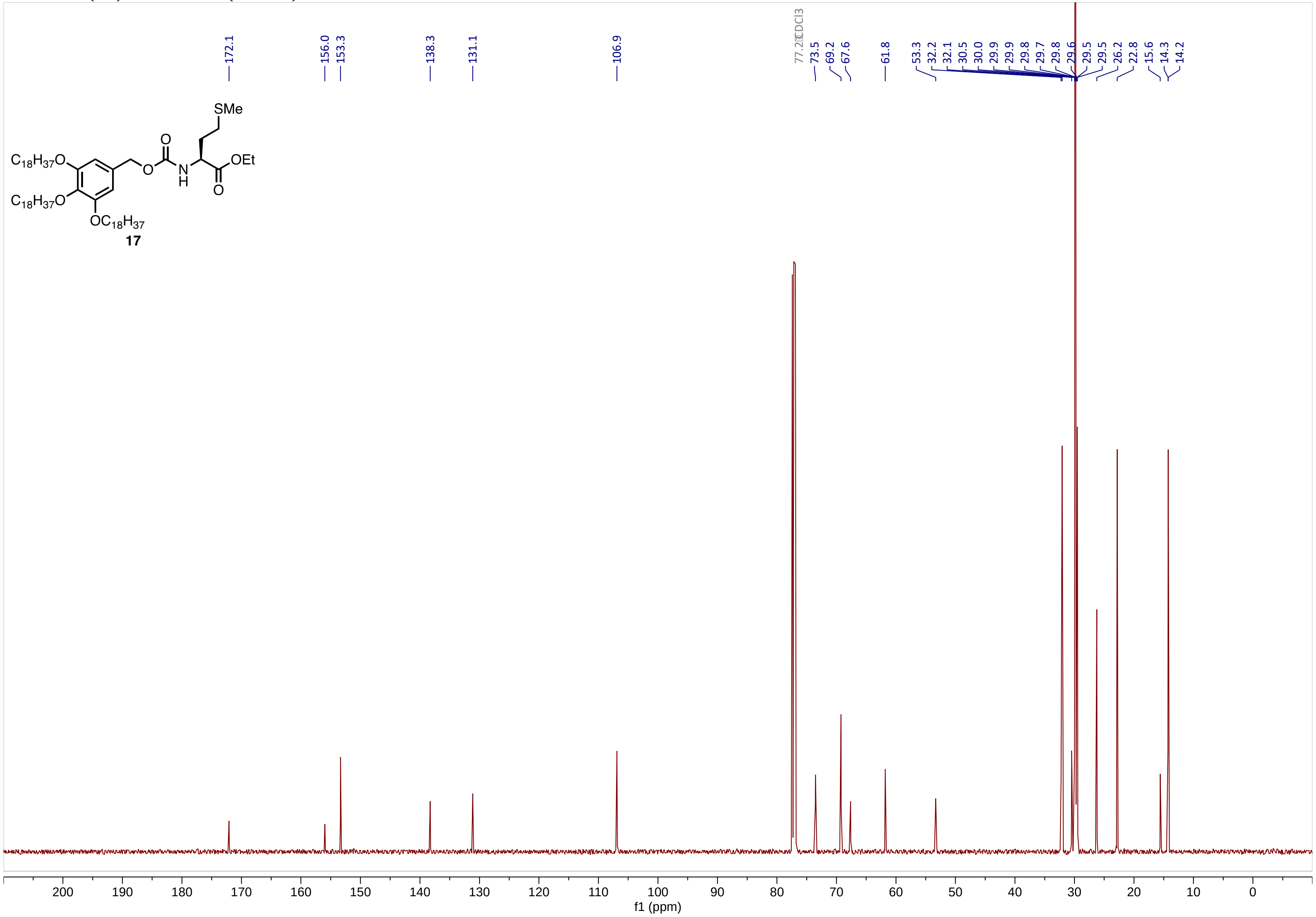

• TCbz-*N*-Me-Gly-OMe (**18**): ^1H NMR ($CDCl_3$)

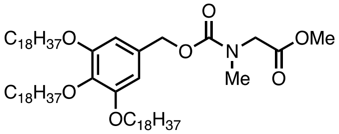

18

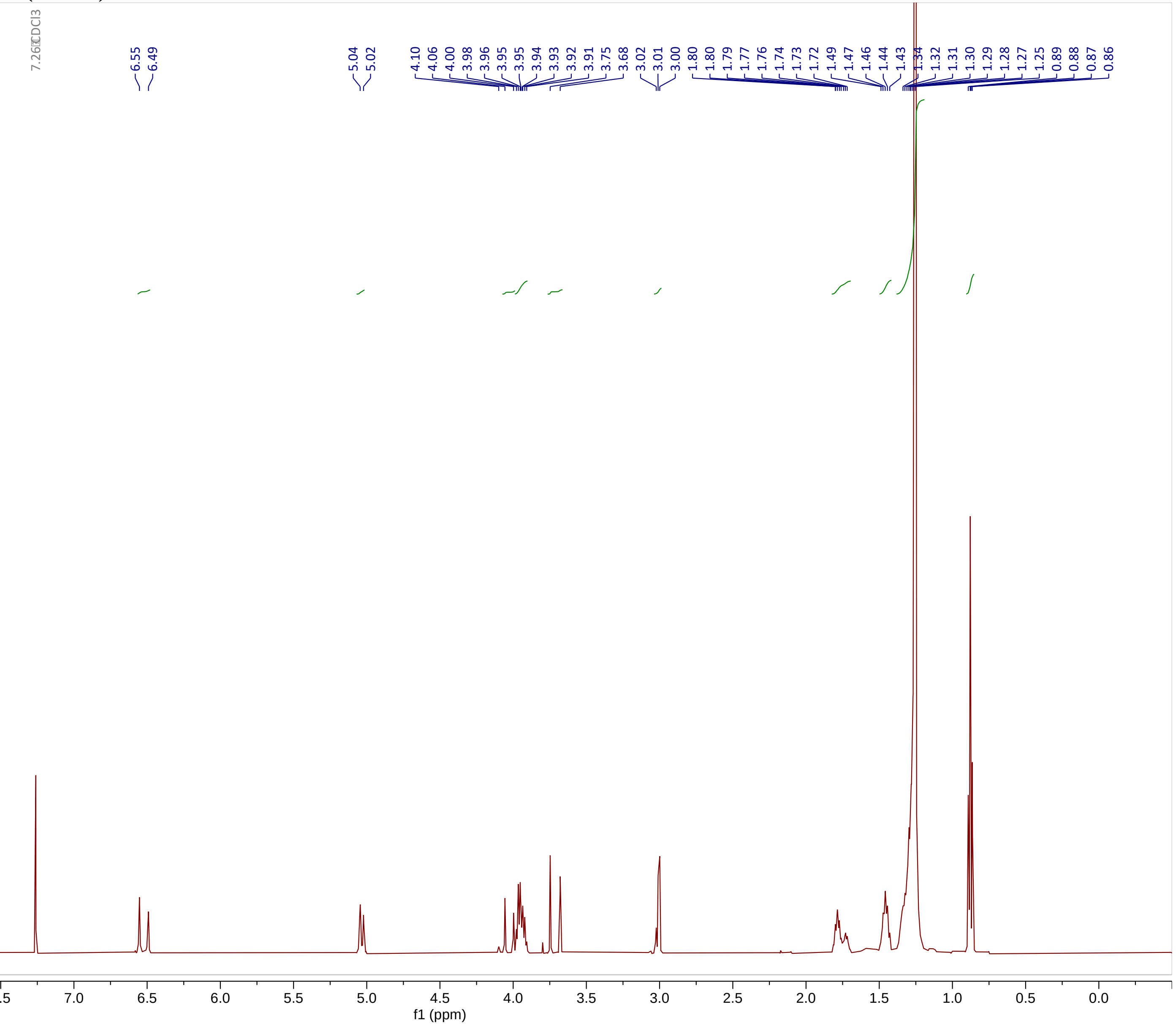

- TCbz-*N*-Me-Gly-OMe (**18**): ^{13}C NMR ($CDCl_3$)

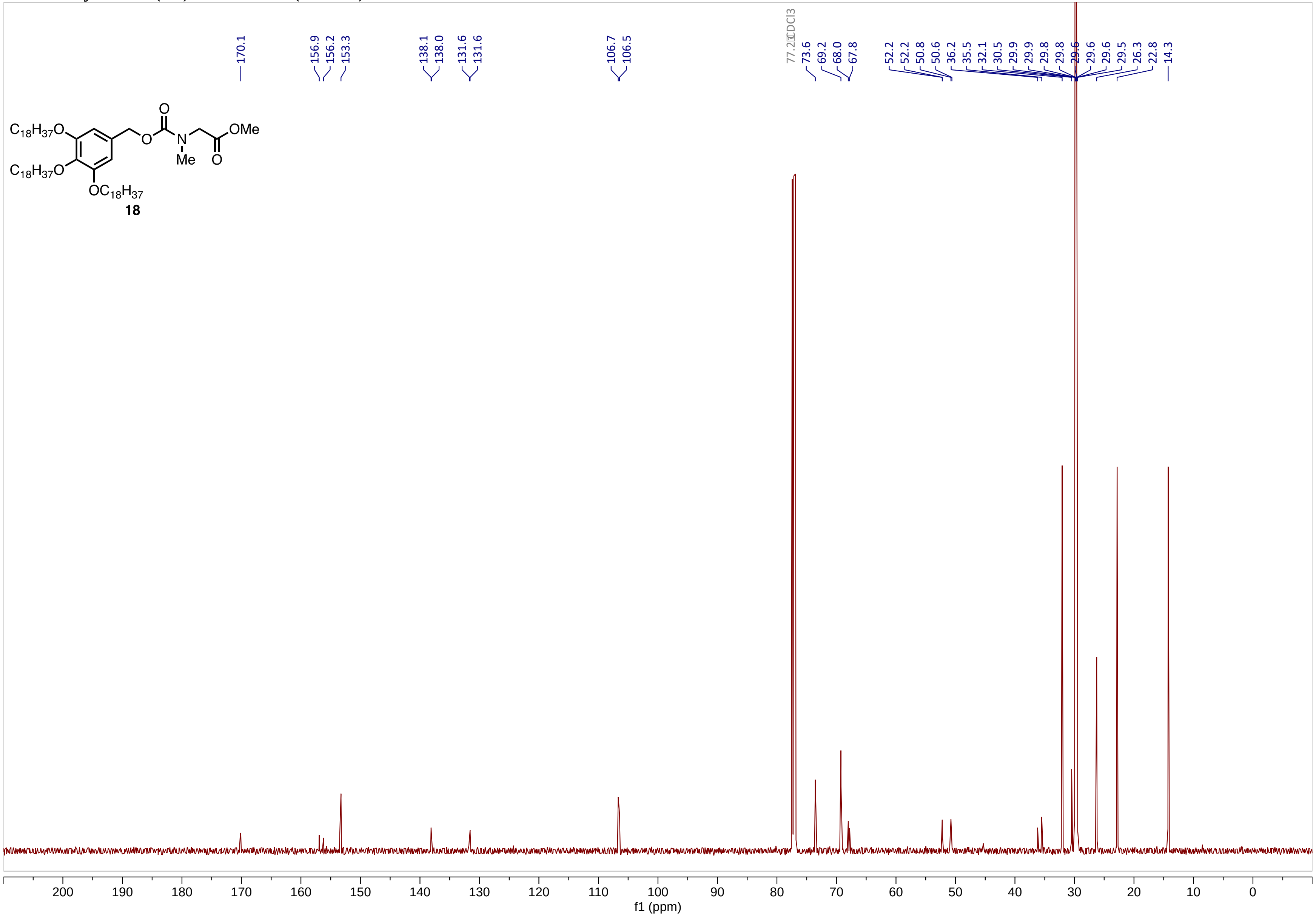

• TCbz-Ser-OMe (**19**): ^{1}H NMR ($CDCl_3$)

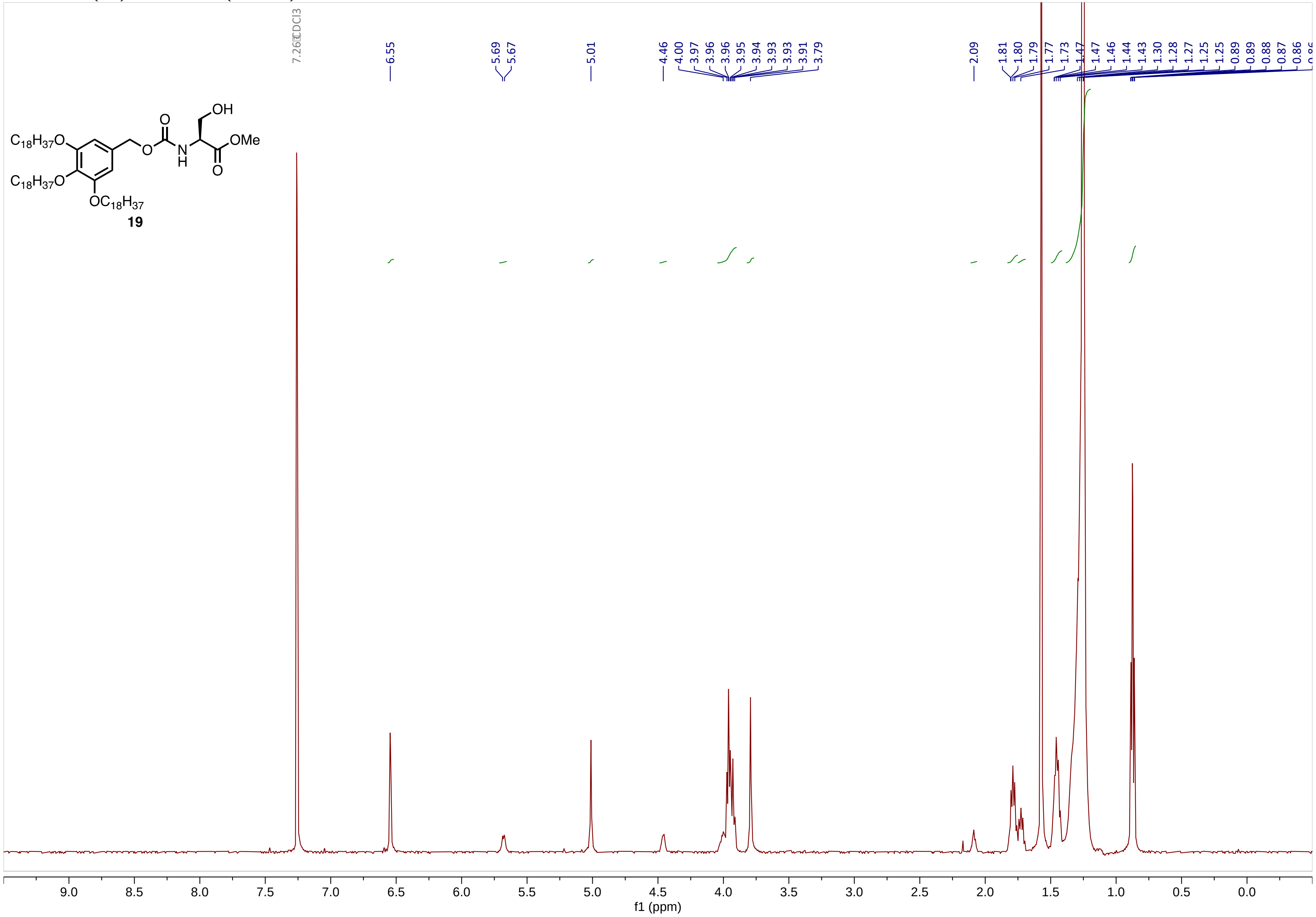

• TCbz-Ser-OMe (**19**): ^{13}C NMR ($CDCl_3$)

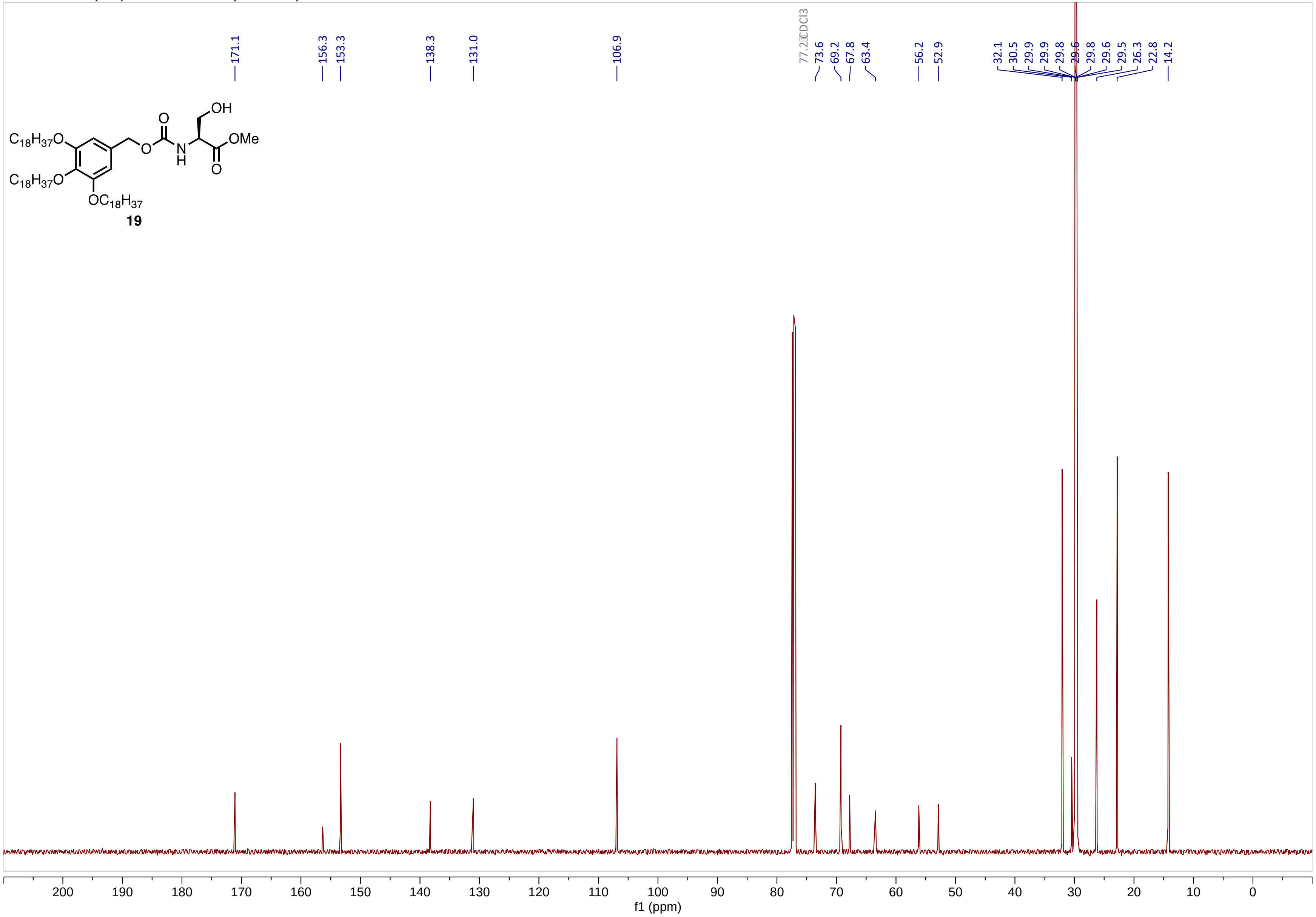

• TCbz-Glu(O^{t}Bu)-OMe (**20**): ^{1}H NMR ($CDCl_3$)

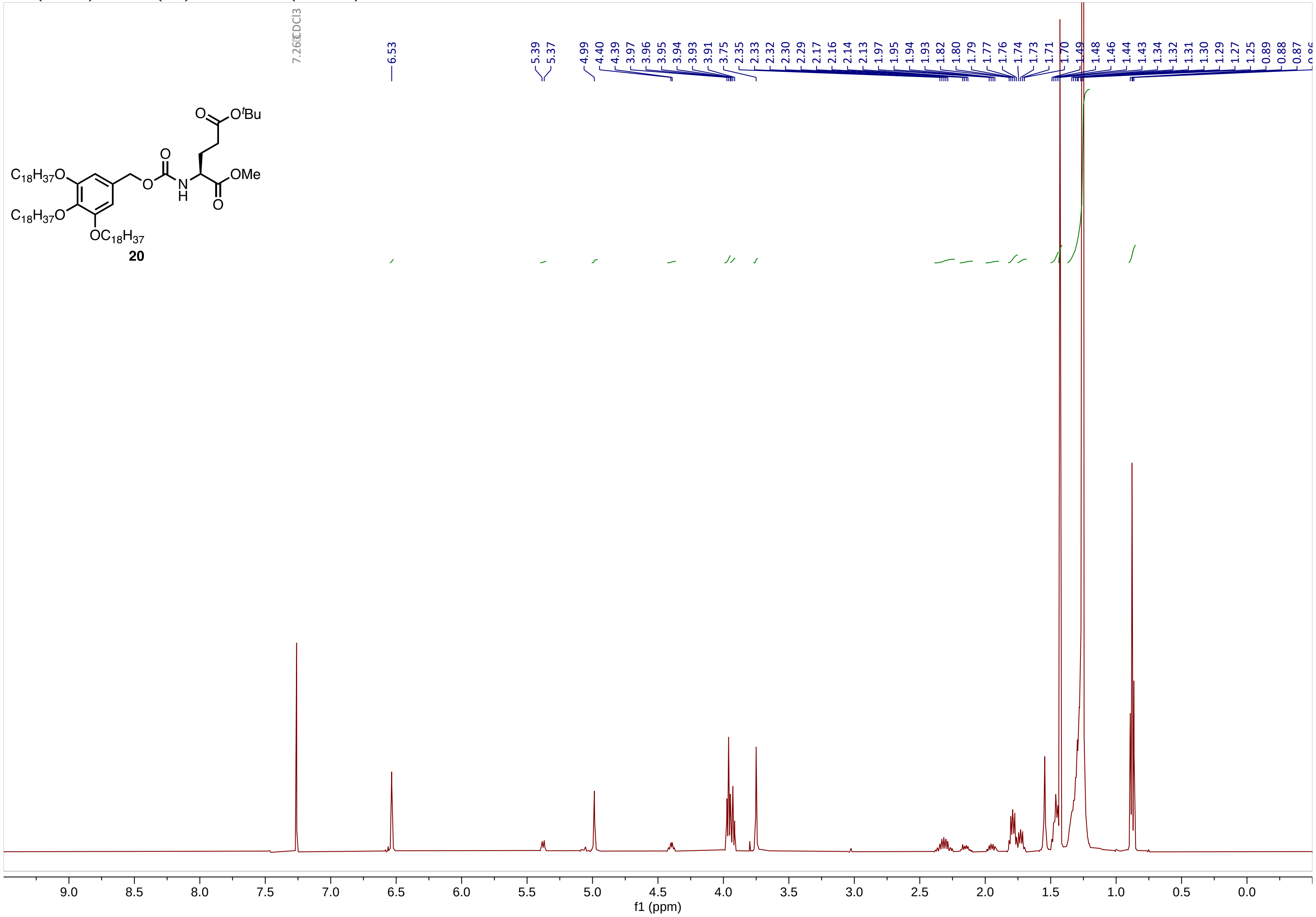

• TCbz-Glu(O^{t}Bu)-OMe (**20**): ^{13}C NMR ($CDCl_3$)

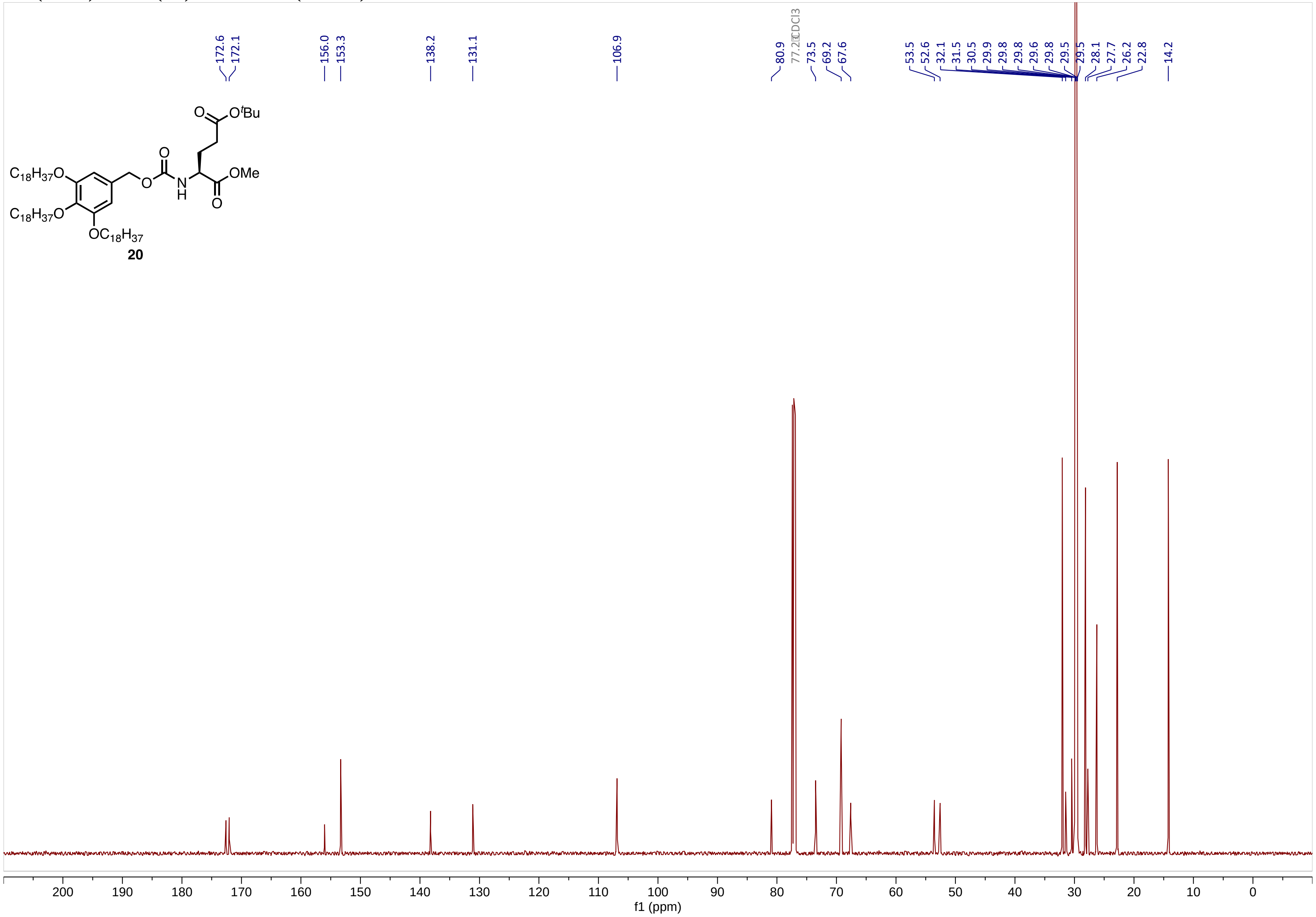

• TCbz-Orn(Cbz)-OMe (**21**): ^{1}H NMR ($CDCl_3$)

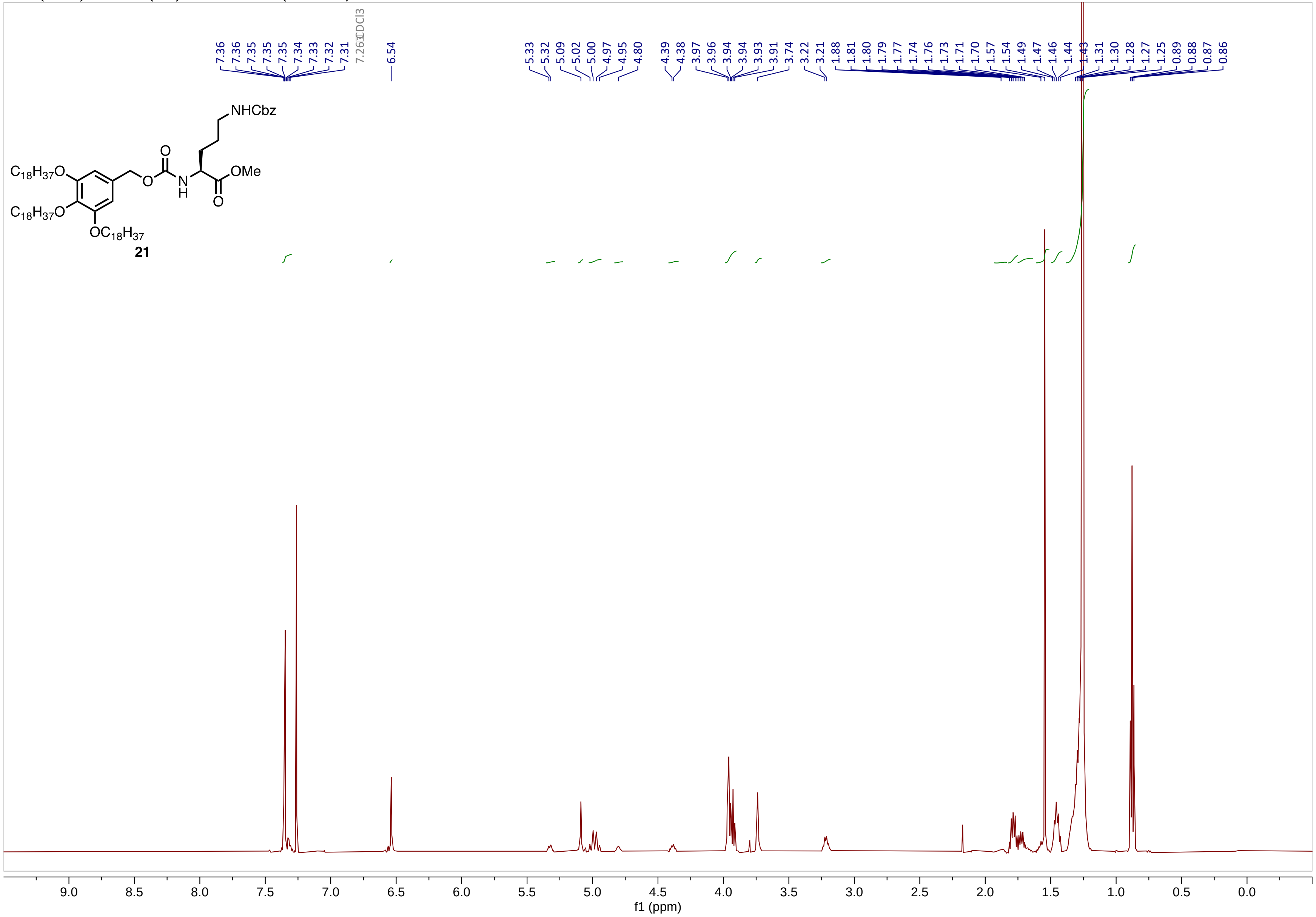

• TCbz-Orn(Cbz)-OMe (**21**): ^{13}C NMR ($CDCl_3$)

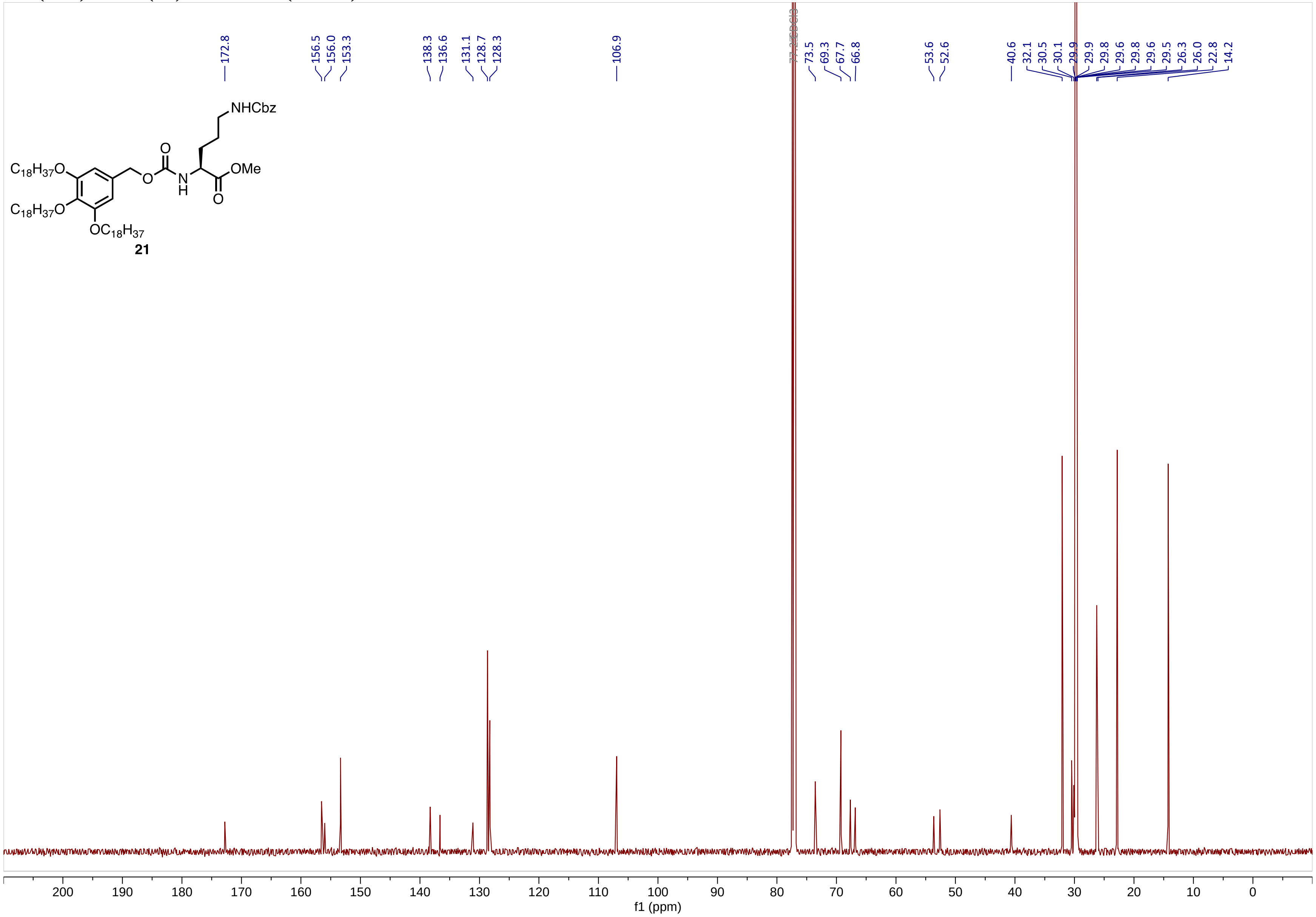

- Fmoc-Orn(TCbz)-OMe (**11**): ^{1}H NMR ($CDCl_3$)

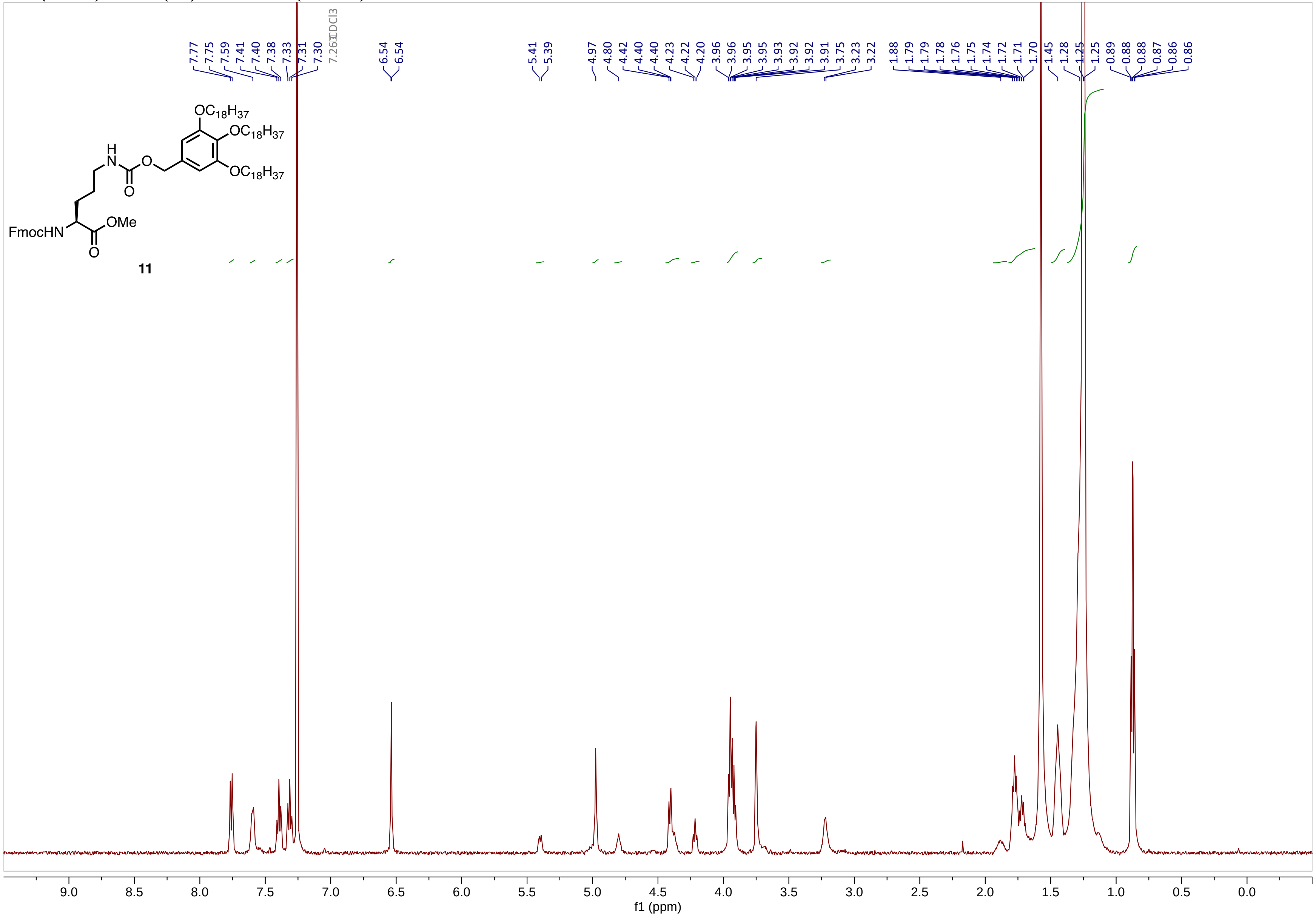

• Fmoc-Orn(TCbz)-OMe (**11**): ^{13}C NMR ($CDCl_3$)

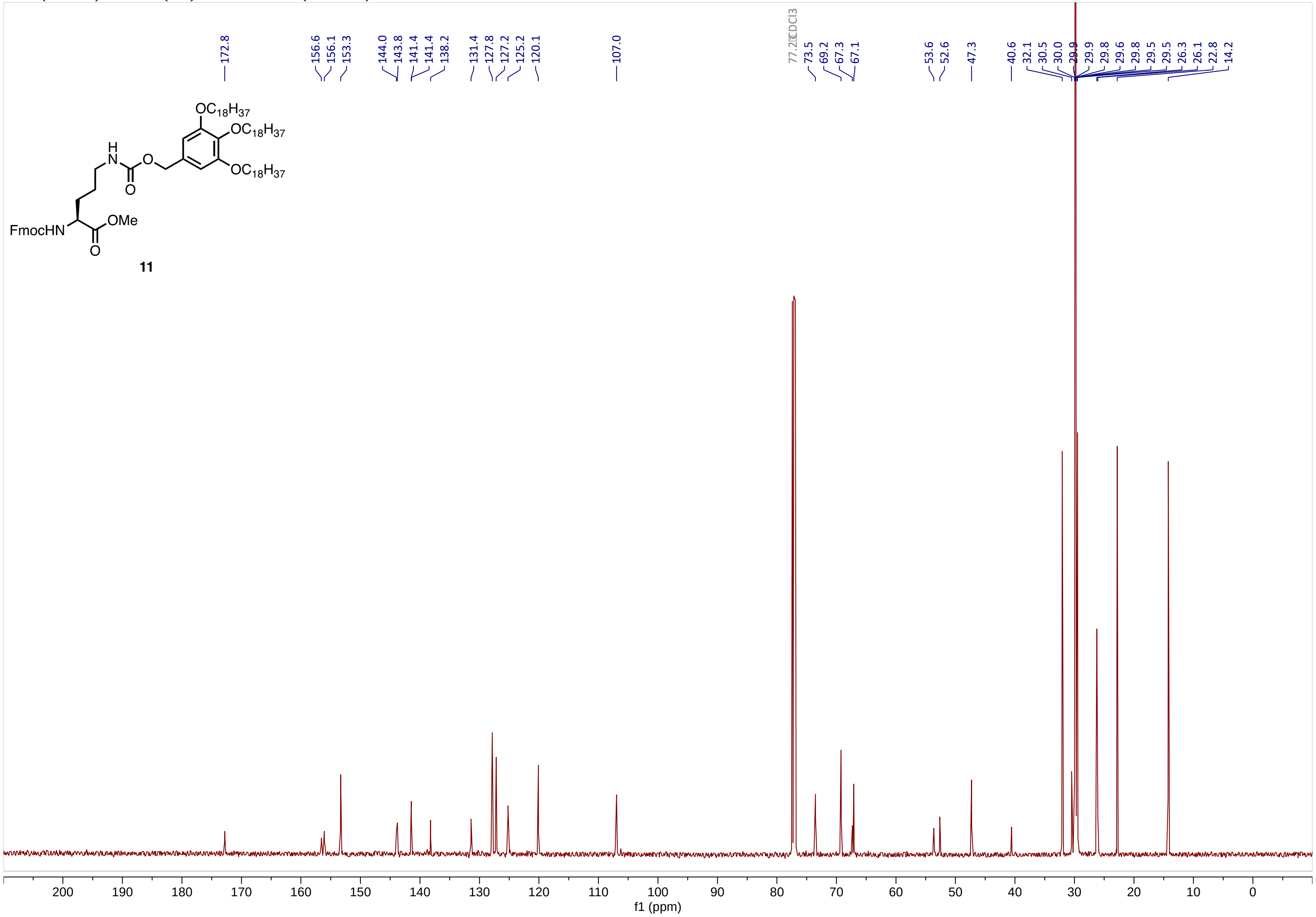

• TCbz-Lys(Boc)-OMe (**22**): ^{1}H NMR ($CDCl_3$)

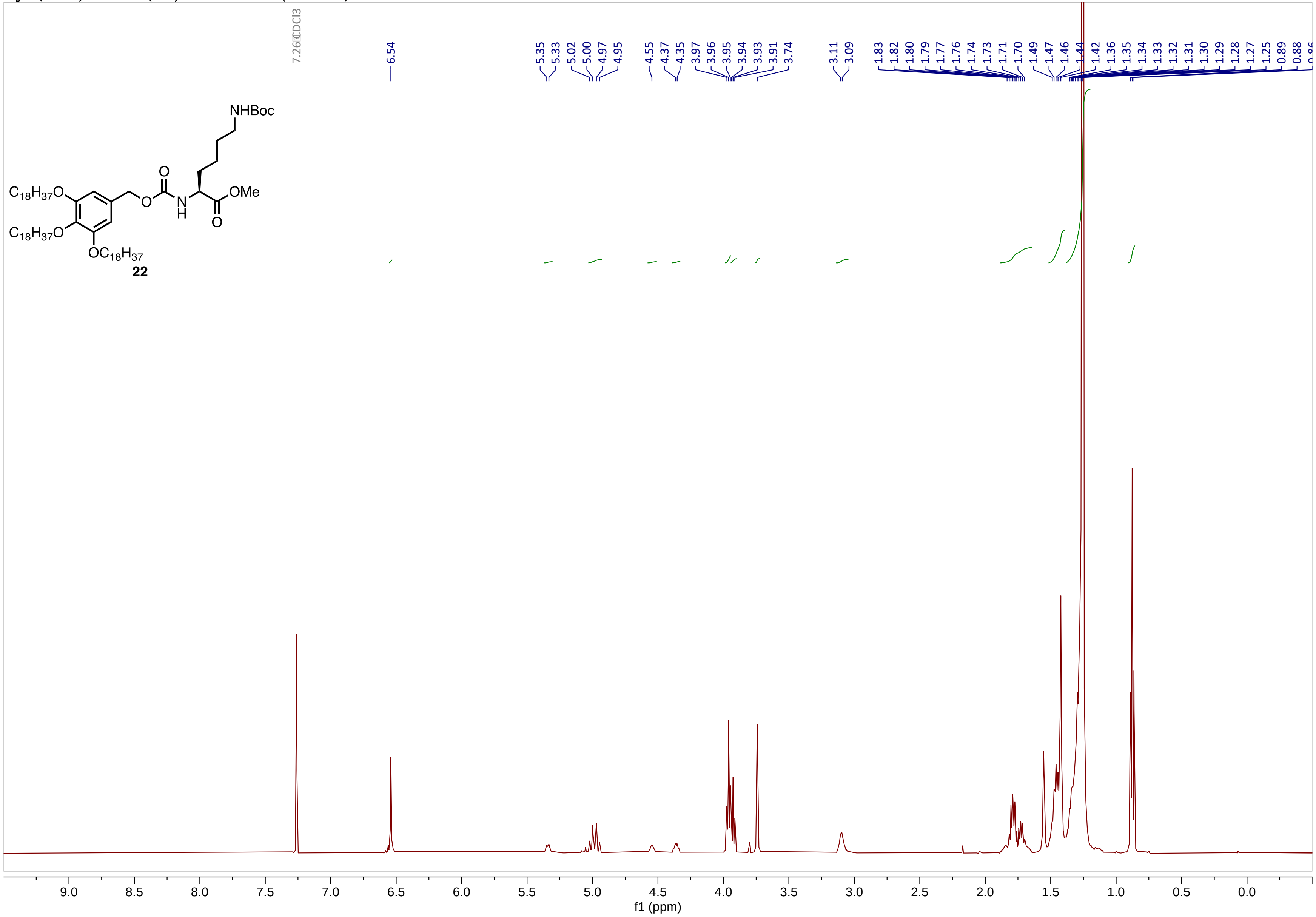

• TCbz-Lys(Boc)-OMe (**22**): ^{13}C NMR ($CDCl_3$)

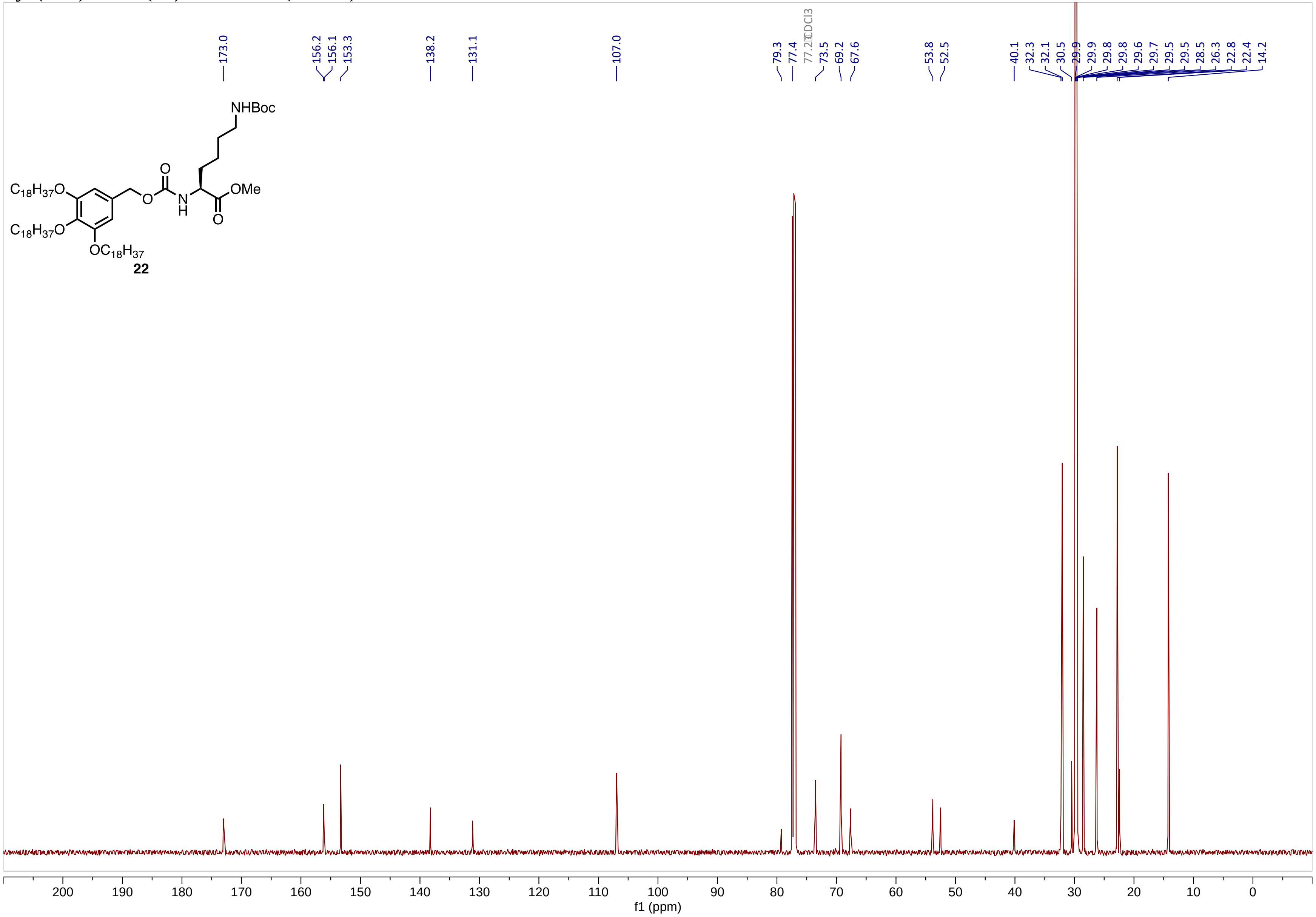

• Fmoc-Lys(TCbz)-OMe (**23**): ^{1}H NMR ($CDCl_3$)

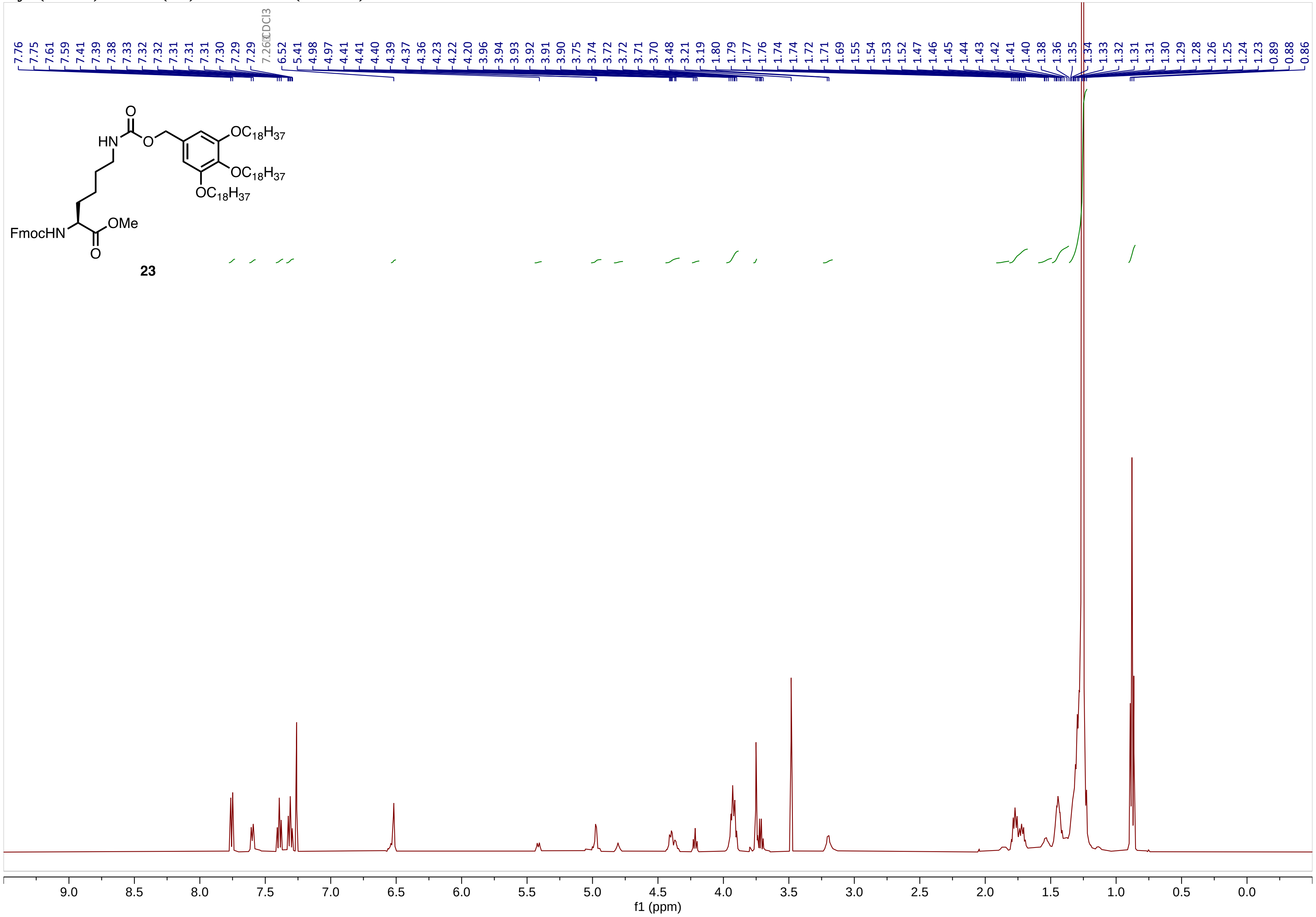

• Fmoc-Lys(TCbz)-OMe (**23**): ^{13}C NMR ($CDCl_3$)

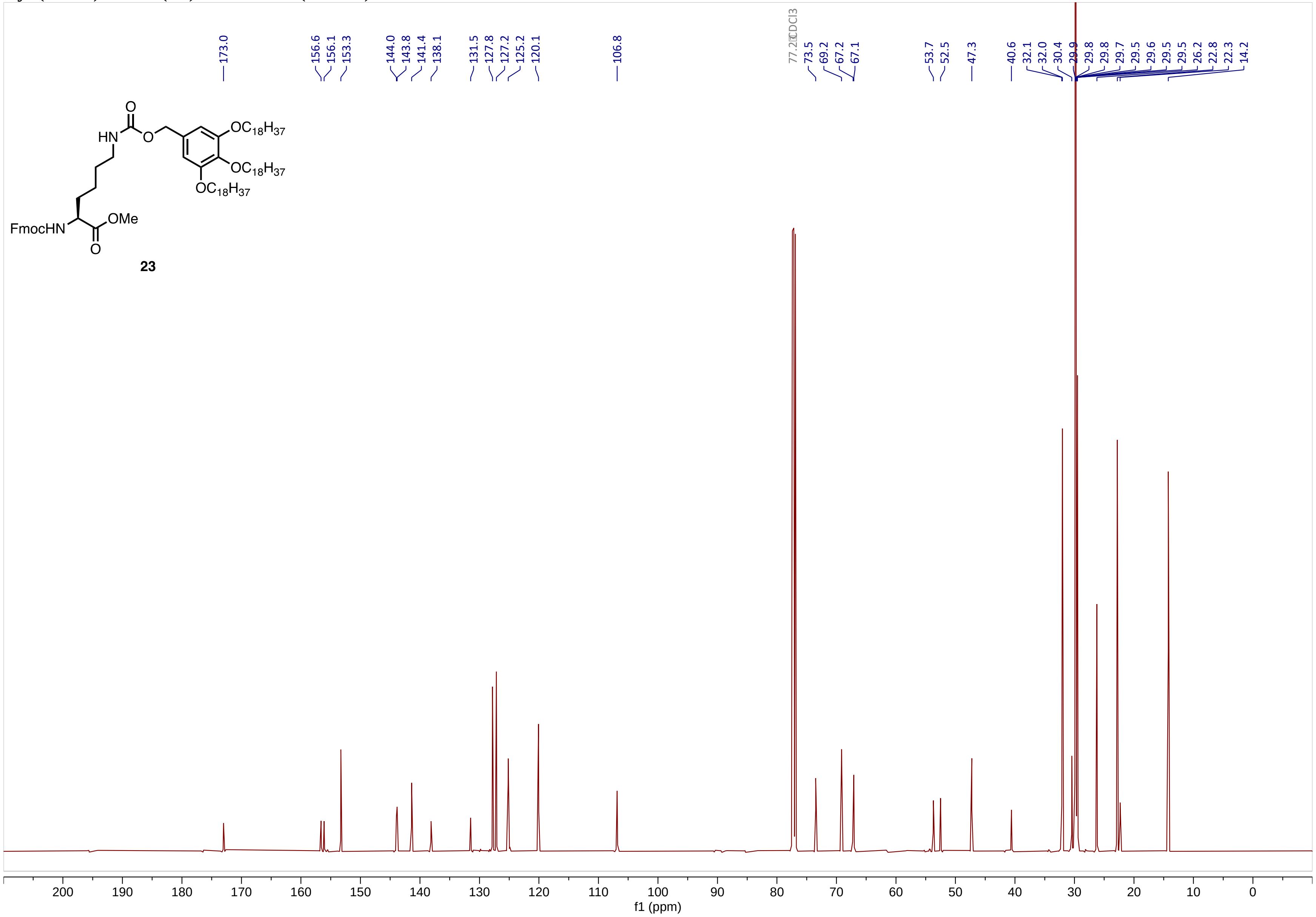

• Cbz-His(TCbz)-OMe (**24a**): ^{1}H NMR ($CDCl_3$)

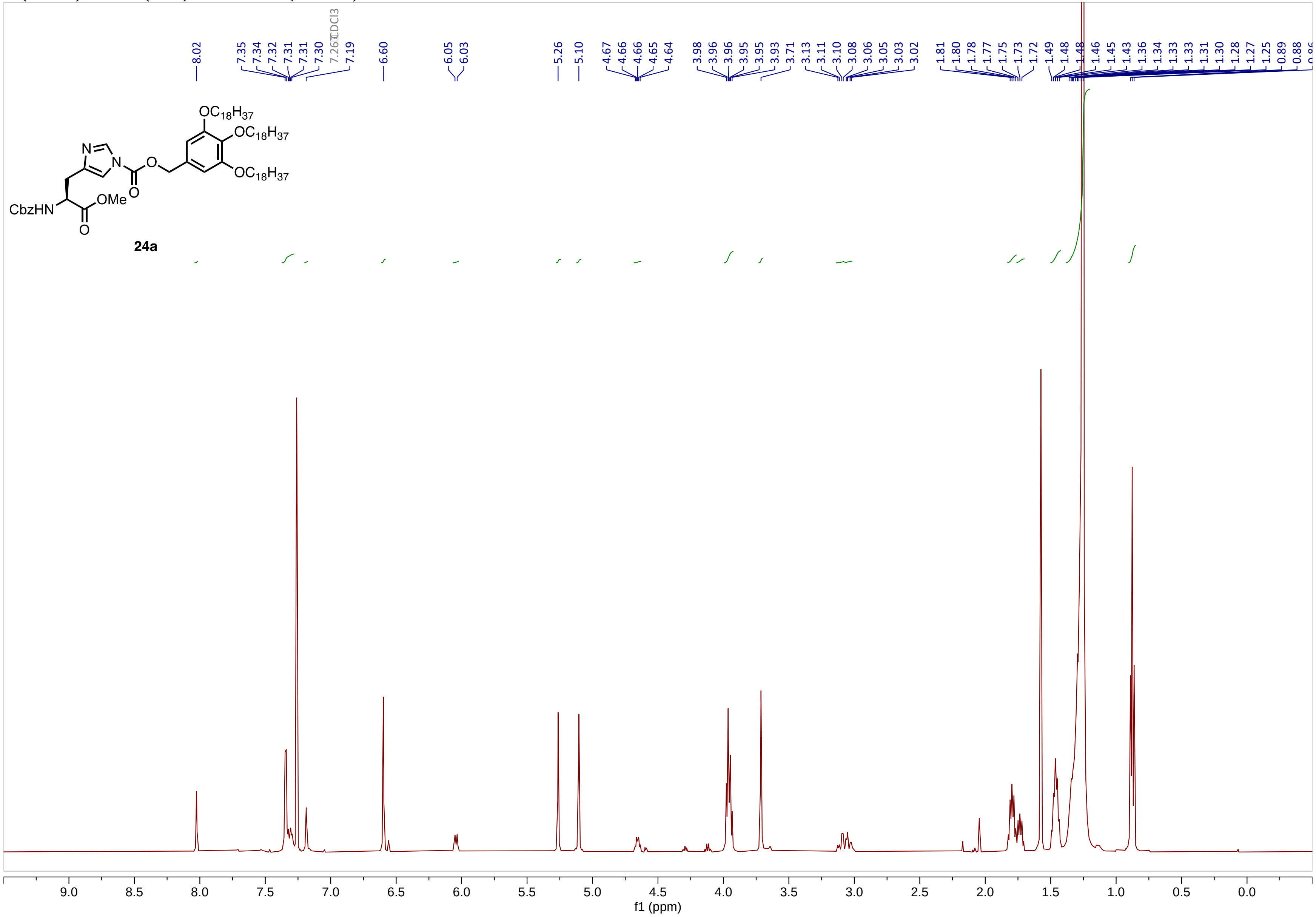

• Cbz-His(TCbz)-OMe (**24a**): ^{13}C NMR ($CDCl_3$)

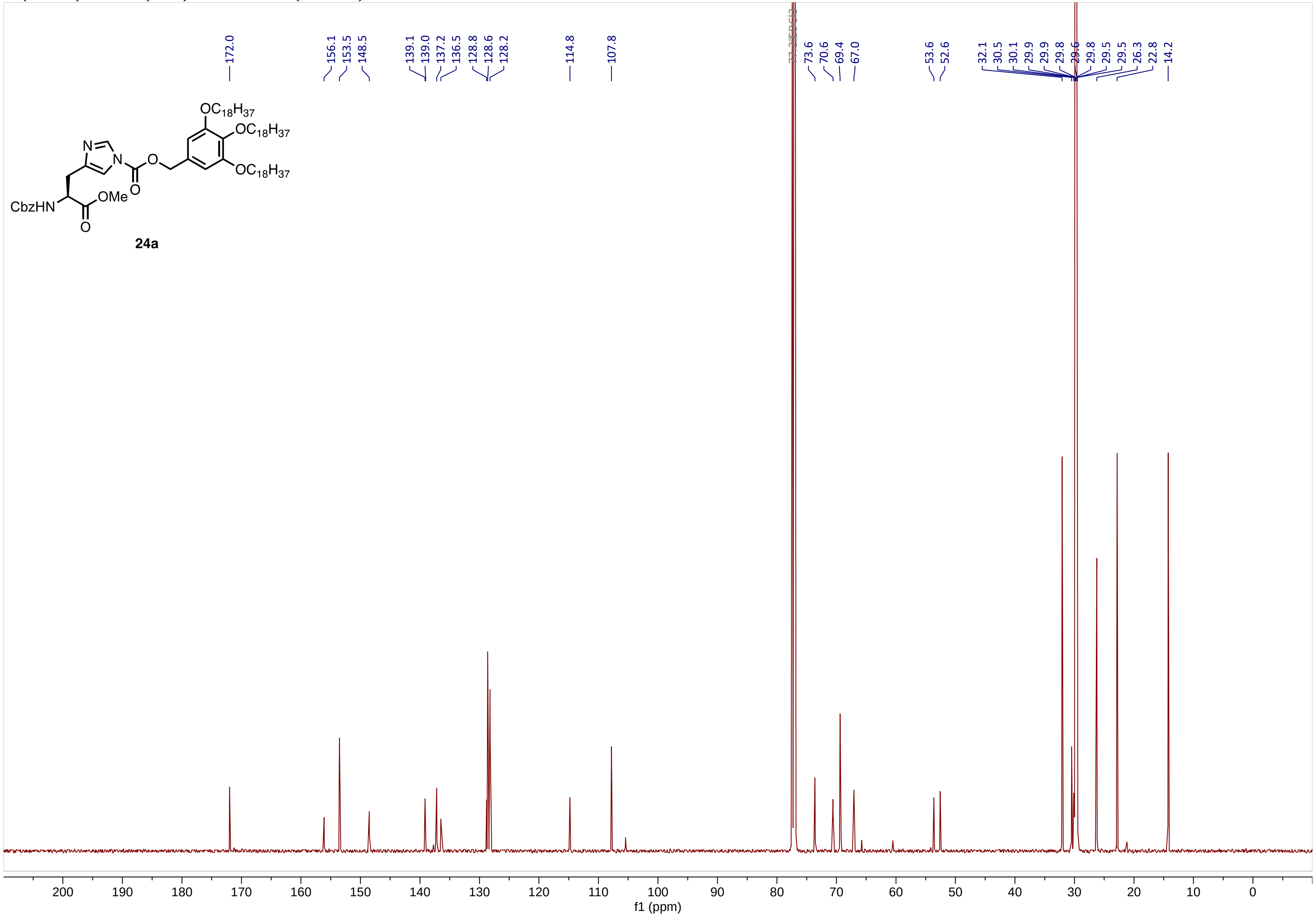

• Cbz-His(TCbz)-OMe (**24b**): ^{1}H NMR ($CDCl_3$)

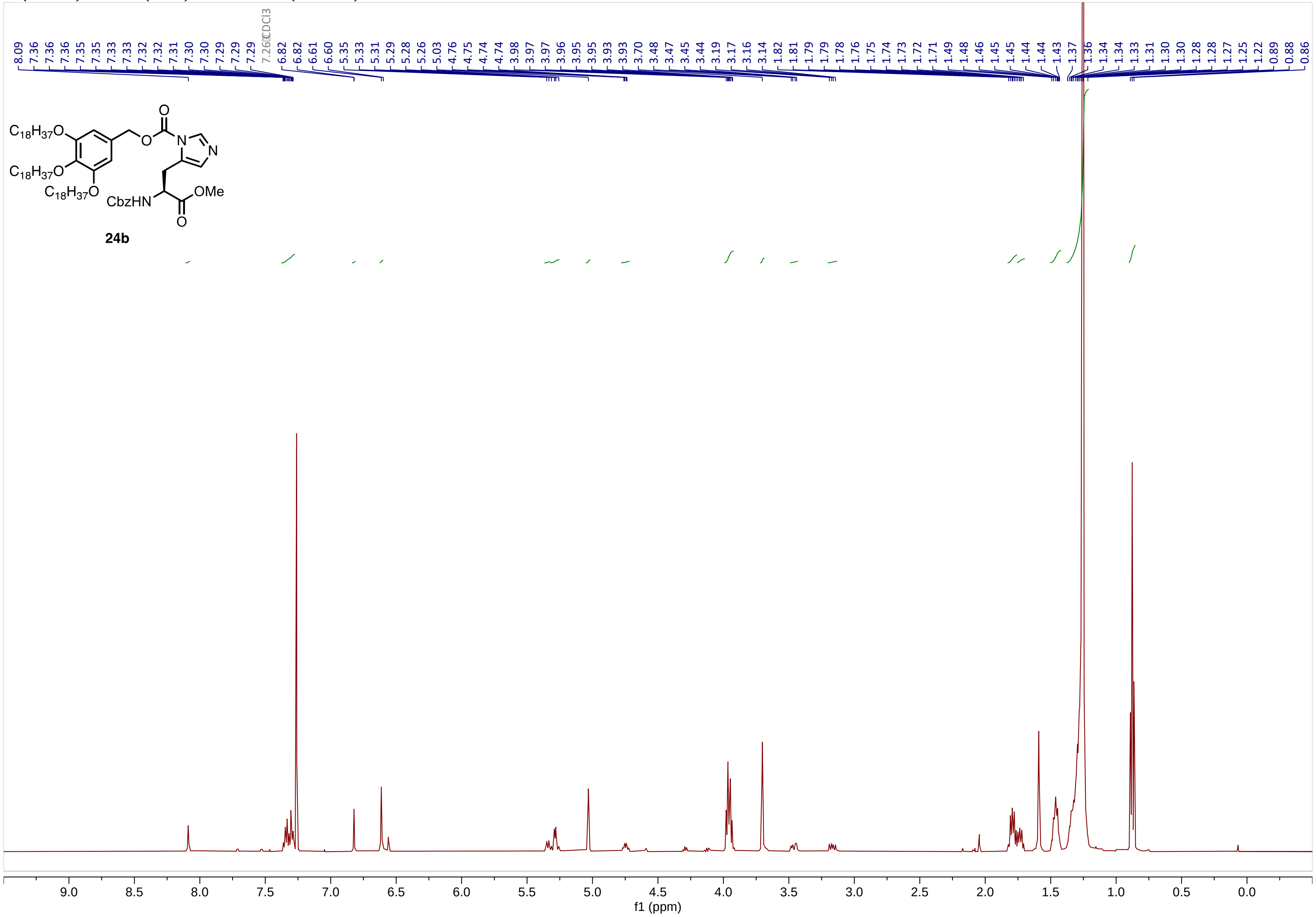

• Cbz-His(TCbz)-OMe (**24b**): ^{13}C NMR ($CDCl_3$)

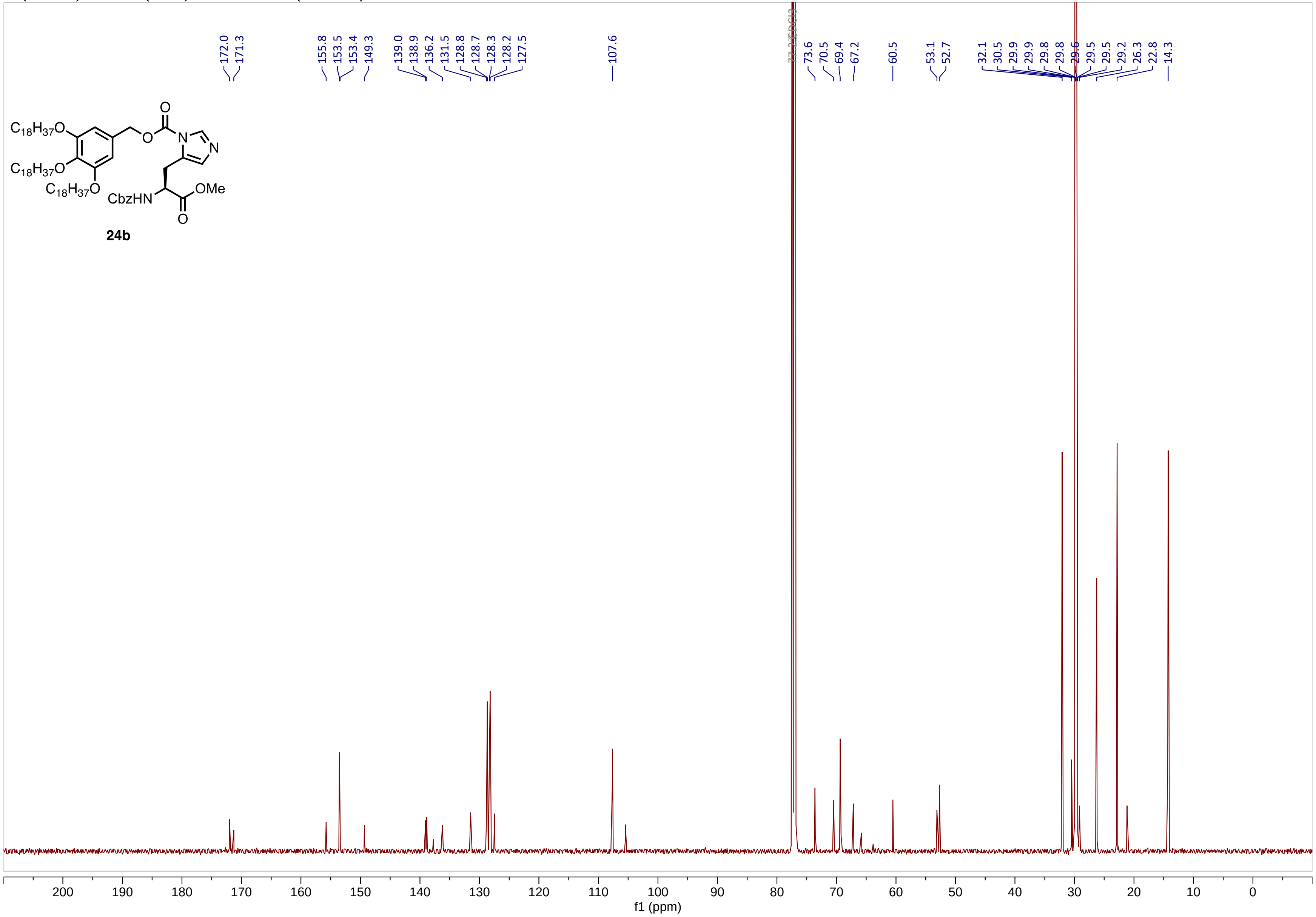

• Fmoc-D-Cys(TCbz)-OAllyl (**25**): ^{1}H NMR ($CDCl_3$)

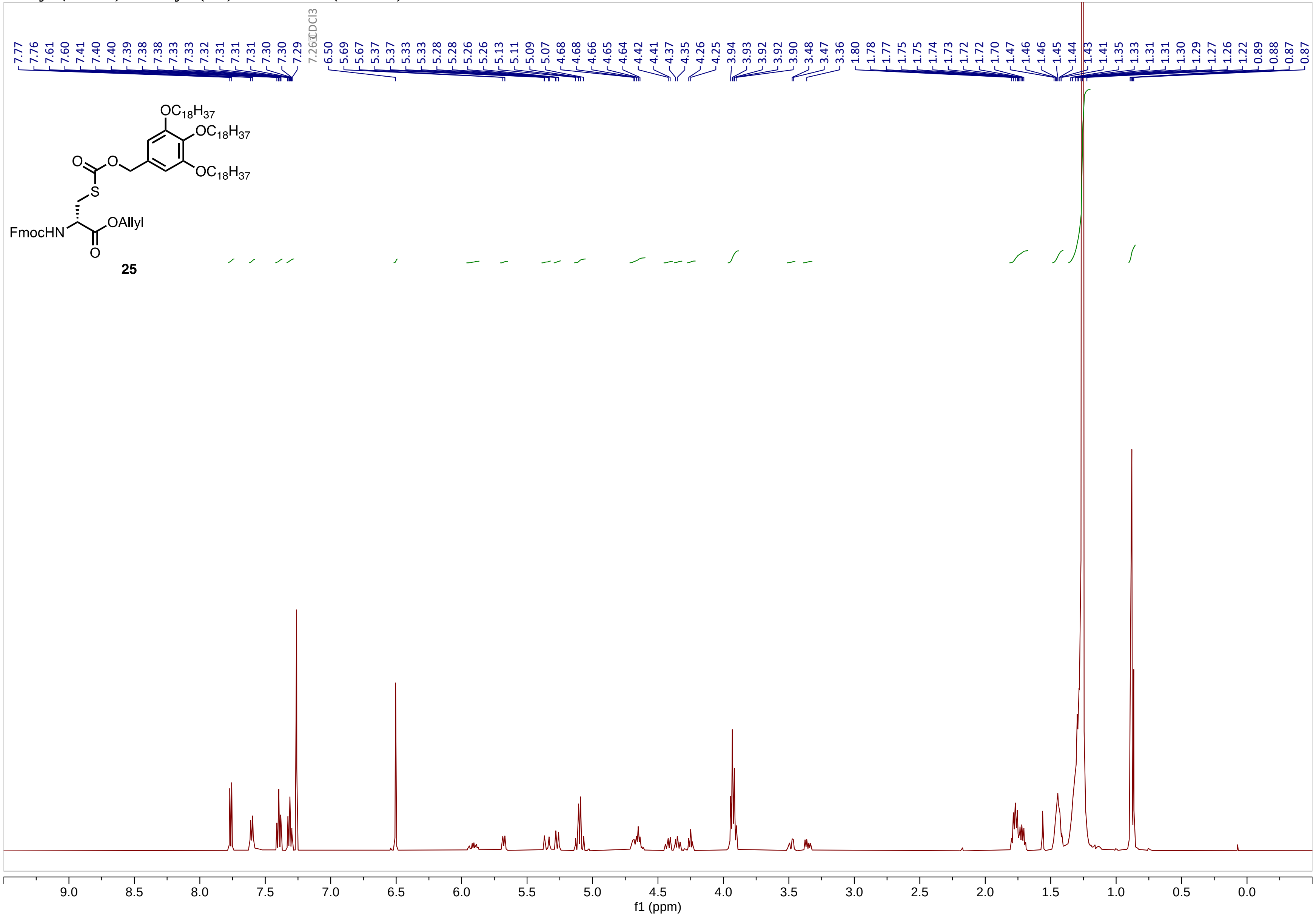

• Fmoc-D-Cys(TCbz)-OAllyl (**25**): ^{13}C NMR ($CDCl_3$)

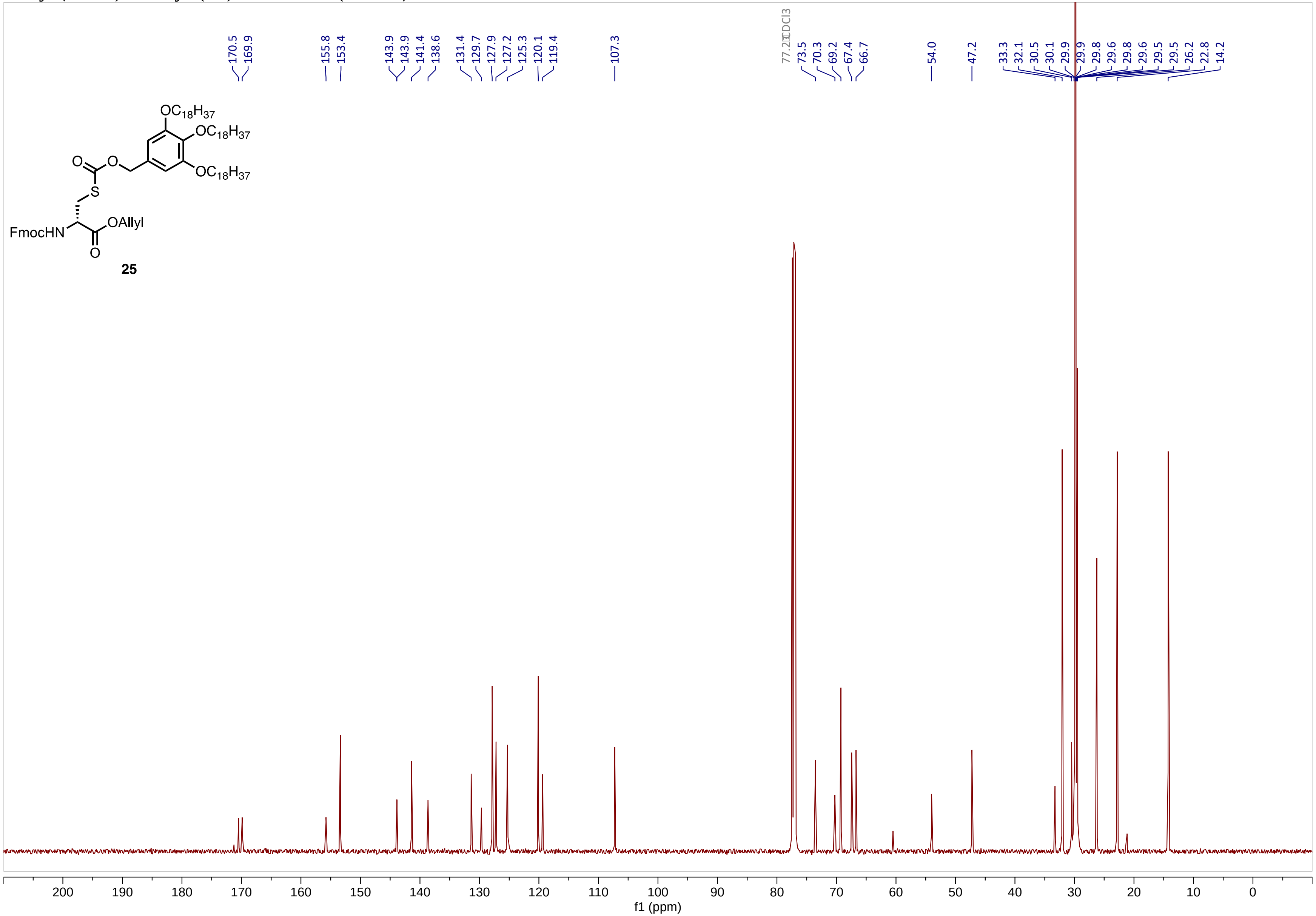

• TCbz-D-Phe-OAllyl (**26**): ^{1}H NMR ($CDCl_3$)

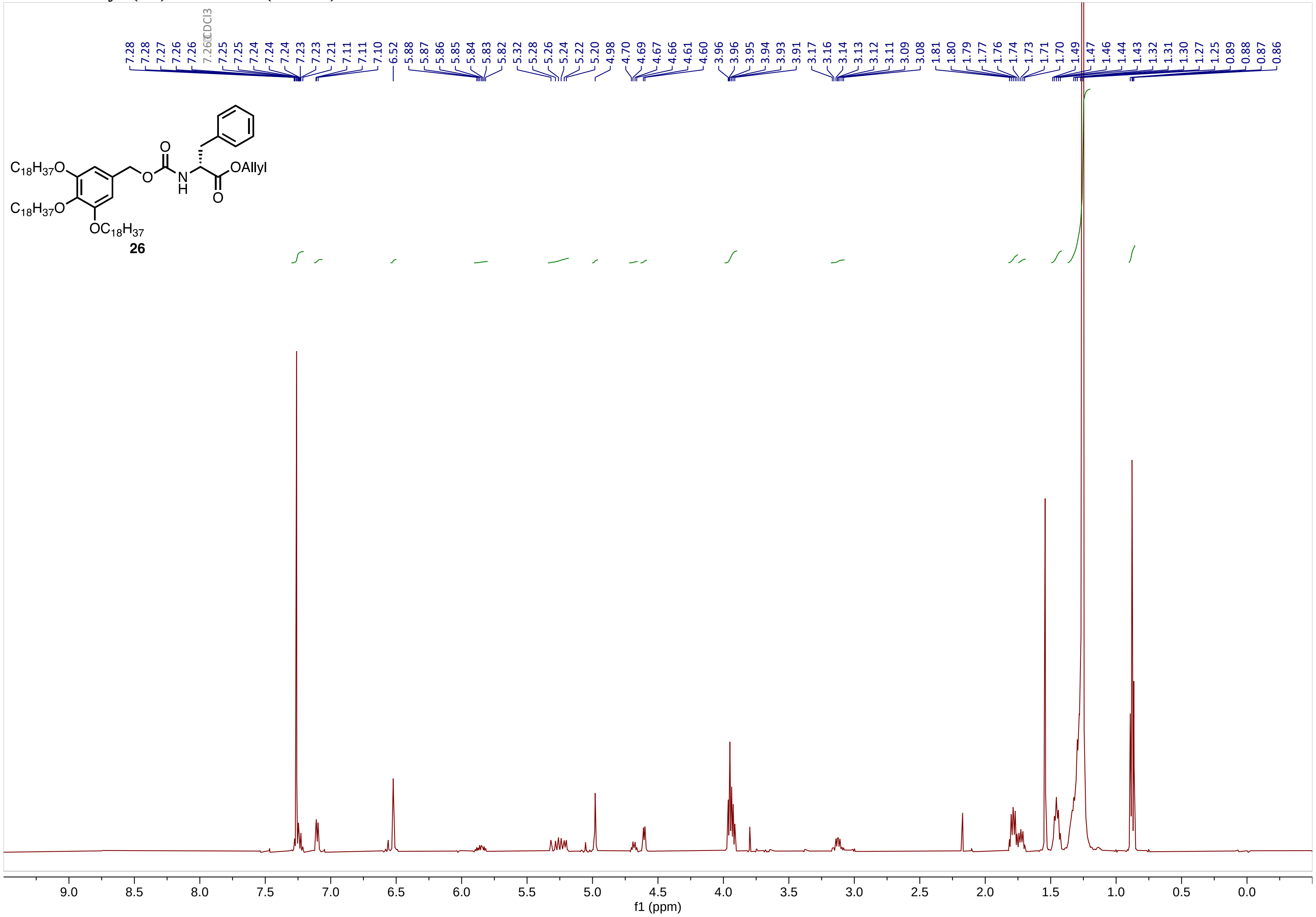

• TCbz-D-Phe-OAllyl (**26**): ^{13}C NMR ($CDCl_3$)

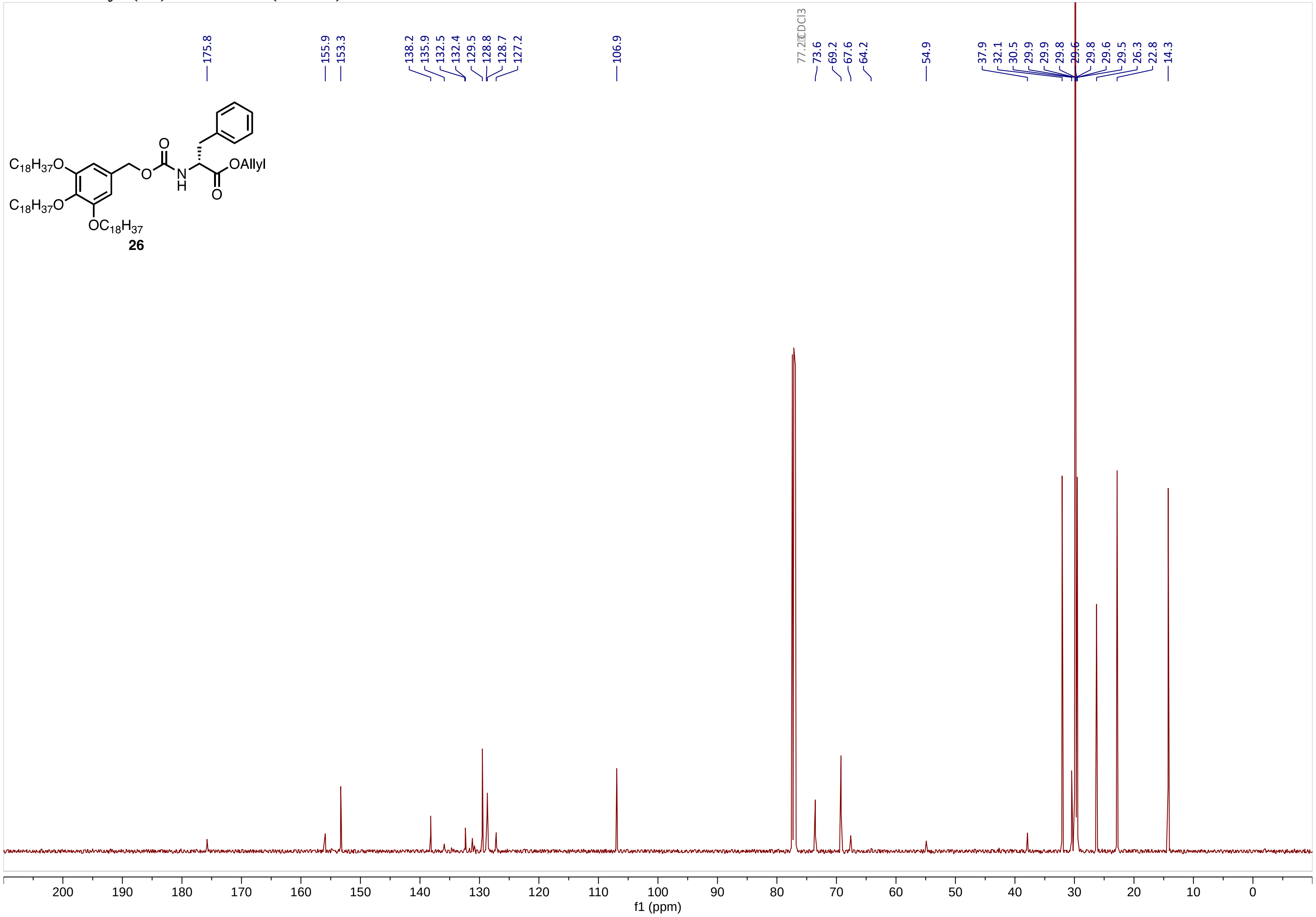

• TAGa-H (**S1**): ^{1}H NMR ($CDCl_3$)

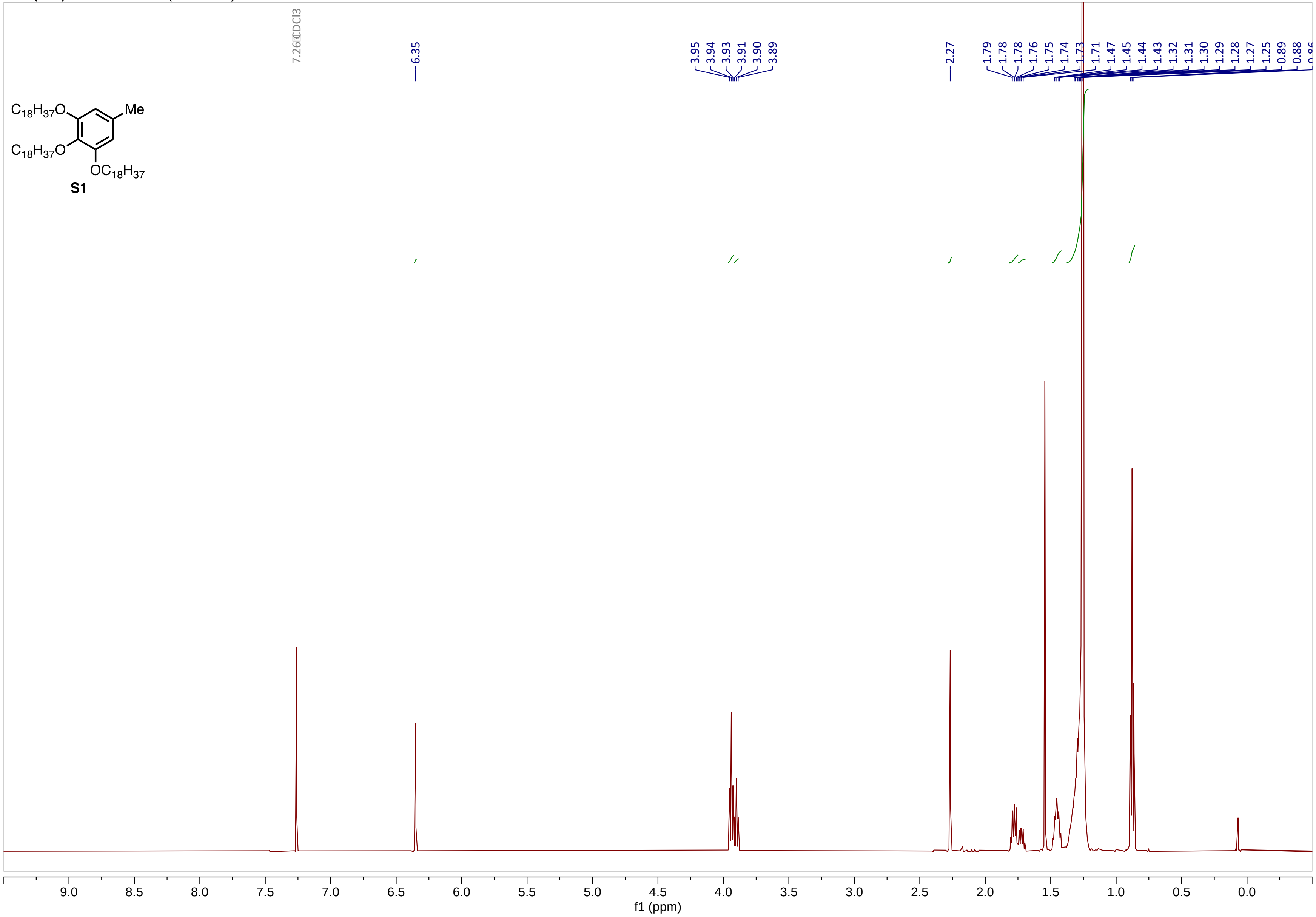

• TAG-H (**S1**): ^{13}C NMR ($CDCl_3$)

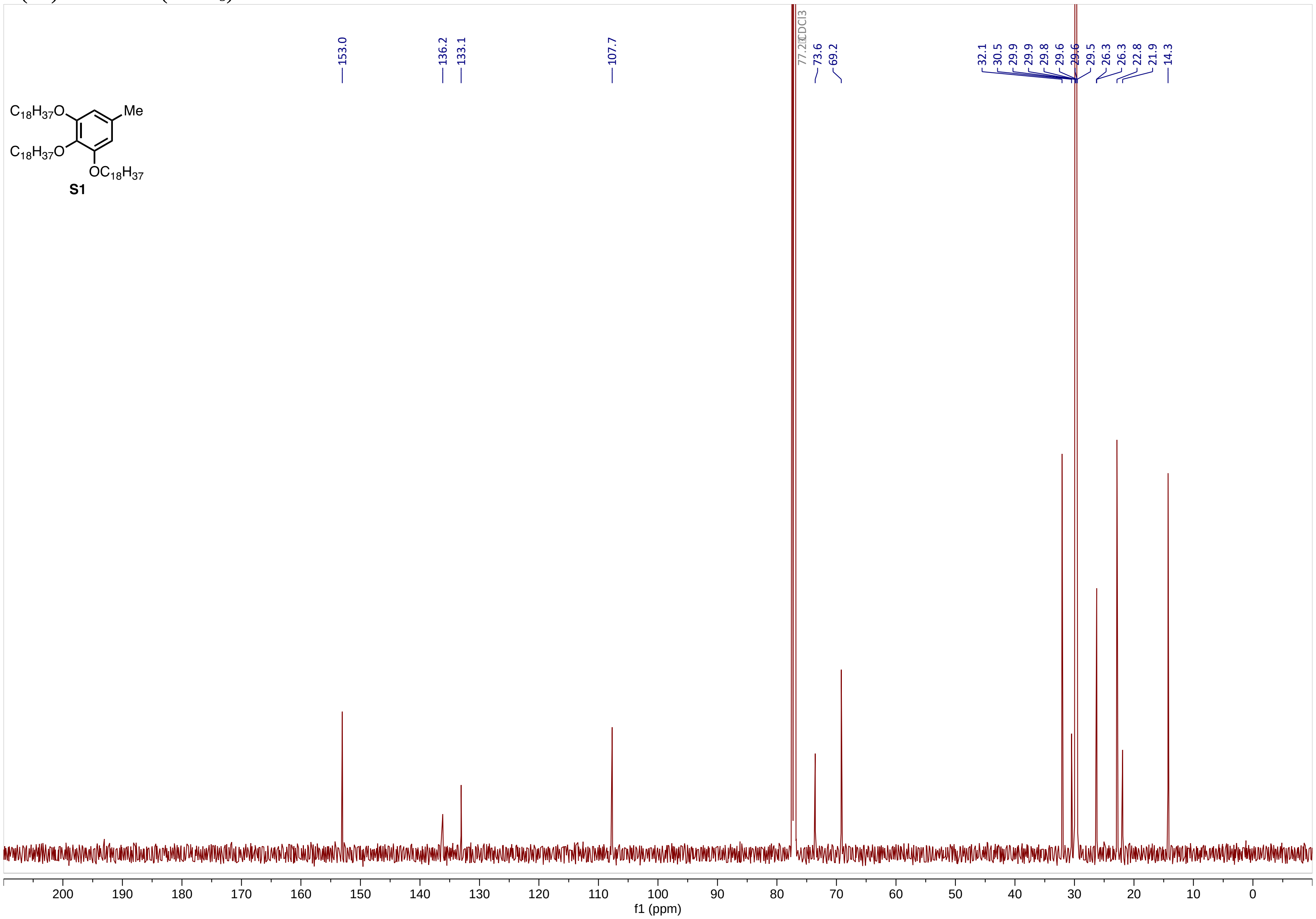

• Orn(TCbz)-OMe (**28**): ^{1}H NMR ($CDCl_3$)

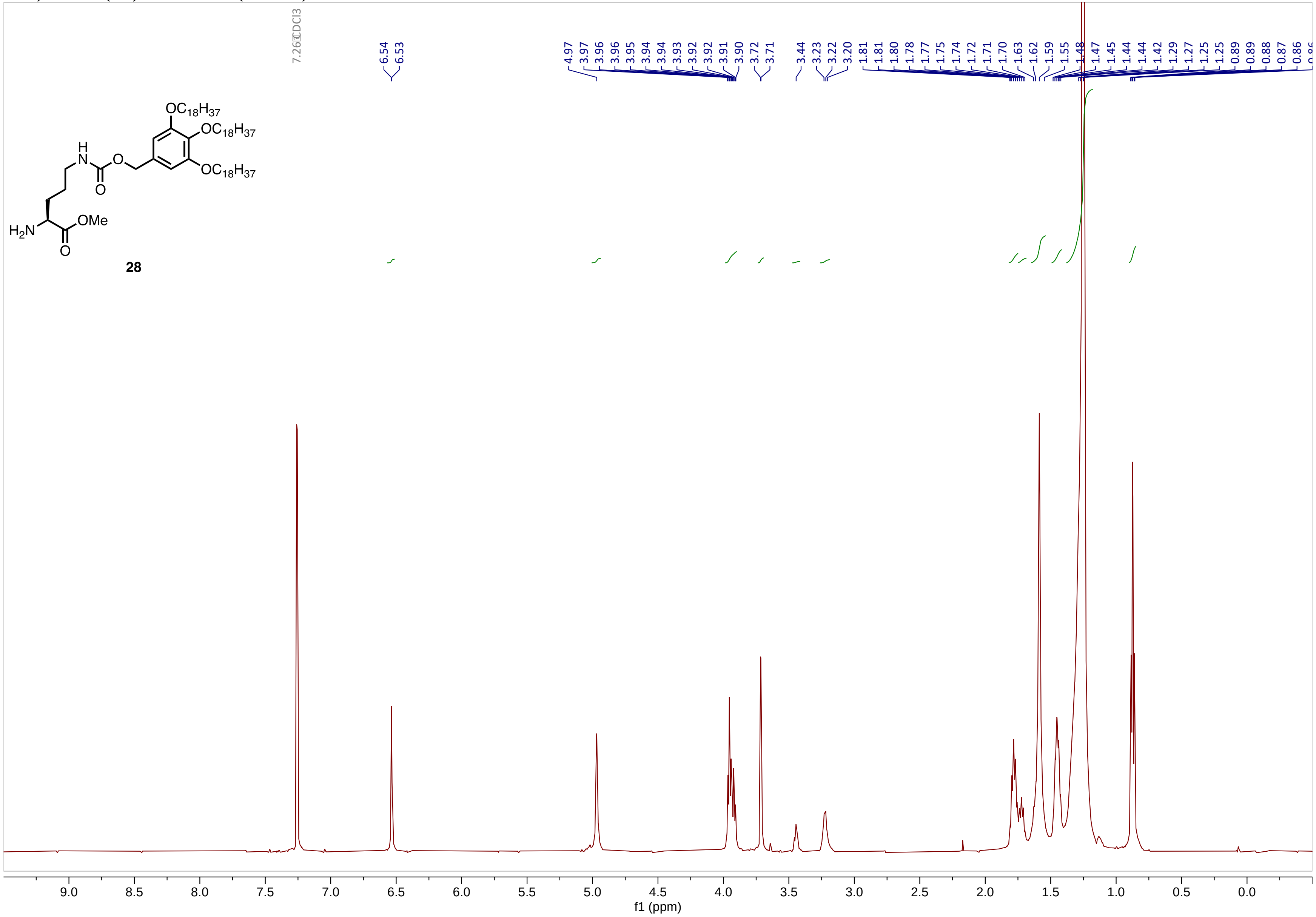

• Orn(TCbz)-OMe (**28**): ^{13}C NMR ($CDCl_3$)

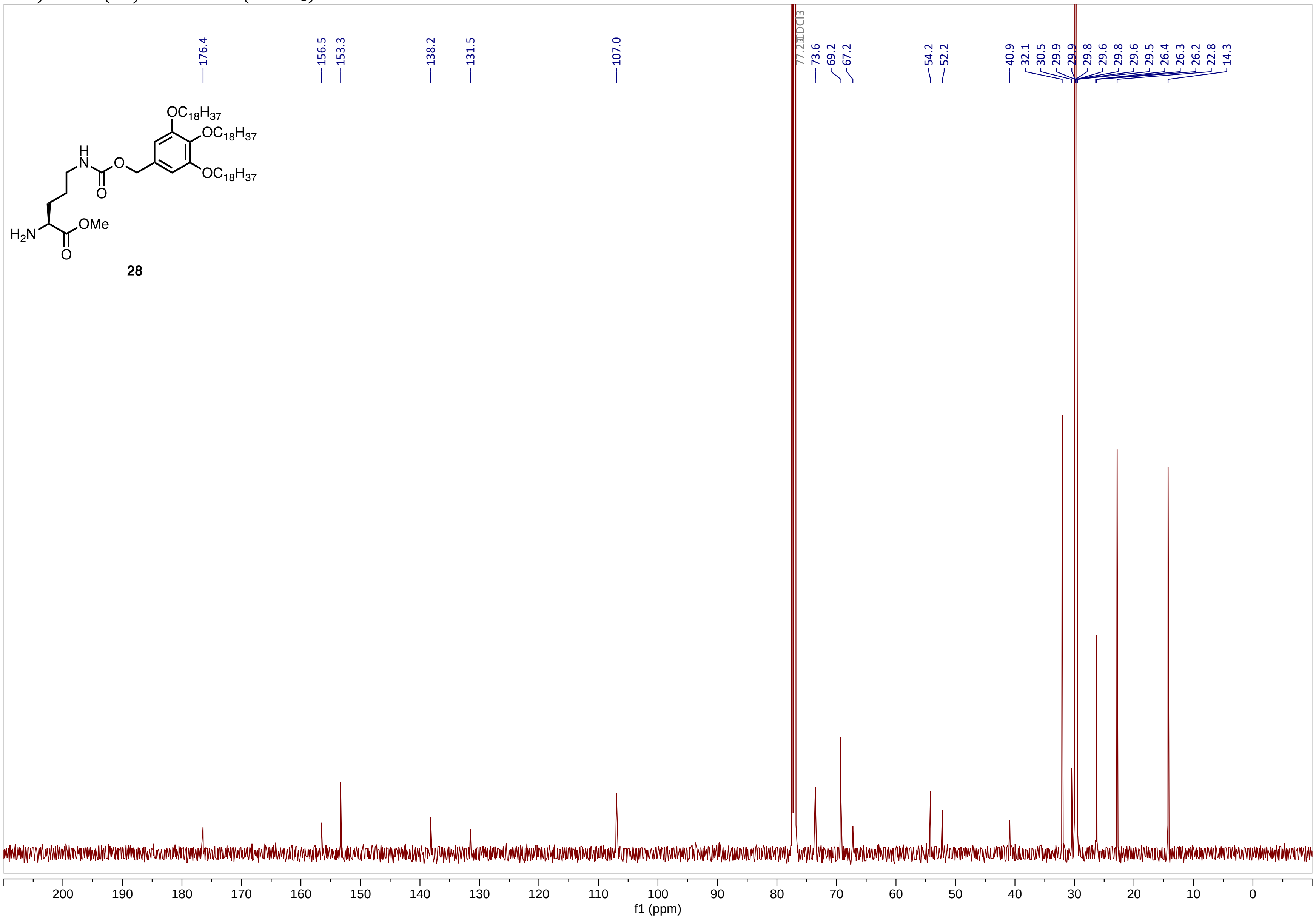

- Lys(TCbz)-OMe (**29**): ^{1}H NMR ($CDCl_3$)

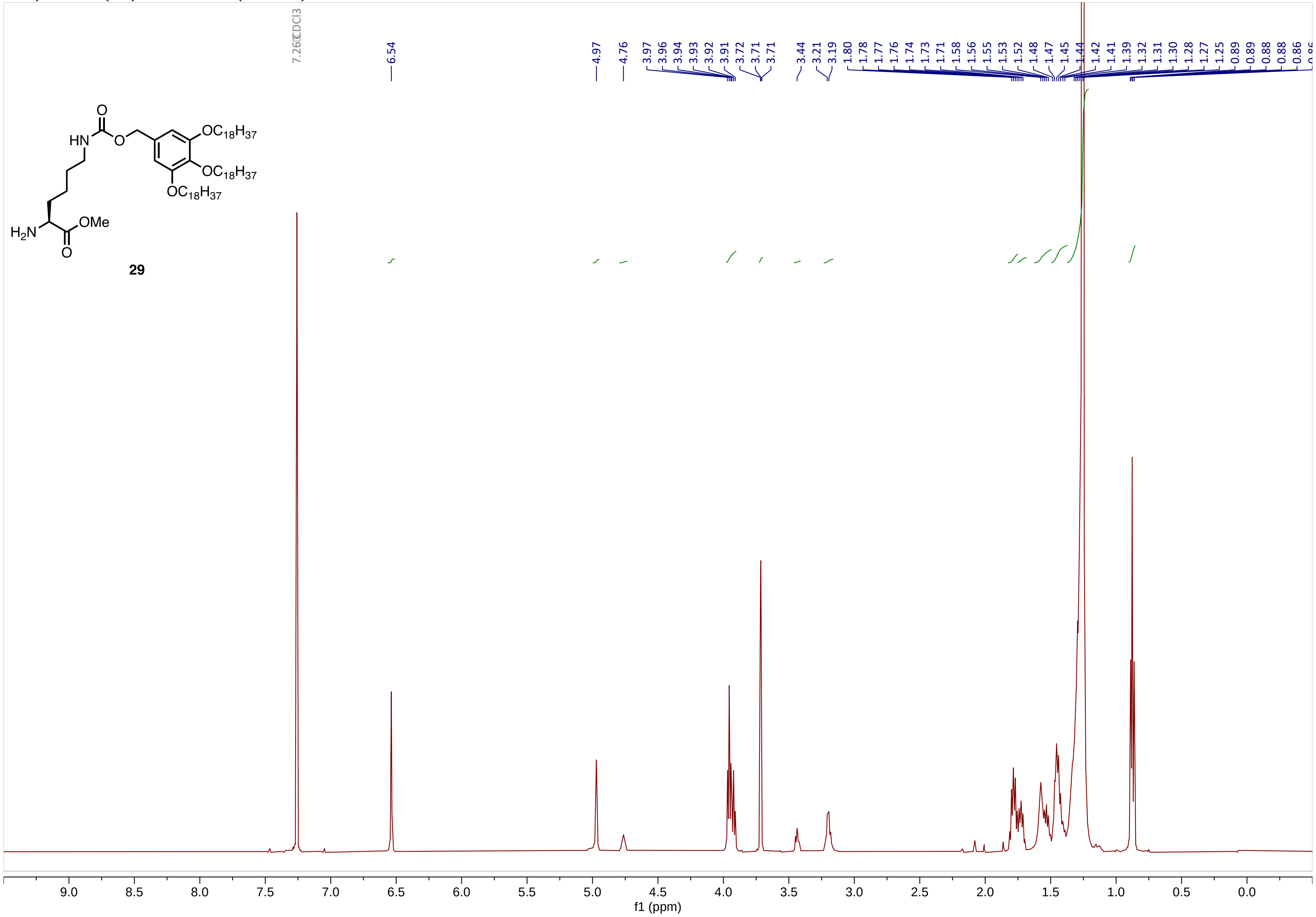

• Lys(TCbz)-OMe (**29**): ^{13}C NMR ($CDCl_3$)

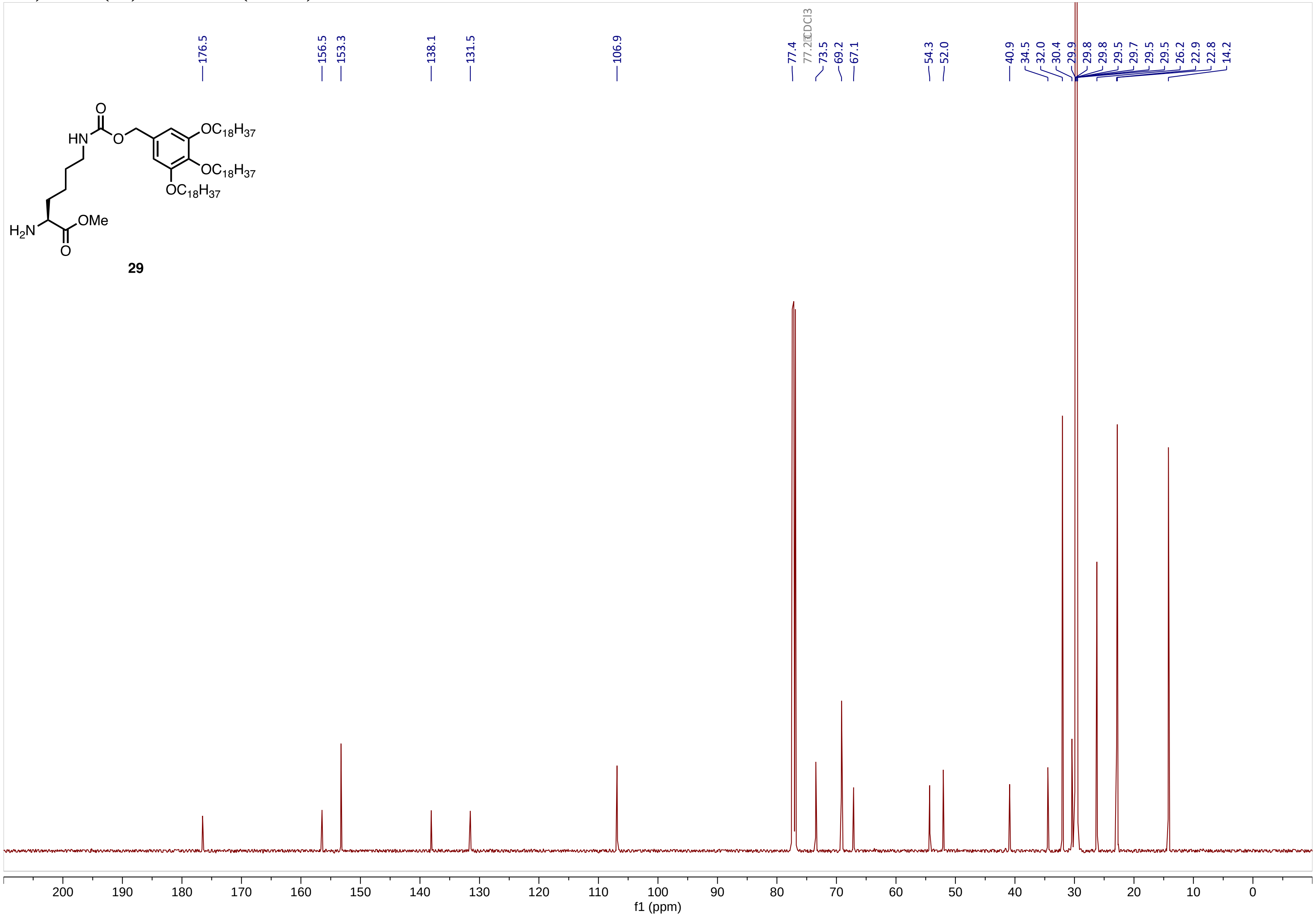

• TCbz-Trp-OH (**30**): ^{1}H NMR ($CDCl_3$)

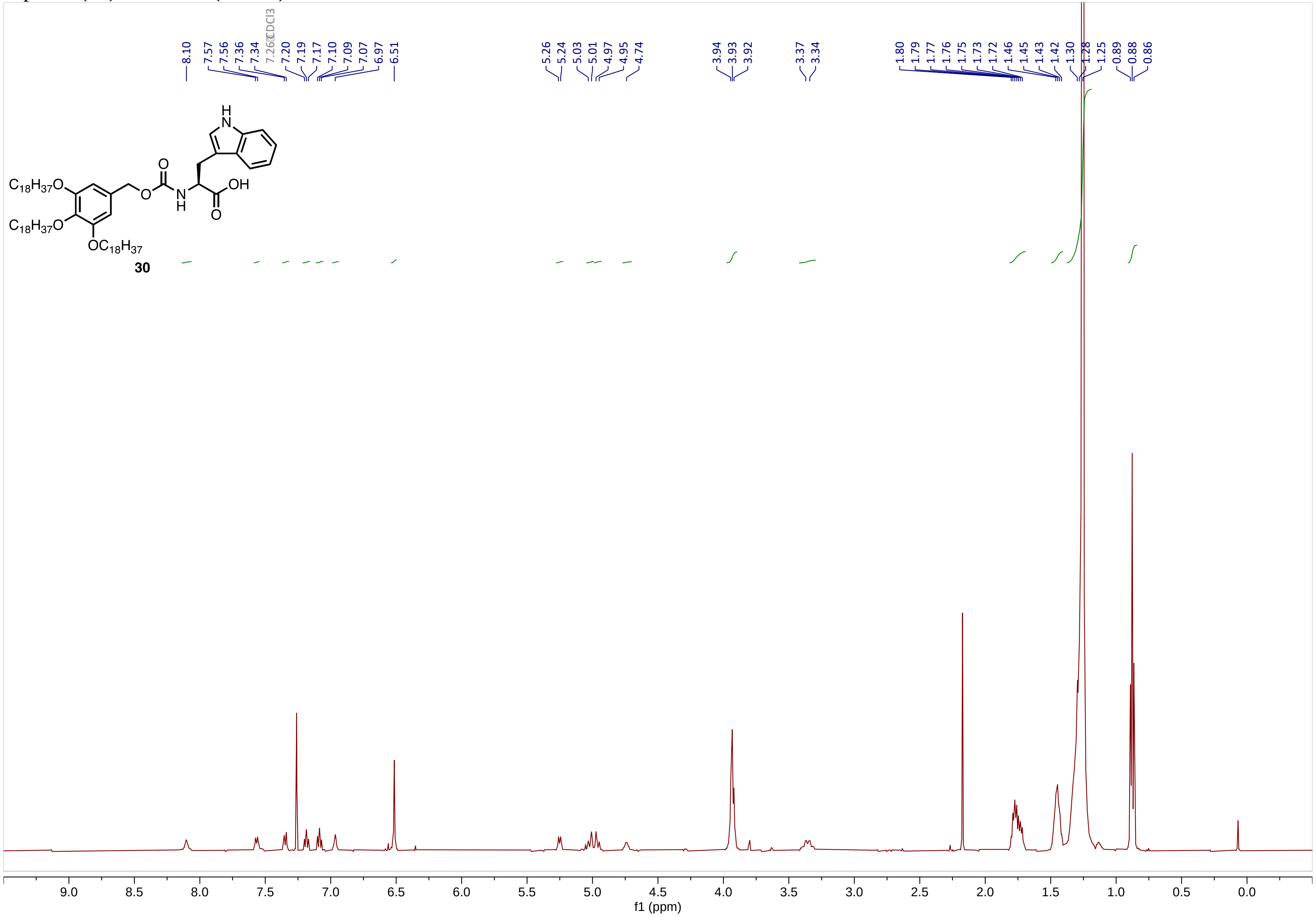

• TCbz-Trp-OH (**30**): ^{13}C NMR ($CDCl_3$)

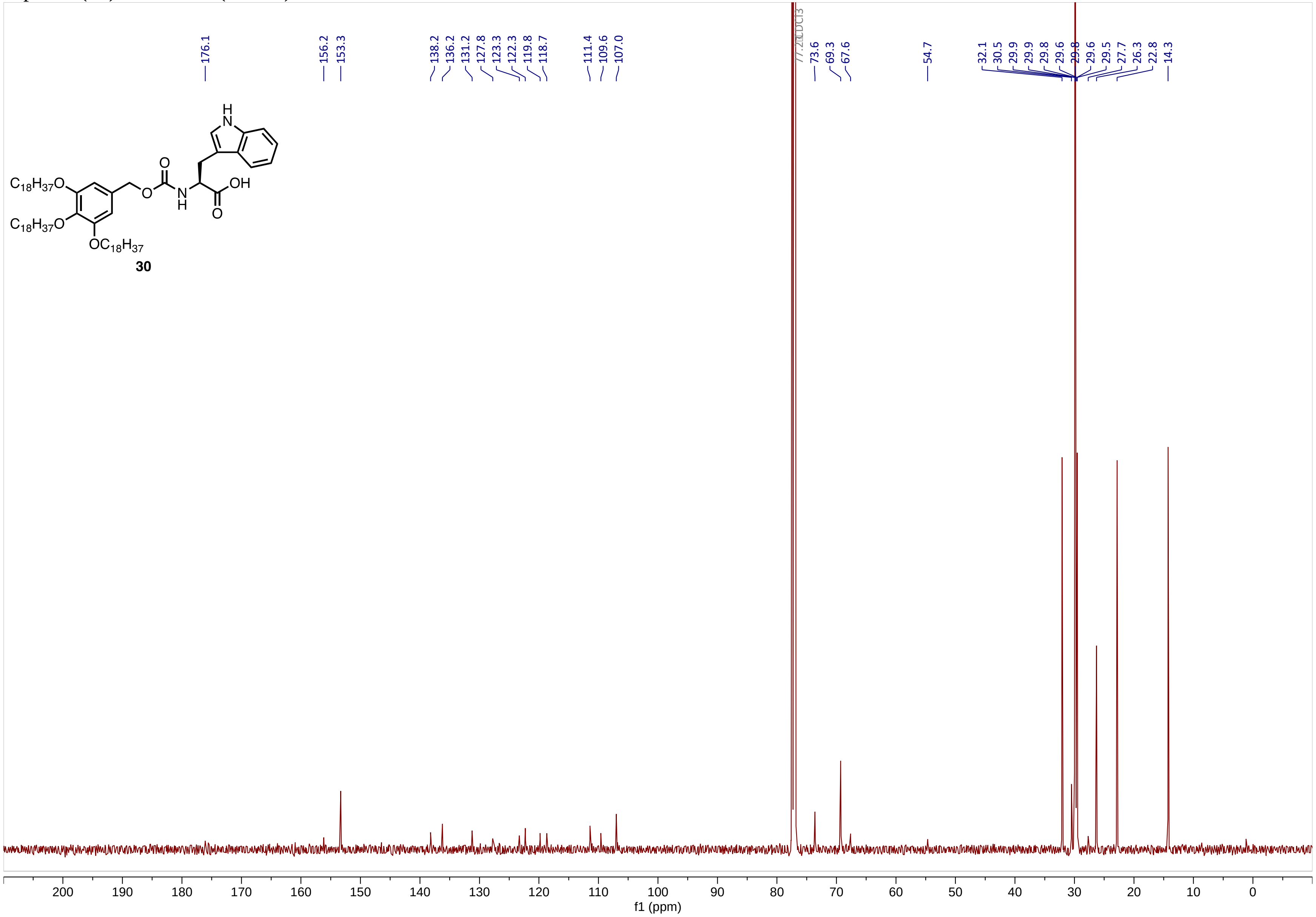

• TCbz-Ile-OH (**31**): ^{1}H NMR ($CDCl_3$)

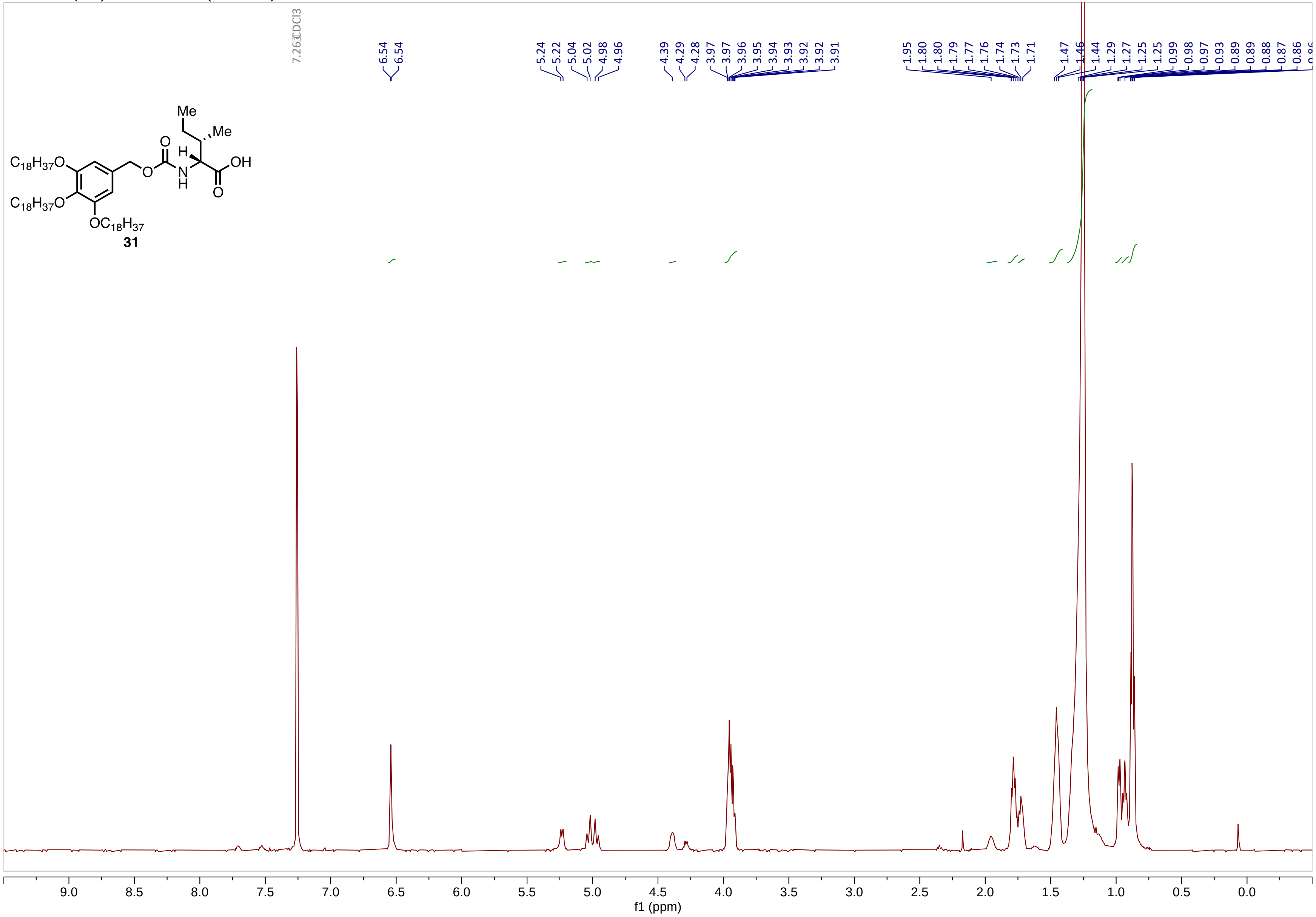

• TCbz-Ile-OH (**31**): ^{13}C NMR ($CDCl_3$)

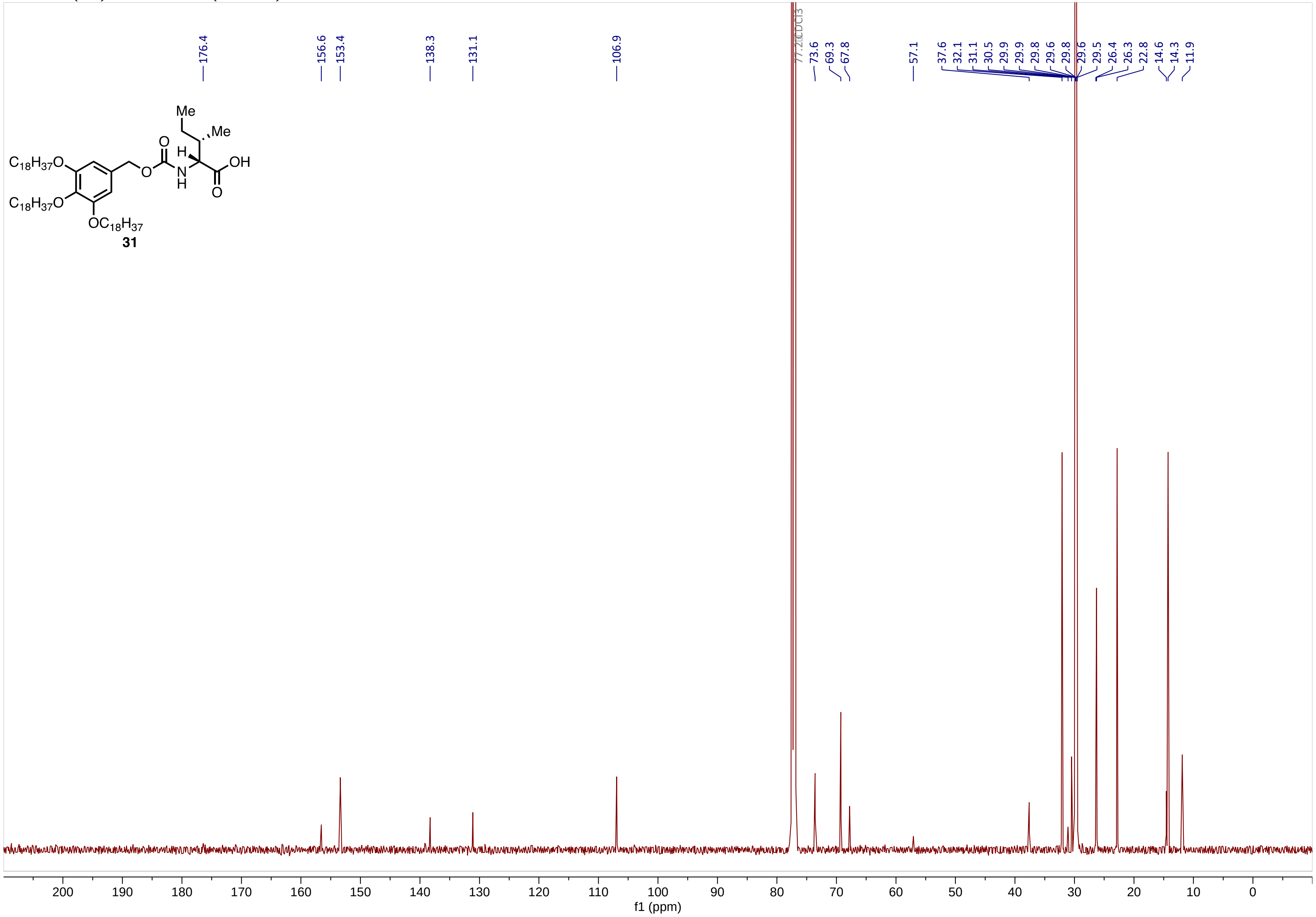

• Fmoc-Orn(TCbz)-OH (**32**): ^{1}H NMR ($CDCl_3$)

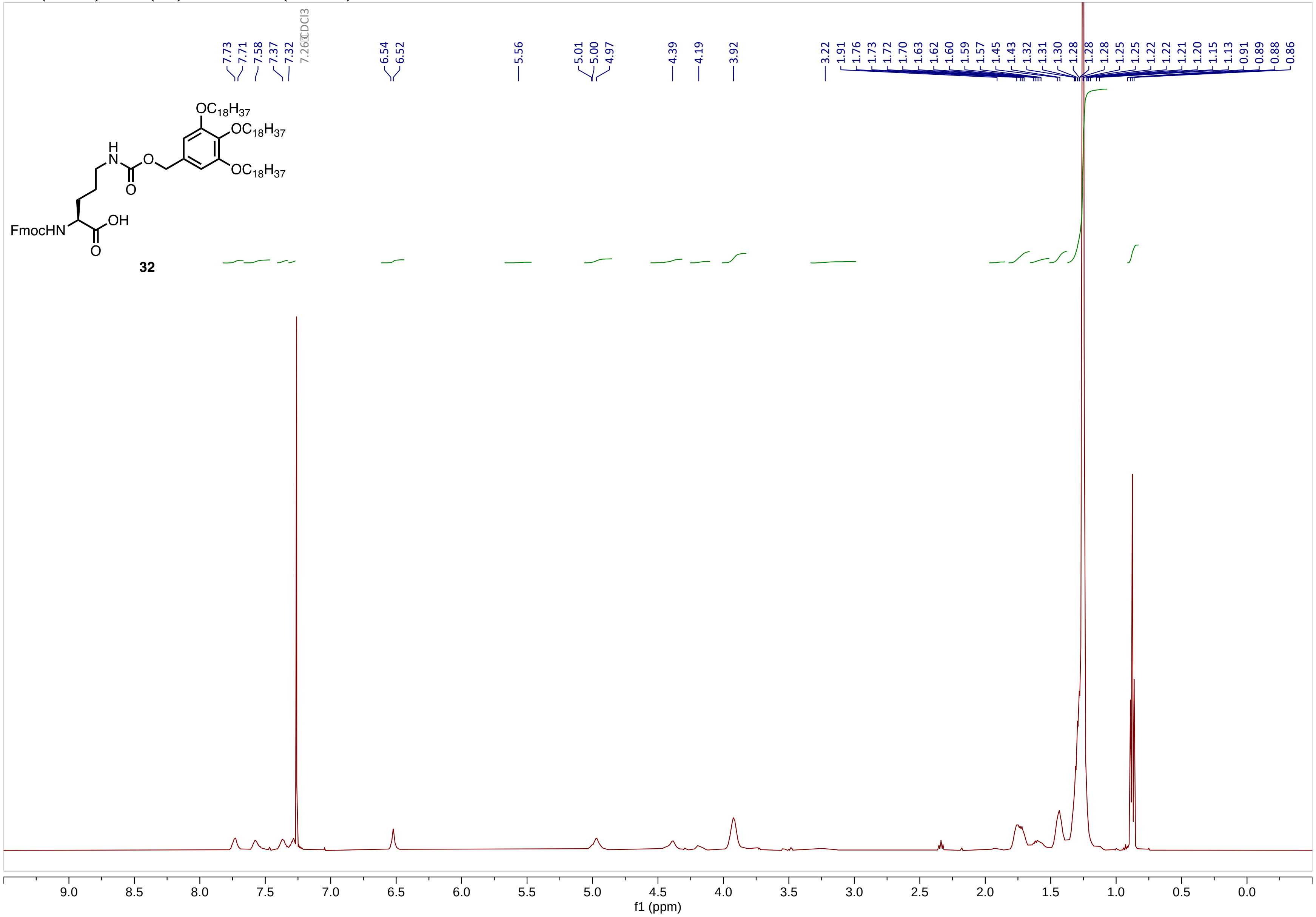

• Fmoc-Orn(TCbz)-OH (**32**): ^{13}C NMR ($CDCl_3$)

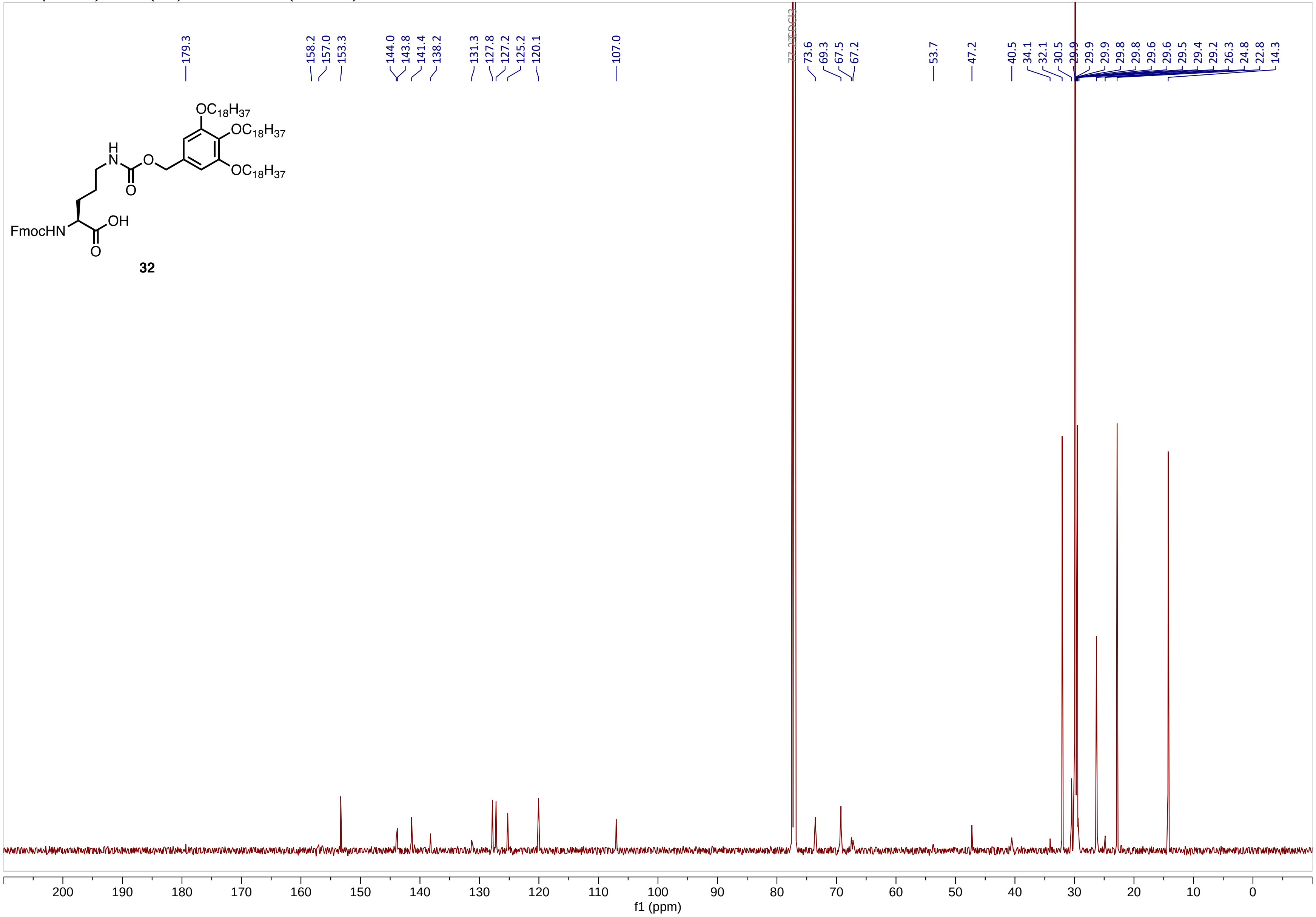

• TCbz-D-Phe-OH (**33**): ^{1}H NMR ($CDCl_3$)

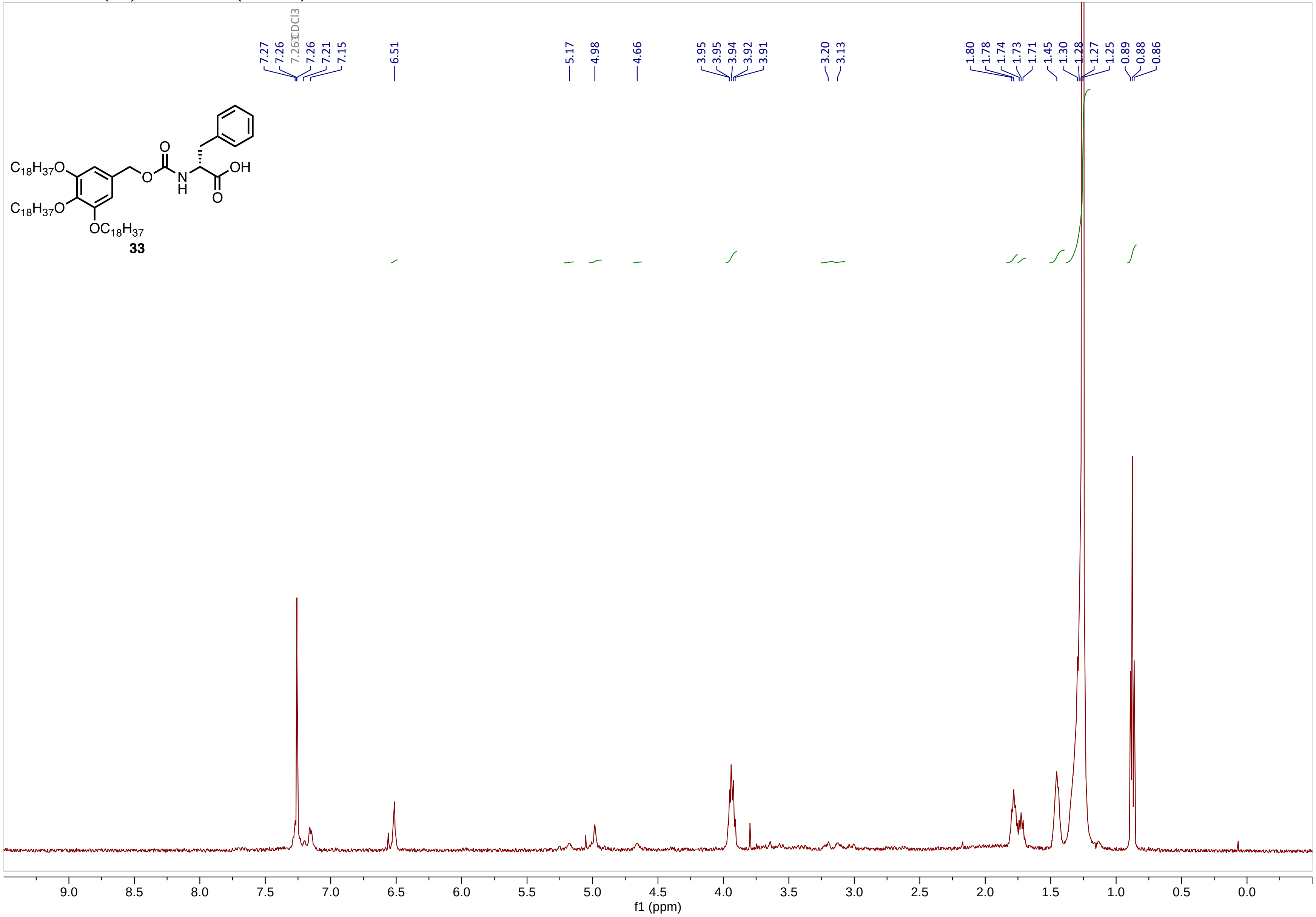

- TCbz-D-Phe-OH (**33**): ^{13}C NMR ($CDCl_3$)

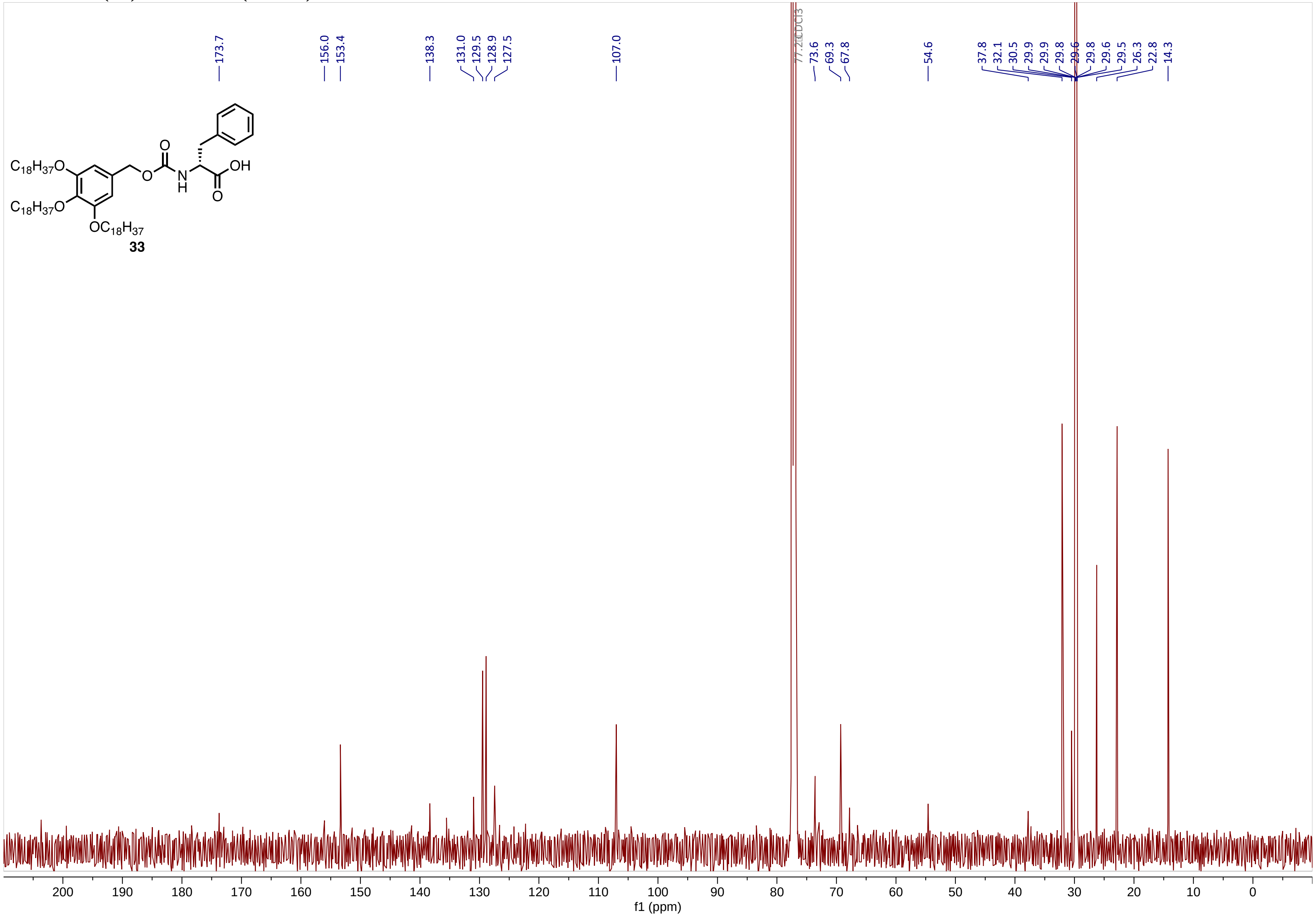

• Depsipeptide fragment (**S2**): ^{1}H NMR ($CDCl_3$)

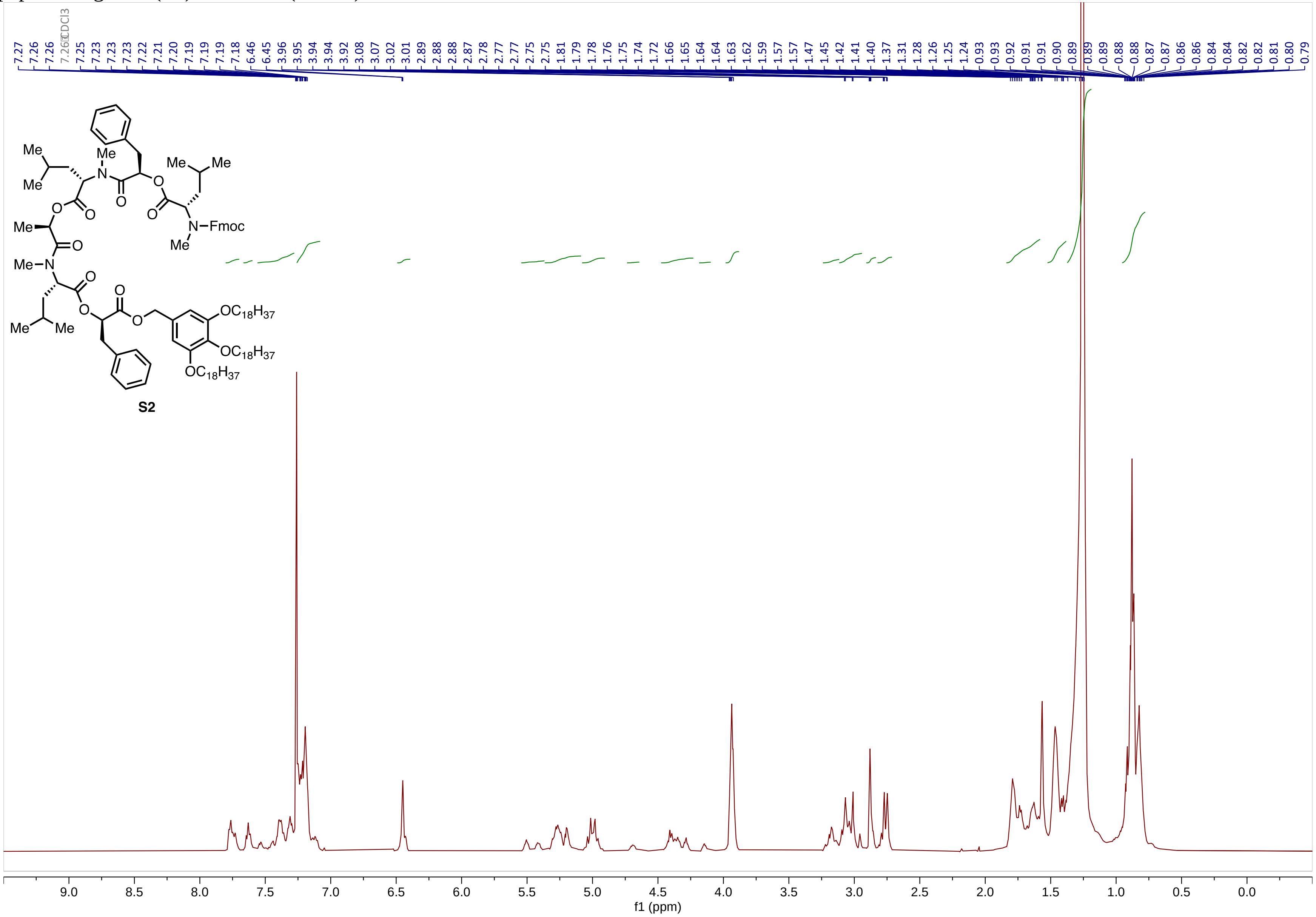

• Depsipeptide fragment (**S2**): ^{13}C NMR ($CDCl_3$)

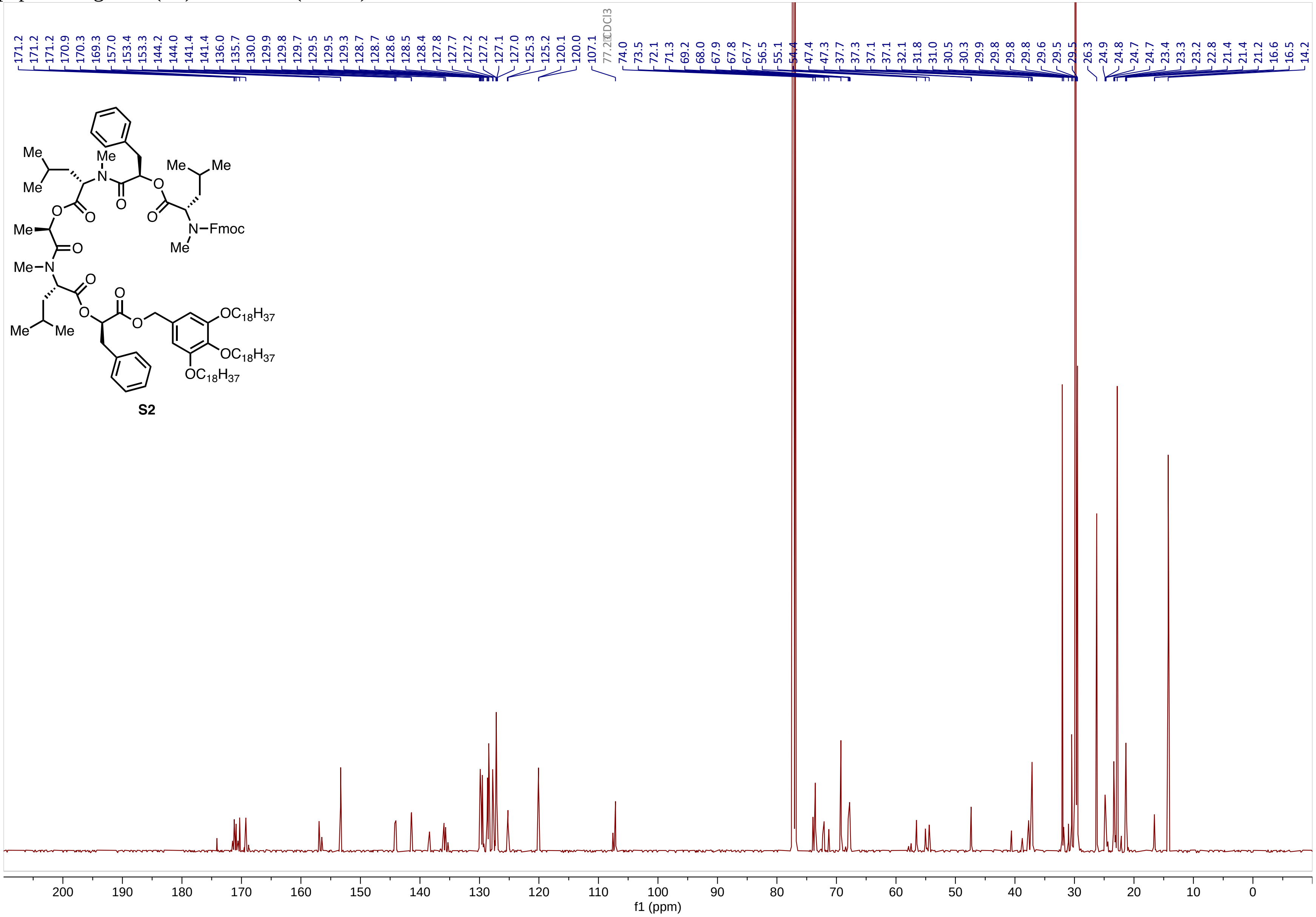

• TCbz-oligodepsipeptide (**34**): ^{1}H NMR ($CDCl_3$)

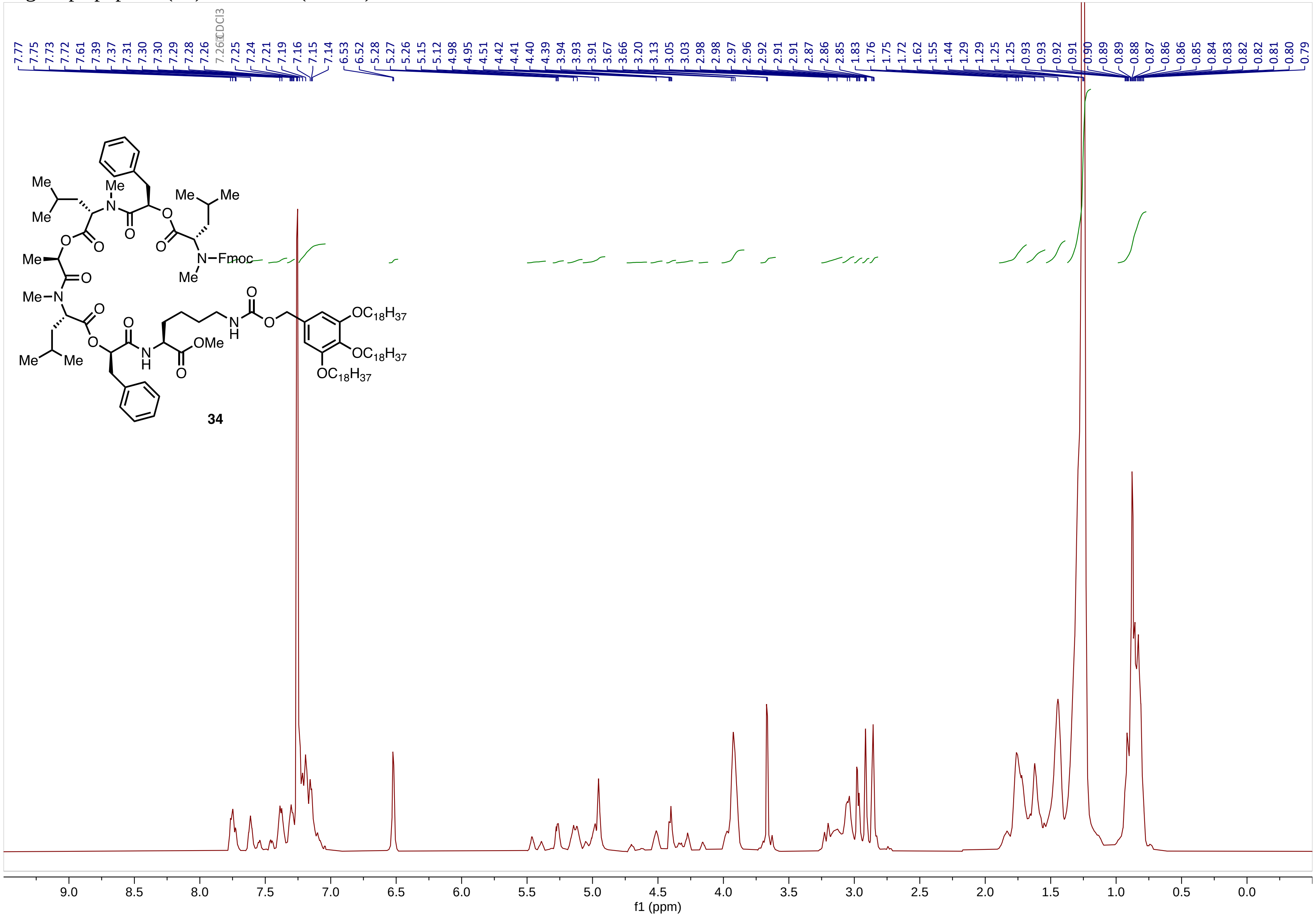

• TCbz-oligodepsipeptide (**34**): ^{13}C NMR ($CDCl_3$)

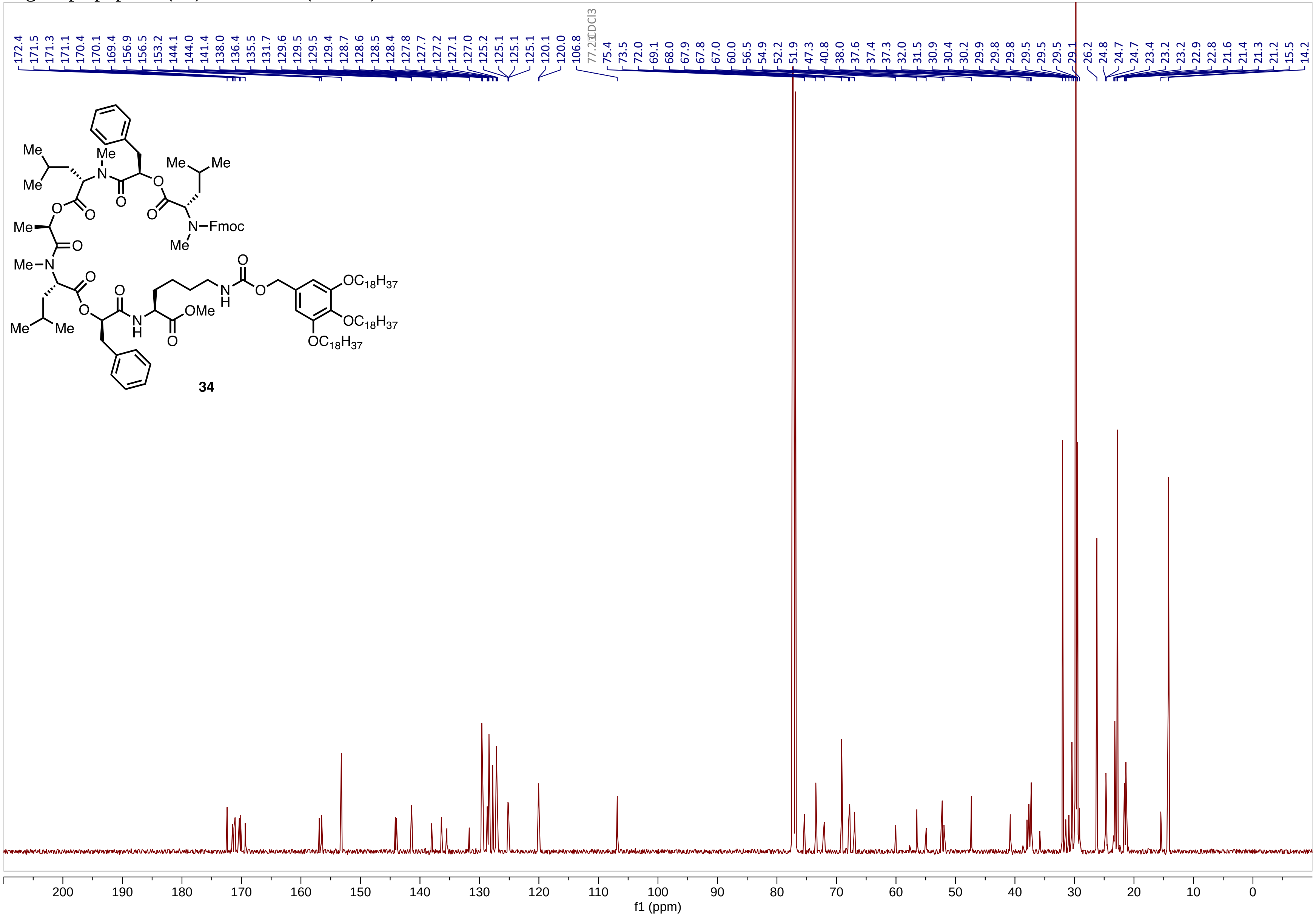

• Pentapeptide-NH$_2$ (**S5**): ^{1}H NMR (CD$_3$OD)

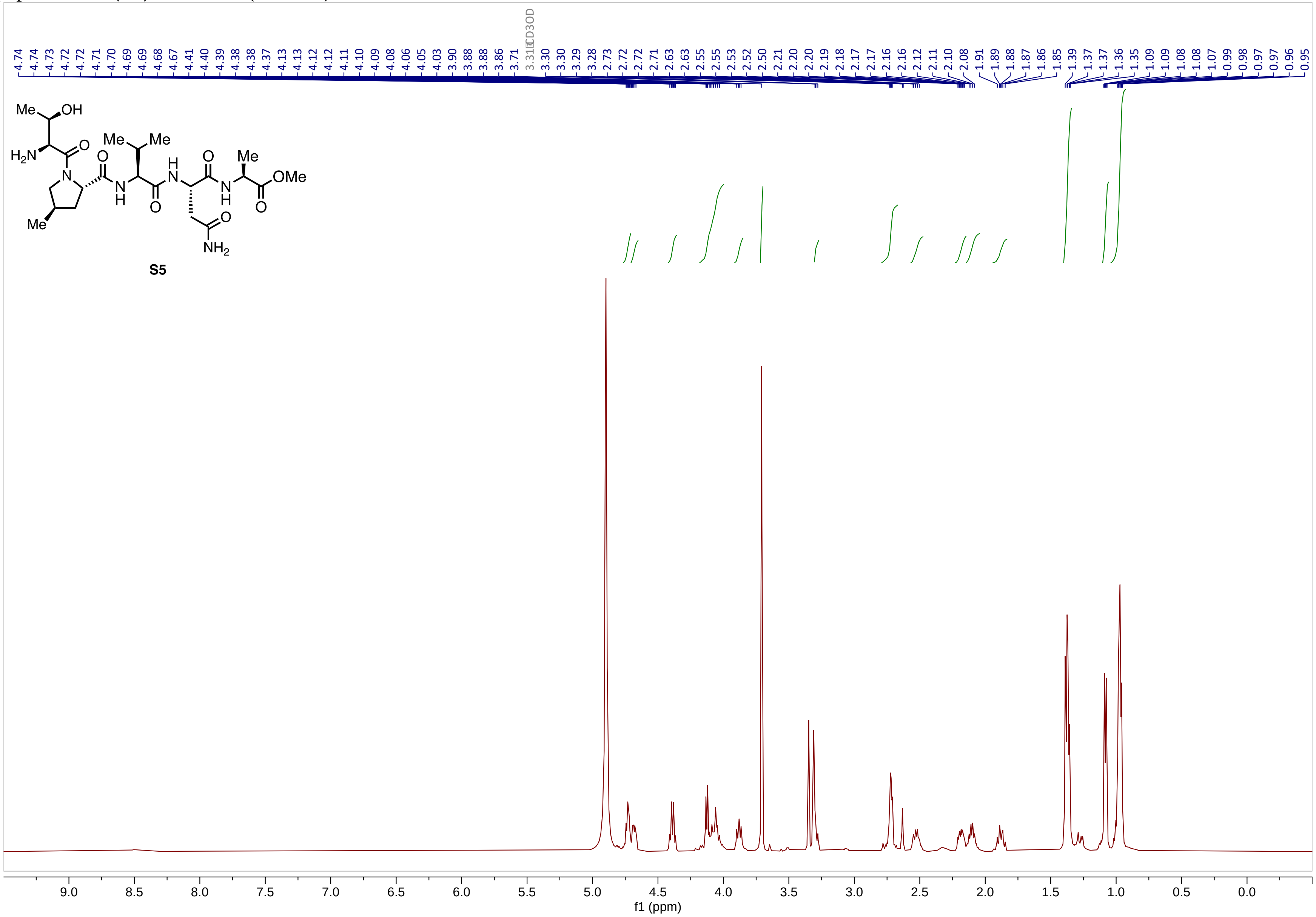

• Pentapeptide-NH_2 (**S5**): ^{13}C NMR (CD_3OD)

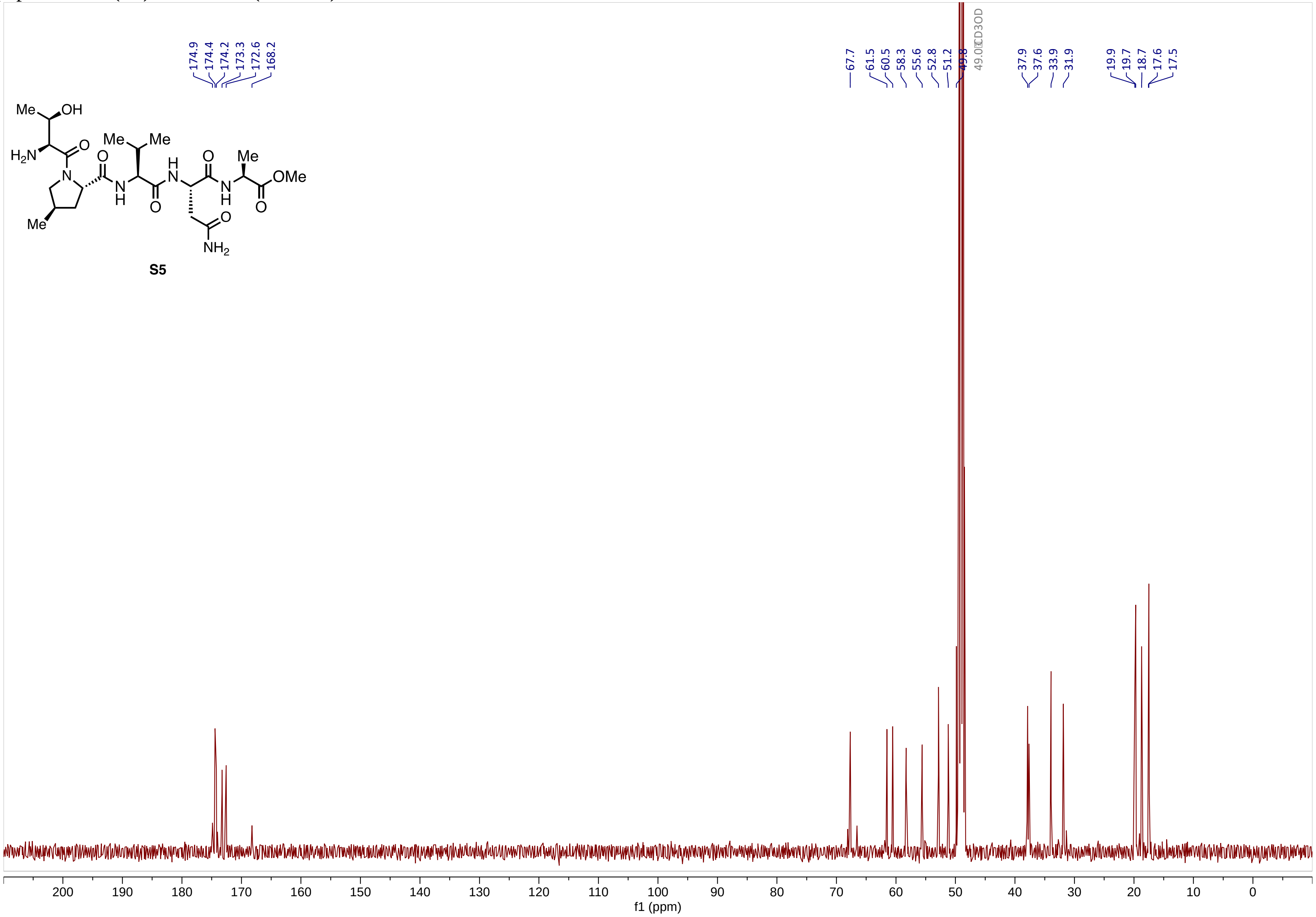

• TCbz-oligopeptide (**35**): ^{1}H NMR ($CDCl_3$)

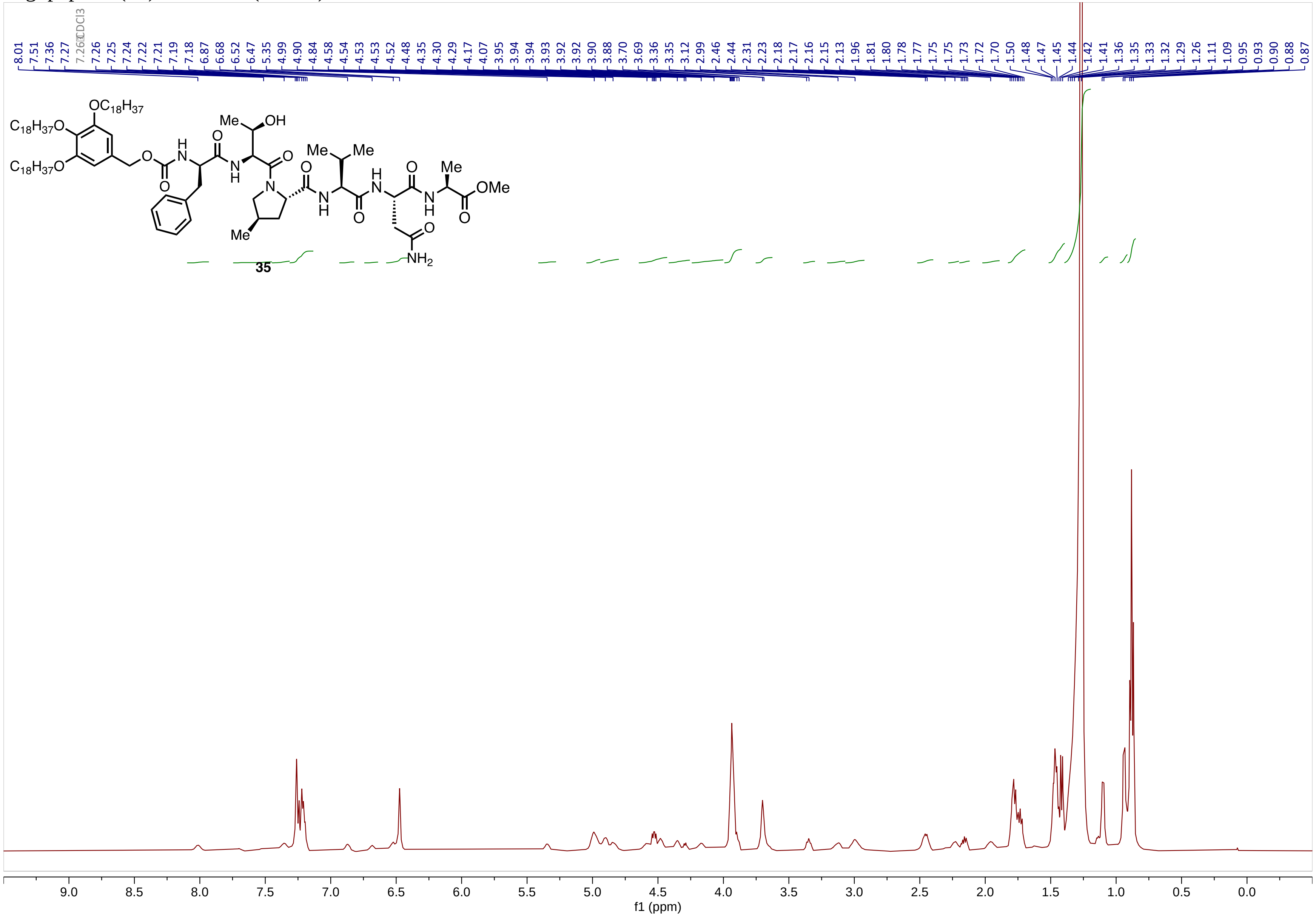

• TCbz-oligopeptide (**35**): ^{13}C NMR ($CDCl_3$)

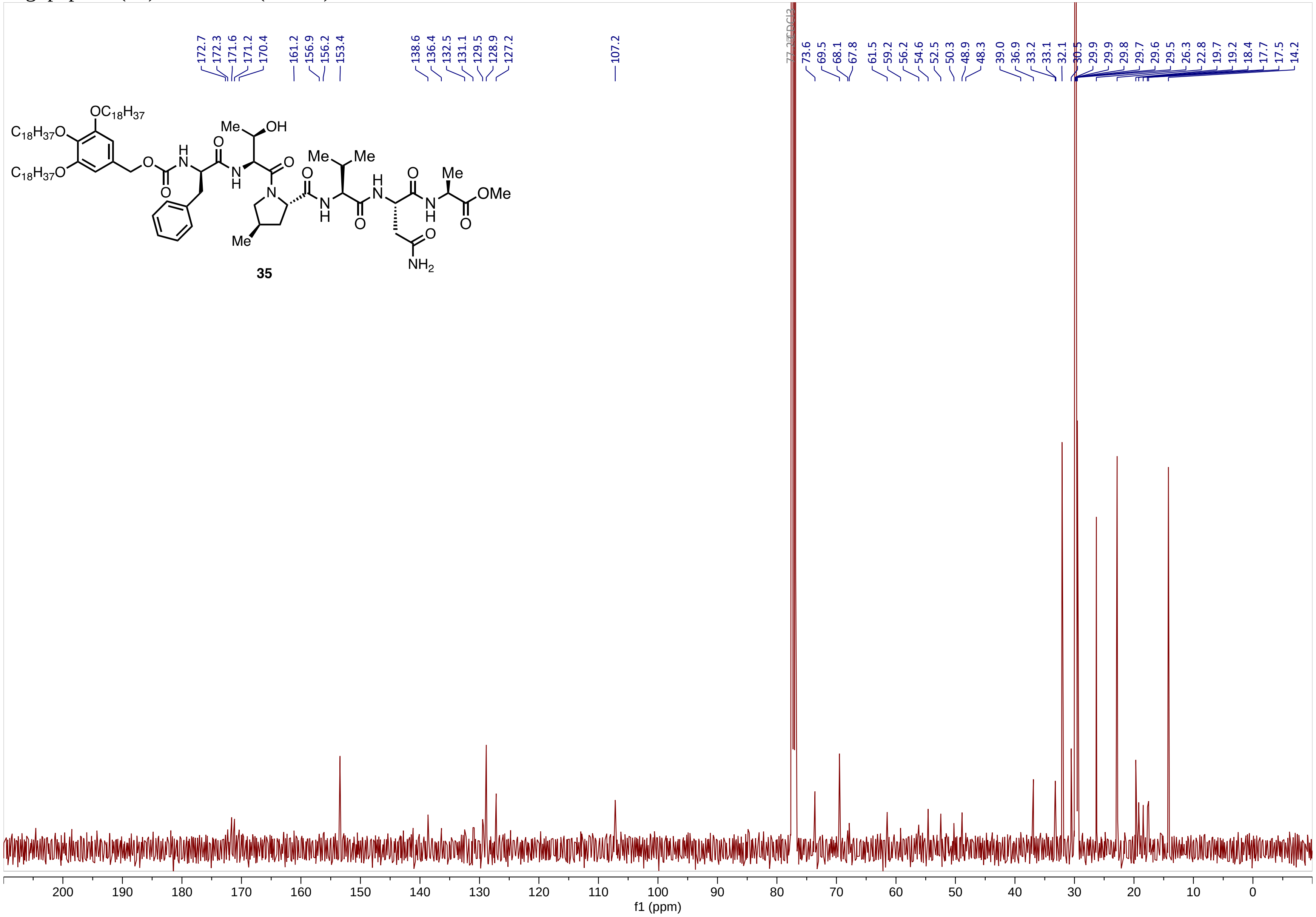

• Bottromycin fragment (**36**): ^{1}H NMR ($CDCl_3$)

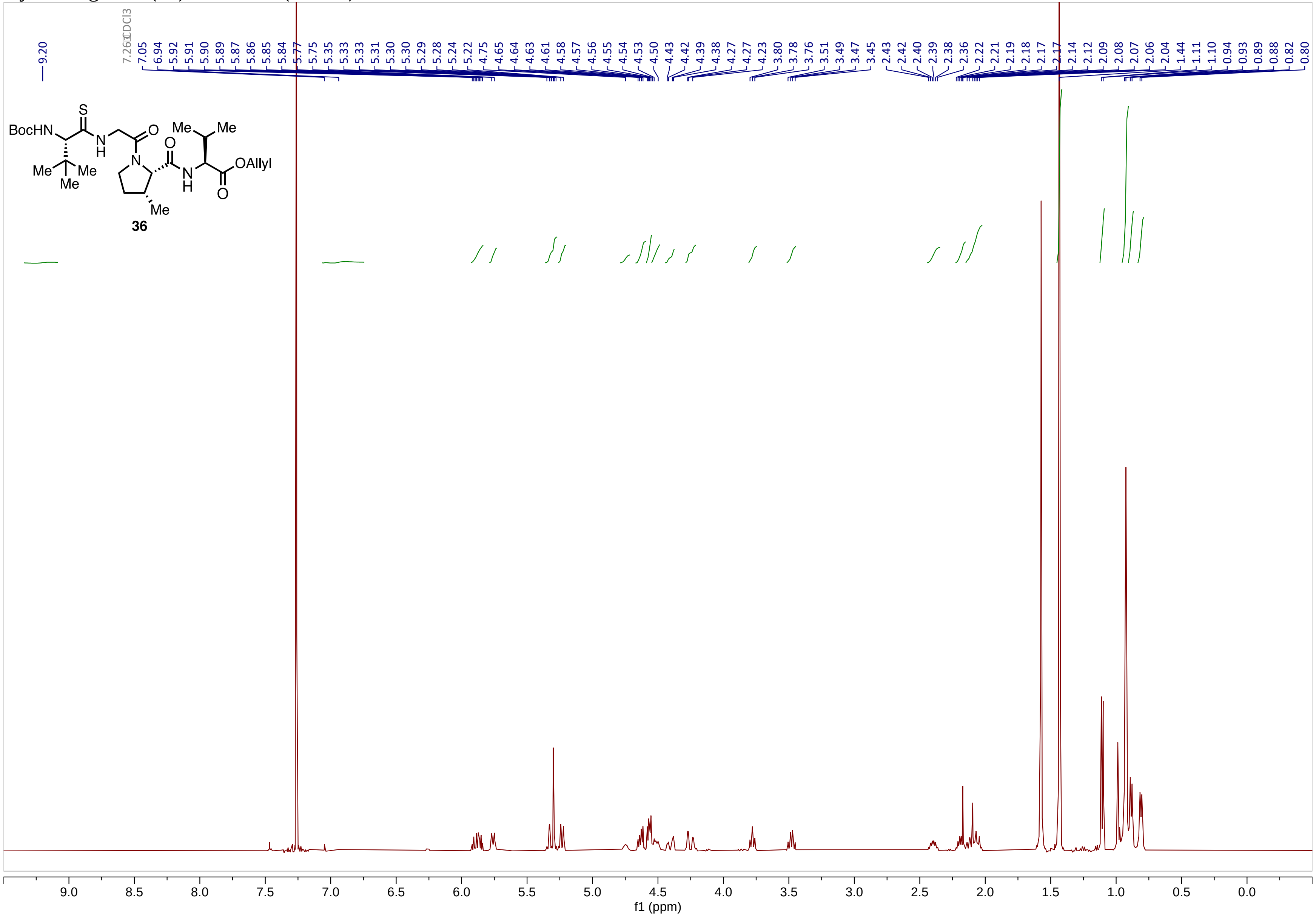

• Bottromycin fragment (**36**): ^{13}C NMR ($CDCl_3$)

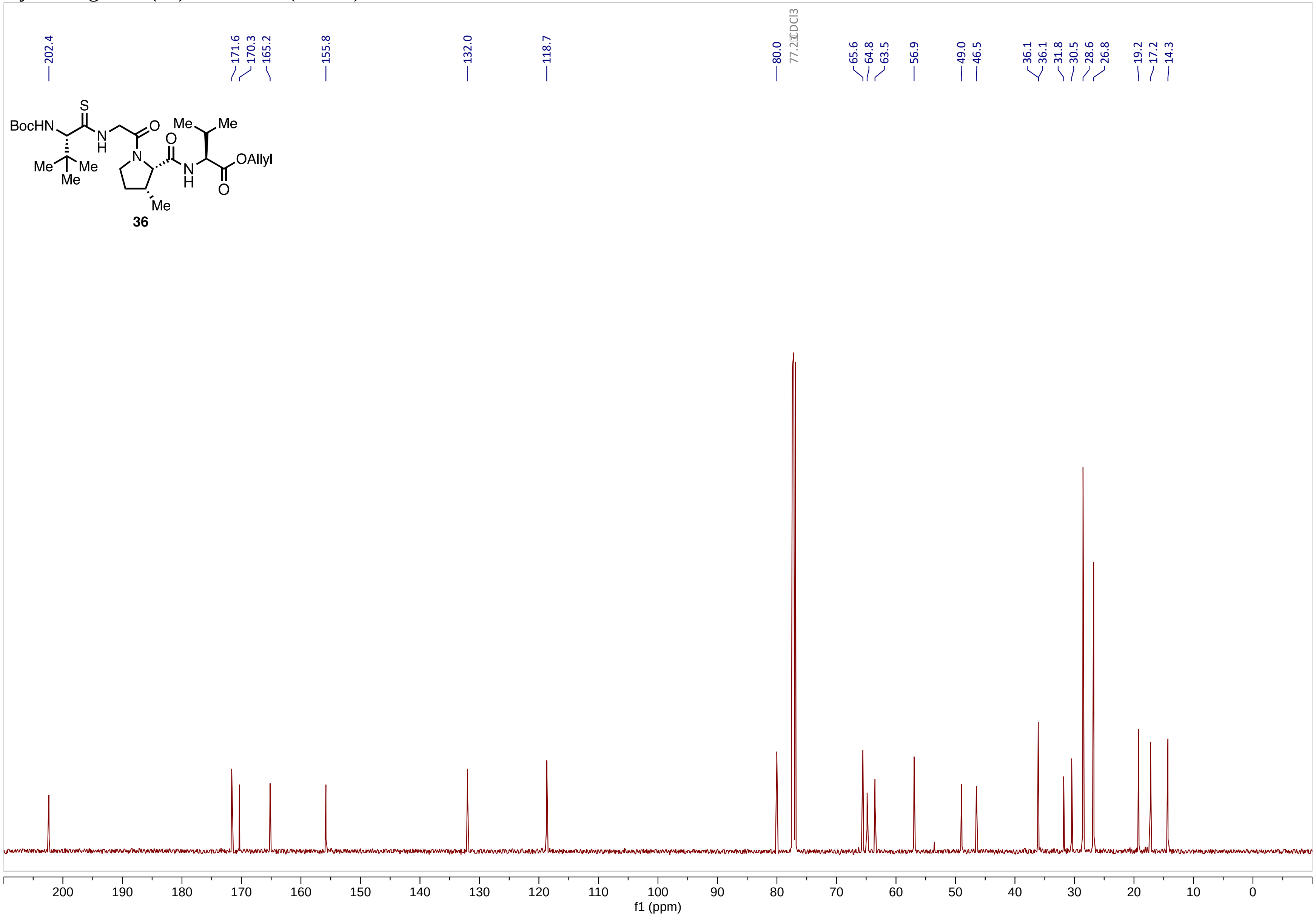

• Tetrapeptide-CO_2H (**S6**): ^{1}H NMR (CD_3OD)

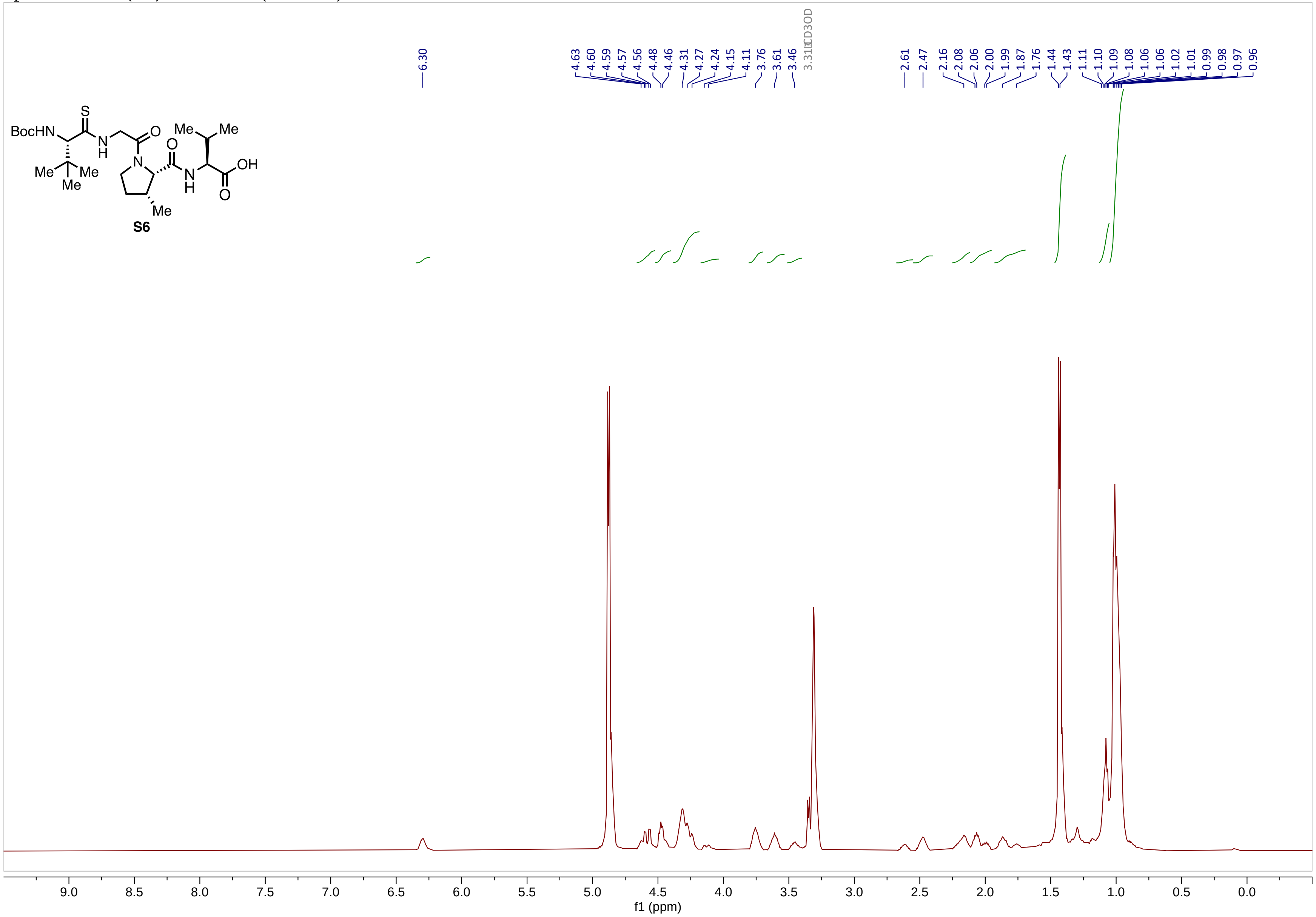

• Tetrapeptide-CO_2H (**S6**): ^{13}C NMR (CD_3OD)

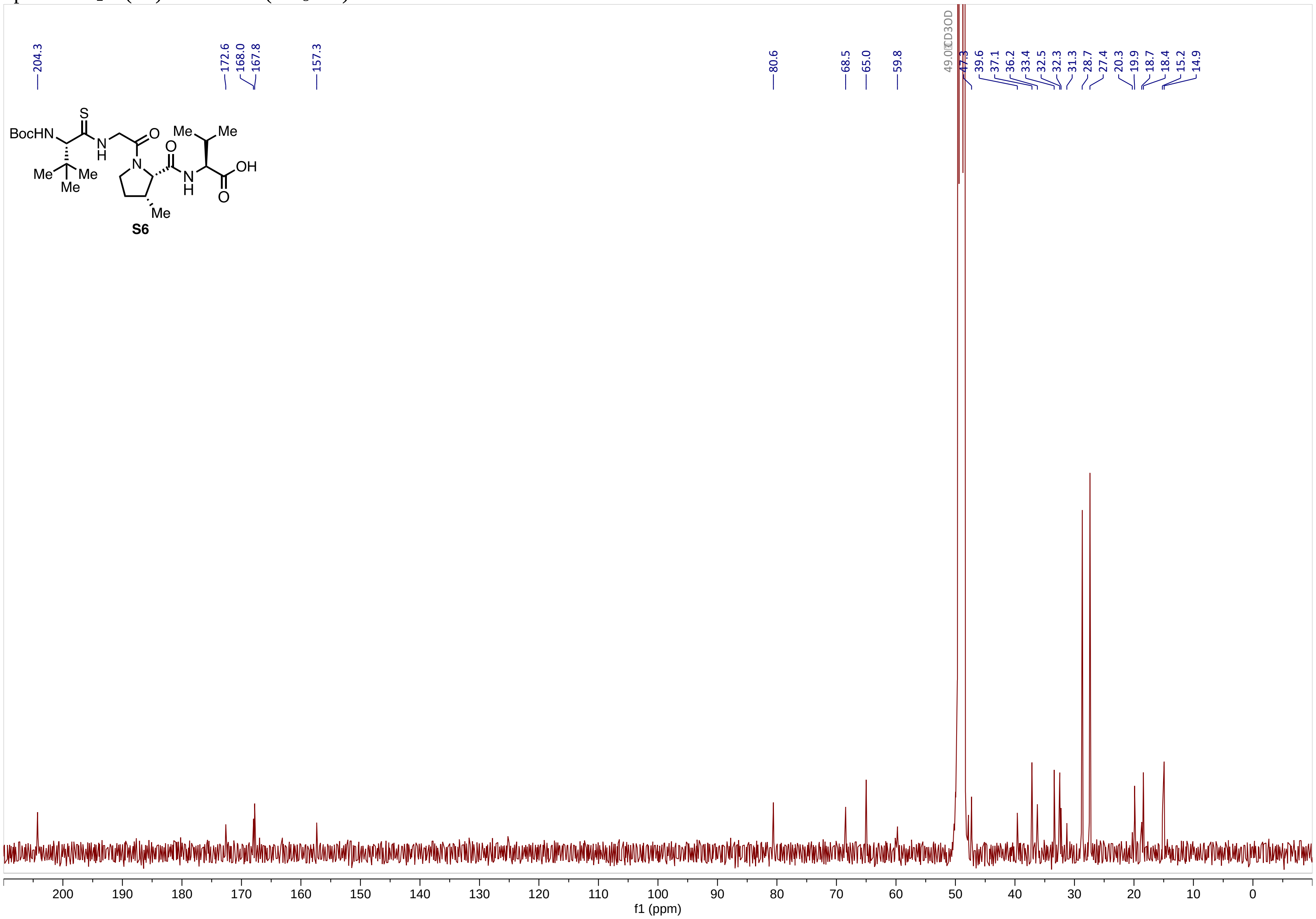

• Tetrapeptide-NH_2 (**S7**): ^{1}H NMR (CD_3OD)

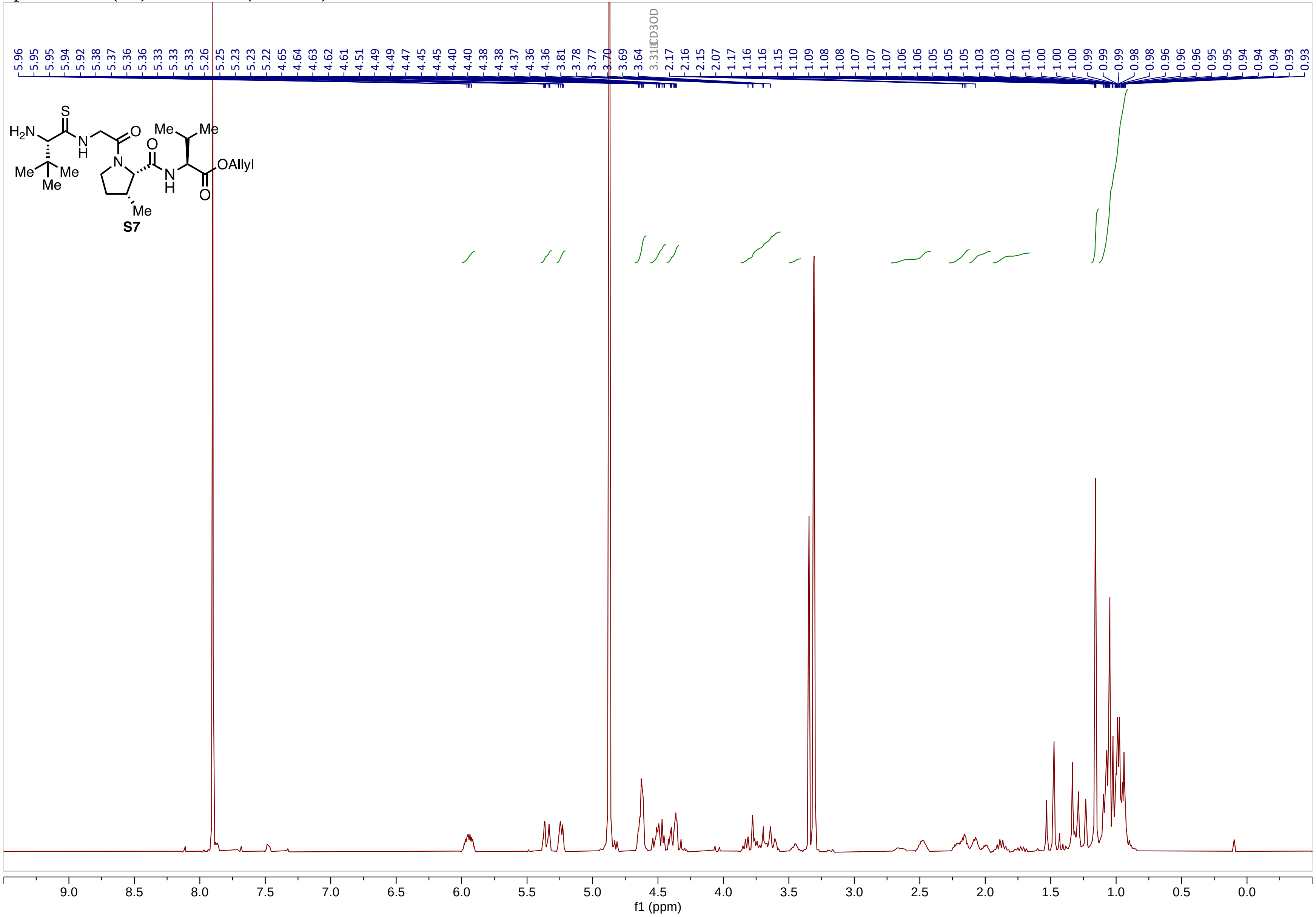

• TCbz-oligopeptide (**37**): ^{1}H NMR ($CDCl_3$)

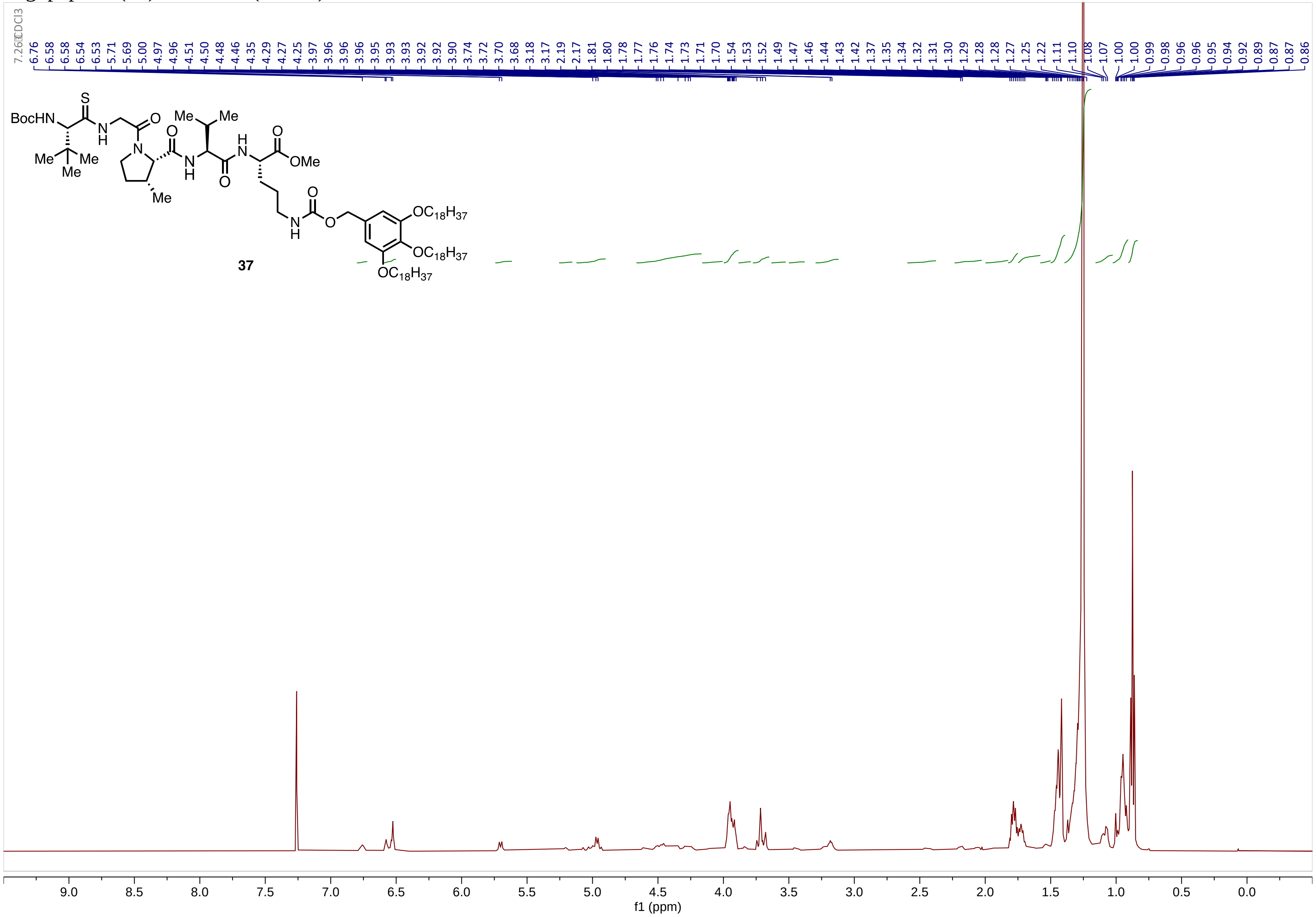

• TCbz-oligopeptide (**37**): ^{13}C NMR ($CDCl_3$)

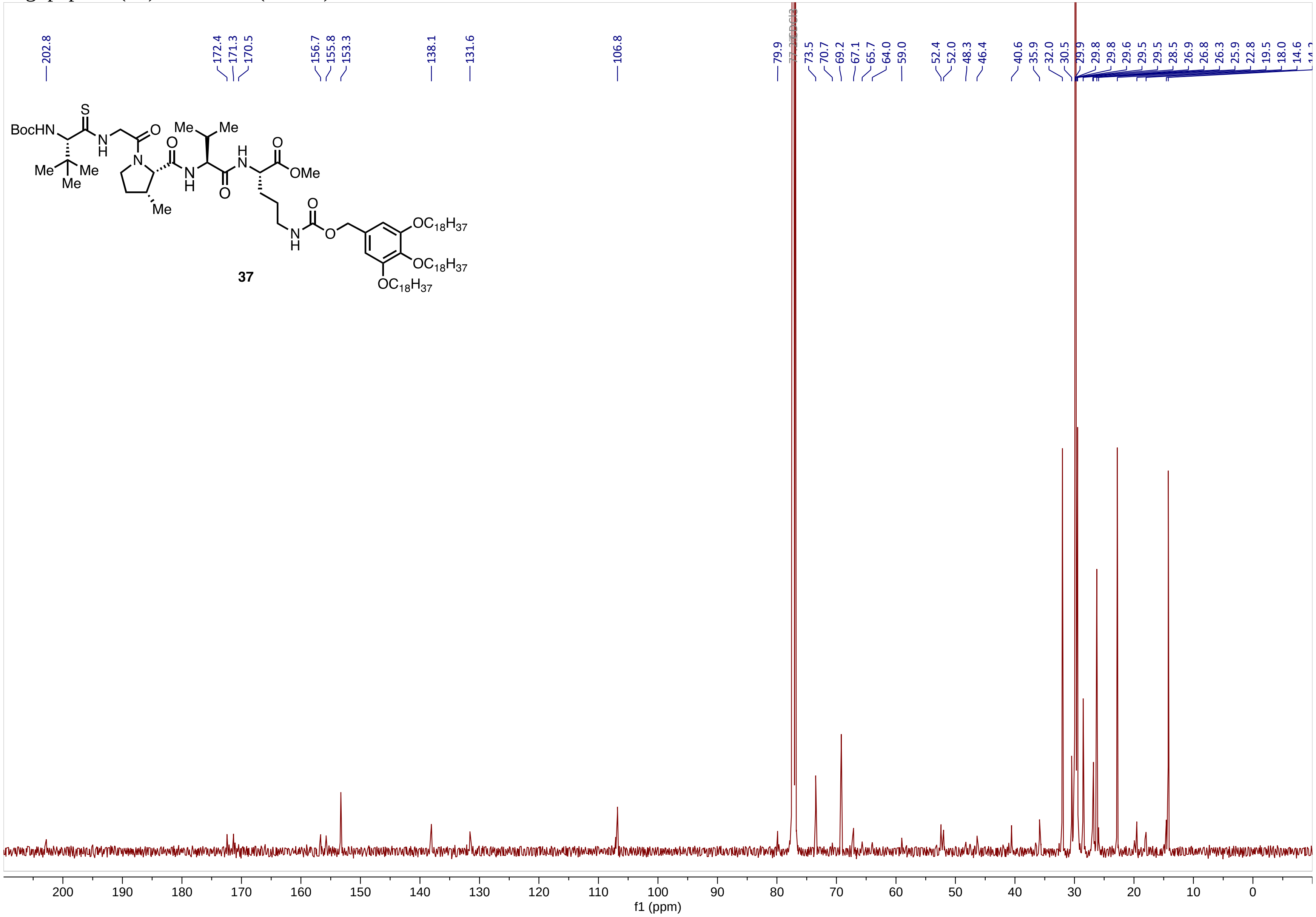

• TCbz-oligopeptide (**38**): ^{1}H NMR ($CDCl_3$)

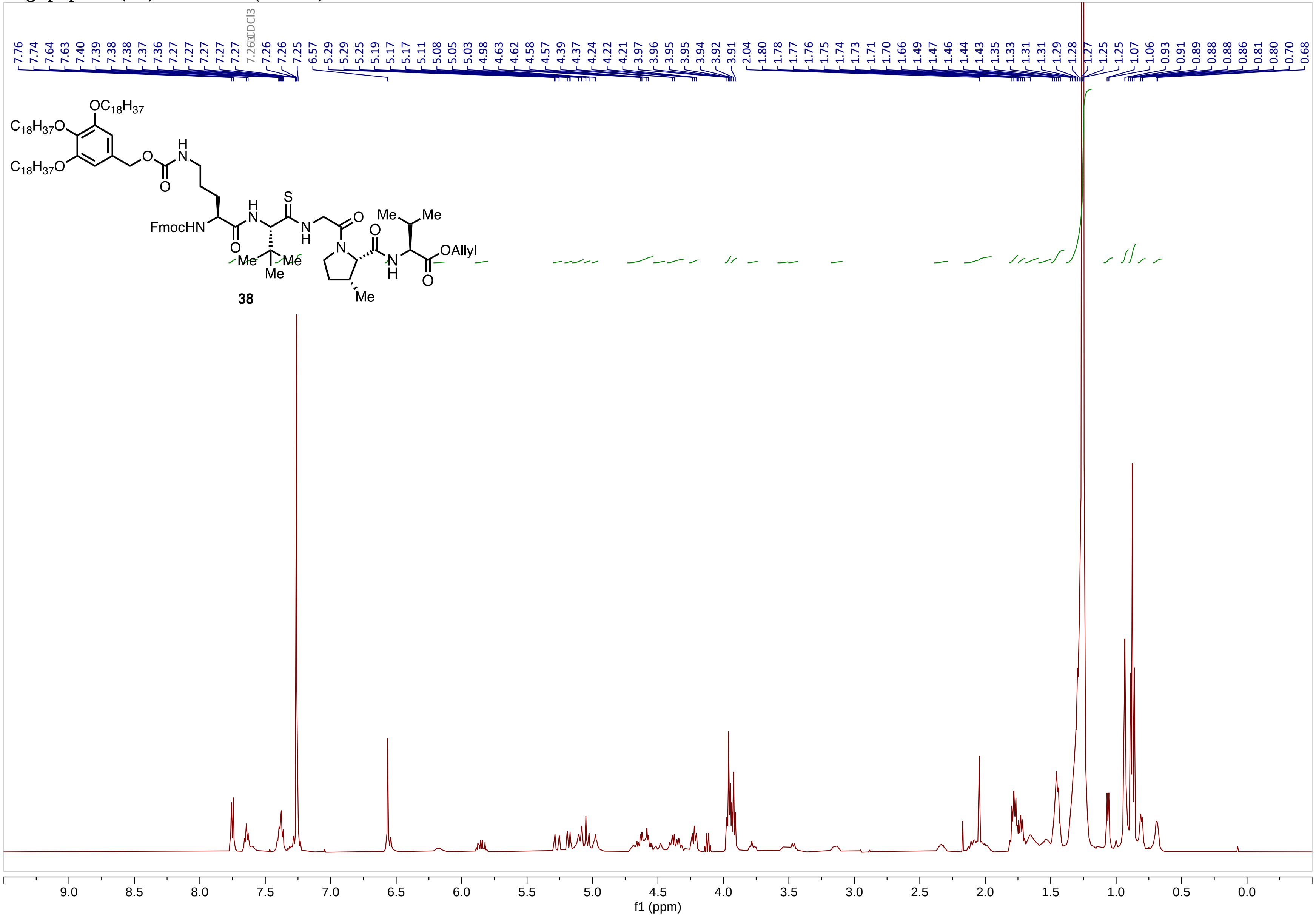

• TCbz-oligopeptide (**38**): ^{13}C NMR ($CDCl_3$)

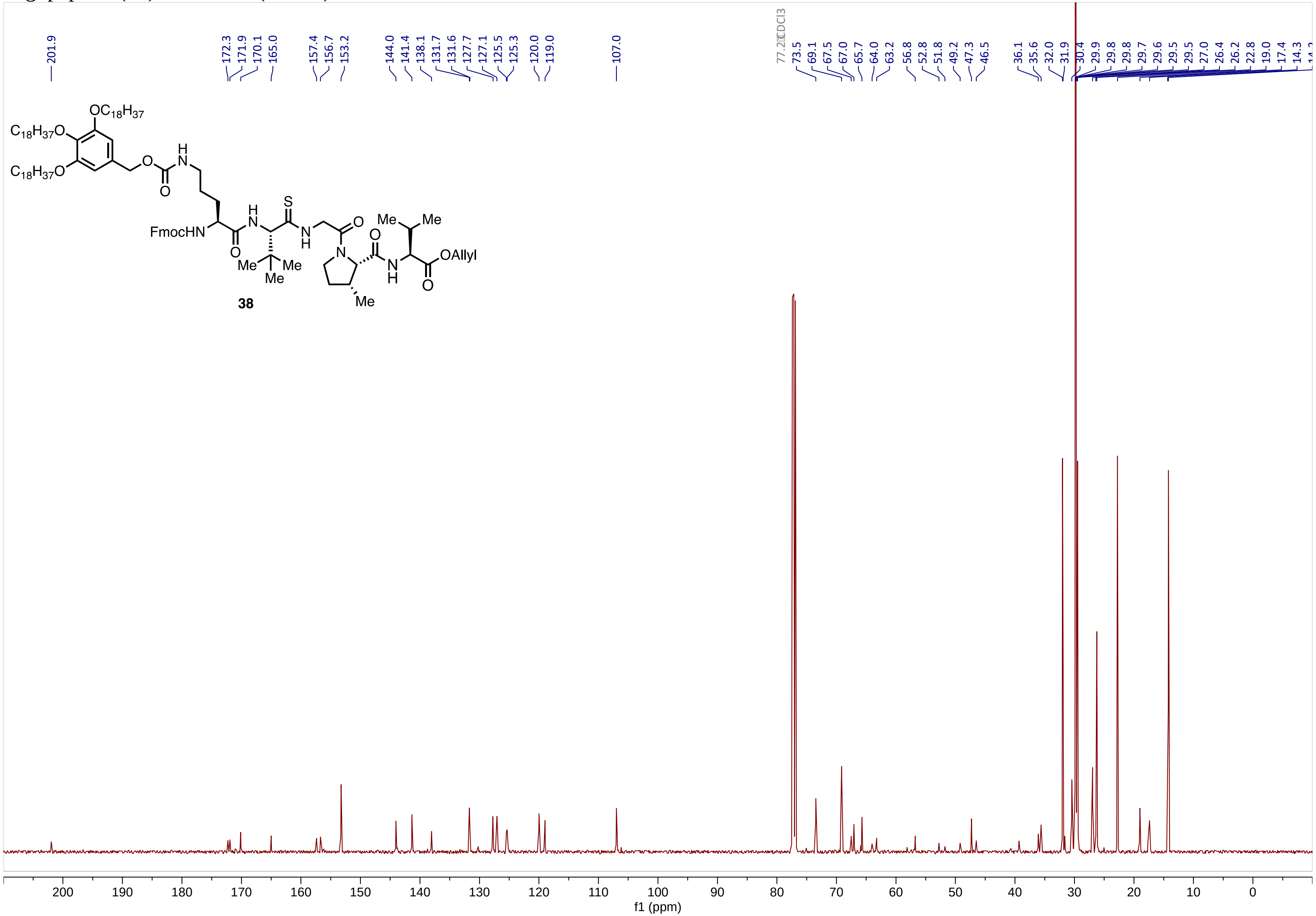

• TCbz-pentapeptide-OH (**43**): ^{1}H NMR ($CDCl_3$)

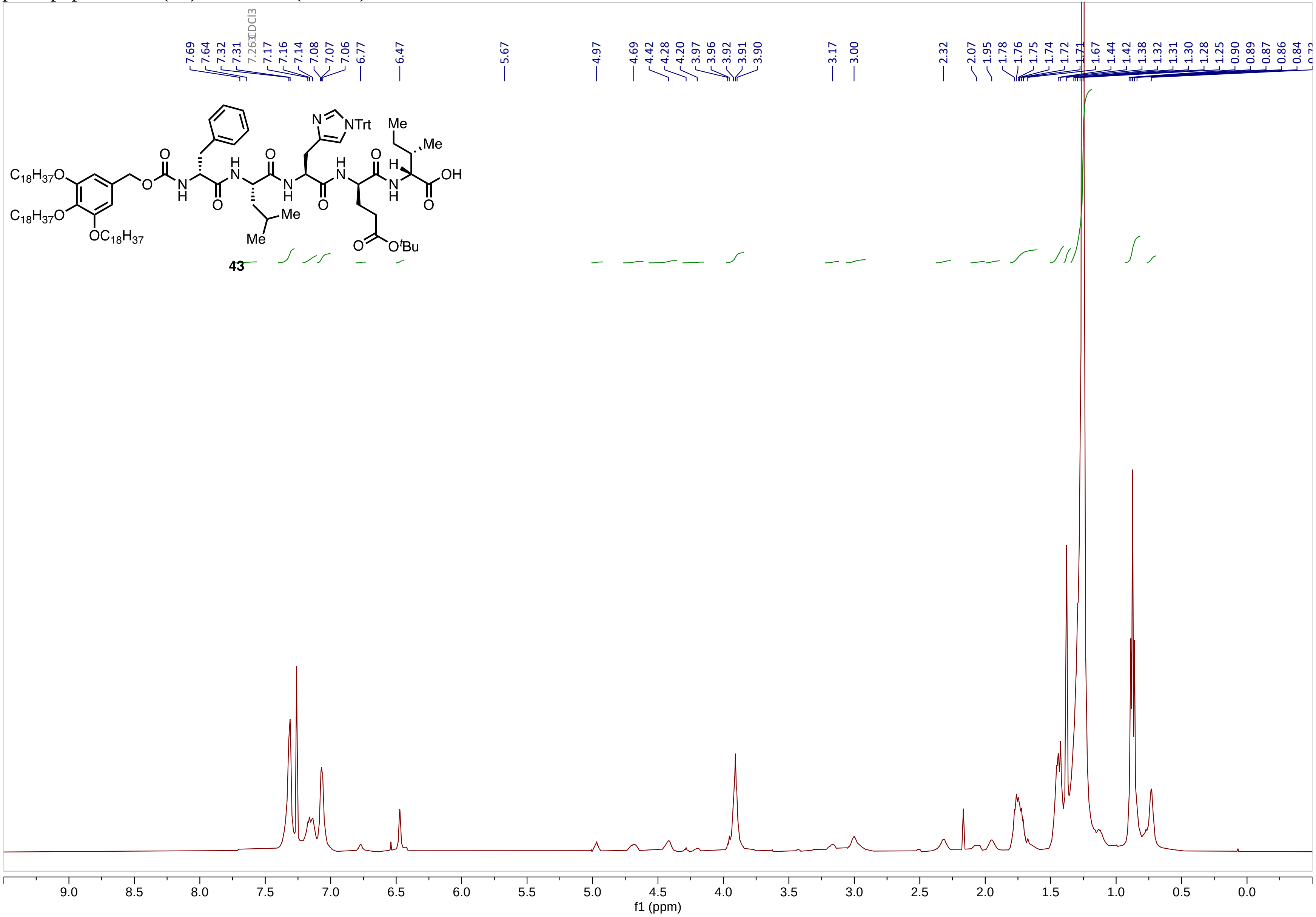

• TCbz-pentapeptide-OH (**43**): ^{13}C NMR ($CDCl_3$)

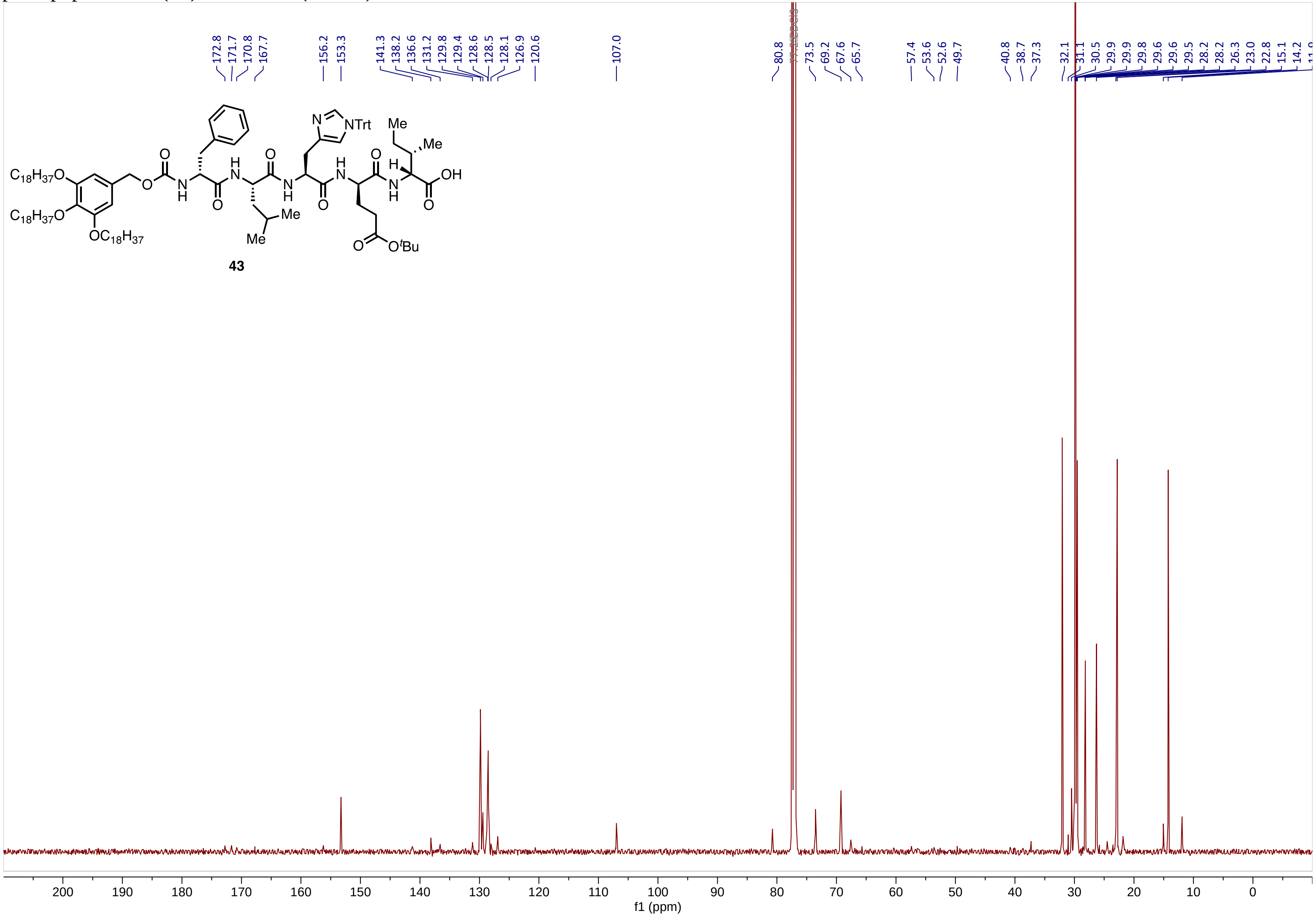

- TCbz-hexapeptide (**46**): ^{1}H NMR ($CDCl_3$)

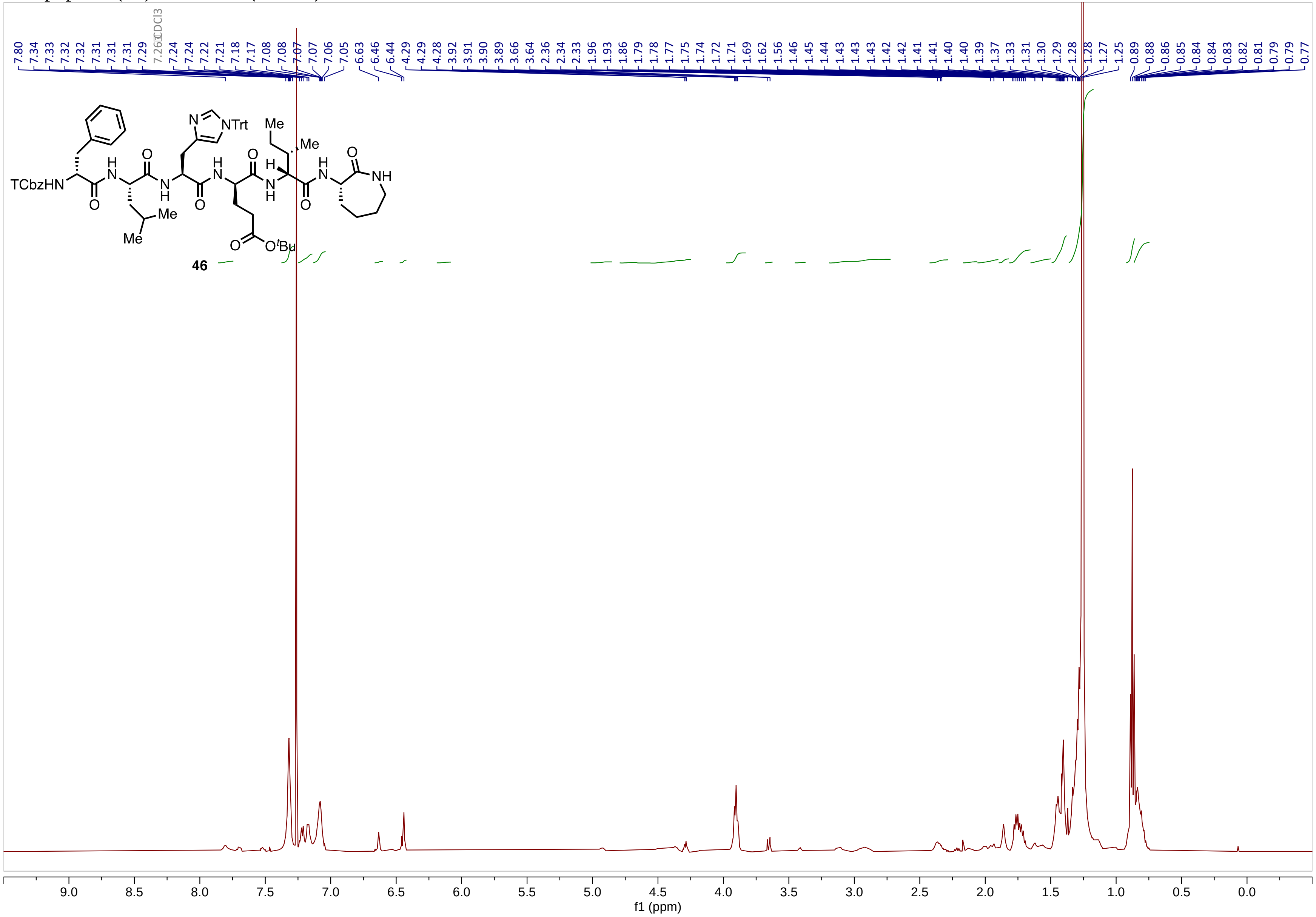

- TCbz-hexapeptide (**46**): ^{13}C NMR ($CDCl_3$)

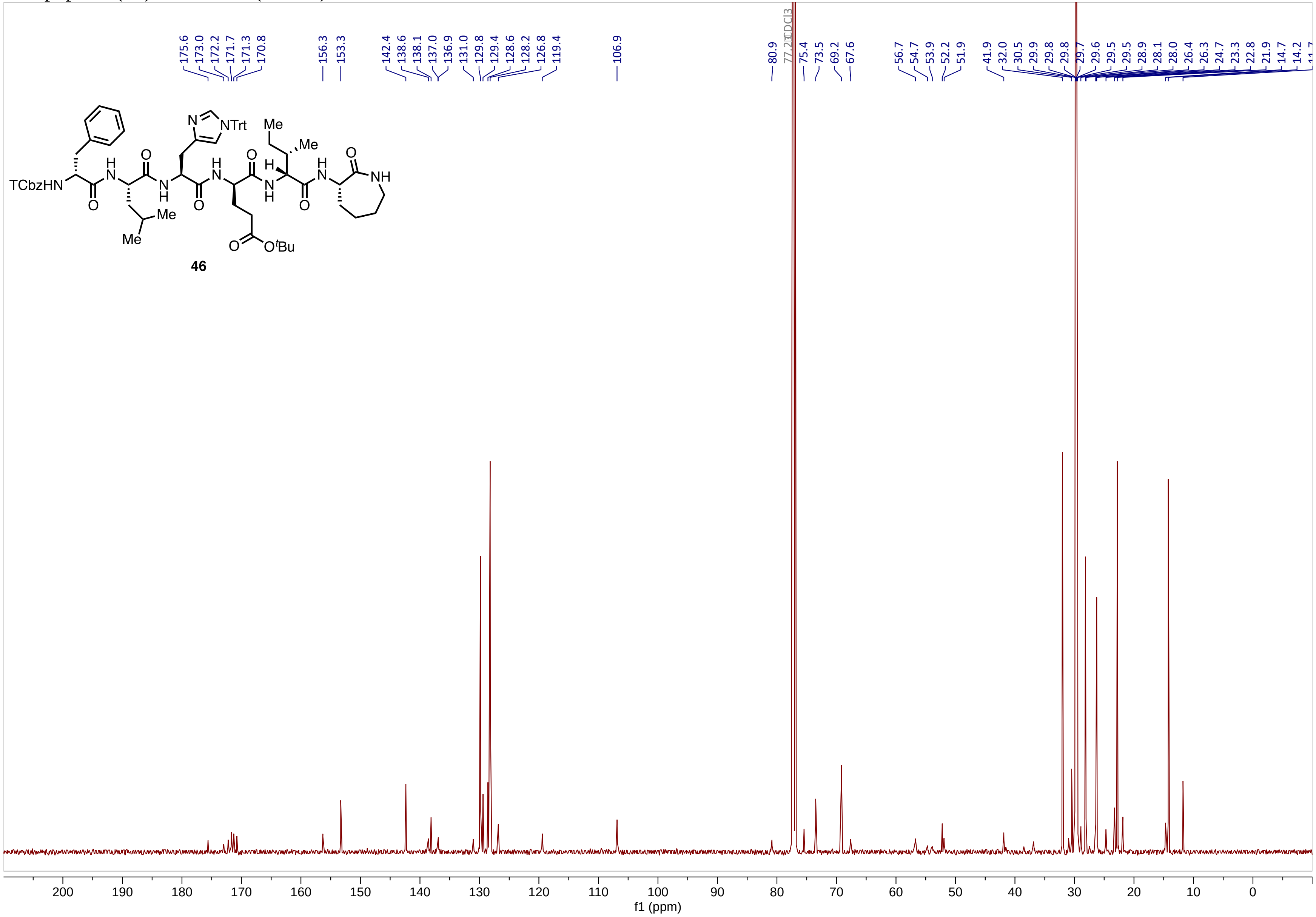

- Calpinactam (**39**): ^{1}H NMR $(CD_3)_2SO$ (two protons of the imidazole ring not detected)

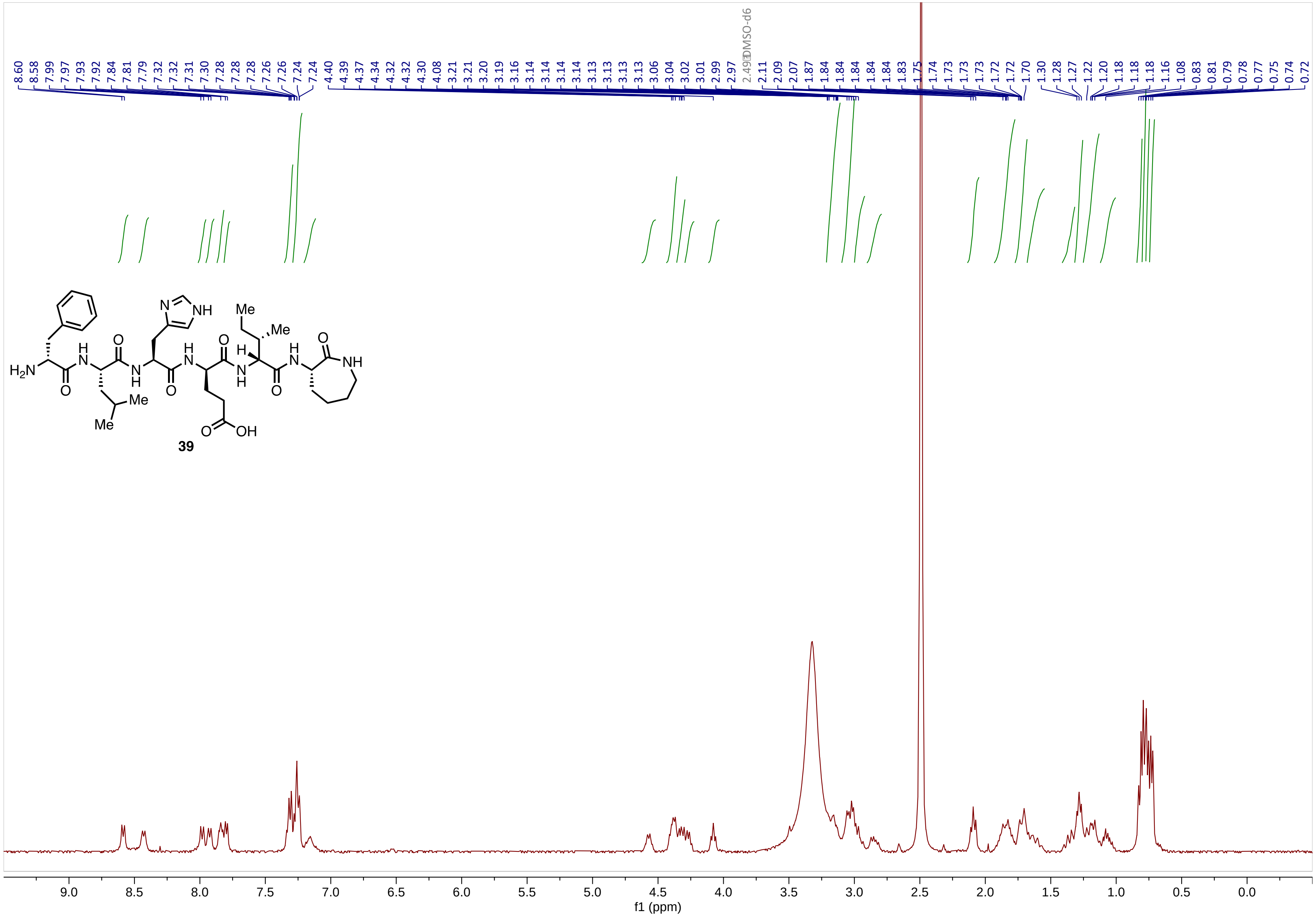

• Calpinactam (**39**): ^{1}H NMR $(CD_3)_2SO$

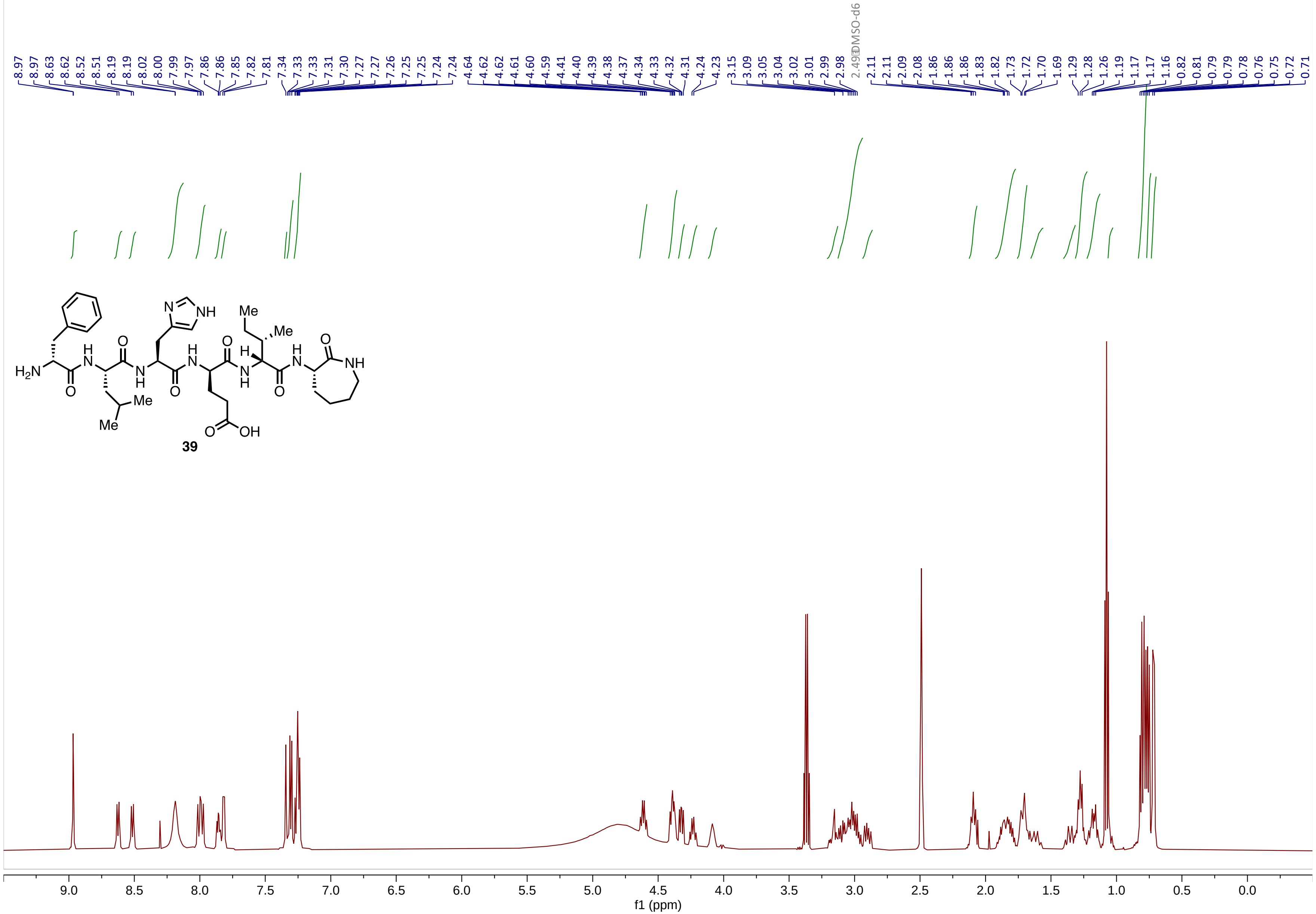

- Calpinactam (**39**): ^{13}C NMR $(CD_3)_2SO$

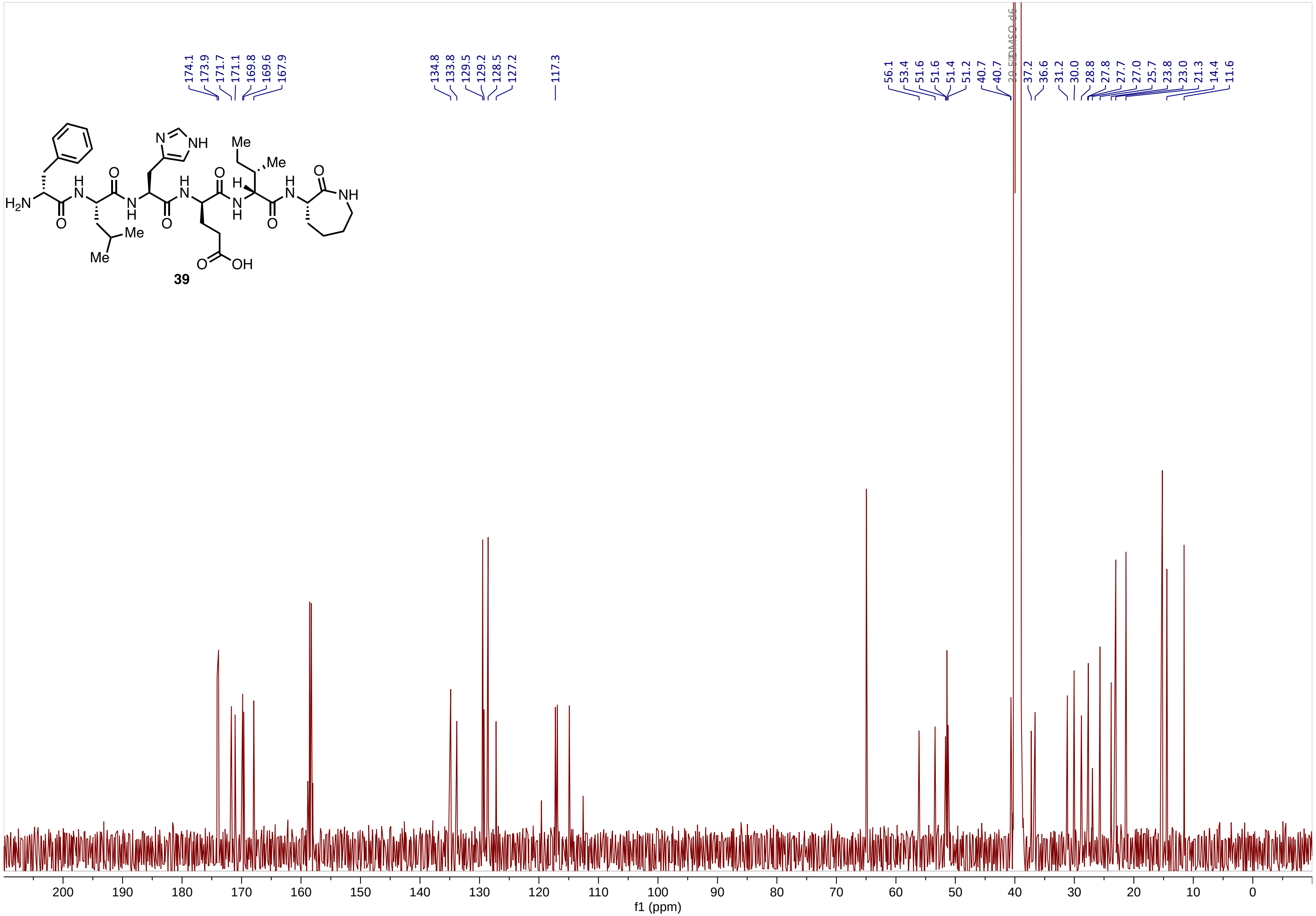

Supplement: SC-014-D3SC01432K-s001 [file SC-014-D3SC01432K-s001.pdf]
